# Derivation and validation of spirometry reference equations for Jordanian preschool children using GAMLSS modelling

**DOI:** 10.1016/j.jped.2026.101606

**Published:** 2026-09-06

**Authors:** Walid Al-Qerem

**Affiliations:** Department of Pharmacy, Faculty of Pharmacy, Al-Zaytoonah University of Jordan, Amman, Jordan

**Keywords:** Spirometry, Respiratory tract diseases, Child, Preschool, Reference values, Respiratory function tests

## Abstract

**Objective:**

To formulate spirometry reference equations that can be implemented on preschool-aged children in Jordan, while accounting for their age, sex and height.

**Methods:**

This two-phase Jordanian study derived sex-specific spirometry reference equations for healthy children aged 3–6 years and evaluated their temporal performance in an independent previously published Jordanian preschool cohort. In the 2025 model-development cohort, generalized additive models for location, scale and shape (GAMLSS) were fitted separately for boys and girls for forced expiratory volume in one second (FEV_1_), forced vital capacity (FVC), and FEV1/FVC, comparing Box-Cox Cole and Green distribution (BCCG), Box-Cox Power Exponential distributions (BCPE), and Normal families and selecting models using GAIC with diagnostic assessment by quantile residuals and worm plots. In the validation cohort described by Al-Qerem and Jarab, calibration and external performance were evaluated and compared against Global lung initiative equations (GLI-2012 Caucasian and GLI-2022 race-neutral equations).

**Results:**

The study included 1676 children: derivation cohort, n = 911, 461 boys; validation cohort, n = 765, 400 boys. BCPE models provided the best fit for FEV₁ and FVC in both sexes, whereas FEV₁/FVC was adequately captured by simpler models (boys: BCCG; girls: Normal). The validation phase indicated minimal systematic bias (intercepts near zero; slopes close to one), although FEV₁/FVC results were less precise. Jordanian and GLI-2012 yielded mean volume z-scores near zero, whereas GLI-2022 shifted volume estimates upward and reduced lower limit of normal yields, particularly for girls’ FEV₁.

**Conclusion:**

These first Jordanian preschool spirometry equations demonstrate good performance and support population-specific interpretation in clinical practice.

## Introduction

Asthma is common among preschool-aged children [1]. The most recent GBD estimates showed asthma prevalence rates of 5145.45 per 100,000 in Jordan and 5416.79 per 100,000 globally among children aged 2–4 years, and 5341.75 and 5706.15 per 100,000, respectively, among those aged 5–9 years [2]. Typically, the initial presentation of asthma symptoms occurs during preschool age [3]. In order to ensure timely treatment and minimize disease-related complications, it is crucial to identify the disease early. However, asthma diagnosis during preschool years presents a major challenge [4], since it is based mostly on subjective observations, including the presence of wheeze and symptom pattern [5].

Spirometry is among the most frequently performed tests to evaluate lung function and it can be implemented for children as young as 3 years old [6]. Given that lung function is influenced by age, gender, height, and ethnicity, these factors are recognized as the primary determinants of spirometry reference values [7]. Accurate interpretation of spirometry results is accomplished through comparing measured values to predicted values inferred from a healthy individual with corresponding anthropometric profile. Predicted values are determined using reference equations, produced based on a healthy population with characteristics that match the tested subject [8].

The Global Lung Initiative (GLI) developed the GLI 2012 spirometry reference equations based on data collected from 33 countries, to cover multiple ethnic groups, namely Caucasians, African-Americans, in addition to North and South East Asians [6]. Next, the race-neutral GLI 2022 equation was generated using the same data set, to improve the external validity [9]. Nevertheless, since non-Caucasian preschoolers were under-represented in the GLI dataset and the only included Arabs were from North Africa [6], both the GLI 2012 and the GLI 2022 prediction equations may not be suitable for preschool-aged children from the Middle East.

Evidence from validation studies, conducted in Jordan, reported that the GLI 2012 equations are not an optimal representation of preschool- and school-aged children, adolescents, and adults [10., 11., 12., 13.]. Furthermore, applying the GLI 2022 equation on Jordanian adults resulted in significant deviations [14].

As a result, population-specific spirometry reference equations were generated, to facilitate accurate diagnosis of respiratory diseases in Jordanian adults[15] and children [16]. However, to date, there are no reference equations produced specifically to be utilized for Jordanian preschoolers. Therefore, the objective of the present study is to formulate spirometry reference equations that can be implemented on preschool-aged children in Jordan, while accounting for their age, sex and height.

## Methods

### Study design

This study was conducted in two phases: Phase One (Derivation) and Phase Two (Validation/Calibration). Phase One aimed to derive preliminary spirometric reference metrics using a sample of healthy children, while Phase Two validated and calibrated these derivations using an independent cohort previously described by Al-Qerem and Jarab [10]. This time-separated validation design allowed evaluation of model performance across different recruitment periods, accounting for potential temporal changes in population characteristics, therefore offering a more rigorous assessment of calibration and predictive accuracy. Both phases adhered to internationally recognized spirometry and pediatric guidelines. The 2025 model-development cohort was collected between April and September 2025 from nurseries, kindergartens, and summer camps in Amman, Az-Zarqa, Irbid, Al-Mafraq, Al-Ramtha, and Madaba. The validation cohort was the previously published Al-Qerem and Jarab preschool dataset [10], collected between April and December 2019 from kindergartens, nursery schools, and summer camps in several Jordanian cities; the time-separated validation was intentional to evaluate performance across recruitment periods.

Ethical approval for the 2025 model-development cohort was obtained from the Al-Zaytoonah University of Jordan ethics committee (Ref. No. 15/12/2024–2025). The validation cohort was approved by the Al-Zaytoonah University of Jordan ethics committee (approval number 08/18/2018–2019), the Ministry of Education, and the Ministry of Health. Written informed consent was obtained from parents or guardians before testing; children received an age-appropriate explanation and proceeded only when they cooperated with the procedure. Parents completed a consent form explaining the spirometry test and a health questionnaire covering the child's health status, medication use, and prematurity status. Exclusion criteria included children with hay fever or any respiratory infection in the past 6 weeks, children who were born prematurely, since respiratory abnormalities are common in preterm-born preschool children [17], and children who had any symptom related to asthma during the past 12 months (including wheezing, dry cough at night not associated with a cold or chest infection, whistling in the chest, and sneezing, runny, or blocked nose not associated with a cold or flu).

### Sample size adequacy

No accepted a priori sample-size formula is available for deriving spirometric reference equations using GAMLSS. Therefore, sample-size adequacy was assessed using a model-complexity approach adapted from continuous-outcome prediction-model methodology [18,19]. Because the equations were developed separately for boys and girls, adequacy was evaluated within each sex-specific derivation group.

The calculation used the residual standard deviation precision criterion. This criterion requires n = 234 + p observations, where p is the number of model parameters. The constant 234 represents the residual degrees of freedom required to estimate the residual standard deviation with a multiplicative margin of error not exceeding 1.10; it is obtained from the chi-square distribution by solving sqrt(v / chi-square_0.025,v) ≤ 1.10, where v is the residual degrees of freedom. In the present analysis, p was defined conservatively as the ceiling of the fitted effective degrees of freedom from each final GAMLSS model.

Across the final sex-specific GAMLSS models, the effective parameter count ranged from 5 to 11, giving minimum required sample sizes ranging between 239 and 245 observations. The derivation cohort included 461 boys and 450 girls, exceeding this requirement for all sex-specific FEV1, FVC, and FEV1/FVC equations. For external validation, the study applied recommendation by Quanjer et al. that at least 150 males and 150 females are needed to validate spirometric reference equations and reduce the likelihood of differences caused by sampling error [20]. The independent validation cohort included 400 boys and 365 girls, exceeding this validation threshold in both sexes.

### Anthropometric measurements

Anthropometric parameters were collected using standardized equipment. Body weight was measured to the nearest 0.1 kg using a calibrated electronic scale, and height was measured to the nearest centimeter using a stadiometer. Body Mass Index (BMI) was calculated using Quetelet’s index (kg/m²) [21].

### Pulmonary function measurements

Spirometry testing followed the American Thoracic Society/European Respiratory Society (ATS/ERS) guidelines for preschool-aged children [22]. All children received detailed instructions and practice trials to ensure familiarity with the forced expiratory maneuver. A Minispir computer-based spirometer was calibrated and checked according to manufacturer instructions and used consistently across participants to minimize measurement variability. Children were tested in a seated position while wearing nose clips. At least three maneuvers were attempted; in which children were instructed to inhale deeply and then exhale as forcefully and rapidly as possible. The following spirometric parameters were tested: FEV1 (Forced Expiratory Volume at one second), FVC (Forced Vital Capacity), and FEV1/FVC. For the maneuver to be accepted, the flow-volume curve must demonstrate a rapid rise to peak flow followed by a smooth, gradually declining expiratory curve, and the exhalation must be stopped at >10% of peak flow; ensuring the maneuver was not terminated too early. Additionally, the maneuvers were repeated until at least two acceptable curves were obtained, with the differences between the highest and second-highest FVC and FEV₁ values kept within 0.1 L or 10%, whichever was larger, to ensure repeatability. Disposable turbines were used for each child, and quality-control review followed ATS/ERS preschool acceptability and repeatability criteria within the device software.

### Statistical analysis

All analyses were stratified by sex. Model development was conducted in the derivation dataset, which included children aged 3–6 years with high-quality spirometry. For each spirometric outcome (FEV₁, FVC, and FEV₁/FVC), a set of candidate models was fitted using the GAMLSS framework, evaluating Box–Cox Cole–Green (BCCG), Box–Cox Power Exponential (BCPE), and Normal distributions under alternative parameterizations of age and height. Competing models were compared using the generalized Akaike Information Criterion (GAIC), with the penalty term defined as k = log(n(parameter)), where n(parameter) is the number of non-missing observations for the specific outcome and sex. For each outcome, the model with the lowest GAIC was selected as the Jordanian reference equation. Model adequacy was assessed using quantile residuals, worm plots, and distributional diagnostics.

In the derivation dataset, predicted values, lower limits of normal (LLNs; defined as z = –1.645), z-scores, and percent-predicted values were generated from each selected equation to evaluate internal performance and to visualize model behavior. Age-specific predicted values and LLNs for the Jordanian models were plotted against GLI 2012 Caucasian and GLI 2022 race-neutral equations to illustrate differences in age-related trajectories.

External validation was performed using the previously published Al-Qerem and Jarab cohort of 765 healthy Jordanian children aged 3–5 years (54% boys), recruited from kindergartens, nursery schools, and summer camps in multiple Jordanian cities [10]. For each child, predicted values, LLNs, and z-scores were computed using the final Jordanian equations and compared with the corresponding quantities derived from the GLI 2012 Caucasian and GLI 2022 race-neutral equations (as provided by the official GLI calculator). Validation analyses included comparison of mean z-scores, standard deviations of z-scores, mean percent-predicted values, and the proportion of children with values below the LLN for each reference system. Calibration of the Jordanian equations in the validation cohort was assessed by regressing observed spirometric values on their predicted values; intercepts approaching zero and slopes approaching one were interpreted as evidence of good calibration. All analyses were performed in R (version 4.5.3) using the gamlss*,* rspiro*,* dplyr*,* and ggplot2 packages.

## Results

The study included a total of 1676 participants. Table 1 summarizes age, height, weight, FEV1, FVC, and FEV1/FVC by sex in the 2025 model-development/testing cohort and the Al-Qerem and Jarab validation cohort. The two cohorts had similar sex distributions (50.6%vs 52.3% boys), Within the 2025 cohort, boys and girls did not differ significantly in age, height, weight, FEV1, FVC, or FEV1/FVC; in the validation cohort, boys had slightly higher FEV1 and FVC than girls (p = 0.013 and p = 0.044, respectively), with no significant sex differences in age, height, weight, or FEV1/FVC.

**Table 1:** Participant characteristics by cohort and sex.

Phase 1 (derivation)	Characteristic	Boys (N = 461)	Girls (N = 450)	P-value
	Age (years)	4.31 (0.87)	4.33 (0.90)	0.8
	Height (cm)	103.65 (9.13)	104.10 (7.18)	0.6
	Weight (kg)	16.56 (3.89)	16.34 (3.82)	0.7
	FEV₁ (L)	0.97 (0.24)	0.95 (0.19)	0.14
	FVC (L)	1.04 (0.27)	1.02 (0.22)	0.3
	FEV₁/FVC	0.94 (0.05)	0.93 (0.05)	0.2
Phase 2 (validation/ calibration)	Characteristic	Boys (N = 400)	Girls (N = 365)	P-value
	Age (years)	4.55 (0.80)	4.65 (0.75)	0.09
	Height (cm)	107.94 (7.96)	107.56 (7.49)	0.50
	Weight (kg)	19.06 (3.17)	18.77 (3.54)	0.24
	FEV1 (L)	1.06 (0.23)	1.02 (0.21)	0.01
	FVC (L)	1.13 (0.24)	1.10 (0.23)	0.04
	FEV1/FVC	0.94 (0.04)	0.93 (0.05)	0.11

FEV₁, forced expiratory volume in 1 s; FVC, forced vital capacity; FEV₁/FVC, ratio of FEV₁ to FVC.

Across all three spirometric outcomes, model comparison using Generalized Akaike Information Criterion (GAIC) with the penalty set to (k = log(length(data$parameter)) consistently favored more flexible distributions for volume outcomes and simpler formulations for the FEV₁/FVC ratio. For FEV₁, the four-parameter BCPE model with log-linked mean and age modelled with penalized B-splines in all distributional parameters (µ, σ, ν, τ) clearly outperformed all alternative specifications in both boys and girls (ΔGAIC ≥ 47.7 and ≥ 48.6, respectively), indicating substantial gains from allowing age-dependent skewness and kurtosis in preschool flow–volume data (Table 2). A very similar pattern emerged for FVC, where the same BCPE formulation ranked first in both sexes; in boys the improvement over the next-best BCCG models was moderate but still clearly above conventional decision thresholds (ΔGAIC ≈ 13–14), while in girls the BCPE model was favored but the gap to simpler log-link BCCG models was smaller (ΔGAIC ≈ 4–5), suggesting that extra distributional flexibility is beneficial but less critical than for FEV₁ (Table 3). In contrast, for the FEV₁/FVC ratio the best-fitting family differed and the advantage of simpler models emerged: in boys, the preferred model was the BCCG distribution with log-linked mean and simple log–log height–age parametrization, with all more flexible spline-based and BCPE variants showing only modestly worse GAIC values; in girls, the normal model with log-linked mean and smooth age-dependent variance provided the best fit, with several BCCG formulations essentially indistinguishable (ΔGAIC < 0.5) (Table 4).

**Table 2:** Model comparison for FEV1 reference equations in preschool children.

Outcome	Sex	Model specification	df	GAIC (k = log(length))	ΔGAIC	Rank
FEV1	Boys	BCPE, log μ; pb(log *age*) in μ, σ, ν, τ	10.22	−639.377	0.000	1
		BCCG, log μ; log(*height*), log(*age*)	5.00	−591.701	47.676	2
		BCCG, log μ; pb(log *age*) in μ & σ, ν fixed	5.59	−585.119	54.258	3
		BCCG, identity μ; pb(log *age*) in μ, σ; ν fixed	5.07	−578.317	61.060	4
		BCCG, log μ; height + pb(log *age*); σ, ν ∼ log(*age*)	8.19	−569.702	69.675	5
		BCCG, identity μ; pb(log *age*) in μ, σ; ν ∼ log(*age*)	7.07	−569.464	69.913	6
		Normal, log μ; pb(log *age*) in σ	5.32	−565.440	73.937	7
	Girls	BCPE, log μ; pb(log *age*) in μ, σ, ν, τ	9.18	−683.763	0.000	1
		BCCG, log μ; log(*height*), log(*age*)	5.00	−635.184	48.579	2
		BCCG, log μ; pb(log *age*) in μ & σ, ν fixed	5.06	−633.586	50.177	3
		BCCG, identity μ; pb(log *age*) in μ, σ; ν fixed	6.06	−628.230	55.533	4
		Normal, log μ; pb(log *age*) in σ	5.06	−625.271	58.492	5
		BCCG, log μ; height + pb(log *age*); σ, ν ∼ log(*age*)	7.07	−619.919	63.844	6
		BCCG, identity μ; pb(log *age*) in μ, σ; ν ∼ log(*age*)	8.11	−617.271	66.492	7

GAIC, Generalized Akaike Information Criterion; df, degrees of freedom; BCPE, Box–Cox power exponential; BCCG, Box–Cox Cole–Green; μ, location (mean or median); σ, scale; ν and τ, shape parameters; FEV₁, forced expiratory volume in 1 s.

**Table 3:** Model comparison for FVC reference equations in preschool children.

Outcome	Sex	Model specification	df	GAIC (k = log(length))	ΔGAIC	Rank
FVC	Boys	BCPE, log μ; pb(log *age*) in μ, σ, ν, τ	9.10	−555.667	0.000	1
		BCCG, log μ; log(*height*), log(*age*)	5.00	−542.012	13.656	2
		BCCG, log μ; pb(log *age*) in μ & σ, ν fixed	5.06	−541.821	13.846	3
		BCCG, log μ; height + pb(log *age*); σ, ν ∼ log(*age*)	7.06	−522.943	32.725	4
		BCCG, identity μ; pb(log *age*) in μ, σ; ν fixed	5.06	−517.249	38.418	5
		Normal, log μ; pb(log *age*) in σ	5.12	−514.682	40.985	6
		BCCG, identity μ; pb(log *age*) in μ, σ; ν ∼ log(*age*)	7.06	−509.640	46.028	7
	Girls	BCPE, log μ; pb(log *age*) in μ, σ, ν, τ	9.08	−507.574	0.000	1
		BCCG, log μ; log(*height*), log(*age*)	5.00	−503.213	4.361	2
		BCCG, log μ; pb(log *age*) in μ & σ, ν fixed	5.06	−502.176	5.397	3
		BCCG, identity μ; pb(log *age*) in μ, σ; ν fixed	6.13	−495.753	11.820	4
		Normal, log μ; pb(log *age*) in σ	5.05	−489.576	17.998	5
		BCCG, log μ; height + pb(log *age*); σ, ν ∼ log(*age*)	7.05	−489.392	18.182	6
		BCCG, identity μ; pb(log *age*) in μ, σ; ν ∼ log(*age*)	8.13	−485.340	22.233	7

GAIC, Generalized Akaike Information Criterion; BCPE, Box–Cox power exponential; BCCG, Box–Cox Cole–Green; μ, location (mean or median); σ, scale; ν and τ, shape parameters; FVC, forced vital capacity.

**Table 4:** Model comparison for FEV1/FVC reference equations in preschool children.

Outcome	Sex	Model specification	df	GAIC (k = log(length))	ΔGAIC	Rank
FEV1/FVC	Boys	BCCG, log μ; log(*height*), log(*age*)	5.00	−1456.910	0.000	1
		BCCG, log μ; height + pb(log *age*); σ, ν ∼ log(*age*)	7.33	−1451.209	5.700	2
		BCCG, identity μ; pb(log *age*) in μ, σ; ν ∼ log(*age*)	7.36	−1449.197	7.713	3
		Normal, log μ; pb(log *age*) in σ	5.28	−1446.174	10.735	4
		BCCG, identity μ; pb(log *age*) in μ, σ; ν fixed	5.33	−1445.146	11.763	5
		BCCG, log μ; pb(log *age*) in μ & σ, ν fixed	5.29	−1445.100	11.810	6
		BCPE, log μ; pb(log *age*) in μ, σ, ν, τ	9.34	−1440.402	16.507	7
	Girls	Normal, log μ; pb(log *age*) in σ	5.57	−1381.665	0.000	1
		BCCG, log μ; pb(log *age*) in μ & σ, ν fixed	5.61	−1381.407	0.259	2
		BCCG, identity μ; pb(log *age*) in μ, σ; ν fixed	5.64	−1381.228	0.437	3
		BCPE, log μ; pb(log *age*) in μ, σ, ν, τ	9.33	−1375.891	5.774	4
		BCCG, log μ; height + pb(log *age*); σ, ν ∼ log(*age*)	7.88	−1374.889	6.776	5
		BCCG, identity μ; pb(log *age*) in μ, σ; ν ∼ log(*age*)	7.92	−1374.671	6.994	6
		BCCG, log μ; log(*height*), log(*age*)	5.00	−1374.294	7.371	7

GAIC, Generalized Akaike Information Criterion; df, degrees of freedom; BCPE, Box–Cox power exponential; BCCG, Box–Cox Cole–Green; μ, location (mean or median); σ, scale; ν and τ, shape parameters; FEV₁, forced expiratory volume in 1 s; FVC, forced vital capacity; FEV₁/FVC, ratio of FEV₁ to FVC.

**Fig. 1:**
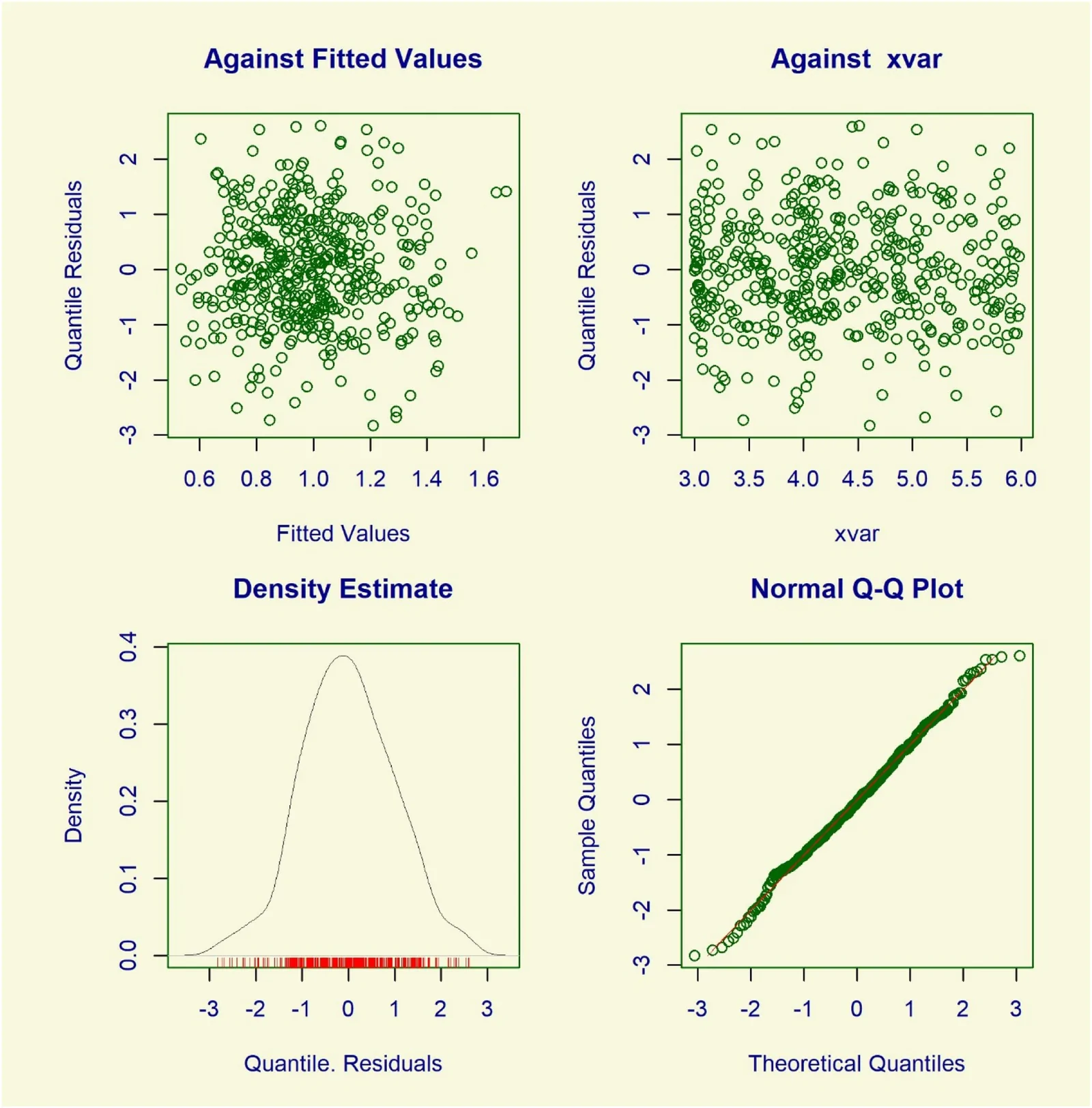
Diagnostic plots for FEV1 in boys.

Diagnostic plots for the selected sex-specific models showed no evidence of major model misspecification (Figure 1) (See Supplementary Figures A1 to A6). Quantile residuals for FEV₁ and FVC under the BCPE formulation were symmetrically distributed around zero, with approximately constant variance across fitted values and age, and without discernible trends or clustering. Kernel density estimates were close to unimodal, symmetric shapes, and normal Q–Q plots showed only minor departures from linearity in the extreme tails. For the FEV₁/FVC ratio, the BCCG model in boys and the normal model in girls produced similarly well-behaved residuals, with random scatter against fitted values and age and Q–Q plots closely following the theoretical line.

**Fig. 2:**
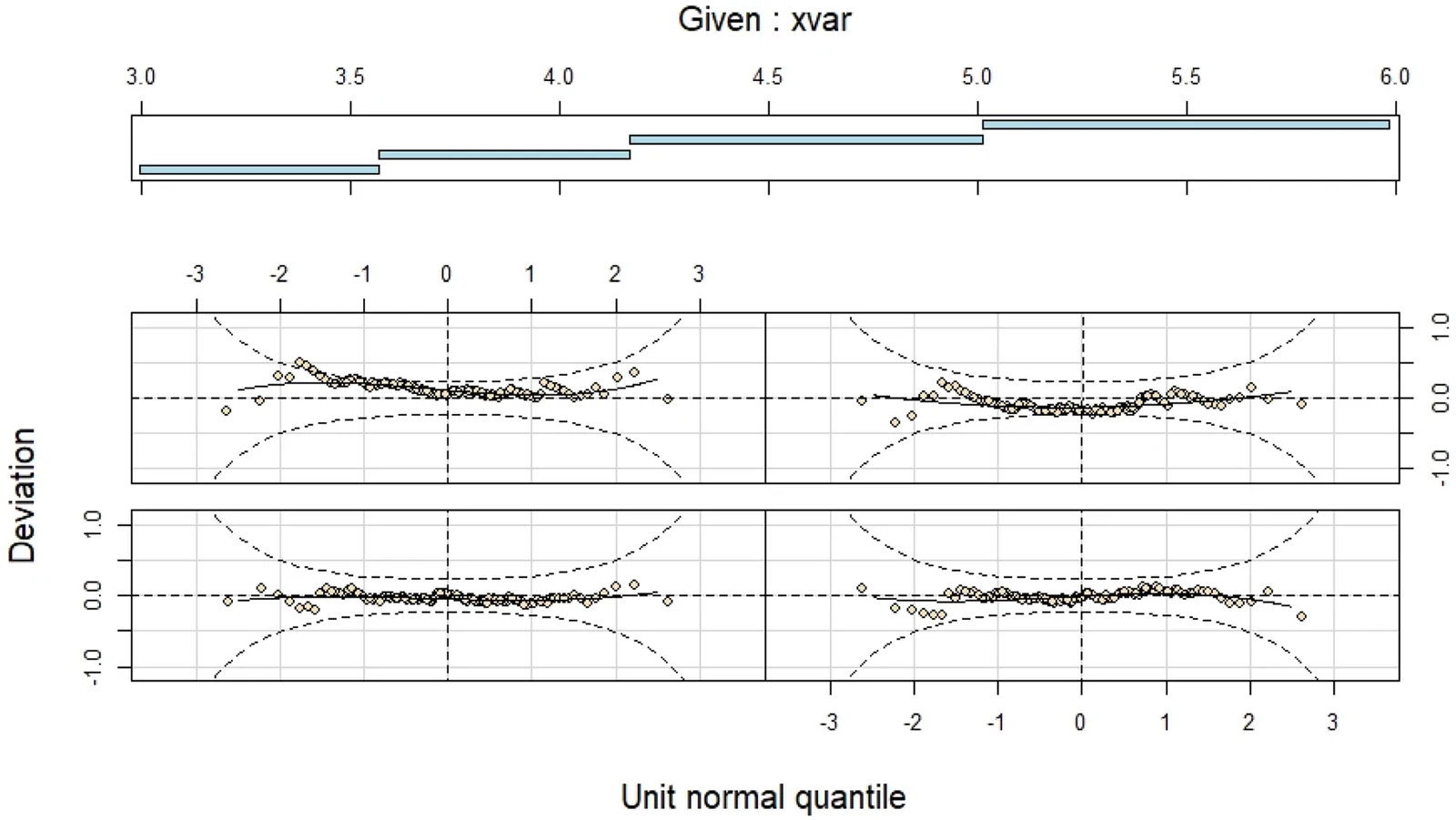
Worm plots for FEV₁ in boys fitted with the BCPE model.

Worm plots stratified by age bands (3–6 years) provided additional support for the adequacy of the selected sex-specific models (Figure 2) (See Supplementary Figures A6-A10). For FEV₁ and FVC, the BCPE formulations in boys and girls showed small, largely random deviations around zero across all age intervals, with most points lying within the 95% reference curves and only mild, smooth curvature in the youngest and oldest bands. These patterns suggest that any residual age-related misspecification in the location, scale, skewness, or kurtosis of the distributions is limited and unlikely to be of practical importance. For the FEV₁/FVC ratio, the BCCG model in boys and the normal model in girls also produced predominantly flat worm plots, with only modest departures in the extreme quantiles and no consistent systematic trends across age groups. The final equations are displayed in Table 5, and a structured equation file was prepared to facilitate transparent use of the final sex-specific models. Normative reference values for FEV₁, FVC, and FEV₁/FVC were derived from the final sex-specific models, providing height- and age-specific predicted values and LLN across the pediatric growth range (Supplementary Tables A1-A3). A supplementary Shiny application was also developed to allow users to enter age, height, sex, FEV₁, and FVC values and obtain the corresponding Jordanian predicted values, LLN, z-scores, and percent-predicted estimates.

**Table 5:** Reference equations for calculating spirometry parameters.

Outcome	Sex	Family	Mu	Sigma	Nu	Tau
FEV1 (L)	Boys	BCPE	EXP(−10.33,131 + 2.17,686 * log(height) + 0.13,810 * pb(log(age)))	EXP(−1.87,182 - 0.14,824 * pb(log(age)))	2.35,805 + 0.12,931 * log(age)	EXP(2.67,502 - 0.30,225 * log(age))
	Girls	BCPE	EXP(−9.49,377 + 1.98,595 * log(height) + 0.15,371 * pb(log(age)))	EXP(−2.19,935 + 0.02,605 * pb(log(age)))	2.22,618 + 0.29,580 * log(age)	EXP(1.85,226 + 0.03,062 * log(age))
FVC (L)	Boys	BCPE	EXP(−11.42,914 + 2.45,484 * log(height) + 0.04,299 * pb(log(age)))	EXP(−2.02,564 - 0.01,954 * pb(log(age)))	3.14,792 - 1.43,619 * log(age)	EXP(0.98,244 + 0.41,707 * log(age))
	Girls	BCPE	EXP(−9.78,720 + 2.06,328 * log(height) + 0.14,625 * pb(log(age)))	EXP(−1.87,539 - 0.09,759 * pb(log(age)))	−0.30,627 + 0.78,914 * log(age)	EXP(−0.39,396 + 1.21,796 * log(age))
FEV1/FVC	Boys	BCCG	EXP(1.05,258 - 0.25,912 * log(height) + 0.06,054 * log(age))	EXP(−2.98,943)	4.26,297	-
	Girls	NO	EXP(0.04,880 - 0.02,653 * log(height) + 0.00,294 * pb(log(age)))	EXP(−1.99,551 - 0.69,048 * pb(log(age)))	-	-

BCPE, Box–Cox power exponential; BCCG, Box–Cox Cole–Green; NO, normal distribution; μ, location (mean or median); σ, scale; ν and τ, shape parameters; FEV₁, forced expiratory volume in 1 s; FVC, forced vital capacity; FEV₁/FVC, ratio of FEV₁ to FVC.

Supplementary Table S1 displays the calibration analyses in the temporal Al-Qerem and Jarab validation cohort which showed that the Jordanian equations were well aligned with observed FEV₁ and FVC across sexes. For FEV₁, intercepts were close to zero with narrow confidence intervals and slopes close to unity in both boys (intercept 0.03, 95% CI −0.05 to 0.11; slope 0.97, 95% CI 0.90 to 1.03) and girls (intercept −0.02, 95% CI −0.10 to 0.07; slope 1.01, 95% CI 0.93 to 1.09), indicating minimal systematic bias and good calibration across the range of predicted values. FVC calibration was similarly acceptable, with intercepts near zero and slopes only slightly below one in boys (intercept 0.09, 95% CI 0.02 to 0.17; slope 0.91, 95% CI 0.84 to 0.97) and essentially unity in girls (intercept 0.02, 95% CI −0.08 to 0.12; slope 0.99, 95% CI 0.90 to 1.08), suggesting a modest tendency towards underestimation at higher predicted FVC volumes in boys but overall good agreement. Calibration of the FEV₁/FVC ratio was less precise, with wide confidence intervals for both intercept and slope, particularly in girls. Agreement was further examined using Bland-Altman analysis. Predicted-raw bias was small for FEV1 (0.007 L in boys and 0.005 L in girls) and FVC (0.013 L in boys and −0.008 L in girls), with 95% limits of agreement shown in Supplementary Table S1. For FEV1/FVC, bias was −0.009 (95% LoA, −0.100 to 0.082) in boys and −0.002 (−0.091 to 0.088) in girls, while the Jordanian FEV1/FVC z-score distribution in girls was close to zero (0.04 [0.89], 95% CI −0.05 to 0.14). These findings indicate minimal systematic bias despite wider regression uncertainty for the ratio.

In the validation cohort, the Jordanian equations and GLI 2012 Caucasian set produced broadly similar distributions of FEV₁ and FVC, with mean z-scores close to zero and mean percent predicted values around 100% in both sexes (e.g. FEV₁ z-score 0.00 and 0.08 in boys, 0.01 and 0.12 in girls for GLI 2012 Caucasian and Jordanian, respectively). By contrast, the GLI 2022 race-neutral equations yielded consistently higher mean z-scores and percent predicted for volumes, particularly in girls (FEV₁ z-score 0.29 and 103.8% predicted; FVC z-score 0.28 and 104.1% predicted), indicating an upward shift in predicted FEV₁ and FVC relative to both GLI 2012 Caucasian and the Jordanian equations. The proportion of children classified below LLN for FEV₁ and FVC was slightly lower with GLI 2022 than with the other two systems, especially among girls (e.g. FEV₁ < LLN 1.9% vs 4.4% for GLI 2012 Caucasian and Jordanian). In contrast, FEV₁/FVC showed close agreement between all three reference systems, with mean z-scores approximating zero, mean percent predicted values around 100%, and proportions below LLN mostly between 3% and 5%, apart from a somewhat lower LLN yield for the Jordanian ratio in girls (1.4%). These findings suggest that in an independent preschool cohort from the same setting, the Jordanian equations calibrate more closely to GLI 2012 Caucasian than to the GLI 2022 race-neutral set for absolute volumes, while agreement across reference systems remains high for the FEV₁/FVC ratio (Supplementary Table S2).

**Fig. 3:**
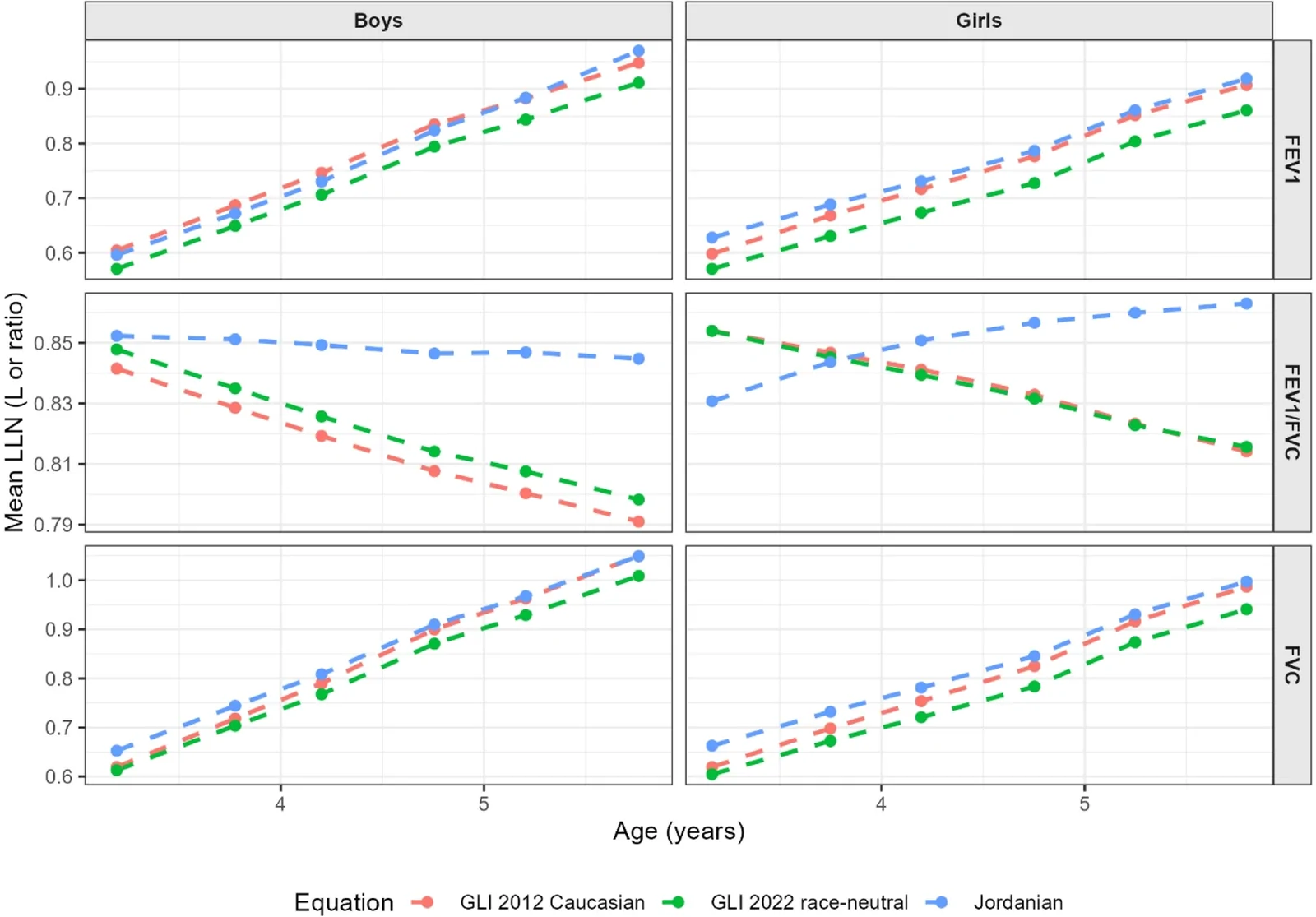
Lower limits of normal (LLNs) by age, sex, and reference system in the derivation cohort.

**Fig. 4:**
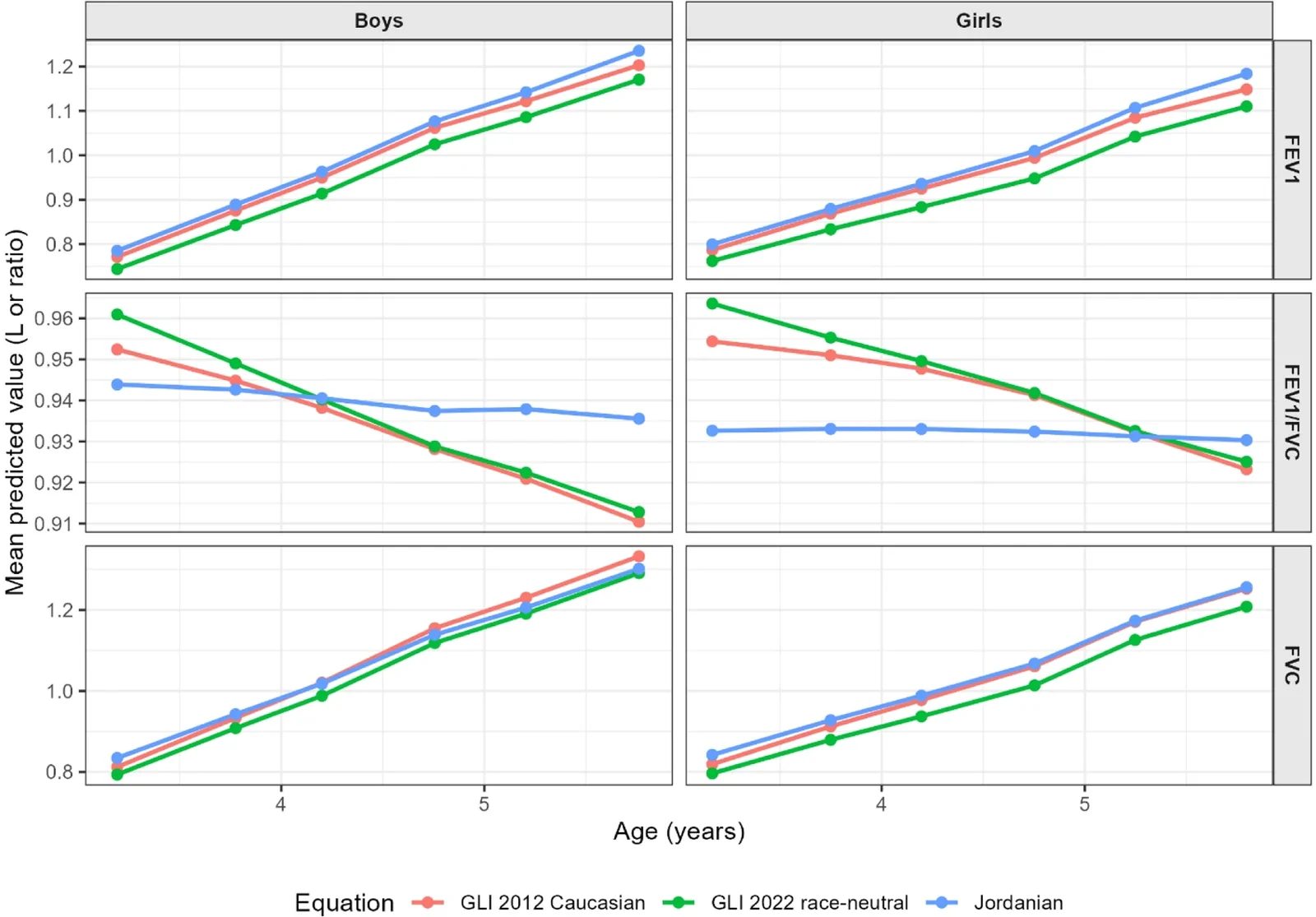
Predicted spirometric values by age, sex, and reference system in the derivation cohort.

Age–specific plots in the derivation cohort showed that the Jordanian equations produced predicted FEV₁ and FVC values that closely followed the age trajectories of both GLI 2012 Caucasian and GLI 2022 race-neutral equations in boys and girls, with a modest upward shift in mean levels and LLNs, particularly at older ages. For FEV₁ and FVC, the Jordanian LLNs remained within a very narrow range around the GLI curves, indicating broadly comparable classification thresholds across the 3–6-year span. In contrast, the age pattern for FEV₁/FVC differed more clearly between systems: whereas GLI 2012 and GLI 2022 predicted a gradual decline in both mean ratio and LLN with increasing age, the Jordanian equations yielded a flatter mean FEV₁/FVC trajectory and LLNs that were slightly higher and more stable over age in boys, and tended to increase rather than decline with age in girls. Despite these differences in shape, the absolute differences in FEV₁/FVC remained small, reinforcing that all three reference systems place healthy children within a narrow, physiologically plausible range for the ratio (Fig. 3, Fig. 4).

## Discussion

This study represents the first sex-specific spirometry reference equations developed and calibrated from a representative sample of Jordanian children using GAMLSS modeling. When evaluating the performance of these equations against the existing GLI reference equations the newly developed equations demonstrated a good fit and accurate prediction of lung function in children aged 3–6 years. Overall, the results suggest strong calibration and reliable performance across key spirometric parameters, particularly for FEV₁ and FVC.

In an attempt to develop spirometry prediction formulas that can be applied to people across the world, the GLI generated the multi-ethnic, age-inclusive GLI 2012 equations [6]. Yet, these formulas have shortcomings that may compromise their generalizability and emphasize the urgent need for population-specific prediction models, designed for different demographic profiles. A significant limitation of the GLI 2012 stems from neglecting the heterogeneity present within the same ethnic group. As demonstrated in the GLI-Caucasian equation, which is based on data from different parts of the world, such as Europe, Australia, America, and North Africa [6]. These highly diverse populations may diminish the usefulness of the GLI 2012 equations, since individuals sharing the same ethnic origin but live in different regions may demonstrate variability in lung function. This difference was evident when the lung function of Indians residing in India was compared to those residing in the United States, as the spirometry measurements were higher in the latter despite accounting for age and height [23]. Furthermore, several ethnic and age groups were lacking adequate representation, such as Middle Eastern Arabs and preschool-aged children [6]. Subsequently, The GLI 2022 equation was developed to overcome the limitations associated with the GLI 2012, by excluding the race element from calculations. Minimizing the bias attributed to over-representation of White Europeans, who constituted >75% of the gathered data. Nevertheless, since the race-neutral equation relied on the same dataset, it fails to resolve generalizability issues related to the under-representation of many ethnicities [9].

Consequently, both equations may not accurately reflect the Jordanian population, highlighting the need for population-specific reference formulas, particularly preschool-aged children. To bridge this gap, the current study derived sex-specific spirometry reference equations from 911 healthy Jordanian children between the ages of 3 and 6 years old, using a GAMLSS modelling approach. The conventional regression analysis is based on multiple assumptions, including fixed variance, linearity between variables, and normality of residuals, which are not suitable for lung function parameters, particularly for the usually skewed distributions of FEV_1_/FVC [24]. In contrast, GAMLSS framework offers the flexibility to adapt assumptions that are more realistic [25]. The lambda, mu, sigma (LMS) method, which is often used to generate pulmonary function reference values, offers multiple strategies for LLN estimation over the spectrum of height and age instead of relying on inaccurate constant estimates, which is crucial for age-dependent measures such as FEV_1_/FVC, since using a set threshold across different age groups is inappropriate [26]. Moreover, a generalized version of the LMS method within GAMLSS enables modeling of all distributional parameters: location (μ), scale (σ), skewness (ν), and kurtosis (τ), as a function of age and/or height. As a result, GAMLSS framework is ideal for modeling spirometry measures, which vary with age and height, and exhibit non-linear and skewed distribution patterns [24]. In the present study, FEV_1_ and FVC values in preschoolers of both sexes were best modeled by BCPE, underscoring the importance of accounting for age-related skewness and kurtosis. These results indicate that the BCCG model used to formulate the GLI 2012 [6], might not offer adequate flexibility to capture pulmonary flow-volumes among 3–6 years old Jordanian children. Conversely, less complicated models provided the best fit for FEV_1_/FVC ratio, namely, simple BCCG and normal distributions for boys and girls, respectively. The diagnostic and worm plots affirm that the selected GAMLSS models and age-height predictors achieve a good and robust fit for preschool spirometry data across the whole age range.

Calibration analyses of the developed Jordanian prediction equations were performed on the separate Al-Qerem and Jarab validation cohort consisting of 765 children, who were not part of the model-development phase. Regarding FEV1 and FVC for boys and girls, the observed and predicted values exhibited a close match, good calibration as evidenced by intercepts approaching zero and slopes near one, and minimal systematic error. However, the FEV1/FVC ratio regression demonstrated less statistical certainty with wider confidence intervals. Importantly, Bland-Altman analysis did not indicate meaningful systematic miscalibration: the predicted-raw bias for FEV1/FVC was −0.009 in boys and −0.002 in girls, with 95% limits of agreement of −0.100 to 0.082 and −0.091 to 0.088, respectively. This was also consistent with the close Jordanian FEV1/FVC z-score distribution. These findings indicate that the lower regression precision for FEV1/FVC reflected scatter around a narrow ratio range rather than a directional bias. This is biologically and technically plausible because FEV1/FVC is bounded and varies over a narrow range in healthy preschoolers; small variation in either FEV1 or FVC can therefore produce proportionally larger shifts in the ratio. This interpretation is consistent with previous preschool and pediatric spirometry datasets, which show that FEV1 and FVC increase clearly with growth whereas FEV1/FVC remains high and restricted in early childhood and therefore requires age- and height-specific interpretation [27., 28., 29., 30., 31.].

In the external validation assessment, the Jordanian equations generated comparable FEV₁ and FVC distributions to the GLI 2012-Caucasian equations, both of which had a mean z-scores close to zero and percent-predicted values around 100%. However, the GLI 2022 resulted in higher mean z-scores and percent-predicted values, in addition to lower percentage of children falling below the LLN, especially in girls. As for the FEV₁/FVC ratio, the three sets of equations displayed similar patterns. Suggesting that the developed equations and the GLI 2012- Caucasian exhibit similar prediction patterns, while the race-neutral GLI 2022 tends to overestimate lung function in this population. This aligns with previous studies demonstrating that applying the multi-ethnic GLI 2012 for preschoolers is reasonable, despite not being a perfect fit [10,32], however, switching to the race-neutral GLI 2022 equation may lead to underdiagnosis of respiratory conditions in some children, and increase false-negative interpretation particularly when measures are near the LLNs [33,34].

In the derivation cohort, the Jordanian predicted FEV₁, FVC, and LLN for both sexes displayed near parallel age-specific trends to the GLI 2012-Caucasian and GLI 2022 equations, with marginal increase at the upper end of the age range. However, the FEV₁/FVC ratio exhibited a more pronounced divergence. While the Jordanian equations produced flatter patterns in boys and slightly upward patterns in girls, the GLI equations demonstrated a decline in the mean ratio and LLNs with age. The curves derived from the Jordanian equations follow the natural trajectory of lung development in healthy preschoolers more closely. During this stage, FEV₁ and FVC rapidly rise with height, whereas FEV₁/FVC exhibit a slow decline between the ages of 5 and 11 years, from around 0.96 to 0.87–0.89 [28]. Regardless of these minor differences in shapes and ratios, the three sets of reference equations displayed comparable tendency to appropriately classify healthy 3–6 years old children within a narrow range. Because this was a single-country cross-sectional sample, the observed ratio pattern should be interpreted as population-specific calibration behavior rather than evidence of a fixed ethnic trait.

Overall, the Jordanian spirometry equations provide an accurate representation of the local preschool children population, which is critical for correct spirometry interpretation.

## Strengths and limitations

The current study has many valuable strengths. Notably, generating the new reference equations from a large sample of preschool children, the first to address this age group in the Arab world, using a flexible modeling approach. Performing spirometry testing with strict adherence to ATS/ERS guidelines. Moreover, conducting calibration analysis and direct comparison with the GLI 2012 and GLI 2022 equations, using an independent dataset. All of which substantiate the robustness and clinical relevance of the newly developed equations.

Nevertheless, few limitations are worth noting. The study sample was collected from one country, which may restrict the applicability of the developed equations to other populations in the Middle East. The equations should therefore be applied cautiously outside Jordan, as intra-regional variation may exist between urban and rural Jordanian communities and between nearby Arab populations such as those in Saudi Arabia, the United Arab Emirates, and Egypt. Furthermore, factors that may influence lung growth and development early in life, including prenatal or postnatal exposure to pollution, passive smoking, and nutritional status, were not measured and therefore could not be used as exclusion criteria or adjustment variables [35]. The 2025 model-development cohort was collected over several months, between April and September 2025, whereas the Al-Qerem and Jarab validation cohort was collected over a similarly extended period, between April and December 2019. The extended recruitment periods in both cohorts reduce the likelihood that findings were driven by a narrow seasonal window, and the temporal separation was intentional to evaluate model performance across different recruitment periods; however, residual seasonal effects cannot be fully excluded. COVID-19 history was not available, although children with recent respiratory infection were excluded.

## Future work

Preschool-aged children may sometimes fail to perform spirometry adequately due to the complexity of the technique [36]. Additionally, spirometry is not a suitable lung function test for preschoolers who were born prematurely [37]. Fortunately, there are other options that can be explored, for instance, the forced oscillation technique (FOT), which is a non-invasive, sensitive, and relatively inexpensive lung function test. Its clinical effectiveness was supported by many studies with a reported success rate between 74% and 98% among preschool children, as it requires minimal subject cooperation [38]. Due to the limited validation studies in the Middle East, future efforts should be directed toward evaluating the applicability of these tests on preschool children in Jordan and toward implementing the new spirometry equations in commercial spirometry software or an online calculator for clinical use.

## Conclusion

This study generated the first spirometry reference equations tailored specifically for Jordanian preschool-aged children. The new equations exhibited strong performance, with good calibration, mean z-scores near zero, and closely mirrored predicted and measured spirometry values in an independent cohort. These findings provide substantial evidence to promote the use of the Jordanian equations in clinical settings to improve the accuracy of lung function assessment, which presents a major diagnostic challenge during preschool years.

## Conflicts of interest

The author declares no conflicts of interest.

## Appendix Supplementary materials

Image, application 1

Image, application 2

Image, application 3

## Footnotes

Supplementary material associated with this article can be found in the online version at doi:10.1016/j.jped.2026.101606.

## References

[1] GBD 2021 Asthma and Allergic Diseases Collaborators (2025). Global, regional, and national burden of asthma and atopic dermatitis, 1990-2021, and projections to 2050: a systematic analysis of the Global Burden of Disease Study 2021. Lancet Respir Med.

[2] Network GB of DC. Global Burden of Disease Study 2023 (GBD 2023). Seattle, United States: Institute for Health Metrics and Evaluation (IHME); 2025.

[3] Larsen GL. (2000). Differences between adult and childhood asthma. J Allergy Clin Immunol.

[4] Chung HL. (2022). Diagnosis and management of asthma in infants and preschoolers. Clin Exp Pediatr.

[5] Saglani S., Menzie-Gow AN. (2019). Approaches to asthma diagnosis in children and adults. Front Pediatr.

[6] Quanjer P.H., Stanojevic S., Cole T.J., Baur X., Hall G.L., Culver B.H. (2012). Multi-ethnic reference values for spirometry for the 3-95-yr age range: the global lung function 2012 equations. Eur Respir J.

[7] Moore VC. (2012). Spirometry: step by step. Breathe.

[8] Choi H.S., Park Y.B., Yoon H.K., Lim S.Y., Kim T.H., Park J.H. (2019). Validation of previous spirometric reference equations and new equations. J Korean Med Sci.

[9] Bowerman C., Bhakta N.R., Brazzale D., Cooper B.R., Cooper J., Gochicoa-Rangel L. (2023). A race-neutral approach to the interpretation of lung function measurements. Am J Respir Crit Care Med.

[10] Al-Qerem W.A., Jarab AS. (2021). Applicability of GLI 2012 spirometry equation among preschool aged Jordanian. Respir Med.

[11] Al-Qerem W.A. (2020). How applicable are GLI 2012 equations to a sample of Middle Eastern school-age children?. Pediatr Pulmonol.

[12] Al-Qerem W., Alassi A., Jarab A.S., Ling J. (2021). The applicability of the global lung initiative equations and other regional equations on a sample of healthy Middle Eastern adolescents. Clin Respir J.

[13] Al-Qerem W.A., Hammad A.M., AlQirem R.A., Ling J. (2019). Do the global lung function initiative reference equations reflect a sample of adult Middle Eastern population?. Clin Respir J.

[14] Al-Qerem W. (2025). Evaluation of spirometry reference equations among healthy Jordanian adults: a comparative analysis of Jordanian and the Global Lung Initiative equations. Expert Rev Respir Med.

[15] Al-Qerem W., Hammad A.M., Gassar E.S., Al-Qirim R.A., Ling J. (2019). Spirometry reference equations for an adult Middle Eastern population. Expert Rev Respir Med.

[16] Al-Qerem W. (2021). Spirometry reference equations for children from a Middle Eastern population. Int J Clin Pract.

[17] Greenough A., Giffin F.J. (1996). Yüksel B. Respiratory morbidity in preschool children born prematurely. Relationship to adverse neonatal events. Acta Paediatr.

[18] Riley R.D., Ensor J., Snell K.I., Harrell F.E., Martin G.P., Reitsma J.B. (2020). Calculating the sample size required for developing a clinical prediction model. BMJ.

[19] Riley R.D., Snell K.I., Ensor J., Burke D.L., Harrell F.E., Moons K.G. (2019). Minimum sample size for developing a multivariable prediction model: part I - continuous outcomes. Stat Med.

[20] Quanjer P.H., Stocks J., Cole T.J., Hall G.L. (2011). Stanojevic S; Global Lungs Initiative. Influence of secular trends and sample size on reference equations for lung function tests. Eur Respir J.

[21] Garrow J.S., Webster J. (1985). Quetelet’s index (W/H2) as a measure of fatness. Int J Obes.

[22] Beydon N., Davis S.D., Lombardi E., Allen J.L., Arets H.G., Aurora P. (2007). An official American Thoracic Society/European Respiratory Society statement: pulmonary function testing in preschool children. Am J Respir Crit Care Med.

[23] Fulambarker A., Copur A.S., Cohen M.E., Patel M., Gill S., Schultz S.T. (2010). Comparison of pulmonary function in immigrant vs US-born Asian Indians. Chest.

[24] Cole T.J., Stanojevic S., Stocks J., Coates A.L., Hankinson J.L., Wade AM. (2009). Age- and size-related reference ranges: a case study of spirometry through childhood and adulthood. Stat Med.

[25] Stasinopoulos D.M., Rigby RA. (2007). Generalized additive models for location scale and shape (GAMLSS) in R. J Stat Softw.

[26] Culver BH. (2012). How should the lower limit of the normal range be defined?. Respir Care.

[27] Stanojevic S., Wade A., Cole T.J., Lum S., Custovic A., Silverman M. (2009). Spirometry centile charts for young Caucasian children: the Asthma UK Collaborative Initiative. Am J Respir Crit Care Med.

[28] Quanjer P.H., Stanojevic S., Stocks J., Hall G.L., Prasad K.V., Cole T.J. (2010). Changes in the FEV₁/FVC ratio during childhood and adolescence: an intercontinental study. Eur Respir J.

[29] Jeng M-J, Chang H-L, Tsai M-C, Tsao P-C, Yang C-F, Lee Y-S (2009). Spirometric pulmonary function parameters of healthy Chinese children aged 3-6 years in Taiwan. Pediatr Pulmonol.

[30] Koopman M., Zanen P., Kruitwagen C.L., van der Ent C.K., Arets HG. (2011). Reference values for paediatric pulmonary function testing: the Utrecht dataset. Respir Med.

[31] Chang S.M., Tsai H.J., Tzeng J.Y., Yeh K.W., Chen L.C., Lai S.H. (2019). Reference equations for spirometry in healthy Asian children aged 5 to 18 years in Taiwan. World Allergy Organ J.

[32] Martín de Vicente C., de Mir Messa I., Rovira Amigo S., Torrent Vernetta A., Gartner S., Iglesias Serrano I. (2018). Validation of global lung function initiative and all ages reference equations for forced spirometry in healthy Spanish preschoolers. Arch Bronconeumol (Engl Ed).

[33] McCoy J., Wilson D., Perrem L. (2023). The impact of GLI Global equations on spirometry interpretation in a diverse pediatric population. Eur Respir J.

[34] Chan K.C., Zhu H., Yu M., Yuen H.M., Dai S., Chin H.Y. (2023). Applicability of the Global Lung Function Initiative prediction equations in Hong Kong Chinese children. Pediatr Pulmonol.

[35] Stocks J., Sonnappa S. (2013). Early life influences on the development of chronic obstructive pulmonary disease. Ther Adv Respir Dis.

[36] Kampschmidt J.C., Brooks E.G., Cherry D.C., Guajardo J.R., Wood PR. (2016). Feasibility of spirometry testing in preschool children. Pediatr Pulmonol.

[37] Kairamkonda V.R., Richardson J., Subhedar N., Bridge P.D., Shaw NJ. (2008). Lung function measurement in prematurely born preschool children with and without chronic lung disease. J Perinatol.

[38] Chaya S., Zar H.J., Gray DM. (2022). Lung function in preschool children in low and middle income countries: an under-represented potential tool to strengthen child health. Front Pediatr.

