# Supplementary material for: Derivation and validation of spirometry reference equations for Jordanian preschool children using GAMLSS modelling
Source: J Pediatr (Rio J). 2026 Sep 6;102(6):101606. doi: 10.1016/j.jped.2026.101606 (PMC13579300; doi:10.1016/j.jped.2026.101606)
Supplement: Supplementary file 1 [file mmc1.docx]

**Figure A1.** Diagnostic plots for FEV1 in girls

**Figure A2.** Diagnostic plots for FVC in boys

**Figure A3.** Diagnostic plots for FVC in girls

**Figure A4.** Diagnostic plots for FEV1/FVC in boys

**Figure A5.** Diagnostic plots for FEV1/FVC in girls

**Figure A6.** Worm plots for FEV₁ in girls fitted with the BCPE model

**Figure A7.** Worm plots for FVC in boys fitted with the BCPE model

**Figure A8.** Worm plots for FVC in girls fitted with the BCPE model\

**Figure A9.** Worm plots for FEV₁/FVC in boys fitted with the BCPE model

**Figure A10.** Worm plots for FEV₁/FVC in girls fitted with the BCPE model
