# Supplementary material for: Derivation and validation of spirometry reference equations for Jordanian preschool children using GAMLSS modelling
Source: J Pediatr (Rio J). 2026 Sep 6;102(6):101606. doi: 10.1016/j.jped.2026.101606 (PMC13579300; doi:10.1016/j.jped.2026.101606)
Supplement: Supplementary file 2 [file mmc2.docx]

**Table A1. Sex-specific LMS parameters, predicted values, and lower limits of normal (LLN) for FEV₁ across the Phase 1 age and height range.**

| **Outcome** | **Sex** | **age** | **Height (cm)** | **Family** | **L** | **M** | **S** | **K** | **Pred** | **LLN** |
| --- | --- | --- | --- | --- | --- | --- | --- | --- | --- | --- |
| FEV1 | Boys | 3.00 | 79 | BCPE | 2.50011 | 0.5126 | 0.12617 | 10.41215 | 0.5126 | 0.3908 |
| FEV1 | Boys | 3.00 | 80 | BCPE | 2.50011 | 0.5269 | 0.12617 | 10.41215 | 0.5269 | 0.4017 |
| FEV1 | Boys | 3.00 | 81 | BCPE | 2.50011 | 0.5413 | 0.12617 | 10.41215 | 0.5413 | 0.4127 |
| FEV1 | Boys | 3.00 | 82 | BCPE | 2.50011 | 0.5560 | 0.12617 | 10.41215 | 0.5560 | 0.4239 |
| FEV1 | Boys | 3.00 | 83 | BCPE | 2.50011 | 0.5708 | 0.12617 | 10.41215 | 0.5708 | 0.4352 |
| FEV1 | Boys | 3.00 | 84 | BCPE | 2.50011 | 0.5859 | 0.12617 | 10.41215 | 0.5859 | 0.4467 |
| FEV1 | Boys | 3.00 | 85 | BCPE | 2.50011 | 0.6012 | 0.12617 | 10.41215 | 0.6012 | 0.4584 |
| FEV1 | Boys | 3.00 | 86 | BCPE | 2.50011 | 0.6167 | 0.12617 | 10.41215 | 0.6167 | 0.4702 |
| FEV1 | Boys | 3.00 | 87 | BCPE | 2.50011 | 0.6324 | 0.12617 | 10.41215 | 0.6324 | 0.4822 |
| FEV1 | Boys | 3.00 | 88 | BCPE | 2.50011 | 0.6484 | 0.12617 | 10.41215 | 0.6484 | 0.4943 |
| FEV1 | Boys | 3.00 | 89 | BCPE | 2.50011 | 0.6645 | 0.12617 | 10.41215 | 0.6645 | 0.5066 |
| FEV1 | Boys | 3.00 | 90 | BCPE | 2.50011 | 0.6809 | 0.12617 | 10.41215 | 0.6809 | 0.5191 |
| FEV1 | Boys | 3.00 | 91 | BCPE | 2.50011 | 0.6974 | 0.12617 | 10.41215 | 0.6974 | 0.5317 |
| FEV1 | Boys | 3.00 | 92 | BCPE | 2.50011 | 0.7142 | 0.12617 | 10.41215 | 0.7142 | 0.5445 |
| FEV1 | Boys | 3.00 | 93 | BCPE | 2.50011 | 0.7312 | 0.12617 | 10.41215 | 0.7312 | 0.5575 |
| FEV1 | Boys | 3.00 | 94 | BCPE | 2.50011 | 0.7485 | 0.12617 | 10.41215 | 0.7485 | 0.5706 |
| FEV1 | Boys | 3.00 | 95 | BCPE | 2.50011 | 0.7659 | 0.12617 | 10.41215 | 0.7659 | 0.5839 |
| FEV1 | Boys | 3.00 | 96 | BCPE | 2.50011 | 0.7836 | 0.12617 | 10.41215 | 0.7836 | 0.5974 |
| FEV1 | Boys | 3.00 | 97 | BCPE | 2.50011 | 0.8015 | 0.12617 | 10.41215 | 0.8015 | 0.6110 |
| FEV1 | Boys | 3.00 | 98 | BCPE | 2.50011 | 0.8195 | 0.12617 | 10.41215 | 0.8195 | 0.6248 |
| FEV1 | Boys | 3.00 | 99 | BCPE | 2.50011 | 0.8379 | 0.12617 | 10.41215 | 0.8379 | 0.6388 |
| FEV1 | Boys | 3.00 | 100 | BCPE | 2.50011 | 0.8564 | 0.12617 | 10.41215 | 0.8564 | 0.6529 |
| FEV1 | Boys | 3.00 | 101 | BCPE | 2.50011 | 0.8752 | 0.12617 | 10.41215 | 0.8752 | 0.6672 |
| FEV1 | Boys | 3.00 | 102 | BCPE | 2.50011 | 0.8941 | 0.12617 | 10.41215 | 0.8941 | 0.6817 |
| FEV1 | Boys | 3.00 | 103 | BCPE | 2.50011 | 0.9133 | 0.12617 | 10.41215 | 0.9133 | 0.6963 |
| FEV1 | Boys | 3.00 | 104 | BCPE | 2.50011 | 0.9327 | 0.12617 | 10.41215 | 0.9327 | 0.7111 |
| FEV1 | Boys | 3.00 | 105 | BCPE | 2.50011 | 0.9524 | 0.12617 | 10.41215 | 0.9524 | 0.7261 |
| FEV1 | Boys | 3.00 | 106 | BCPE | 2.50011 | 0.9722 | 0.12617 | 10.41215 | 0.9722 | 0.7412 |
| FEV1 | Boys | 3.00 | 107 | BCPE | 2.50011 | 0.9923 | 0.12617 | 10.41215 | 0.9923 | 0.7565 |
| FEV1 | Boys | 3.00 | 108 | BCPE | 2.50011 | 1.0126 | 0.12617 | 10.41215 | 1.0126 | 0.7720 |
| FEV1 | Boys | 3.00 | 109 | BCPE | 2.50011 | 1.0331 | 0.12617 | 10.41215 | 1.0331 | 0.7876 |
| FEV1 | Boys | 3.00 | 110 | BCPE | 2.50011 | 1.0539 | 0.12617 | 10.41215 | 1.0539 | 0.8035 |
| FEV1 | Boys | 3.00 | 111 | BCPE | 2.50011 | 1.0748 | 0.12617 | 10.41215 | 1.0748 | 0.8194 |
| FEV1 | Boys | 3.00 | 112 | BCPE | 2.50011 | 1.0960 | 0.12617 | 10.41215 | 1.0960 | 0.8356 |
| FEV1 | Boys | 3.00 | 113 | BCPE | 2.50011 | 1.1174 | 0.12617 | 10.41215 | 1.1174 | 0.8519 |
| FEV1 | Boys | 3.00 | 114 | BCPE | 2.50011 | 1.1391 | 0.12617 | 10.41215 | 1.1391 | 0.8684 |
| FEV1 | Boys | 3.00 | 115 | BCPE | 2.50011 | 1.1610 | 0.12617 | 10.41215 | 1.1610 | 0.8851 |
| FEV1 | Boys | 3.00 | 116 | BCPE | 2.50011 | 1.1830 | 0.12617 | 10.41215 | 1.1830 | 0.9019 |
| FEV1 | Boys | 3.00 | 117 | BCPE | 2.50011 | 1.2054 | 0.12617 | 10.41215 | 1.2054 | 0.9189 |
| FEV1 | Boys | 3.00 | 118 | BCPE | 2.50011 | 1.2279 | 0.12617 | 10.41215 | 1.2279 | 0.9361 |
| FEV1 | Boys | 3.00 | 119 | BCPE | 2.50011 | 1.2507 | 0.12617 | 10.41215 | 1.2507 | 0.9535 |
| FEV1 | Boys | 3.00 | 120 | BCPE | 2.50011 | 1.2737 | 0.12617 | 10.41215 | 1.2737 | 0.9710 |
| FEV1 | Boys | 3.00 | 121 | BCPE | 2.50011 | 1.2969 | 0.12617 | 10.41215 | 1.2969 | 0.9887 |
| FEV1 | Boys | 3.00 | 122 | BCPE | 2.50011 | 1.3203 | 0.12617 | 10.41215 | 1.3203 | 1.0066 |
| FEV1 | Boys | 3.00 | 123 | BCPE | 2.50011 | 1.3440 | 0.12617 | 10.41215 | 1.3440 | 1.0246 |
| FEV1 | Boys | 3.00 | 124 | BCPE | 2.50011 | 1.3679 | 0.12617 | 10.41215 | 1.3679 | 1.0429 |
| FEV1 | Boys | 3.00 | 125 | BCPE | 2.50011 | 1.3920 | 0.12617 | 10.41215 | 1.3920 | 1.0613 |
| FEV1 | Boys | 3.00 | 126 | BCPE | 2.50011 | 1.4164 | 0.12617 | 10.41215 | 1.4164 | 1.0798 |
| FEV1 | Boys | 3.00 | 127 | BCPE | 2.50011 | 1.4410 | 0.12617 | 10.41215 | 1.4410 | 1.0986 |
| FEV1 | Boys | 3.00 | 128 | BCPE | 2.50011 | 1.4658 | 0.12617 | 10.41215 | 1.4658 | 1.1175 |
| FEV1 | Boys | 3.00 | 129 | BCPE | 2.50011 | 1.4908 | 0.12617 | 10.41215 | 1.4908 | 1.1366 |
| FEV1 | Boys | 3.00 | 130 | BCPE | 2.50011 | 1.5161 | 0.12617 | 10.41215 | 1.5161 | 1.1559 |
| FEV1 | Boys | 3.00 | 131 | BCPE | 2.50011 | 1.5416 | 0.12617 | 10.41215 | 1.5416 | 1.1753 |
| FEV1 | Boys | 3.00 | 132 | BCPE | 2.50011 | 1.5673 | 0.12617 | 10.41215 | 1.5673 | 1.1949 |
| FEV1 | Boys | 3.25 | 79 | BCPE | 2.51046 | 0.5184 | 0.12761 | 10.16328 | 0.5184 | 0.3932 |
| FEV1 | Boys | 3.25 | 80 | BCPE | 2.51046 | 0.5328 | 0.12761 | 10.16328 | 0.5328 | 0.4041 |
| FEV1 | Boys | 3.25 | 81 | BCPE | 2.51046 | 0.5474 | 0.12761 | 10.16328 | 0.5474 | 0.4152 |
| FEV1 | Boys | 3.25 | 82 | BCPE | 2.51046 | 0.5622 | 0.12761 | 10.16328 | 0.5622 | 0.4264 |
| FEV1 | Boys | 3.25 | 83 | BCPE | 2.51046 | 0.5772 | 0.12761 | 10.16328 | 0.5772 | 0.4378 |
| FEV1 | Boys | 3.25 | 84 | BCPE | 2.51046 | 0.5925 | 0.12761 | 10.16328 | 0.5925 | 0.4494 |
| FEV1 | Boys | 3.25 | 85 | BCPE | 2.51046 | 0.6079 | 0.12761 | 10.16328 | 0.6079 | 0.4611 |
| FEV1 | Boys | 3.25 | 86 | BCPE | 2.51046 | 0.6236 | 0.12761 | 10.16328 | 0.6236 | 0.4730 |
| FEV1 | Boys | 3.25 | 87 | BCPE | 2.51046 | 0.6395 | 0.12761 | 10.16328 | 0.6395 | 0.4851 |
| FEV1 | Boys | 3.25 | 88 | BCPE | 2.51046 | 0.6556 | 0.12761 | 10.16328 | 0.6556 | 0.4973 |
| FEV1 | Boys | 3.25 | 89 | BCPE | 2.51046 | 0.6719 | 0.12761 | 10.16328 | 0.6719 | 0.5097 |
| FEV1 | Boys | 3.25 | 90 | BCPE | 2.51046 | 0.6885 | 0.12761 | 10.16328 | 0.6885 | 0.5222 |
| FEV1 | Boys | 3.25 | 91 | BCPE | 2.51046 | 0.7053 | 0.12761 | 10.16328 | 0.7053 | 0.5350 |
| FEV1 | Boys | 3.25 | 92 | BCPE | 2.51046 | 0.7222 | 0.12761 | 10.16328 | 0.7222 | 0.5478 |
| FEV1 | Boys | 3.25 | 93 | BCPE | 2.51046 | 0.7394 | 0.12761 | 10.16328 | 0.7394 | 0.5609 |
| FEV1 | Boys | 3.25 | 94 | BCPE | 2.51046 | 0.7569 | 0.12761 | 10.16328 | 0.7569 | 0.5741 |
| FEV1 | Boys | 3.25 | 95 | BCPE | 2.51046 | 0.7745 | 0.12761 | 10.16328 | 0.7745 | 0.5875 |
| FEV1 | Boys | 3.25 | 96 | BCPE | 2.51046 | 0.7924 | 0.12761 | 10.16328 | 0.7924 | 0.6010 |
| FEV1 | Boys | 3.25 | 97 | BCPE | 2.51046 | 0.8104 | 0.12761 | 10.16328 | 0.8104 | 0.6147 |
| FEV1 | Boys | 3.25 | 98 | BCPE | 2.51046 | 0.8287 | 0.12761 | 10.16328 | 0.8287 | 0.6286 |
| FEV1 | Boys | 3.25 | 99 | BCPE | 2.51046 | 0.8472 | 0.12761 | 10.16328 | 0.8472 | 0.6427 |
| FEV1 | Boys | 3.25 | 100 | BCPE | 2.51046 | 0.8660 | 0.12761 | 10.16328 | 0.8660 | 0.6569 |
| FEV1 | Boys | 3.25 | 101 | BCPE | 2.51046 | 0.8850 | 0.12761 | 10.16328 | 0.8850 | 0.6713 |
| FEV1 | Boys | 3.25 | 102 | BCPE | 2.51046 | 0.9041 | 0.12761 | 10.16328 | 0.9041 | 0.6858 |
| FEV1 | Boys | 3.25 | 103 | BCPE | 2.51046 | 0.9235 | 0.12761 | 10.16328 | 0.9235 | 0.7005 |
| FEV1 | Boys | 3.25 | 104 | BCPE | 2.51046 | 0.9432 | 0.12761 | 10.16328 | 0.9432 | 0.7154 |
| FEV1 | Boys | 3.25 | 105 | BCPE | 2.51046 | 0.9630 | 0.12761 | 10.16328 | 0.9630 | 0.7305 |
| FEV1 | Boys | 3.25 | 106 | BCPE | 2.51046 | 0.9831 | 0.12761 | 10.16328 | 0.9831 | 0.7457 |
| FEV1 | Boys | 3.25 | 107 | BCPE | 2.51046 | 1.0034 | 0.12761 | 10.16328 | 1.0034 | 0.7611 |
| FEV1 | Boys | 3.25 | 108 | BCPE | 2.51046 | 1.0239 | 0.12761 | 10.16328 | 1.0239 | 0.7767 |
| FEV1 | Boys | 3.25 | 109 | BCPE | 2.51046 | 1.0447 | 0.12761 | 10.16328 | 1.0447 | 0.7924 |
| FEV1 | Boys | 3.25 | 110 | BCPE | 2.51046 | 1.0657 | 0.12761 | 10.16328 | 1.0657 | 0.8083 |
| FEV1 | Boys | 3.25 | 111 | BCPE | 2.51046 | 1.0869 | 0.12761 | 10.16328 | 1.0869 | 0.8244 |
| FEV1 | Boys | 3.25 | 112 | BCPE | 2.51046 | 1.1083 | 0.12761 | 10.16328 | 1.1083 | 0.8407 |
| FEV1 | Boys | 3.25 | 113 | BCPE | 2.51046 | 1.1300 | 0.12761 | 10.16328 | 1.1300 | 0.8571 |
| FEV1 | Boys | 3.25 | 114 | BCPE | 2.51046 | 1.1518 | 0.12761 | 10.16328 | 1.1518 | 0.8737 |
| FEV1 | Boys | 3.25 | 115 | BCPE | 2.51046 | 1.1740 | 0.12761 | 10.16328 | 1.1740 | 0.8905 |
| FEV1 | Boys | 3.25 | 116 | BCPE | 2.51046 | 1.1963 | 0.12761 | 10.16328 | 1.1963 | 0.9074 |
| FEV1 | Boys | 3.25 | 117 | BCPE | 2.51046 | 1.2189 | 0.12761 | 10.16328 | 1.2189 | 0.9245 |
| FEV1 | Boys | 3.25 | 118 | BCPE | 2.51046 | 1.2417 | 0.12761 | 10.16328 | 1.2417 | 0.9418 |
| FEV1 | Boys | 3.25 | 119 | BCPE | 2.51046 | 1.2647 | 0.12761 | 10.16328 | 1.2647 | 0.9593 |
| FEV1 | Boys | 3.25 | 120 | BCPE | 2.51046 | 1.2879 | 0.12761 | 10.16328 | 1.2879 | 0.9769 |
| FEV1 | Boys | 3.25 | 121 | BCPE | 2.51046 | 1.3114 | 0.12761 | 10.16328 | 1.3114 | 0.9947 |
| FEV1 | Boys | 3.25 | 122 | BCPE | 2.51046 | 1.3351 | 0.12761 | 10.16328 | 1.3351 | 1.0127 |
| FEV1 | Boys | 3.25 | 123 | BCPE | 2.51046 | 1.3591 | 0.12761 | 10.16328 | 1.3591 | 1.0309 |
| FEV1 | Boys | 3.25 | 124 | BCPE | 2.51046 | 1.3832 | 0.12761 | 10.16328 | 1.3832 | 1.0492 |
| FEV1 | Boys | 3.25 | 125 | BCPE | 2.51046 | 1.4076 | 0.12761 | 10.16328 | 1.4076 | 1.0677 |
| FEV1 | Boys | 3.25 | 126 | BCPE | 2.51046 | 1.4322 | 0.12761 | 10.16328 | 1.4322 | 1.0864 |
| FEV1 | Boys | 3.25 | 127 | BCPE | 2.51046 | 1.4571 | 0.12761 | 10.16328 | 1.4571 | 1.1053 |
| FEV1 | Boys | 3.25 | 128 | BCPE | 2.51046 | 1.4822 | 0.12761 | 10.16328 | 1.4822 | 1.1243 |
| FEV1 | Boys | 3.25 | 129 | BCPE | 2.51046 | 1.5075 | 0.12761 | 10.16328 | 1.5075 | 1.1435 |
| FEV1 | Boys | 3.25 | 130 | BCPE | 2.51046 | 1.5331 | 0.12761 | 10.16328 | 1.5331 | 1.1629 |
| FEV1 | Boys | 3.25 | 131 | BCPE | 2.51046 | 1.5589 | 0.12761 | 10.16328 | 1.5589 | 1.1825 |
| FEV1 | Boys | 3.25 | 132 | BCPE | 2.51046 | 1.5849 | 0.12761 | 10.16328 | 1.5849 | 1.2022 |
| FEV1 | Boys | 3.50 | 79 | BCPE | 2.52005 | 0.5238 | 0.12836 | 9.93817 | 0.5238 | 0.3961 |
| FEV1 | Boys | 3.50 | 80 | BCPE | 2.52005 | 0.5383 | 0.12836 | 9.93817 | 0.5383 | 0.4071 |
| FEV1 | Boys | 3.50 | 81 | BCPE | 2.52005 | 0.5531 | 0.12836 | 9.93817 | 0.5531 | 0.4183 |
| FEV1 | Boys | 3.50 | 82 | BCPE | 2.52005 | 0.5680 | 0.12836 | 9.93817 | 0.5680 | 0.4296 |
| FEV1 | Boys | 3.50 | 83 | BCPE | 2.52005 | 0.5832 | 0.12836 | 9.93817 | 0.5832 | 0.4411 |
| FEV1 | Boys | 3.50 | 84 | BCPE | 2.52005 | 0.5986 | 0.12836 | 9.93817 | 0.5986 | 0.4527 |
| FEV1 | Boys | 3.50 | 85 | BCPE | 2.52005 | 0.6142 | 0.12836 | 9.93817 | 0.6142 | 0.4645 |
| FEV1 | Boys | 3.50 | 86 | BCPE | 2.52005 | 0.6301 | 0.12836 | 9.93817 | 0.6301 | 0.4765 |
| FEV1 | Boys | 3.50 | 87 | BCPE | 2.52005 | 0.6461 | 0.12836 | 9.93817 | 0.6461 | 0.4887 |
| FEV1 | Boys | 3.50 | 88 | BCPE | 2.52005 | 0.6624 | 0.12836 | 9.93817 | 0.6624 | 0.5010 |
| FEV1 | Boys | 3.50 | 89 | BCPE | 2.52005 | 0.6789 | 0.12836 | 9.93817 | 0.6789 | 0.5135 |
| FEV1 | Boys | 3.50 | 90 | BCPE | 2.52005 | 0.6956 | 0.12836 | 9.93817 | 0.6956 | 0.5261 |
| FEV1 | Boys | 3.50 | 91 | BCPE | 2.52005 | 0.7126 | 0.12836 | 9.93817 | 0.7126 | 0.5389 |
| FEV1 | Boys | 3.50 | 92 | BCPE | 2.52005 | 0.7297 | 0.12836 | 9.93817 | 0.7297 | 0.5519 |
| FEV1 | Boys | 3.50 | 93 | BCPE | 2.52005 | 0.7471 | 0.12836 | 9.93817 | 0.7471 | 0.5650 |
| FEV1 | Boys | 3.50 | 94 | BCPE | 2.52005 | 0.7647 | 0.12836 | 9.93817 | 0.7647 | 0.5783 |
| FEV1 | Boys | 3.50 | 95 | BCPE | 2.52005 | 0.7825 | 0.12836 | 9.93817 | 0.7825 | 0.5918 |
| FEV1 | Boys | 3.50 | 96 | BCPE | 2.52005 | 0.8006 | 0.12836 | 9.93817 | 0.8006 | 0.6055 |
| FEV1 | Boys | 3.50 | 97 | BCPE | 2.52005 | 0.8188 | 0.12836 | 9.93817 | 0.8188 | 0.6193 |
| FEV1 | Boys | 3.50 | 98 | BCPE | 2.52005 | 0.8373 | 0.12836 | 9.93817 | 0.8373 | 0.6333 |
| FEV1 | Boys | 3.50 | 99 | BCPE | 2.52005 | 0.8560 | 0.12836 | 9.93817 | 0.8560 | 0.6474 |
| FEV1 | Boys | 3.50 | 100 | BCPE | 2.52005 | 0.8750 | 0.12836 | 9.93817 | 0.8750 | 0.6617 |
| FEV1 | Boys | 3.50 | 101 | BCPE | 2.52005 | 0.8941 | 0.12836 | 9.93817 | 0.8941 | 0.6762 |
| FEV1 | Boys | 3.50 | 102 | BCPE | 2.52005 | 0.9135 | 0.12836 | 9.93817 | 0.9135 | 0.6909 |
| FEV1 | Boys | 3.50 | 103 | BCPE | 2.52005 | 0.9331 | 0.12836 | 9.93817 | 0.9331 | 0.7057 |
| FEV1 | Boys | 3.50 | 104 | BCPE | 2.52005 | 0.9530 | 0.12836 | 9.93817 | 0.9530 | 0.7207 |
| FEV1 | Boys | 3.50 | 105 | BCPE | 2.52005 | 0.9730 | 0.12836 | 9.93817 | 0.9730 | 0.7359 |
| FEV1 | Boys | 3.50 | 106 | BCPE | 2.52005 | 0.9933 | 0.12836 | 9.93817 | 0.9933 | 0.7512 |
| FEV1 | Boys | 3.50 | 107 | BCPE | 2.52005 | 1.0138 | 0.12836 | 9.93817 | 1.0138 | 0.7667 |
| FEV1 | Boys | 3.50 | 108 | BCPE | 2.52005 | 1.0346 | 0.12836 | 9.93817 | 1.0346 | 0.7824 |
| FEV1 | Boys | 3.50 | 109 | BCPE | 2.52005 | 1.0555 | 0.12836 | 9.93817 | 1.0555 | 0.7983 |
| FEV1 | Boys | 3.50 | 110 | BCPE | 2.52005 | 1.0767 | 0.12836 | 9.93817 | 1.0767 | 0.8143 |
| FEV1 | Boys | 3.50 | 111 | BCPE | 2.52005 | 1.0981 | 0.12836 | 9.93817 | 1.0981 | 0.8305 |
| FEV1 | Boys | 3.50 | 112 | BCPE | 2.52005 | 1.1198 | 0.12836 | 9.93817 | 1.1198 | 0.8469 |
| FEV1 | Boys | 3.50 | 113 | BCPE | 2.52005 | 1.1417 | 0.12836 | 9.93817 | 1.1417 | 0.8634 |
| FEV1 | Boys | 3.50 | 114 | BCPE | 2.52005 | 1.1638 | 0.12836 | 9.93817 | 1.1638 | 0.8802 |
| FEV1 | Boys | 3.50 | 115 | BCPE | 2.52005 | 1.1861 | 0.12836 | 9.93817 | 1.1861 | 0.8970 |
| FEV1 | Boys | 3.50 | 116 | BCPE | 2.52005 | 1.2087 | 0.12836 | 9.93817 | 1.2087 | 0.9141 |
| FEV1 | Boys | 3.50 | 117 | BCPE | 2.52005 | 1.2315 | 0.12836 | 9.93817 | 1.2315 | 0.9314 |
| FEV1 | Boys | 3.50 | 118 | BCPE | 2.52005 | 1.2545 | 0.12836 | 9.93817 | 1.2545 | 0.9488 |
| FEV1 | Boys | 3.50 | 119 | BCPE | 2.52005 | 1.2778 | 0.12836 | 9.93817 | 1.2778 | 0.9664 |
| FEV1 | Boys | 3.50 | 120 | BCPE | 2.52005 | 1.3013 | 0.12836 | 9.93817 | 1.3013 | 0.9841 |
| FEV1 | Boys | 3.50 | 121 | BCPE | 2.52005 | 1.3250 | 0.12836 | 9.93817 | 1.3250 | 1.0021 |
| FEV1 | Boys | 3.50 | 122 | BCPE | 2.52005 | 1.3490 | 0.12836 | 9.93817 | 1.3490 | 1.0202 |
| FEV1 | Boys | 3.50 | 123 | BCPE | 2.52005 | 1.3731 | 0.12836 | 9.93817 | 1.3731 | 1.0385 |
| FEV1 | Boys | 3.50 | 124 | BCPE | 2.52005 | 1.3976 | 0.12836 | 9.93817 | 1.3976 | 1.0570 |
| FEV1 | Boys | 3.50 | 125 | BCPE | 2.52005 | 1.4222 | 0.12836 | 9.93817 | 1.4222 | 1.0756 |
| FEV1 | Boys | 3.50 | 126 | BCPE | 2.52005 | 1.4471 | 0.12836 | 9.93817 | 1.4471 | 1.0944 |
| FEV1 | Boys | 3.50 | 127 | BCPE | 2.52005 | 1.4722 | 0.12836 | 9.93817 | 1.4722 | 1.1134 |
| FEV1 | Boys | 3.50 | 128 | BCPE | 2.52005 | 1.4976 | 0.12836 | 9.93817 | 1.4976 | 1.1326 |
| FEV1 | Boys | 3.50 | 129 | BCPE | 2.52005 | 1.5232 | 0.12836 | 9.93817 | 1.5232 | 1.1519 |
| FEV1 | Boys | 3.50 | 130 | BCPE | 2.52005 | 1.5490 | 0.12836 | 9.93817 | 1.5490 | 1.1715 |
| FEV1 | Boys | 3.50 | 131 | BCPE | 2.52005 | 1.5750 | 0.12836 | 9.93817 | 1.5750 | 1.1912 |
| FEV1 | Boys | 3.50 | 132 | BCPE | 2.52005 | 1.6013 | 0.12836 | 9.93817 | 1.6013 | 1.2111 |
| FEV1 | Boys | 3.75 | 79 | BCPE | 2.52897 | 0.5288 | 0.12839 | 9.73307 | 0.5288 | 0.3997 |
| FEV1 | Boys | 3.75 | 80 | BCPE | 2.52897 | 0.5435 | 0.12839 | 9.73307 | 0.5435 | 0.4108 |
| FEV1 | Boys | 3.75 | 81 | BCPE | 2.52897 | 0.5584 | 0.12839 | 9.73307 | 0.5584 | 0.4220 |
| FEV1 | Boys | 3.75 | 82 | BCPE | 2.52897 | 0.5735 | 0.12839 | 9.73307 | 0.5735 | 0.4334 |
| FEV1 | Boys | 3.75 | 83 | BCPE | 2.52897 | 0.5888 | 0.12839 | 9.73307 | 0.5888 | 0.4450 |
| FEV1 | Boys | 3.75 | 84 | BCPE | 2.52897 | 0.6044 | 0.12839 | 9.73307 | 0.6044 | 0.4568 |
| FEV1 | Boys | 3.75 | 85 | BCPE | 2.52897 | 0.6202 | 0.12839 | 9.73307 | 0.6202 | 0.4687 |
| FEV1 | Boys | 3.75 | 86 | BCPE | 2.52897 | 0.6362 | 0.12839 | 9.73307 | 0.6362 | 0.4808 |
| FEV1 | Boys | 3.75 | 87 | BCPE | 2.52897 | 0.6524 | 0.12839 | 9.73307 | 0.6524 | 0.4931 |
| FEV1 | Boys | 3.75 | 88 | BCPE | 2.52897 | 0.6688 | 0.12839 | 9.73307 | 0.6688 | 0.5055 |
| FEV1 | Boys | 3.75 | 89 | BCPE | 2.52897 | 0.6855 | 0.12839 | 9.73307 | 0.6855 | 0.5181 |
| FEV1 | Boys | 3.75 | 90 | BCPE | 2.52897 | 0.7024 | 0.12839 | 9.73307 | 0.7024 | 0.5308 |
| FEV1 | Boys | 3.75 | 91 | BCPE | 2.52897 | 0.7195 | 0.12839 | 9.73307 | 0.7195 | 0.5437 |
| FEV1 | Boys | 3.75 | 92 | BCPE | 2.52897 | 0.7368 | 0.12839 | 9.73307 | 0.7368 | 0.5568 |
| FEV1 | Boys | 3.75 | 93 | BCPE | 2.52897 | 0.7543 | 0.12839 | 9.73307 | 0.7543 | 0.5701 |
| FEV1 | Boys | 3.75 | 94 | BCPE | 2.52897 | 0.7721 | 0.12839 | 9.73307 | 0.7721 | 0.5835 |
| FEV1 | Boys | 3.75 | 95 | BCPE | 2.52897 | 0.7901 | 0.12839 | 9.73307 | 0.7901 | 0.5971 |
| FEV1 | Boys | 3.75 | 96 | BCPE | 2.52897 | 0.8083 | 0.12839 | 9.73307 | 0.8083 | 0.6109 |
| FEV1 | Boys | 3.75 | 97 | BCPE | 2.52897 | 0.8267 | 0.12839 | 9.73307 | 0.8267 | 0.6248 |
| FEV1 | Boys | 3.75 | 98 | BCPE | 2.52897 | 0.8454 | 0.12839 | 9.73307 | 0.8454 | 0.6389 |
| FEV1 | Boys | 3.75 | 99 | BCPE | 2.52897 | 0.8643 | 0.12839 | 9.73307 | 0.8643 | 0.6532 |
| FEV1 | Boys | 3.75 | 100 | BCPE | 2.52897 | 0.8834 | 0.12839 | 9.73307 | 0.8834 | 0.6677 |
| FEV1 | Boys | 3.75 | 101 | BCPE | 2.52897 | 0.9028 | 0.12839 | 9.73307 | 0.9028 | 0.6823 |
| FEV1 | Boys | 3.75 | 102 | BCPE | 2.52897 | 0.9223 | 0.12839 | 9.73307 | 0.9223 | 0.6971 |
| FEV1 | Boys | 3.75 | 103 | BCPE | 2.52897 | 0.9421 | 0.12839 | 9.73307 | 0.9421 | 0.7120 |
| FEV1 | Boys | 3.75 | 104 | BCPE | 2.52897 | 0.9622 | 0.12839 | 9.73307 | 0.9622 | 0.7272 |
| FEV1 | Boys | 3.75 | 105 | BCPE | 2.52897 | 0.9824 | 0.12839 | 9.73307 | 0.9824 | 0.7425 |
| FEV1 | Boys | 3.75 | 106 | BCPE | 2.52897 | 1.0029 | 0.12839 | 9.73307 | 1.0029 | 0.7580 |
| FEV1 | Boys | 3.75 | 107 | BCPE | 2.52897 | 1.0236 | 0.12839 | 9.73307 | 1.0236 | 0.7736 |
| FEV1 | Boys | 3.75 | 108 | BCPE | 2.52897 | 1.0446 | 0.12839 | 9.73307 | 1.0446 | 0.7895 |
| FEV1 | Boys | 3.75 | 109 | BCPE | 2.52897 | 1.0657 | 0.12839 | 9.73307 | 1.0657 | 0.8055 |
| FEV1 | Boys | 3.75 | 110 | BCPE | 2.52897 | 1.0871 | 0.12839 | 9.73307 | 1.0871 | 0.8216 |
| FEV1 | Boys | 3.75 | 111 | BCPE | 2.52897 | 1.1088 | 0.12839 | 9.73307 | 1.1088 | 0.8380 |
| FEV1 | Boys | 3.75 | 112 | BCPE | 2.52897 | 1.1306 | 0.12839 | 9.73307 | 1.1306 | 0.8545 |
| FEV1 | Boys | 3.75 | 113 | BCPE | 2.52897 | 1.1527 | 0.12839 | 9.73307 | 1.1527 | 0.8712 |
| FEV1 | Boys | 3.75 | 114 | BCPE | 2.52897 | 1.1750 | 0.12839 | 9.73307 | 1.1750 | 0.8881 |
| FEV1 | Boys | 3.75 | 115 | BCPE | 2.52897 | 1.1976 | 0.12839 | 9.73307 | 1.1976 | 0.9051 |
| FEV1 | Boys | 3.75 | 116 | BCPE | 2.52897 | 1.2204 | 0.12839 | 9.73307 | 1.2204 | 0.9223 |
| FEV1 | Boys | 3.75 | 117 | BCPE | 2.52897 | 1.2434 | 0.12839 | 9.73307 | 1.2434 | 0.9397 |
| FEV1 | Boys | 3.75 | 118 | BCPE | 2.52897 | 1.2666 | 0.12839 | 9.73307 | 1.2666 | 0.9573 |
| FEV1 | Boys | 3.75 | 119 | BCPE | 2.52897 | 1.2901 | 0.12839 | 9.73307 | 1.2901 | 0.9751 |
| FEV1 | Boys | 3.75 | 120 | BCPE | 2.52897 | 1.3138 | 0.12839 | 9.73307 | 1.3138 | 0.9930 |
| FEV1 | Boys | 3.75 | 121 | BCPE | 2.52897 | 1.3378 | 0.12839 | 9.73307 | 1.3378 | 1.0111 |
| FEV1 | Boys | 3.75 | 122 | BCPE | 2.52897 | 1.3620 | 0.12839 | 9.73307 | 1.3620 | 1.0294 |
| FEV1 | Boys | 3.75 | 123 | BCPE | 2.52897 | 1.3864 | 0.12839 | 9.73307 | 1.3864 | 1.0478 |
| FEV1 | Boys | 3.75 | 124 | BCPE | 2.52897 | 1.4111 | 0.12839 | 9.73307 | 1.4111 | 1.0665 |
| FEV1 | Boys | 3.75 | 125 | BCPE | 2.52897 | 1.4360 | 0.12839 | 9.73307 | 1.4360 | 1.0853 |
| FEV1 | Boys | 3.75 | 126 | BCPE | 2.52897 | 1.4611 | 0.12839 | 9.73307 | 1.4611 | 1.1043 |
| FEV1 | Boys | 3.75 | 127 | BCPE | 2.52897 | 1.4864 | 0.12839 | 9.73307 | 1.4864 | 1.1234 |
| FEV1 | Boys | 3.75 | 128 | BCPE | 2.52897 | 1.5120 | 0.12839 | 9.73307 | 1.5120 | 1.1428 |
| FEV1 | Boys | 3.75 | 129 | BCPE | 2.52897 | 1.5379 | 0.12839 | 9.73307 | 1.5379 | 1.1623 |
| FEV1 | Boys | 3.75 | 130 | BCPE | 2.52897 | 1.5639 | 0.12839 | 9.73307 | 1.5639 | 1.1820 |
| FEV1 | Boys | 3.75 | 131 | BCPE | 2.52897 | 1.5903 | 0.12839 | 9.73307 | 1.5903 | 1.2019 |
| FEV1 | Boys | 3.75 | 132 | BCPE | 2.52897 | 1.6168 | 0.12839 | 9.73307 | 1.6168 | 1.2219 |
| FEV1 | Boys | 4.00 | 79 | BCPE | 2.53731 | 0.5336 | 0.12783 | 9.54505 | 0.5336 | 0.4038 |
| FEV1 | Boys | 4.00 | 80 | BCPE | 2.53731 | 0.5484 | 0.12783 | 9.54505 | 0.5484 | 0.4150 |
| FEV1 | Boys | 4.00 | 81 | BCPE | 2.53731 | 0.5634 | 0.12783 | 9.54505 | 0.5634 | 0.4264 |
| FEV1 | Boys | 4.00 | 82 | BCPE | 2.53731 | 0.5787 | 0.12783 | 9.54505 | 0.5787 | 0.4379 |
| FEV1 | Boys | 4.00 | 83 | BCPE | 2.53731 | 0.5942 | 0.12783 | 9.54505 | 0.5942 | 0.4496 |
| FEV1 | Boys | 4.00 | 84 | BCPE | 2.53731 | 0.6099 | 0.12783 | 9.54505 | 0.6099 | 0.4615 |
| FEV1 | Boys | 4.00 | 85 | BCPE | 2.53731 | 0.6258 | 0.12783 | 9.54505 | 0.6258 | 0.4735 |
| FEV1 | Boys | 4.00 | 86 | BCPE | 2.53731 | 0.6419 | 0.12783 | 9.54505 | 0.6419 | 0.4858 |
| FEV1 | Boys | 4.00 | 87 | BCPE | 2.53731 | 0.6583 | 0.12783 | 9.54505 | 0.6583 | 0.4981 |
| FEV1 | Boys | 4.00 | 88 | BCPE | 2.53731 | 0.6749 | 0.12783 | 9.54505 | 0.6749 | 0.5107 |
| FEV1 | Boys | 4.00 | 89 | BCPE | 2.53731 | 0.6917 | 0.12783 | 9.54505 | 0.6917 | 0.5234 |
| FEV1 | Boys | 4.00 | 90 | BCPE | 2.53731 | 0.7087 | 0.12783 | 9.54505 | 0.7087 | 0.5363 |
| FEV1 | Boys | 4.00 | 91 | BCPE | 2.53731 | 0.7260 | 0.12783 | 9.54505 | 0.7260 | 0.5493 |
| FEV1 | Boys | 4.00 | 92 | BCPE | 2.53731 | 0.7434 | 0.12783 | 9.54505 | 0.7434 | 0.5626 |
| FEV1 | Boys | 4.00 | 93 | BCPE | 2.53731 | 0.7611 | 0.12783 | 9.54505 | 0.7611 | 0.5760 |
| FEV1 | Boys | 4.00 | 94 | BCPE | 2.53731 | 0.7791 | 0.12783 | 9.54505 | 0.7791 | 0.5895 |
| FEV1 | Boys | 4.00 | 95 | BCPE | 2.53731 | 0.7972 | 0.12783 | 9.54505 | 0.7972 | 0.6033 |
| FEV1 | Boys | 4.00 | 96 | BCPE | 2.53731 | 0.8156 | 0.12783 | 9.54505 | 0.8156 | 0.6172 |
| FEV1 | Boys | 4.00 | 97 | BCPE | 2.53731 | 0.8342 | 0.12783 | 9.54505 | 0.8342 | 0.6313 |
| FEV1 | Boys | 4.00 | 98 | BCPE | 2.53731 | 0.8531 | 0.12783 | 9.54505 | 0.8531 | 0.6455 |
| FEV1 | Boys | 4.00 | 99 | BCPE | 2.53731 | 0.8721 | 0.12783 | 9.54505 | 0.8721 | 0.6599 |
| FEV1 | Boys | 4.00 | 100 | BCPE | 2.53731 | 0.8914 | 0.12783 | 9.54505 | 0.8914 | 0.6745 |
| FEV1 | Boys | 4.00 | 101 | BCPE | 2.53731 | 0.9109 | 0.12783 | 9.54505 | 0.9109 | 0.6893 |
| FEV1 | Boys | 4.00 | 102 | BCPE | 2.53731 | 0.9307 | 0.12783 | 9.54505 | 0.9307 | 0.7043 |
| FEV1 | Boys | 4.00 | 103 | BCPE | 2.53731 | 0.9507 | 0.12783 | 9.54505 | 0.9507 | 0.7194 |
| FEV1 | Boys | 4.00 | 104 | BCPE | 2.53731 | 0.9709 | 0.12783 | 9.54505 | 0.9709 | 0.7347 |
| FEV1 | Boys | 4.00 | 105 | BCPE | 2.53731 | 0.9913 | 0.12783 | 9.54505 | 0.9913 | 0.7501 |
| FEV1 | Boys | 4.00 | 106 | BCPE | 2.53731 | 1.0120 | 0.12783 | 9.54505 | 1.0120 | 0.7658 |
| FEV1 | Boys | 4.00 | 107 | BCPE | 2.53731 | 1.0329 | 0.12783 | 9.54505 | 1.0329 | 0.7816 |
| FEV1 | Boys | 4.00 | 108 | BCPE | 2.53731 | 1.0540 | 0.12783 | 9.54505 | 1.0540 | 0.7976 |
| FEV1 | Boys | 4.00 | 109 | BCPE | 2.53731 | 1.0754 | 0.12783 | 9.54505 | 1.0754 | 0.8137 |
| FEV1 | Boys | 4.00 | 110 | BCPE | 2.53731 | 1.0970 | 0.12783 | 9.54505 | 1.0970 | 0.8301 |
| FEV1 | Boys | 4.00 | 111 | BCPE | 2.53731 | 1.1188 | 0.12783 | 9.54505 | 1.1188 | 0.8466 |
| FEV1 | Boys | 4.00 | 112 | BCPE | 2.53731 | 1.1408 | 0.12783 | 9.54505 | 1.1408 | 0.8633 |
| FEV1 | Boys | 4.00 | 113 | BCPE | 2.53731 | 1.1631 | 0.12783 | 9.54505 | 1.1631 | 0.8802 |
| FEV1 | Boys | 4.00 | 114 | BCPE | 2.53731 | 1.1857 | 0.12783 | 9.54505 | 1.1857 | 0.8972 |
| FEV1 | Boys | 4.00 | 115 | BCPE | 2.53731 | 1.2084 | 0.12783 | 9.54505 | 1.2084 | 0.9144 |
| FEV1 | Boys | 4.00 | 116 | BCPE | 2.53731 | 1.2314 | 0.12783 | 9.54505 | 1.2314 | 0.9318 |
| FEV1 | Boys | 4.00 | 117 | BCPE | 2.53731 | 1.2546 | 0.12783 | 9.54505 | 1.2546 | 0.9494 |
| FEV1 | Boys | 4.00 | 118 | BCPE | 2.53731 | 1.2781 | 0.12783 | 9.54505 | 1.2781 | 0.9672 |
| FEV1 | Boys | 4.00 | 119 | BCPE | 2.53731 | 1.3018 | 0.12783 | 9.54505 | 1.3018 | 0.9851 |
| FEV1 | Boys | 4.00 | 120 | BCPE | 2.53731 | 1.3257 | 0.12783 | 9.54505 | 1.3257 | 1.0032 |
| FEV1 | Boys | 4.00 | 121 | BCPE | 2.53731 | 1.3499 | 0.12783 | 9.54505 | 1.3499 | 1.0215 |
| FEV1 | Boys | 4.00 | 122 | BCPE | 2.53731 | 1.3743 | 0.12783 | 9.54505 | 1.3743 | 1.0400 |
| FEV1 | Boys | 4.00 | 123 | BCPE | 2.53731 | 1.3989 | 0.12783 | 9.54505 | 1.3989 | 1.0586 |
| FEV1 | Boys | 4.00 | 124 | BCPE | 2.53731 | 1.4238 | 0.12783 | 9.54505 | 1.4238 | 1.0774 |
| FEV1 | Boys | 4.00 | 125 | BCPE | 2.53731 | 1.4489 | 0.12783 | 9.54505 | 1.4489 | 1.0964 |
| FEV1 | Boys | 4.00 | 126 | BCPE | 2.53731 | 1.4743 | 0.12783 | 9.54505 | 1.4743 | 1.1156 |
| FEV1 | Boys | 4.00 | 127 | BCPE | 2.53731 | 1.4999 | 0.12783 | 9.54505 | 1.4999 | 1.1350 |
| FEV1 | Boys | 4.00 | 128 | BCPE | 2.53731 | 1.5257 | 0.12783 | 9.54505 | 1.5257 | 1.1545 |
| FEV1 | Boys | 4.00 | 129 | BCPE | 2.53731 | 1.5518 | 0.12783 | 9.54505 | 1.5518 | 1.1743 |
| FEV1 | Boys | 4.00 | 130 | BCPE | 2.53731 | 1.5781 | 0.12783 | 9.54505 | 1.5781 | 1.1942 |
| FEV1 | Boys | 4.00 | 131 | BCPE | 2.53731 | 1.6046 | 0.12783 | 9.54505 | 1.6046 | 1.2142 |
| FEV1 | Boys | 4.00 | 132 | BCPE | 2.53731 | 1.6314 | 0.12783 | 9.54505 | 1.6314 | 1.2345 |
| FEV1 | Boys | 4.25 | 79 | BCPE | 2.54515 | 0.5381 | 0.12684 | 9.37175 | 0.5381 | 0.4083 |
| FEV1 | Boys | 4.25 | 80 | BCPE | 2.54515 | 0.5531 | 0.12684 | 9.37175 | 0.5531 | 0.4196 |
| FEV1 | Boys | 4.25 | 81 | BCPE | 2.54515 | 0.5682 | 0.12684 | 9.37175 | 0.5682 | 0.4311 |
| FEV1 | Boys | 4.25 | 82 | BCPE | 2.54515 | 0.5836 | 0.12684 | 9.37175 | 0.5836 | 0.4428 |
| FEV1 | Boys | 4.25 | 83 | BCPE | 2.54515 | 0.5992 | 0.12684 | 9.37175 | 0.5992 | 0.4546 |
| FEV1 | Boys | 4.25 | 84 | BCPE | 2.54515 | 0.6150 | 0.12684 | 9.37175 | 0.6150 | 0.4666 |
| FEV1 | Boys | 4.25 | 85 | BCPE | 2.54515 | 0.6311 | 0.12684 | 9.37175 | 0.6311 | 0.4788 |
| FEV1 | Boys | 4.25 | 86 | BCPE | 2.54515 | 0.6474 | 0.12684 | 9.37175 | 0.6474 | 0.4912 |
| FEV1 | Boys | 4.25 | 87 | BCPE | 2.54515 | 0.6639 | 0.12684 | 9.37175 | 0.6639 | 0.5037 |
| FEV1 | Boys | 4.25 | 88 | BCPE | 2.54515 | 0.6806 | 0.12684 | 9.37175 | 0.6806 | 0.5164 |
| FEV1 | Boys | 4.25 | 89 | BCPE | 2.54515 | 0.6975 | 0.12684 | 9.37175 | 0.6975 | 0.5292 |
| FEV1 | Boys | 4.25 | 90 | BCPE | 2.54515 | 0.7147 | 0.12684 | 9.37175 | 0.7147 | 0.5423 |
| FEV1 | Boys | 4.25 | 91 | BCPE | 2.54515 | 0.7321 | 0.12684 | 9.37175 | 0.7321 | 0.5555 |
| FEV1 | Boys | 4.25 | 92 | BCPE | 2.54515 | 0.7497 | 0.12684 | 9.37175 | 0.7497 | 0.5688 |
| FEV1 | Boys | 4.25 | 93 | BCPE | 2.54515 | 0.7676 | 0.12684 | 9.37175 | 0.7676 | 0.5824 |
| FEV1 | Boys | 4.25 | 94 | BCPE | 2.54515 | 0.7857 | 0.12684 | 9.37175 | 0.7857 | 0.5961 |
| FEV1 | Boys | 4.25 | 95 | BCPE | 2.54515 | 0.8040 | 0.12684 | 9.37175 | 0.8040 | 0.6100 |
| FEV1 | Boys | 4.25 | 96 | BCPE | 2.54515 | 0.8225 | 0.12684 | 9.37175 | 0.8225 | 0.6241 |
| FEV1 | Boys | 4.25 | 97 | BCPE | 2.54515 | 0.8413 | 0.12684 | 9.37175 | 0.8413 | 0.6383 |
| FEV1 | Boys | 4.25 | 98 | BCPE | 2.54515 | 0.8603 | 0.12684 | 9.37175 | 0.8603 | 0.6527 |
| FEV1 | Boys | 4.25 | 99 | BCPE | 2.54515 | 0.8795 | 0.12684 | 9.37175 | 0.8795 | 0.6673 |
| FEV1 | Boys | 4.25 | 100 | BCPE | 2.54515 | 0.8990 | 0.12684 | 9.37175 | 0.8990 | 0.6821 |
| FEV1 | Boys | 4.25 | 101 | BCPE | 2.54515 | 0.9187 | 0.12684 | 9.37175 | 0.9187 | 0.6970 |
| FEV1 | Boys | 4.25 | 102 | BCPE | 2.54515 | 0.9386 | 0.12684 | 9.37175 | 0.9386 | 0.7121 |
| FEV1 | Boys | 4.25 | 103 | BCPE | 2.54515 | 0.9587 | 0.12684 | 9.37175 | 0.9587 | 0.7274 |
| FEV1 | Boys | 4.25 | 104 | BCPE | 2.54515 | 0.9791 | 0.12684 | 9.37175 | 0.9791 | 0.7429 |
| FEV1 | Boys | 4.25 | 105 | BCPE | 2.54515 | 0.9997 | 0.12684 | 9.37175 | 0.9997 | 0.7585 |
| FEV1 | Boys | 4.25 | 106 | BCPE | 2.54515 | 1.0206 | 0.12684 | 9.37175 | 1.0206 | 0.7743 |
| FEV1 | Boys | 4.25 | 107 | BCPE | 2.54515 | 1.0416 | 0.12684 | 9.37175 | 1.0416 | 0.7903 |
| FEV1 | Boys | 4.25 | 108 | BCPE | 2.54515 | 1.0629 | 0.12684 | 9.37175 | 1.0629 | 0.8065 |
| FEV1 | Boys | 4.25 | 109 | BCPE | 2.54515 | 1.0845 | 0.12684 | 9.37175 | 1.0845 | 0.8228 |
| FEV1 | Boys | 4.25 | 110 | BCPE | 2.54515 | 1.1063 | 0.12684 | 9.37175 | 1.1063 | 0.8393 |
| FEV1 | Boys | 4.25 | 111 | BCPE | 2.54515 | 1.1283 | 0.12684 | 9.37175 | 1.1283 | 0.8560 |
| FEV1 | Boys | 4.25 | 112 | BCPE | 2.54515 | 1.1505 | 0.12684 | 9.37175 | 1.1505 | 0.8729 |
| FEV1 | Boys | 4.25 | 113 | BCPE | 2.54515 | 1.1730 | 0.12684 | 9.37175 | 1.1730 | 0.8900 |
| FEV1 | Boys | 4.25 | 114 | BCPE | 2.54515 | 1.1957 | 0.12684 | 9.37175 | 1.1957 | 0.9072 |
| FEV1 | Boys | 4.25 | 115 | BCPE | 2.54515 | 1.2187 | 0.12684 | 9.37175 | 1.2187 | 0.9246 |
| FEV1 | Boys | 4.25 | 116 | BCPE | 2.54515 | 1.2419 | 0.12684 | 9.37175 | 1.2419 | 0.9422 |
| FEV1 | Boys | 4.25 | 117 | BCPE | 2.54515 | 1.2653 | 0.12684 | 9.37175 | 1.2653 | 0.9600 |
| FEV1 | Boys | 4.25 | 118 | BCPE | 2.54515 | 1.2889 | 0.12684 | 9.37175 | 1.2889 | 0.9779 |
| FEV1 | Boys | 4.25 | 119 | BCPE | 2.54515 | 1.3128 | 0.12684 | 9.37175 | 1.3128 | 0.9961 |
| FEV1 | Boys | 4.25 | 120 | BCPE | 2.54515 | 1.3370 | 0.12684 | 9.37175 | 1.3370 | 1.0144 |
| FEV1 | Boys | 4.25 | 121 | BCPE | 2.54515 | 1.3613 | 0.12684 | 9.37175 | 1.3613 | 1.0329 |
| FEV1 | Boys | 4.25 | 122 | BCPE | 2.54515 | 1.3860 | 0.12684 | 9.37175 | 1.3860 | 1.0516 |
| FEV1 | Boys | 4.25 | 123 | BCPE | 2.54515 | 1.4108 | 0.12684 | 9.37175 | 1.4108 | 1.0704 |
| FEV1 | Boys | 4.25 | 124 | BCPE | 2.54515 | 1.4359 | 0.12684 | 9.37175 | 1.4359 | 1.0894 |
| FEV1 | Boys | 4.25 | 125 | BCPE | 2.54515 | 1.4612 | 0.12684 | 9.37175 | 1.4612 | 1.1087 |
| FEV1 | Boys | 4.25 | 126 | BCPE | 2.54515 | 1.4868 | 0.12684 | 9.37175 | 1.4868 | 1.1281 |
| FEV1 | Boys | 4.25 | 127 | BCPE | 2.54515 | 1.5126 | 0.12684 | 9.37175 | 1.5126 | 1.1476 |
| FEV1 | Boys | 4.25 | 128 | BCPE | 2.54515 | 1.5387 | 0.12684 | 9.37175 | 1.5387 | 1.1674 |
| FEV1 | Boys | 4.25 | 129 | BCPE | 2.54515 | 1.5649 | 0.12684 | 9.37175 | 1.5649 | 1.1874 |
| FEV1 | Boys | 4.25 | 130 | BCPE | 2.54515 | 1.5915 | 0.12684 | 9.37175 | 1.5915 | 1.2075 |
| FEV1 | Boys | 4.25 | 131 | BCPE | 2.54515 | 1.6182 | 0.12684 | 9.37175 | 1.6182 | 1.2278 |
| FEV1 | Boys | 4.25 | 132 | BCPE | 2.54515 | 1.6453 | 0.12684 | 9.37175 | 1.6453 | 1.2483 |
| FEV1 | Boys | 4.50 | 79 | BCPE | 2.55254 | 0.5424 | 0.12550 | 9.21123 | 0.5424 | 0.4131 |
| FEV1 | Boys | 4.50 | 80 | BCPE | 2.55254 | 0.5575 | 0.12550 | 9.21123 | 0.5575 | 0.4245 |
| FEV1 | Boys | 4.50 | 81 | BCPE | 2.55254 | 0.5727 | 0.12550 | 9.21123 | 0.5727 | 0.4362 |
| FEV1 | Boys | 4.50 | 82 | BCPE | 2.55254 | 0.5883 | 0.12550 | 9.21123 | 0.5883 | 0.4480 |
| FEV1 | Boys | 4.50 | 83 | BCPE | 2.55254 | 0.6040 | 0.12550 | 9.21123 | 0.6040 | 0.4600 |
| FEV1 | Boys | 4.50 | 84 | BCPE | 2.55254 | 0.6199 | 0.12550 | 9.21123 | 0.6199 | 0.4721 |
| FEV1 | Boys | 4.50 | 85 | BCPE | 2.55254 | 0.6361 | 0.12550 | 9.21123 | 0.6361 | 0.4844 |
| FEV1 | Boys | 4.50 | 86 | BCPE | 2.55254 | 0.6525 | 0.12550 | 9.21123 | 0.6525 | 0.4969 |
| FEV1 | Boys | 4.50 | 87 | BCPE | 2.55254 | 0.6692 | 0.12550 | 9.21123 | 0.6692 | 0.5096 |
| FEV1 | Boys | 4.50 | 88 | BCPE | 2.55254 | 0.6860 | 0.12550 | 9.21123 | 0.6860 | 0.5224 |
| FEV1 | Boys | 4.50 | 89 | BCPE | 2.55254 | 0.7031 | 0.12550 | 9.21123 | 0.7031 | 0.5355 |
| FEV1 | Boys | 4.50 | 90 | BCPE | 2.55254 | 0.7204 | 0.12550 | 9.21123 | 0.7204 | 0.5486 |
| FEV1 | Boys | 4.50 | 91 | BCPE | 2.55254 | 0.7379 | 0.12550 | 9.21123 | 0.7379 | 0.5620 |
| FEV1 | Boys | 4.50 | 92 | BCPE | 2.55254 | 0.7557 | 0.12550 | 9.21123 | 0.7557 | 0.5755 |
| FEV1 | Boys | 4.50 | 93 | BCPE | 2.55254 | 0.7737 | 0.12550 | 9.21123 | 0.7737 | 0.5892 |
| FEV1 | Boys | 4.50 | 94 | BCPE | 2.55254 | 0.7919 | 0.12550 | 9.21123 | 0.7919 | 0.6031 |
| FEV1 | Boys | 4.50 | 95 | BCPE | 2.55254 | 0.8104 | 0.12550 | 9.21123 | 0.8104 | 0.6172 |
| FEV1 | Boys | 4.50 | 96 | BCPE | 2.55254 | 0.8291 | 0.12550 | 9.21123 | 0.8291 | 0.6314 |
| FEV1 | Boys | 4.50 | 97 | BCPE | 2.55254 | 0.8480 | 0.12550 | 9.21123 | 0.8480 | 0.6458 |
| FEV1 | Boys | 4.50 | 98 | BCPE | 2.55254 | 0.8671 | 0.12550 | 9.21123 | 0.8671 | 0.6604 |
| FEV1 | Boys | 4.50 | 99 | BCPE | 2.55254 | 0.8865 | 0.12550 | 9.21123 | 0.8865 | 0.6751 |
| FEV1 | Boys | 4.50 | 100 | BCPE | 2.55254 | 0.9061 | 0.12550 | 9.21123 | 0.9061 | 0.6901 |
| FEV1 | Boys | 4.50 | 101 | BCPE | 2.55254 | 0.9260 | 0.12550 | 9.21123 | 0.9260 | 0.7052 |
| FEV1 | Boys | 4.50 | 102 | BCPE | 2.55254 | 0.9460 | 0.12550 | 9.21123 | 0.9460 | 0.7205 |
| FEV1 | Boys | 4.50 | 103 | BCPE | 2.55254 | 0.9664 | 0.12550 | 9.21123 | 0.9664 | 0.7359 |
| FEV1 | Boys | 4.50 | 104 | BCPE | 2.55254 | 0.9869 | 0.12550 | 9.21123 | 0.9869 | 0.7516 |
| FEV1 | Boys | 4.50 | 105 | BCPE | 2.55254 | 1.0077 | 0.12550 | 9.21123 | 1.0077 | 0.7674 |
| FEV1 | Boys | 4.50 | 106 | BCPE | 2.55254 | 1.0287 | 0.12550 | 9.21123 | 1.0287 | 0.7834 |
| FEV1 | Boys | 4.50 | 107 | BCPE | 2.55254 | 1.0499 | 0.12550 | 9.21123 | 1.0499 | 0.7996 |
| FEV1 | Boys | 4.50 | 108 | BCPE | 2.55254 | 1.0714 | 0.12550 | 9.21123 | 1.0714 | 0.8159 |
| FEV1 | Boys | 4.50 | 109 | BCPE | 2.55254 | 1.0931 | 0.12550 | 9.21123 | 1.0931 | 0.8325 |
| FEV1 | Boys | 4.50 | 110 | BCPE | 2.55254 | 1.1151 | 0.12550 | 9.21123 | 1.1151 | 0.8492 |
| FEV1 | Boys | 4.50 | 111 | BCPE | 2.55254 | 1.1373 | 0.12550 | 9.21123 | 1.1373 | 0.8661 |
| FEV1 | Boys | 4.50 | 112 | BCPE | 2.55254 | 1.1597 | 0.12550 | 9.21123 | 1.1597 | 0.8832 |
| FEV1 | Boys | 4.50 | 113 | BCPE | 2.55254 | 1.1823 | 0.12550 | 9.21123 | 1.1823 | 0.9004 |
| FEV1 | Boys | 4.50 | 114 | BCPE | 2.55254 | 1.2052 | 0.12550 | 9.21123 | 1.2052 | 0.9179 |
| FEV1 | Boys | 4.50 | 115 | BCPE | 2.55254 | 1.2284 | 0.12550 | 9.21123 | 1.2284 | 0.9355 |
| FEV1 | Boys | 4.50 | 116 | BCPE | 2.55254 | 1.2517 | 0.12550 | 9.21123 | 1.2517 | 0.9533 |
| FEV1 | Boys | 4.50 | 117 | BCPE | 2.55254 | 1.2754 | 0.12550 | 9.21123 | 1.2754 | 0.9713 |
| FEV1 | Boys | 4.50 | 118 | BCPE | 2.55254 | 1.2992 | 0.12550 | 9.21123 | 1.2992 | 0.9894 |
| FEV1 | Boys | 4.50 | 119 | BCPE | 2.55254 | 1.3233 | 0.12550 | 9.21123 | 1.3233 | 1.0078 |
| FEV1 | Boys | 4.50 | 120 | BCPE | 2.55254 | 1.3476 | 0.12550 | 9.21123 | 1.3476 | 1.0263 |
| FEV1 | Boys | 4.50 | 121 | BCPE | 2.55254 | 1.3722 | 0.12550 | 9.21123 | 1.3722 | 1.0450 |
| FEV1 | Boys | 4.50 | 122 | BCPE | 2.55254 | 1.3970 | 0.12550 | 9.21123 | 1.3970 | 1.0639 |
| FEV1 | Boys | 4.50 | 123 | BCPE | 2.55254 | 1.4220 | 0.12550 | 9.21123 | 1.4220 | 1.0830 |
| FEV1 | Boys | 4.50 | 124 | BCPE | 2.55254 | 1.4473 | 0.12550 | 9.21123 | 1.4473 | 1.1022 |
| FEV1 | Boys | 4.50 | 125 | BCPE | 2.55254 | 1.4729 | 0.12550 | 9.21123 | 1.4729 | 1.1217 |
| FEV1 | Boys | 4.50 | 126 | BCPE | 2.55254 | 1.4986 | 0.12550 | 9.21123 | 1.4986 | 1.1413 |
| FEV1 | Boys | 4.50 | 127 | BCPE | 2.55254 | 1.5246 | 0.12550 | 9.21123 | 1.5246 | 1.1611 |
| FEV1 | Boys | 4.50 | 128 | BCPE | 2.55254 | 1.5509 | 0.12550 | 9.21123 | 1.5509 | 1.1811 |
| FEV1 | Boys | 4.50 | 129 | BCPE | 2.55254 | 1.5774 | 0.12550 | 9.21123 | 1.5774 | 1.2013 |
| FEV1 | Boys | 4.50 | 130 | BCPE | 2.55254 | 1.6041 | 0.12550 | 9.21123 | 1.6041 | 1.2217 |
| FEV1 | Boys | 4.50 | 131 | BCPE | 2.55254 | 1.6311 | 0.12550 | 9.21123 | 1.6311 | 1.2422 |
| FEV1 | Boys | 4.50 | 132 | BCPE | 2.55254 | 1.6584 | 0.12550 | 9.21123 | 1.6584 | 1.2629 |
| FEV1 | Boys | 4.75 | 79 | BCPE | 2.55954 | 0.5465 | 0.12376 | 9.06193 | 0.5465 | 0.4183 |
| FEV1 | Boys | 4.75 | 80 | BCPE | 2.55954 | 0.5616 | 0.12376 | 9.06193 | 0.5616 | 0.4299 |
| FEV1 | Boys | 4.75 | 81 | BCPE | 2.55954 | 0.5770 | 0.12376 | 9.06193 | 0.5770 | 0.4416 |
| FEV1 | Boys | 4.75 | 82 | BCPE | 2.55954 | 0.5926 | 0.12376 | 9.06193 | 0.5926 | 0.4536 |
| FEV1 | Boys | 4.75 | 83 | BCPE | 2.55954 | 0.6085 | 0.12376 | 9.06193 | 0.6085 | 0.4657 |
| FEV1 | Boys | 4.75 | 84 | BCPE | 2.55954 | 0.6246 | 0.12376 | 9.06193 | 0.6246 | 0.4780 |
| FEV1 | Boys | 4.75 | 85 | BCPE | 2.55954 | 0.6409 | 0.12376 | 9.06193 | 0.6409 | 0.4905 |
| FEV1 | Boys | 4.75 | 86 | BCPE | 2.55954 | 0.6574 | 0.12376 | 9.06193 | 0.6574 | 0.5032 |
| FEV1 | Boys | 4.75 | 87 | BCPE | 2.55954 | 0.6741 | 0.12376 | 9.06193 | 0.6741 | 0.5160 |
| FEV1 | Boys | 4.75 | 88 | BCPE | 2.55954 | 0.6911 | 0.12376 | 9.06193 | 0.6911 | 0.5290 |
| FEV1 | Boys | 4.75 | 89 | BCPE | 2.55954 | 0.7083 | 0.12376 | 9.06193 | 0.7083 | 0.5422 |
| FEV1 | Boys | 4.75 | 90 | BCPE | 2.55954 | 0.7258 | 0.12376 | 9.06193 | 0.7258 | 0.5555 |
| FEV1 | Boys | 4.75 | 91 | BCPE | 2.55954 | 0.7435 | 0.12376 | 9.06193 | 0.7435 | 0.5690 |
| FEV1 | Boys | 4.75 | 92 | BCPE | 2.55954 | 0.7614 | 0.12376 | 9.06193 | 0.7614 | 0.5827 |
| FEV1 | Boys | 4.75 | 93 | BCPE | 2.55954 | 0.7795 | 0.12376 | 9.06193 | 0.7795 | 0.5966 |
| FEV1 | Boys | 4.75 | 94 | BCPE | 2.55954 | 0.7978 | 0.12376 | 9.06193 | 0.7978 | 0.6107 |
| FEV1 | Boys | 4.75 | 95 | BCPE | 2.55954 | 0.8164 | 0.12376 | 9.06193 | 0.8164 | 0.6249 |
| FEV1 | Boys | 4.75 | 96 | BCPE | 2.55954 | 0.8353 | 0.12376 | 9.06193 | 0.8353 | 0.6393 |
| FEV1 | Boys | 4.75 | 97 | BCPE | 2.55954 | 0.8543 | 0.12376 | 9.06193 | 0.8543 | 0.6539 |
| FEV1 | Boys | 4.75 | 98 | BCPE | 2.55954 | 0.8736 | 0.12376 | 9.06193 | 0.8736 | 0.6687 |
| FEV1 | Boys | 4.75 | 99 | BCPE | 2.55954 | 0.8931 | 0.12376 | 9.06193 | 0.8931 | 0.6836 |
| FEV1 | Boys | 4.75 | 100 | BCPE | 2.55954 | 0.9129 | 0.12376 | 9.06193 | 0.9129 | 0.6987 |
| FEV1 | Boys | 4.75 | 101 | BCPE | 2.55954 | 0.9329 | 0.12376 | 9.06193 | 0.9329 | 0.7140 |
| FEV1 | Boys | 4.75 | 102 | BCPE | 2.55954 | 0.9531 | 0.12376 | 9.06193 | 0.9531 | 0.7295 |
| FEV1 | Boys | 4.75 | 103 | BCPE | 2.55954 | 0.9736 | 0.12376 | 9.06193 | 0.9736 | 0.7452 |
| FEV1 | Boys | 4.75 | 104 | BCPE | 2.55954 | 0.9943 | 0.12376 | 9.06193 | 0.9943 | 0.7610 |
| FEV1 | Boys | 4.75 | 105 | BCPE | 2.55954 | 1.0152 | 0.12376 | 9.06193 | 1.0152 | 0.7770 |
| FEV1 | Boys | 4.75 | 106 | BCPE | 2.55954 | 1.0364 | 0.12376 | 9.06193 | 1.0364 | 0.7932 |
| FEV1 | Boys | 4.75 | 107 | BCPE | 2.55954 | 1.0578 | 0.12376 | 9.06193 | 1.0578 | 0.8096 |
| FEV1 | Boys | 4.75 | 108 | BCPE | 2.55954 | 1.0794 | 0.12376 | 9.06193 | 1.0794 | 0.8262 |
| FEV1 | Boys | 4.75 | 109 | BCPE | 2.55954 | 1.1013 | 0.12376 | 9.06193 | 1.1013 | 0.8429 |
| FEV1 | Boys | 4.75 | 110 | BCPE | 2.55954 | 1.1234 | 0.12376 | 9.06193 | 1.1234 | 0.8598 |
| FEV1 | Boys | 4.75 | 111 | BCPE | 2.55954 | 1.1457 | 0.12376 | 9.06193 | 1.1457 | 0.8769 |
| FEV1 | Boys | 4.75 | 112 | BCPE | 2.55954 | 1.1683 | 0.12376 | 9.06193 | 1.1683 | 0.8942 |
| FEV1 | Boys | 4.75 | 113 | BCPE | 2.55954 | 1.1912 | 0.12376 | 9.06193 | 1.1912 | 0.9117 |
| FEV1 | Boys | 4.75 | 114 | BCPE | 2.55954 | 1.2142 | 0.12376 | 9.06193 | 1.2142 | 0.9294 |
| FEV1 | Boys | 4.75 | 115 | BCPE | 2.55954 | 1.2375 | 0.12376 | 9.06193 | 1.2375 | 0.9472 |
| FEV1 | Boys | 4.75 | 116 | BCPE | 2.55954 | 1.2611 | 0.12376 | 9.06193 | 1.2611 | 0.9652 |
| FEV1 | Boys | 4.75 | 117 | BCPE | 2.55954 | 1.2849 | 0.12376 | 9.06193 | 1.2849 | 0.9834 |
| FEV1 | Boys | 4.75 | 118 | BCPE | 2.55954 | 1.3089 | 0.12376 | 9.06193 | 1.3089 | 1.0018 |
| FEV1 | Boys | 4.75 | 119 | BCPE | 2.55954 | 1.3332 | 0.12376 | 9.06193 | 1.3332 | 1.0204 |
| FEV1 | Boys | 4.75 | 120 | BCPE | 2.55954 | 1.3577 | 0.12376 | 9.06193 | 1.3577 | 1.0392 |
| FEV1 | Boys | 4.75 | 121 | BCPE | 2.55954 | 1.3824 | 0.12376 | 9.06193 | 1.3824 | 1.0581 |
| FEV1 | Boys | 4.75 | 122 | BCPE | 2.55954 | 1.4074 | 0.12376 | 9.06193 | 1.4074 | 1.0772 |
| FEV1 | Boys | 4.75 | 123 | BCPE | 2.55954 | 1.4327 | 0.12376 | 9.06193 | 1.4327 | 1.0965 |
| FEV1 | Boys | 4.75 | 124 | BCPE | 2.55954 | 1.4581 | 0.12376 | 9.06193 | 1.4581 | 1.1160 |
| FEV1 | Boys | 4.75 | 125 | BCPE | 2.55954 | 1.4839 | 0.12376 | 9.06193 | 1.4839 | 1.1357 |
| FEV1 | Boys | 4.75 | 126 | BCPE | 2.55954 | 1.5098 | 0.12376 | 9.06193 | 1.5098 | 1.1556 |
| FEV1 | Boys | 4.75 | 127 | BCPE | 2.55954 | 1.5360 | 0.12376 | 9.06193 | 1.5360 | 1.1757 |
| FEV1 | Boys | 4.75 | 128 | BCPE | 2.55954 | 1.5625 | 0.12376 | 9.06193 | 1.5625 | 1.1959 |
| FEV1 | Boys | 4.75 | 129 | BCPE | 2.55954 | 1.5892 | 0.12376 | 9.06193 | 1.5892 | 1.2163 |
| FEV1 | Boys | 4.75 | 130 | BCPE | 2.55954 | 1.6161 | 0.12376 | 9.06193 | 1.6161 | 1.2370 |
| FEV1 | Boys | 4.75 | 131 | BCPE | 2.55954 | 1.6433 | 0.12376 | 9.06193 | 1.6433 | 1.2578 |
| FEV1 | Boys | 4.75 | 132 | BCPE | 2.55954 | 1.6707 | 0.12376 | 9.06193 | 1.6707 | 1.2788 |
| FEV1 | Boys | 5.00 | 79 | BCPE | 2.56617 | 0.5503 | 0.12172 | 8.92253 | 0.5503 | 0.4237 |
| FEV1 | Boys | 5.00 | 80 | BCPE | 2.56617 | 0.5656 | 0.12172 | 8.92253 | 0.5656 | 0.4355 |
| FEV1 | Boys | 5.00 | 81 | BCPE | 2.56617 | 0.5811 | 0.12172 | 8.92253 | 0.5811 | 0.4474 |
| FEV1 | Boys | 5.00 | 82 | BCPE | 2.56617 | 0.5968 | 0.12172 | 8.92253 | 0.5968 | 0.4595 |
| FEV1 | Boys | 5.00 | 83 | BCPE | 2.56617 | 0.6128 | 0.12172 | 8.92253 | 0.6128 | 0.4718 |
| FEV1 | Boys | 5.00 | 84 | BCPE | 2.56617 | 0.6290 | 0.12172 | 8.92253 | 0.6290 | 0.4843 |
| FEV1 | Boys | 5.00 | 85 | BCPE | 2.56617 | 0.6454 | 0.12172 | 8.92253 | 0.6454 | 0.4969 |
| FEV1 | Boys | 5.00 | 86 | BCPE | 2.56617 | 0.6620 | 0.12172 | 8.92253 | 0.6620 | 0.5097 |
| FEV1 | Boys | 5.00 | 87 | BCPE | 2.56617 | 0.6789 | 0.12172 | 8.92253 | 0.6789 | 0.5227 |
| FEV1 | Boys | 5.00 | 88 | BCPE | 2.56617 | 0.6960 | 0.12172 | 8.92253 | 0.6960 | 0.5359 |
| FEV1 | Boys | 5.00 | 89 | BCPE | 2.56617 | 0.7133 | 0.12172 | 8.92253 | 0.7133 | 0.5492 |
| FEV1 | Boys | 5.00 | 90 | BCPE | 2.56617 | 0.7309 | 0.12172 | 8.92253 | 0.7309 | 0.5627 |
| FEV1 | Boys | 5.00 | 91 | BCPE | 2.56617 | 0.7487 | 0.12172 | 8.92253 | 0.7487 | 0.5764 |
| FEV1 | Boys | 5.00 | 92 | BCPE | 2.56617 | 0.7667 | 0.12172 | 8.92253 | 0.7667 | 0.5903 |
| FEV1 | Boys | 5.00 | 93 | BCPE | 2.56617 | 0.7850 | 0.12172 | 8.92253 | 0.7850 | 0.6044 |
| FEV1 | Boys | 5.00 | 94 | BCPE | 2.56617 | 0.8035 | 0.12172 | 8.92253 | 0.8035 | 0.6186 |
| FEV1 | Boys | 5.00 | 95 | BCPE | 2.56617 | 0.8222 | 0.12172 | 8.92253 | 0.8222 | 0.6330 |
| FEV1 | Boys | 5.00 | 96 | BCPE | 2.56617 | 0.8411 | 0.12172 | 8.92253 | 0.8411 | 0.6476 |
| FEV1 | Boys | 5.00 | 97 | BCPE | 2.56617 | 0.8603 | 0.12172 | 8.92253 | 0.8603 | 0.6624 |
| FEV1 | Boys | 5.00 | 98 | BCPE | 2.56617 | 0.8798 | 0.12172 | 8.92253 | 0.8798 | 0.6773 |
| FEV1 | Boys | 5.00 | 99 | BCPE | 2.56617 | 0.8994 | 0.12172 | 8.92253 | 0.8994 | 0.6925 |
| FEV1 | Boys | 5.00 | 100 | BCPE | 2.56617 | 0.9193 | 0.12172 | 8.92253 | 0.9193 | 0.7078 |
| FEV1 | Boys | 5.00 | 101 | BCPE | 2.56617 | 0.9394 | 0.12172 | 8.92253 | 0.9394 | 0.7233 |
| FEV1 | Boys | 5.00 | 102 | BCPE | 2.56617 | 0.9598 | 0.12172 | 8.92253 | 0.9598 | 0.7390 |
| FEV1 | Boys | 5.00 | 103 | BCPE | 2.56617 | 0.9804 | 0.12172 | 8.92253 | 0.9804 | 0.7548 |
| FEV1 | Boys | 5.00 | 104 | BCPE | 2.56617 | 1.0013 | 0.12172 | 8.92253 | 1.0013 | 0.7709 |
| FEV1 | Boys | 5.00 | 105 | BCPE | 2.56617 | 1.0223 | 0.12172 | 8.92253 | 1.0223 | 0.7871 |
| FEV1 | Boys | 5.00 | 106 | BCPE | 2.56617 | 1.0436 | 0.12172 | 8.92253 | 1.0436 | 0.8035 |
| FEV1 | Boys | 5.00 | 107 | BCPE | 2.56617 | 1.0652 | 0.12172 | 8.92253 | 1.0652 | 0.8201 |
| FEV1 | Boys | 5.00 | 108 | BCPE | 2.56617 | 1.0870 | 0.12172 | 8.92253 | 1.0870 | 0.8369 |
| FEV1 | Boys | 5.00 | 109 | BCPE | 2.56617 | 1.1090 | 0.12172 | 8.92253 | 1.1090 | 0.8539 |
| FEV1 | Boys | 5.00 | 110 | BCPE | 2.56617 | 1.1313 | 0.12172 | 8.92253 | 1.1313 | 0.8710 |
| FEV1 | Boys | 5.00 | 111 | BCPE | 2.56617 | 1.1538 | 0.12172 | 8.92253 | 1.1538 | 0.8883 |
| FEV1 | Boys | 5.00 | 112 | BCPE | 2.56617 | 1.1765 | 0.12172 | 8.92253 | 1.1765 | 0.9059 |
| FEV1 | Boys | 5.00 | 113 | BCPE | 2.56617 | 1.1995 | 0.12172 | 8.92253 | 1.1995 | 0.9236 |
| FEV1 | Boys | 5.00 | 114 | BCPE | 2.56617 | 1.2228 | 0.12172 | 8.92253 | 1.2228 | 0.9414 |
| FEV1 | Boys | 5.00 | 115 | BCPE | 2.56617 | 1.2462 | 0.12172 | 8.92253 | 1.2462 | 0.9595 |
| FEV1 | Boys | 5.00 | 116 | BCPE | 2.56617 | 1.2700 | 0.12172 | 8.92253 | 1.2700 | 0.9778 |
| FEV1 | Boys | 5.00 | 117 | BCPE | 2.56617 | 1.2939 | 0.12172 | 8.92253 | 1.2939 | 0.9962 |
| FEV1 | Boys | 5.00 | 118 | BCPE | 2.56617 | 1.3181 | 0.12172 | 8.92253 | 1.3181 | 1.0148 |
| FEV1 | Boys | 5.00 | 119 | BCPE | 2.56617 | 1.3425 | 0.12172 | 8.92253 | 1.3425 | 1.0337 |
| FEV1 | Boys | 5.00 | 120 | BCPE | 2.56617 | 1.3672 | 0.12172 | 8.92253 | 1.3672 | 1.0527 |
| FEV1 | Boys | 5.00 | 121 | BCPE | 2.56617 | 1.3922 | 0.12172 | 8.92253 | 1.3922 | 1.0719 |
| FEV1 | Boys | 5.00 | 122 | BCPE | 2.56617 | 1.4173 | 0.12172 | 8.92253 | 1.4173 | 1.0912 |
| FEV1 | Boys | 5.00 | 123 | BCPE | 2.56617 | 1.4427 | 0.12172 | 8.92253 | 1.4427 | 1.1108 |
| FEV1 | Boys | 5.00 | 124 | BCPE | 2.56617 | 1.4684 | 0.12172 | 8.92253 | 1.4684 | 1.1306 |
| FEV1 | Boys | 5.00 | 125 | BCPE | 2.56617 | 1.4943 | 0.12172 | 8.92253 | 1.4943 | 1.1505 |
| FEV1 | Boys | 5.00 | 126 | BCPE | 2.56617 | 1.5204 | 0.12172 | 8.92253 | 1.5204 | 1.1706 |
| FEV1 | Boys | 5.00 | 127 | BCPE | 2.56617 | 1.5468 | 0.12172 | 8.92253 | 1.5468 | 1.1909 |
| FEV1 | Boys | 5.00 | 128 | BCPE | 2.56617 | 1.5735 | 0.12172 | 8.92253 | 1.5735 | 1.2115 |
| FEV1 | Boys | 5.00 | 129 | BCPE | 2.56617 | 1.6004 | 0.12172 | 8.92253 | 1.6004 | 1.2322 |
| FEV1 | Boys | 5.00 | 130 | BCPE | 2.56617 | 1.6275 | 0.12172 | 8.92253 | 1.6275 | 1.2530 |
| FEV1 | Boys | 5.00 | 131 | BCPE | 2.56617 | 1.6549 | 0.12172 | 8.92253 | 1.6549 | 1.2741 |
| FEV1 | Boys | 5.00 | 132 | BCPE | 2.56617 | 1.6825 | 0.12172 | 8.92253 | 1.6825 | 1.2954 |
| FEV1 | Boys | 5.25 | 79 | BCPE | 2.57248 | 0.5540 | 0.11951 | 8.79191 | 0.5540 | 0.4292 |
| FEV1 | Boys | 5.25 | 80 | BCPE | 2.57248 | 0.5693 | 0.11951 | 8.79191 | 0.5693 | 0.4411 |
| FEV1 | Boys | 5.25 | 81 | BCPE | 2.57248 | 0.5849 | 0.11951 | 8.79191 | 0.5849 | 0.4532 |
| FEV1 | Boys | 5.25 | 82 | BCPE | 2.57248 | 0.6008 | 0.11951 | 8.79191 | 0.6008 | 0.4655 |
| FEV1 | Boys | 5.25 | 83 | BCPE | 2.57248 | 0.6168 | 0.11951 | 8.79191 | 0.6168 | 0.4779 |
| FEV1 | Boys | 5.25 | 84 | BCPE | 2.57248 | 0.6331 | 0.11951 | 8.79191 | 0.6331 | 0.4906 |
| FEV1 | Boys | 5.25 | 85 | BCPE | 2.57248 | 0.6497 | 0.11951 | 8.79191 | 0.6497 | 0.5034 |
| FEV1 | Boys | 5.25 | 86 | BCPE | 2.57248 | 0.6664 | 0.11951 | 8.79191 | 0.6664 | 0.5164 |
| FEV1 | Boys | 5.25 | 87 | BCPE | 2.57248 | 0.6834 | 0.11951 | 8.79191 | 0.6834 | 0.5295 |
| FEV1 | Boys | 5.25 | 88 | BCPE | 2.57248 | 0.7006 | 0.11951 | 8.79191 | 0.7006 | 0.5429 |
| FEV1 | Boys | 5.25 | 89 | BCPE | 2.57248 | 0.7181 | 0.11951 | 8.79191 | 0.7181 | 0.5564 |
| FEV1 | Boys | 5.25 | 90 | BCPE | 2.57248 | 0.7358 | 0.11951 | 8.79191 | 0.7358 | 0.5701 |
| FEV1 | Boys | 5.25 | 91 | BCPE | 2.57248 | 0.7537 | 0.11951 | 8.79191 | 0.7537 | 0.5840 |
| FEV1 | Boys | 5.25 | 92 | BCPE | 2.57248 | 0.7718 | 0.11951 | 8.79191 | 0.7718 | 0.5980 |
| FEV1 | Boys | 5.25 | 93 | BCPE | 2.57248 | 0.7902 | 0.11951 | 8.79191 | 0.7902 | 0.6123 |
| FEV1 | Boys | 5.25 | 94 | BCPE | 2.57248 | 0.8088 | 0.11951 | 8.79191 | 0.8088 | 0.6267 |
| FEV1 | Boys | 5.25 | 95 | BCPE | 2.57248 | 0.8277 | 0.11951 | 8.79191 | 0.8277 | 0.6413 |
| FEV1 | Boys | 5.25 | 96 | BCPE | 2.57248 | 0.8467 | 0.11951 | 8.79191 | 0.8467 | 0.6561 |
| FEV1 | Boys | 5.25 | 97 | BCPE | 2.57248 | 0.8661 | 0.11951 | 8.79191 | 0.8661 | 0.6710 |
| FEV1 | Boys | 5.25 | 98 | BCPE | 2.57248 | 0.8856 | 0.11951 | 8.79191 | 0.8856 | 0.6862 |
| FEV1 | Boys | 5.25 | 99 | BCPE | 2.57248 | 0.9054 | 0.11951 | 8.79191 | 0.9054 | 0.7015 |
| FEV1 | Boys | 5.25 | 100 | BCPE | 2.57248 | 0.9254 | 0.11951 | 8.79191 | 0.9254 | 0.7170 |
| FEV1 | Boys | 5.25 | 101 | BCPE | 2.57248 | 0.9457 | 0.11951 | 8.79191 | 0.9457 | 0.7328 |
| FEV1 | Boys | 5.25 | 102 | BCPE | 2.57248 | 0.9662 | 0.11951 | 8.79191 | 0.9662 | 0.7486 |
| FEV1 | Boys | 5.25 | 103 | BCPE | 2.57248 | 0.9869 | 0.11951 | 8.79191 | 0.9869 | 0.7647 |
| FEV1 | Boys | 5.25 | 104 | BCPE | 2.57248 | 1.0079 | 0.11951 | 8.79191 | 1.0079 | 0.7810 |
| FEV1 | Boys | 5.25 | 105 | BCPE | 2.57248 | 1.0291 | 0.11951 | 8.79191 | 1.0291 | 0.7974 |
| FEV1 | Boys | 5.25 | 106 | BCPE | 2.57248 | 1.0506 | 0.11951 | 8.79191 | 1.0506 | 0.8140 |
| FEV1 | Boys | 5.25 | 107 | BCPE | 2.57248 | 1.0723 | 0.11951 | 8.79191 | 1.0723 | 0.8308 |
| FEV1 | Boys | 5.25 | 108 | BCPE | 2.57248 | 1.0942 | 0.11951 | 8.79191 | 1.0942 | 0.8478 |
| FEV1 | Boys | 5.25 | 109 | BCPE | 2.57248 | 1.1164 | 0.11951 | 8.79191 | 1.1164 | 0.8650 |
| FEV1 | Boys | 5.25 | 110 | BCPE | 2.57248 | 1.1388 | 0.11951 | 8.79191 | 1.1388 | 0.8824 |
| FEV1 | Boys | 5.25 | 111 | BCPE | 2.57248 | 1.1615 | 0.11951 | 8.79191 | 1.1615 | 0.8999 |
| FEV1 | Boys | 5.25 | 112 | BCPE | 2.57248 | 1.1844 | 0.11951 | 8.79191 | 1.1844 | 0.9177 |
| FEV1 | Boys | 5.25 | 113 | BCPE | 2.57248 | 1.2075 | 0.11951 | 8.79191 | 1.2075 | 0.9356 |
| FEV1 | Boys | 5.25 | 114 | BCPE | 2.57248 | 1.2309 | 0.11951 | 8.79191 | 1.2309 | 0.9537 |
| FEV1 | Boys | 5.25 | 115 | BCPE | 2.57248 | 1.2545 | 0.11951 | 8.79191 | 1.2545 | 0.9720 |
| FEV1 | Boys | 5.25 | 116 | BCPE | 2.57248 | 1.2784 | 0.11951 | 8.79191 | 1.2784 | 0.9905 |
| FEV1 | Boys | 5.25 | 117 | BCPE | 2.57248 | 1.3025 | 0.11951 | 8.79191 | 1.3025 | 1.0092 |
| FEV1 | Boys | 5.25 | 118 | BCPE | 2.57248 | 1.3269 | 0.11951 | 8.79191 | 1.3269 | 1.0281 |
| FEV1 | Boys | 5.25 | 119 | BCPE | 2.57248 | 1.3515 | 0.11951 | 8.79191 | 1.3515 | 1.0472 |
| FEV1 | Boys | 5.25 | 120 | BCPE | 2.57248 | 1.3763 | 0.11951 | 8.79191 | 1.3763 | 1.0664 |
| FEV1 | Boys | 5.25 | 121 | BCPE | 2.57248 | 1.4014 | 0.11951 | 8.79191 | 1.4014 | 1.0859 |
| FEV1 | Boys | 5.25 | 122 | BCPE | 2.57248 | 1.4268 | 0.11951 | 8.79191 | 1.4268 | 1.1055 |
| FEV1 | Boys | 5.25 | 123 | BCPE | 2.57248 | 1.4523 | 0.11951 | 8.79191 | 1.4523 | 1.1253 |
| FEV1 | Boys | 5.25 | 124 | BCPE | 2.57248 | 1.4782 | 0.11951 | 8.79191 | 1.4782 | 1.1453 |
| FEV1 | Boys | 5.25 | 125 | BCPE | 2.57248 | 1.5042 | 0.11951 | 8.79191 | 1.5042 | 1.1655 |
| FEV1 | Boys | 5.25 | 126 | BCPE | 2.57248 | 1.5306 | 0.11951 | 8.79191 | 1.5306 | 1.1859 |
| FEV1 | Boys | 5.25 | 127 | BCPE | 2.57248 | 1.5571 | 0.11951 | 8.79191 | 1.5571 | 1.2065 |
| FEV1 | Boys | 5.25 | 128 | BCPE | 2.57248 | 1.5839 | 0.11951 | 8.79191 | 1.5839 | 1.2273 |
| FEV1 | Boys | 5.25 | 129 | BCPE | 2.57248 | 1.6110 | 0.11951 | 8.79191 | 1.6110 | 1.2482 |
| FEV1 | Boys | 5.25 | 130 | BCPE | 2.57248 | 1.6383 | 0.11951 | 8.79191 | 1.6383 | 1.2694 |
| FEV1 | Boys | 5.25 | 131 | BCPE | 2.57248 | 1.6659 | 0.11951 | 8.79191 | 1.6659 | 1.2908 |
| FEV1 | Boys | 5.25 | 132 | BCPE | 2.57248 | 1.6937 | 0.11951 | 8.79191 | 1.6937 | 1.3123 |
| FEV1 | Boys | 5.50 | 79 | BCPE | 2.57849 | 0.5575 | 0.11726 | 8.66916 | 0.5575 | 0.4347 |
| FEV1 | Boys | 5.50 | 80 | BCPE | 2.57849 | 0.5729 | 0.11726 | 8.66916 | 0.5729 | 0.4468 |
| FEV1 | Boys | 5.50 | 81 | BCPE | 2.57849 | 0.5887 | 0.11726 | 8.66916 | 0.5887 | 0.4590 |
| FEV1 | Boys | 5.50 | 82 | BCPE | 2.57849 | 0.6046 | 0.11726 | 8.66916 | 0.6046 | 0.4715 |
| FEV1 | Boys | 5.50 | 83 | BCPE | 2.57849 | 0.6208 | 0.11726 | 8.66916 | 0.6208 | 0.4841 |
| FEV1 | Boys | 5.50 | 84 | BCPE | 2.57849 | 0.6372 | 0.11726 | 8.66916 | 0.6372 | 0.4969 |
| FEV1 | Boys | 5.50 | 85 | BCPE | 2.57849 | 0.6538 | 0.11726 | 8.66916 | 0.6538 | 0.5098 |
| FEV1 | Boys | 5.50 | 86 | BCPE | 2.57849 | 0.6706 | 0.11726 | 8.66916 | 0.6706 | 0.5230 |
| FEV1 | Boys | 5.50 | 87 | BCPE | 2.57849 | 0.6877 | 0.11726 | 8.66916 | 0.6877 | 0.5363 |
| FEV1 | Boys | 5.50 | 88 | BCPE | 2.57849 | 0.7051 | 0.11726 | 8.66916 | 0.7051 | 0.5498 |
| FEV1 | Boys | 5.50 | 89 | BCPE | 2.57849 | 0.7226 | 0.11726 | 8.66916 | 0.7226 | 0.5635 |
| FEV1 | Boys | 5.50 | 90 | BCPE | 2.57849 | 0.7404 | 0.11726 | 8.66916 | 0.7404 | 0.5774 |
| FEV1 | Boys | 5.50 | 91 | BCPE | 2.57849 | 0.7584 | 0.11726 | 8.66916 | 0.7584 | 0.5915 |
| FEV1 | Boys | 5.50 | 92 | BCPE | 2.57849 | 0.7767 | 0.11726 | 8.66916 | 0.7767 | 0.6057 |
| FEV1 | Boys | 5.50 | 93 | BCPE | 2.57849 | 0.7952 | 0.11726 | 8.66916 | 0.7952 | 0.6201 |
| FEV1 | Boys | 5.50 | 94 | BCPE | 2.57849 | 0.8139 | 0.11726 | 8.66916 | 0.8139 | 0.6347 |
| FEV1 | Boys | 5.50 | 95 | BCPE | 2.57849 | 0.8329 | 0.11726 | 8.66916 | 0.8329 | 0.6495 |
| FEV1 | Boys | 5.50 | 96 | BCPE | 2.57849 | 0.8521 | 0.11726 | 8.66916 | 0.8521 | 0.6645 |
| FEV1 | Boys | 5.50 | 97 | BCPE | 2.57849 | 0.8715 | 0.11726 | 8.66916 | 0.8715 | 0.6797 |
| FEV1 | Boys | 5.50 | 98 | BCPE | 2.57849 | 0.8912 | 0.11726 | 8.66916 | 0.8912 | 0.6950 |
| FEV1 | Boys | 5.50 | 99 | BCPE | 2.57849 | 0.9111 | 0.11726 | 8.66916 | 0.9111 | 0.7105 |
| FEV1 | Boys | 5.50 | 100 | BCPE | 2.57849 | 0.9313 | 0.11726 | 8.66916 | 0.9313 | 0.7262 |
| FEV1 | Boys | 5.50 | 101 | BCPE | 2.57849 | 0.9517 | 0.11726 | 8.66916 | 0.9517 | 0.7422 |
| FEV1 | Boys | 5.50 | 102 | BCPE | 2.57849 | 0.9723 | 0.11726 | 8.66916 | 0.9723 | 0.7582 |
| FEV1 | Boys | 5.50 | 103 | BCPE | 2.57849 | 0.9932 | 0.11726 | 8.66916 | 0.9932 | 0.7745 |
| FEV1 | Boys | 5.50 | 104 | BCPE | 2.57849 | 1.0143 | 0.11726 | 8.66916 | 1.0143 | 0.7910 |
| FEV1 | Boys | 5.50 | 105 | BCPE | 2.57849 | 1.0357 | 0.11726 | 8.66916 | 1.0357 | 0.8076 |
| FEV1 | Boys | 5.50 | 106 | BCPE | 2.57849 | 1.0572 | 0.11726 | 8.66916 | 1.0572 | 0.8245 |
| FEV1 | Boys | 5.50 | 107 | BCPE | 2.57849 | 1.0791 | 0.11726 | 8.66916 | 1.0791 | 0.8415 |
| FEV1 | Boys | 5.50 | 108 | BCPE | 2.57849 | 1.1012 | 0.11726 | 8.66916 | 1.1012 | 0.8587 |
| FEV1 | Boys | 5.50 | 109 | BCPE | 2.57849 | 1.1235 | 0.11726 | 8.66916 | 1.1235 | 0.8761 |
| FEV1 | Boys | 5.50 | 110 | BCPE | 2.57849 | 1.1460 | 0.11726 | 8.66916 | 1.1460 | 0.8937 |
| FEV1 | Boys | 5.50 | 111 | BCPE | 2.57849 | 1.1688 | 0.11726 | 8.66916 | 1.1688 | 0.9115 |
| FEV1 | Boys | 5.50 | 112 | BCPE | 2.57849 | 1.1919 | 0.11726 | 8.66916 | 1.1919 | 0.9295 |
| FEV1 | Boys | 5.50 | 113 | BCPE | 2.57849 | 1.2152 | 0.11726 | 8.66916 | 1.2152 | 0.9476 |
| FEV1 | Boys | 5.50 | 114 | BCPE | 2.57849 | 1.2387 | 0.11726 | 8.66916 | 1.2387 | 0.9660 |
| FEV1 | Boys | 5.50 | 115 | BCPE | 2.57849 | 1.2625 | 0.11726 | 8.66916 | 1.2625 | 0.9845 |
| FEV1 | Boys | 5.50 | 116 | BCPE | 2.57849 | 1.2865 | 0.11726 | 8.66916 | 1.2865 | 1.0033 |
| FEV1 | Boys | 5.50 | 117 | BCPE | 2.57849 | 1.3108 | 0.11726 | 8.66916 | 1.3108 | 1.0222 |
| FEV1 | Boys | 5.50 | 118 | BCPE | 2.57849 | 1.3353 | 0.11726 | 8.66916 | 1.3353 | 1.0413 |
| FEV1 | Boys | 5.50 | 119 | BCPE | 2.57849 | 1.3600 | 0.11726 | 8.66916 | 1.3600 | 1.0606 |
| FEV1 | Boys | 5.50 | 120 | BCPE | 2.57849 | 1.3850 | 0.11726 | 8.66916 | 1.3850 | 1.0801 |
| FEV1 | Boys | 5.50 | 121 | BCPE | 2.57849 | 1.4103 | 0.11726 | 8.66916 | 1.4103 | 1.0998 |
| FEV1 | Boys | 5.50 | 122 | BCPE | 2.57849 | 1.4358 | 0.11726 | 8.66916 | 1.4358 | 1.1197 |
| FEV1 | Boys | 5.50 | 123 | BCPE | 2.57849 | 1.4615 | 0.11726 | 8.66916 | 1.4615 | 1.1397 |
| FEV1 | Boys | 5.50 | 124 | BCPE | 2.57849 | 1.4875 | 0.11726 | 8.66916 | 1.4875 | 1.1600 |
| FEV1 | Boys | 5.50 | 125 | BCPE | 2.57849 | 1.5138 | 0.11726 | 8.66916 | 1.5138 | 1.1805 |
| FEV1 | Boys | 5.50 | 126 | BCPE | 2.57849 | 1.5402 | 0.11726 | 8.66916 | 1.5402 | 1.2011 |
| FEV1 | Boys | 5.50 | 127 | BCPE | 2.57849 | 1.5670 | 0.11726 | 8.66916 | 1.5670 | 1.2220 |
| FEV1 | Boys | 5.50 | 128 | BCPE | 2.57849 | 1.5940 | 0.11726 | 8.66916 | 1.5940 | 1.2430 |
| FEV1 | Boys | 5.50 | 129 | BCPE | 2.57849 | 1.6212 | 0.11726 | 8.66916 | 1.6212 | 1.2643 |
| FEV1 | Boys | 5.50 | 130 | BCPE | 2.57849 | 1.6487 | 0.11726 | 8.66916 | 1.6487 | 1.2857 |
| FEV1 | Boys | 5.50 | 131 | BCPE | 2.57849 | 1.6764 | 0.11726 | 8.66916 | 1.6764 | 1.3073 |
| FEV1 | Boys | 5.50 | 132 | BCPE | 2.57849 | 1.7044 | 0.11726 | 8.66916 | 1.7044 | 1.3291 |
| FEV1 | Boys | 5.75 | 79 | BCPE | 2.58424 | 0.5608 | 0.11504 | 8.55347 | 0.5608 | 0.4401 |
| FEV1 | Boys | 5.75 | 80 | BCPE | 2.58424 | 0.5764 | 0.11504 | 8.55347 | 0.5764 | 0.4523 |
| FEV1 | Boys | 5.75 | 81 | BCPE | 2.58424 | 0.5922 | 0.11504 | 8.55347 | 0.5922 | 0.4647 |
| FEV1 | Boys | 5.75 | 82 | BCPE | 2.58424 | 0.6082 | 0.11504 | 8.55347 | 0.6082 | 0.4773 |
| FEV1 | Boys | 5.75 | 83 | BCPE | 2.58424 | 0.6245 | 0.11504 | 8.55347 | 0.6245 | 0.4901 |
| FEV1 | Boys | 5.75 | 84 | BCPE | 2.58424 | 0.6410 | 0.11504 | 8.55347 | 0.6410 | 0.5030 |
| FEV1 | Boys | 5.75 | 85 | BCPE | 2.58424 | 0.6577 | 0.11504 | 8.55347 | 0.6577 | 0.5161 |
| FEV1 | Boys | 5.75 | 86 | BCPE | 2.58424 | 0.6747 | 0.11504 | 8.55347 | 0.6747 | 0.5294 |
| FEV1 | Boys | 5.75 | 87 | BCPE | 2.58424 | 0.6919 | 0.11504 | 8.55347 | 0.6919 | 0.5429 |
| FEV1 | Boys | 5.75 | 88 | BCPE | 2.58424 | 0.7093 | 0.11504 | 8.55347 | 0.7093 | 0.5566 |
| FEV1 | Boys | 5.75 | 89 | BCPE | 2.58424 | 0.7270 | 0.11504 | 8.55347 | 0.7270 | 0.5705 |
| FEV1 | Boys | 5.75 | 90 | BCPE | 2.58424 | 0.7449 | 0.11504 | 8.55347 | 0.7449 | 0.5845 |
| FEV1 | Boys | 5.75 | 91 | BCPE | 2.58424 | 0.7630 | 0.11504 | 8.55347 | 0.7630 | 0.5987 |
| FEV1 | Boys | 5.75 | 92 | BCPE | 2.58424 | 0.7814 | 0.11504 | 8.55347 | 0.7814 | 0.6132 |
| FEV1 | Boys | 5.75 | 93 | BCPE | 2.58424 | 0.8000 | 0.11504 | 8.55347 | 0.8000 | 0.6278 |
| FEV1 | Boys | 5.75 | 94 | BCPE | 2.58424 | 0.8188 | 0.11504 | 8.55347 | 0.8188 | 0.6426 |
| FEV1 | Boys | 5.75 | 95 | BCPE | 2.58424 | 0.8379 | 0.11504 | 8.55347 | 0.8379 | 0.6575 |
| FEV1 | Boys | 5.75 | 96 | BCPE | 2.58424 | 0.8572 | 0.11504 | 8.55347 | 0.8572 | 0.6727 |
| FEV1 | Boys | 5.75 | 97 | BCPE | 2.58424 | 0.8768 | 0.11504 | 8.55347 | 0.8768 | 0.6880 |
| FEV1 | Boys | 5.75 | 98 | BCPE | 2.58424 | 0.8966 | 0.11504 | 8.55347 | 0.8966 | 0.7036 |
| FEV1 | Boys | 5.75 | 99 | BCPE | 2.58424 | 0.9166 | 0.11504 | 8.55347 | 0.9166 | 0.7193 |
| FEV1 | Boys | 5.75 | 100 | BCPE | 2.58424 | 0.9369 | 0.11504 | 8.55347 | 0.9369 | 0.7352 |
| FEV1 | Boys | 5.75 | 101 | BCPE | 2.58424 | 0.9574 | 0.11504 | 8.55347 | 0.9574 | 0.7513 |
| FEV1 | Boys | 5.75 | 102 | BCPE | 2.58424 | 0.9782 | 0.11504 | 8.55347 | 0.9782 | 0.7676 |
| FEV1 | Boys | 5.75 | 103 | BCPE | 2.58424 | 0.9992 | 0.11504 | 8.55347 | 0.9992 | 0.7841 |
| FEV1 | Boys | 5.75 | 104 | BCPE | 2.58424 | 1.0204 | 0.11504 | 8.55347 | 1.0204 | 0.8007 |
| FEV1 | Boys | 5.75 | 105 | BCPE | 2.58424 | 1.0419 | 0.11504 | 8.55347 | 1.0419 | 0.8176 |
| FEV1 | Boys | 5.75 | 106 | BCPE | 2.58424 | 1.0636 | 0.11504 | 8.55347 | 1.0636 | 0.8346 |
| FEV1 | Boys | 5.75 | 107 | BCPE | 2.58424 | 1.0856 | 0.11504 | 8.55347 | 1.0856 | 0.8519 |
| FEV1 | Boys | 5.75 | 108 | BCPE | 2.58424 | 1.1078 | 0.11504 | 8.55347 | 1.1078 | 0.8693 |
| FEV1 | Boys | 5.75 | 109 | BCPE | 2.58424 | 1.1303 | 0.11504 | 8.55347 | 1.1303 | 0.8869 |
| FEV1 | Boys | 5.75 | 110 | BCPE | 2.58424 | 1.1530 | 0.11504 | 8.55347 | 1.1530 | 0.9047 |
| FEV1 | Boys | 5.75 | 111 | BCPE | 2.58424 | 1.1759 | 0.11504 | 8.55347 | 1.1759 | 0.9227 |
| FEV1 | Boys | 5.75 | 112 | BCPE | 2.58424 | 1.1991 | 0.11504 | 8.55347 | 1.1991 | 0.9409 |
| FEV1 | Boys | 5.75 | 113 | BCPE | 2.58424 | 1.2225 | 0.11504 | 8.55347 | 1.2225 | 0.9593 |
| FEV1 | Boys | 5.75 | 114 | BCPE | 2.58424 | 1.2462 | 0.11504 | 8.55347 | 1.2462 | 0.9779 |
| FEV1 | Boys | 5.75 | 115 | BCPE | 2.58424 | 1.2701 | 0.11504 | 8.55347 | 1.2701 | 0.9967 |
| FEV1 | Boys | 5.75 | 116 | BCPE | 2.58424 | 1.2943 | 0.11504 | 8.55347 | 1.2943 | 1.0156 |
| FEV1 | Boys | 5.75 | 117 | BCPE | 2.58424 | 1.3187 | 0.11504 | 8.55347 | 1.3187 | 1.0348 |
| FEV1 | Boys | 5.75 | 118 | BCPE | 2.58424 | 1.3433 | 0.11504 | 8.55347 | 1.3433 | 1.0541 |
| FEV1 | Boys | 5.75 | 119 | BCPE | 2.58424 | 1.3683 | 0.11504 | 8.55347 | 1.3683 | 1.0737 |
| FEV1 | Boys | 5.75 | 120 | BCPE | 2.58424 | 1.3934 | 0.11504 | 8.55347 | 1.3934 | 1.0934 |
| FEV1 | Boys | 5.75 | 121 | BCPE | 2.58424 | 1.4188 | 0.11504 | 8.55347 | 1.4188 | 1.1134 |
| FEV1 | Boys | 5.75 | 122 | BCPE | 2.58424 | 1.4445 | 0.11504 | 8.55347 | 1.4445 | 1.1335 |
| FEV1 | Boys | 5.75 | 123 | BCPE | 2.58424 | 1.4704 | 0.11504 | 8.55347 | 1.4704 | 1.1538 |
| FEV1 | Boys | 5.75 | 124 | BCPE | 2.58424 | 1.4965 | 0.11504 | 8.55347 | 1.4965 | 1.1743 |
| FEV1 | Boys | 5.75 | 125 | BCPE | 2.58424 | 1.5229 | 0.11504 | 8.55347 | 1.5229 | 1.1950 |
| FEV1 | Boys | 5.75 | 126 | BCPE | 2.58424 | 1.5496 | 0.11504 | 8.55347 | 1.5496 | 1.2159 |
| FEV1 | Boys | 5.75 | 127 | BCPE | 2.58424 | 1.5765 | 0.11504 | 8.55347 | 1.5765 | 1.2371 |
| FEV1 | Boys | 5.75 | 128 | BCPE | 2.58424 | 1.6036 | 0.11504 | 8.55347 | 1.6036 | 1.2584 |
| FEV1 | Boys | 5.75 | 129 | BCPE | 2.58424 | 1.6310 | 0.11504 | 8.55347 | 1.6310 | 1.2799 |
| FEV1 | Boys | 5.75 | 130 | BCPE | 2.58424 | 1.6587 | 0.11504 | 8.55347 | 1.6587 | 1.3016 |
| FEV1 | Boys | 5.75 | 131 | BCPE | 2.58424 | 1.6866 | 0.11504 | 8.55347 | 1.6866 | 1.3235 |
| FEV1 | Boys | 5.75 | 132 | BCPE | 2.58424 | 1.7147 | 0.11504 | 8.55347 | 1.7147 | 1.3455 |
| FEV1 | Boys | 6.00 | 79 | BCPE | 2.58975 | 0.5641 | 0.11293 | 8.44414 | 0.5641 | 0.4453 |
| FEV1 | Boys | 6.00 | 80 | BCPE | 2.58975 | 0.5797 | 0.11293 | 8.44414 | 0.5797 | 0.4576 |
| FEV1 | Boys | 6.00 | 81 | BCPE | 2.58975 | 0.5956 | 0.11293 | 8.44414 | 0.5956 | 0.4702 |
| FEV1 | Boys | 6.00 | 82 | BCPE | 2.58975 | 0.6118 | 0.11293 | 8.44414 | 0.6118 | 0.4829 |
| FEV1 | Boys | 6.00 | 83 | BCPE | 2.58975 | 0.6281 | 0.11293 | 8.44414 | 0.6281 | 0.4958 |
| FEV1 | Boys | 6.00 | 84 | BCPE | 2.58975 | 0.6447 | 0.11293 | 8.44414 | 0.6447 | 0.5089 |
| FEV1 | Boys | 6.00 | 85 | BCPE | 2.58975 | 0.6615 | 0.11293 | 8.44414 | 0.6615 | 0.5222 |
| FEV1 | Boys | 6.00 | 86 | BCPE | 2.58975 | 0.6786 | 0.11293 | 8.44414 | 0.6786 | 0.5356 |
| FEV1 | Boys | 6.00 | 87 | BCPE | 2.58975 | 0.6959 | 0.11293 | 8.44414 | 0.6959 | 0.5493 |
| FEV1 | Boys | 6.00 | 88 | BCPE | 2.58975 | 0.7134 | 0.11293 | 8.44414 | 0.7134 | 0.5631 |
| FEV1 | Boys | 6.00 | 89 | BCPE | 2.58975 | 0.7312 | 0.11293 | 8.44414 | 0.7312 | 0.5772 |
| FEV1 | Boys | 6.00 | 90 | BCPE | 2.58975 | 0.7492 | 0.11293 | 8.44414 | 0.7492 | 0.5914 |
| FEV1 | Boys | 6.00 | 91 | BCPE | 2.58975 | 0.7674 | 0.11293 | 8.44414 | 0.7674 | 0.6058 |
| FEV1 | Boys | 6.00 | 92 | BCPE | 2.58975 | 0.7859 | 0.11293 | 8.44414 | 0.7859 | 0.6203 |
| FEV1 | Boys | 6.00 | 93 | BCPE | 2.58975 | 0.8046 | 0.11293 | 8.44414 | 0.8046 | 0.6351 |
| FEV1 | Boys | 6.00 | 94 | BCPE | 2.58975 | 0.8236 | 0.11293 | 8.44414 | 0.8236 | 0.6501 |
| FEV1 | Boys | 6.00 | 95 | BCPE | 2.58975 | 0.8428 | 0.11293 | 8.44414 | 0.8428 | 0.6652 |
| FEV1 | Boys | 6.00 | 96 | BCPE | 2.58975 | 0.8622 | 0.11293 | 8.44414 | 0.8622 | 0.6806 |
| FEV1 | Boys | 6.00 | 97 | BCPE | 2.58975 | 0.8819 | 0.11293 | 8.44414 | 0.8819 | 0.6961 |
| FEV1 | Boys | 6.00 | 98 | BCPE | 2.58975 | 0.9018 | 0.11293 | 8.44414 | 0.9018 | 0.7118 |
| FEV1 | Boys | 6.00 | 99 | BCPE | 2.58975 | 0.9219 | 0.11293 | 8.44414 | 0.9219 | 0.7277 |
| FEV1 | Boys | 6.00 | 100 | BCPE | 2.58975 | 0.9423 | 0.11293 | 8.44414 | 0.9423 | 0.7438 |
| FEV1 | Boys | 6.00 | 101 | BCPE | 2.58975 | 0.9630 | 0.11293 | 8.44414 | 0.9630 | 0.7601 |
| FEV1 | Boys | 6.00 | 102 | BCPE | 2.58975 | 0.9838 | 0.11293 | 8.44414 | 0.9838 | 0.7766 |
| FEV1 | Boys | 6.00 | 103 | BCPE | 2.58975 | 1.0050 | 0.11293 | 8.44414 | 1.0050 | 0.7933 |
| FEV1 | Boys | 6.00 | 104 | BCPE | 2.58975 | 1.0263 | 0.11293 | 8.44414 | 1.0263 | 0.8101 |
| FEV1 | Boys | 6.00 | 105 | BCPE | 2.58975 | 1.0479 | 0.11293 | 8.44414 | 1.0479 | 0.8272 |
| FEV1 | Boys | 6.00 | 106 | BCPE | 2.58975 | 1.0698 | 0.11293 | 8.44414 | 1.0698 | 0.8444 |
| FEV1 | Boys | 6.00 | 107 | BCPE | 2.58975 | 1.0919 | 0.11293 | 8.44414 | 1.0919 | 0.8619 |
| FEV1 | Boys | 6.00 | 108 | BCPE | 2.58975 | 1.1142 | 0.11293 | 8.44414 | 1.1142 | 0.8795 |
| FEV1 | Boys | 6.00 | 109 | BCPE | 2.58975 | 1.1368 | 0.11293 | 8.44414 | 1.1368 | 0.8973 |
| FEV1 | Boys | 6.00 | 110 | BCPE | 2.58975 | 1.1596 | 0.11293 | 8.44414 | 1.1596 | 0.9153 |
| FEV1 | Boys | 6.00 | 111 | BCPE | 2.58975 | 1.1827 | 0.11293 | 8.44414 | 1.1827 | 0.9336 |
| FEV1 | Boys | 6.00 | 112 | BCPE | 2.58975 | 1.2060 | 0.11293 | 8.44414 | 1.2060 | 0.9520 |
| FEV1 | Boys | 6.00 | 113 | BCPE | 2.58975 | 1.2296 | 0.11293 | 8.44414 | 1.2296 | 0.9706 |
| FEV1 | Boys | 6.00 | 114 | BCPE | 2.58975 | 1.2534 | 0.11293 | 8.44414 | 1.2534 | 0.9894 |
| FEV1 | Boys | 6.00 | 115 | BCPE | 2.58975 | 1.2775 | 0.11293 | 8.44414 | 1.2775 | 1.0083 |
| FEV1 | Boys | 6.00 | 116 | BCPE | 2.58975 | 1.3018 | 0.11293 | 8.44414 | 1.3018 | 1.0275 |
| FEV1 | Boys | 6.00 | 117 | BCPE | 2.58975 | 1.3263 | 0.11293 | 8.44414 | 1.3263 | 1.0469 |
| FEV1 | Boys | 6.00 | 118 | BCPE | 2.58975 | 1.3511 | 0.11293 | 8.44414 | 1.3511 | 1.0665 |
| FEV1 | Boys | 6.00 | 119 | BCPE | 2.58975 | 1.3762 | 0.11293 | 8.44414 | 1.3762 | 1.0863 |
| FEV1 | Boys | 6.00 | 120 | BCPE | 2.58975 | 1.4015 | 0.11293 | 8.44414 | 1.4015 | 1.1062 |
| FEV1 | Boys | 6.00 | 121 | BCPE | 2.58975 | 1.4270 | 0.11293 | 8.44414 | 1.4270 | 1.1264 |
| FEV1 | Boys | 6.00 | 122 | BCPE | 2.58975 | 1.4528 | 0.11293 | 8.44414 | 1.4528 | 1.1468 |
| FEV1 | Boys | 6.00 | 123 | BCPE | 2.58975 | 1.4789 | 0.11293 | 8.44414 | 1.4789 | 1.1673 |
| FEV1 | Boys | 6.00 | 124 | BCPE | 2.58975 | 1.5052 | 0.11293 | 8.44414 | 1.5052 | 1.1881 |
| FEV1 | Boys | 6.00 | 125 | BCPE | 2.58975 | 1.5317 | 0.11293 | 8.44414 | 1.5317 | 1.2090 |
| FEV1 | Boys | 6.00 | 126 | BCPE | 2.58975 | 1.5585 | 0.11293 | 8.44414 | 1.5585 | 1.2302 |
| FEV1 | Boys | 6.00 | 127 | BCPE | 2.58975 | 1.5856 | 0.11293 | 8.44414 | 1.5856 | 1.2516 |
| FEV1 | Boys | 6.00 | 128 | BCPE | 2.58975 | 1.6129 | 0.11293 | 8.44414 | 1.6129 | 1.2731 |
| FEV1 | Boys | 6.00 | 129 | BCPE | 2.58975 | 1.6404 | 0.11293 | 8.44414 | 1.6404 | 1.2949 |
| FEV1 | Boys | 6.00 | 130 | BCPE | 2.58975 | 1.6682 | 0.11293 | 8.44414 | 1.6682 | 1.3168 |
| FEV1 | Boys | 6.00 | 131 | BCPE | 2.58975 | 1.6963 | 0.11293 | 8.44414 | 1.6963 | 1.3390 |
| FEV1 | Boys | 6.00 | 132 | BCPE | 2.58975 | 1.7246 | 0.11293 | 8.44414 | 1.7246 | 1.3613 |
| FEV1 | Girls | 3.00 | 88 | BCPE | 2.55115 | 0.6487 | 0.11409 | 6.59226 | 0.6487 | 0.5101 |
| FEV1 | Girls | 3.00 | 89 | BCPE | 2.55115 | 0.6634 | 0.11409 | 6.59226 | 0.6634 | 0.5217 |
| FEV1 | Girls | 3.00 | 90 | BCPE | 2.55115 | 0.6783 | 0.11409 | 6.59226 | 0.6783 | 0.5334 |
| FEV1 | Girls | 3.00 | 91 | BCPE | 2.55115 | 0.6934 | 0.11409 | 6.59226 | 0.6934 | 0.5452 |
| FEV1 | Girls | 3.00 | 92 | BCPE | 2.55115 | 0.7086 | 0.11409 | 6.59226 | 0.7086 | 0.5572 |
| FEV1 | Girls | 3.00 | 93 | BCPE | 2.55115 | 0.7240 | 0.11409 | 6.59226 | 0.7240 | 0.5693 |
| FEV1 | Girls | 3.00 | 94 | BCPE | 2.55115 | 0.7395 | 0.11409 | 6.59226 | 0.7395 | 0.5815 |
| FEV1 | Girls | 3.00 | 95 | BCPE | 2.55115 | 0.7552 | 0.11409 | 6.59226 | 0.7552 | 0.5939 |
| FEV1 | Girls | 3.00 | 96 | BCPE | 2.55115 | 0.7711 | 0.11409 | 6.59226 | 0.7711 | 0.6063 |
| FEV1 | Girls | 3.00 | 97 | BCPE | 2.55115 | 0.7871 | 0.11409 | 6.59226 | 0.7871 | 0.6190 |
| FEV1 | Girls | 3.00 | 98 | BCPE | 2.55115 | 0.8033 | 0.11409 | 6.59226 | 0.8033 | 0.6317 |
| FEV1 | Girls | 3.00 | 99 | BCPE | 2.55115 | 0.8197 | 0.11409 | 6.59226 | 0.8197 | 0.6446 |
| FEV1 | Girls | 3.00 | 100 | BCPE | 2.55115 | 0.8362 | 0.11409 | 6.59226 | 0.8362 | 0.6575 |
| FEV1 | Girls | 3.00 | 101 | BCPE | 2.55115 | 0.8529 | 0.11409 | 6.59226 | 0.8529 | 0.6707 |
| FEV1 | Girls | 3.00 | 102 | BCPE | 2.55115 | 0.8697 | 0.11409 | 6.59226 | 0.8697 | 0.6839 |
| FEV1 | Girls | 3.00 | 103 | BCPE | 2.55115 | 0.8868 | 0.11409 | 6.59226 | 0.8868 | 0.6973 |
| FEV1 | Girls | 3.00 | 104 | BCPE | 2.55115 | 0.9039 | 0.11409 | 6.59226 | 0.9039 | 0.7108 |
| FEV1 | Girls | 3.00 | 105 | BCPE | 2.55115 | 0.9213 | 0.11409 | 6.59226 | 0.9213 | 0.7245 |
| FEV1 | Girls | 3.00 | 106 | BCPE | 2.55115 | 0.9388 | 0.11409 | 6.59226 | 0.9388 | 0.7382 |
| FEV1 | Girls | 3.00 | 107 | BCPE | 2.55115 | 0.9565 | 0.11409 | 6.59226 | 0.9565 | 0.7521 |
| FEV1 | Girls | 3.00 | 108 | BCPE | 2.55115 | 0.9743 | 0.11409 | 6.59226 | 0.9743 | 0.7661 |
| FEV1 | Girls | 3.00 | 109 | BCPE | 2.55115 | 0.9923 | 0.11409 | 6.59226 | 0.9923 | 0.7803 |
| FEV1 | Girls | 3.00 | 110 | BCPE | 2.55115 | 1.0105 | 0.11409 | 6.59226 | 1.0105 | 0.7946 |
| FEV1 | Girls | 3.00 | 111 | BCPE | 2.55115 | 1.0288 | 0.11409 | 6.59226 | 1.0288 | 0.8090 |
| FEV1 | Girls | 3.00 | 112 | BCPE | 2.55115 | 1.0473 | 0.11409 | 6.59226 | 1.0473 | 0.8235 |
| FEV1 | Girls | 3.00 | 113 | BCPE | 2.55115 | 1.0659 | 0.11409 | 6.59226 | 1.0659 | 0.8382 |
| FEV1 | Girls | 3.00 | 114 | BCPE | 2.55115 | 1.0847 | 0.11409 | 6.59226 | 1.0847 | 0.8530 |
| FEV1 | Girls | 3.00 | 115 | BCPE | 2.55115 | 1.1037 | 0.11409 | 6.59226 | 1.1037 | 0.8679 |
| FEV1 | Girls | 3.00 | 116 | BCPE | 2.55115 | 1.1229 | 0.11409 | 6.59226 | 1.1229 | 0.8830 |
| FEV1 | Girls | 3.00 | 117 | BCPE | 2.55115 | 1.1422 | 0.11409 | 6.59226 | 1.1422 | 0.8981 |
| FEV1 | Girls | 3.00 | 118 | BCPE | 2.55115 | 1.1616 | 0.11409 | 6.59226 | 1.1616 | 0.9134 |
| FEV1 | Girls | 3.00 | 119 | BCPE | 2.55115 | 1.1813 | 0.11409 | 6.59226 | 1.1813 | 0.9289 |
| FEV1 | Girls | 3.00 | 120 | BCPE | 2.55115 | 1.2011 | 0.11409 | 6.59226 | 1.2011 | 0.9444 |
| FEV1 | Girls | 3.00 | 121 | BCPE | 2.55115 | 1.2210 | 0.11409 | 6.59226 | 1.2210 | 0.9601 |
| FEV1 | Girls | 3.00 | 122 | BCPE | 2.55115 | 1.2411 | 0.11409 | 6.59226 | 1.2411 | 0.9760 |
| FEV1 | Girls | 3.00 | 123 | BCPE | 2.55115 | 1.2614 | 0.11409 | 6.59226 | 1.2614 | 0.9919 |
| FEV1 | Girls | 3.00 | 124 | BCPE | 2.55115 | 1.2819 | 0.11409 | 6.59226 | 1.2819 | 1.0080 |
| FEV1 | Girls | 3.25 | 88 | BCPE | 2.57483 | 0.6566 | 0.11433 | 6.60844 | 0.6566 | 0.5155 |
| FEV1 | Girls | 3.25 | 89 | BCPE | 2.57483 | 0.6715 | 0.11433 | 6.60844 | 0.6715 | 0.5272 |
| FEV1 | Girls | 3.25 | 90 | BCPE | 2.57483 | 0.6865 | 0.11433 | 6.60844 | 0.6865 | 0.5390 |
| FEV1 | Girls | 3.25 | 91 | BCPE | 2.57483 | 0.7018 | 0.11433 | 6.60844 | 0.7018 | 0.5510 |
| FEV1 | Girls | 3.25 | 92 | BCPE | 2.57483 | 0.7172 | 0.11433 | 6.60844 | 0.7172 | 0.5631 |
| FEV1 | Girls | 3.25 | 93 | BCPE | 2.57483 | 0.7327 | 0.11433 | 6.60844 | 0.7327 | 0.5753 |
| FEV1 | Girls | 3.25 | 94 | BCPE | 2.57483 | 0.7485 | 0.11433 | 6.60844 | 0.7485 | 0.5877 |
| FEV1 | Girls | 3.25 | 95 | BCPE | 2.57483 | 0.7644 | 0.11433 | 6.60844 | 0.7644 | 0.6001 |
| FEV1 | Girls | 3.25 | 96 | BCPE | 2.57483 | 0.7804 | 0.11433 | 6.60844 | 0.7804 | 0.6127 |
| FEV1 | Girls | 3.25 | 97 | BCPE | 2.57483 | 0.7967 | 0.11433 | 6.60844 | 0.7967 | 0.6255 |
| FEV1 | Girls | 3.25 | 98 | BCPE | 2.57483 | 0.8131 | 0.11433 | 6.60844 | 0.8131 | 0.6384 |
| FEV1 | Girls | 3.25 | 99 | BCPE | 2.57483 | 0.8296 | 0.11433 | 6.60844 | 0.8296 | 0.6514 |
| FEV1 | Girls | 3.25 | 100 | BCPE | 2.57483 | 0.8463 | 0.11433 | 6.60844 | 0.8463 | 0.6645 |
| FEV1 | Girls | 3.25 | 101 | BCPE | 2.57483 | 0.8632 | 0.11433 | 6.60844 | 0.8632 | 0.6778 |
| FEV1 | Girls | 3.25 | 102 | BCPE | 2.57483 | 0.8803 | 0.11433 | 6.60844 | 0.8803 | 0.6911 |
| FEV1 | Girls | 3.25 | 103 | BCPE | 2.57483 | 0.8975 | 0.11433 | 6.60844 | 0.8975 | 0.7047 |
| FEV1 | Girls | 3.25 | 104 | BCPE | 2.57483 | 0.9149 | 0.11433 | 6.60844 | 0.9149 | 0.7183 |
| FEV1 | Girls | 3.25 | 105 | BCPE | 2.57483 | 0.9324 | 0.11433 | 6.60844 | 0.9324 | 0.7321 |
| FEV1 | Girls | 3.25 | 106 | BCPE | 2.57483 | 0.9502 | 0.11433 | 6.60844 | 0.9502 | 0.7460 |
| FEV1 | Girls | 3.25 | 107 | BCPE | 2.57483 | 0.9680 | 0.11433 | 6.60844 | 0.9680 | 0.7601 |
| FEV1 | Girls | 3.25 | 108 | BCPE | 2.57483 | 0.9861 | 0.11433 | 6.60844 | 0.9861 | 0.7742 |
| FEV1 | Girls | 3.25 | 109 | BCPE | 2.57483 | 1.0043 | 0.11433 | 6.60844 | 1.0043 | 0.7885 |
| FEV1 | Girls | 3.25 | 110 | BCPE | 2.57483 | 1.0227 | 0.11433 | 6.60844 | 1.0227 | 0.8030 |
| FEV1 | Girls | 3.25 | 111 | BCPE | 2.57483 | 1.0412 | 0.11433 | 6.60844 | 1.0412 | 0.8175 |
| FEV1 | Girls | 3.25 | 112 | BCPE | 2.57483 | 1.0600 | 0.11433 | 6.60844 | 1.0600 | 0.8322 |
| FEV1 | Girls | 3.25 | 113 | BCPE | 2.57483 | 1.0788 | 0.11433 | 6.60844 | 1.0788 | 0.8470 |
| FEV1 | Girls | 3.25 | 114 | BCPE | 2.57483 | 1.0979 | 0.11433 | 6.60844 | 1.0979 | 0.8620 |
| FEV1 | Girls | 3.25 | 115 | BCPE | 2.57483 | 1.1171 | 0.11433 | 6.60844 | 1.1171 | 0.8771 |
| FEV1 | Girls | 3.25 | 116 | BCPE | 2.57483 | 1.1365 | 0.11433 | 6.60844 | 1.1365 | 0.8923 |
| FEV1 | Girls | 3.25 | 117 | BCPE | 2.57483 | 1.1560 | 0.11433 | 6.60844 | 1.1560 | 0.9076 |
| FEV1 | Girls | 3.25 | 118 | BCPE | 2.57483 | 1.1757 | 0.11433 | 6.60844 | 1.1757 | 0.9231 |
| FEV1 | Girls | 3.25 | 119 | BCPE | 2.57483 | 1.1956 | 0.11433 | 6.60844 | 1.1956 | 0.9387 |
| FEV1 | Girls | 3.25 | 120 | BCPE | 2.57483 | 1.2156 | 0.11433 | 6.60844 | 1.2156 | 0.9544 |
| FEV1 | Girls | 3.25 | 121 | BCPE | 2.57483 | 1.2358 | 0.11433 | 6.60844 | 1.2358 | 0.9703 |
| FEV1 | Girls | 3.25 | 122 | BCPE | 2.57483 | 1.2562 | 0.11433 | 6.60844 | 1.2562 | 0.9863 |
| FEV1 | Girls | 3.25 | 123 | BCPE | 2.57483 | 1.2767 | 0.11433 | 6.60844 | 1.2767 | 1.0024 |
| FEV1 | Girls | 3.25 | 124 | BCPE | 2.57483 | 1.2974 | 0.11433 | 6.60844 | 1.2974 | 1.0186 |
| FEV1 | Girls | 3.50 | 88 | BCPE | 2.59675 | 0.6640 | 0.11455 | 6.62345 | 0.6640 | 0.5206 |
| FEV1 | Girls | 3.50 | 89 | BCPE | 2.59675 | 0.6790 | 0.11455 | 6.62345 | 0.6790 | 0.5324 |
| FEV1 | Girls | 3.50 | 90 | BCPE | 2.59675 | 0.6943 | 0.11455 | 6.62345 | 0.6943 | 0.5443 |
| FEV1 | Girls | 3.50 | 91 | BCPE | 2.59675 | 0.7097 | 0.11455 | 6.62345 | 0.7097 | 0.5564 |
| FEV1 | Girls | 3.50 | 92 | BCPE | 2.59675 | 0.7252 | 0.11455 | 6.62345 | 0.7252 | 0.5686 |
| FEV1 | Girls | 3.50 | 93 | BCPE | 2.59675 | 0.7410 | 0.11455 | 6.62345 | 0.7410 | 0.5809 |
| FEV1 | Girls | 3.50 | 94 | BCPE | 2.59675 | 0.7569 | 0.11455 | 6.62345 | 0.7569 | 0.5934 |
| FEV1 | Girls | 3.50 | 95 | BCPE | 2.59675 | 0.7730 | 0.11455 | 6.62345 | 0.7730 | 0.6060 |
| FEV1 | Girls | 3.50 | 96 | BCPE | 2.59675 | 0.7892 | 0.11455 | 6.62345 | 0.7892 | 0.6187 |
| FEV1 | Girls | 3.50 | 97 | BCPE | 2.59675 | 0.8056 | 0.11455 | 6.62345 | 0.8056 | 0.6316 |
| FEV1 | Girls | 3.50 | 98 | BCPE | 2.59675 | 0.8222 | 0.11455 | 6.62345 | 0.8222 | 0.6446 |
| FEV1 | Girls | 3.50 | 99 | BCPE | 2.59675 | 0.8389 | 0.11455 | 6.62345 | 0.8389 | 0.6577 |
| FEV1 | Girls | 3.50 | 100 | BCPE | 2.59675 | 0.8558 | 0.11455 | 6.62345 | 0.8558 | 0.6710 |
| FEV1 | Girls | 3.50 | 101 | BCPE | 2.59675 | 0.8729 | 0.11455 | 6.62345 | 0.8729 | 0.6844 |
| FEV1 | Girls | 3.50 | 102 | BCPE | 2.59675 | 0.8902 | 0.11455 | 6.62345 | 0.8902 | 0.6979 |
| FEV1 | Girls | 3.50 | 103 | BCPE | 2.59675 | 0.9076 | 0.11455 | 6.62345 | 0.9076 | 0.7116 |
| FEV1 | Girls | 3.50 | 104 | BCPE | 2.59675 | 0.9252 | 0.11455 | 6.62345 | 0.9252 | 0.7253 |
| FEV1 | Girls | 3.50 | 105 | BCPE | 2.59675 | 0.9429 | 0.11455 | 6.62345 | 0.9429 | 0.7393 |
| FEV1 | Girls | 3.50 | 106 | BCPE | 2.59675 | 0.9608 | 0.11455 | 6.62345 | 0.9608 | 0.7533 |
| FEV1 | Girls | 3.50 | 107 | BCPE | 2.59675 | 0.9789 | 0.11455 | 6.62345 | 0.9789 | 0.7675 |
| FEV1 | Girls | 3.50 | 108 | BCPE | 2.59675 | 0.9972 | 0.11455 | 6.62345 | 0.9972 | 0.7818 |
| FEV1 | Girls | 3.50 | 109 | BCPE | 2.59675 | 1.0156 | 0.11455 | 6.62345 | 1.0156 | 0.7962 |
| FEV1 | Girls | 3.50 | 110 | BCPE | 2.59675 | 1.0342 | 0.11455 | 6.62345 | 1.0342 | 0.8108 |
| FEV1 | Girls | 3.50 | 111 | BCPE | 2.59675 | 1.0529 | 0.11455 | 6.62345 | 1.0529 | 0.8255 |
| FEV1 | Girls | 3.50 | 112 | BCPE | 2.59675 | 1.0719 | 0.11455 | 6.62345 | 1.0719 | 0.8404 |
| FEV1 | Girls | 3.50 | 113 | BCPE | 2.59675 | 1.0909 | 0.11455 | 6.62345 | 1.0909 | 0.8553 |
| FEV1 | Girls | 3.50 | 114 | BCPE | 2.59675 | 1.1102 | 0.11455 | 6.62345 | 1.1102 | 0.8704 |
| FEV1 | Girls | 3.50 | 115 | BCPE | 2.59675 | 1.1296 | 0.11455 | 6.62345 | 1.1296 | 0.8856 |
| FEV1 | Girls | 3.50 | 116 | BCPE | 2.59675 | 1.1492 | 0.11455 | 6.62345 | 1.1492 | 0.9010 |
| FEV1 | Girls | 3.50 | 117 | BCPE | 2.59675 | 1.1690 | 0.11455 | 6.62345 | 1.1690 | 0.9165 |
| FEV1 | Girls | 3.50 | 118 | BCPE | 2.59675 | 1.1889 | 0.11455 | 6.62345 | 1.1889 | 0.9321 |
| FEV1 | Girls | 3.50 | 119 | BCPE | 2.59675 | 1.2090 | 0.11455 | 6.62345 | 1.2090 | 0.9479 |
| FEV1 | Girls | 3.50 | 120 | BCPE | 2.59675 | 1.2293 | 0.11455 | 6.62345 | 1.2293 | 0.9638 |
| FEV1 | Girls | 3.50 | 121 | BCPE | 2.59675 | 1.2497 | 0.11455 | 6.62345 | 1.2497 | 0.9798 |
| FEV1 | Girls | 3.50 | 122 | BCPE | 2.59675 | 1.2703 | 0.11455 | 6.62345 | 1.2703 | 0.9959 |
| FEV1 | Girls | 3.50 | 123 | BCPE | 2.59675 | 1.2910 | 0.11455 | 6.62345 | 1.2910 | 1.0122 |
| FEV1 | Girls | 3.50 | 124 | BCPE | 2.59675 | 1.3120 | 0.11455 | 6.62345 | 1.3120 | 1.0286 |
| FEV1 | Girls | 3.75 | 88 | BCPE | 2.61715 | 0.6709 | 0.11476 | 6.63746 | 0.6709 | 0.5253 |
| FEV1 | Girls | 3.75 | 89 | BCPE | 2.61715 | 0.6862 | 0.11476 | 6.63746 | 0.6862 | 0.5372 |
| FEV1 | Girls | 3.75 | 90 | BCPE | 2.61715 | 0.7016 | 0.11476 | 6.63746 | 0.7016 | 0.5493 |
| FEV1 | Girls | 3.75 | 91 | BCPE | 2.61715 | 0.7171 | 0.11476 | 6.63746 | 0.7171 | 0.5615 |
| FEV1 | Girls | 3.75 | 92 | BCPE | 2.61715 | 0.7329 | 0.11476 | 6.63746 | 0.7329 | 0.5738 |
| FEV1 | Girls | 3.75 | 93 | BCPE | 2.61715 | 0.7488 | 0.11476 | 6.63746 | 0.7488 | 0.5862 |
| FEV1 | Girls | 3.75 | 94 | BCPE | 2.61715 | 0.7648 | 0.11476 | 6.63746 | 0.7648 | 0.5988 |
| FEV1 | Girls | 3.75 | 95 | BCPE | 2.61715 | 0.7811 | 0.11476 | 6.63746 | 0.7811 | 0.6116 |
| FEV1 | Girls | 3.75 | 96 | BCPE | 2.61715 | 0.7975 | 0.11476 | 6.63746 | 0.7975 | 0.6244 |
| FEV1 | Girls | 3.75 | 97 | BCPE | 2.61715 | 0.8141 | 0.11476 | 6.63746 | 0.8141 | 0.6374 |
| FEV1 | Girls | 3.75 | 98 | BCPE | 2.61715 | 0.8308 | 0.11476 | 6.63746 | 0.8308 | 0.6505 |
| FEV1 | Girls | 3.75 | 99 | BCPE | 2.61715 | 0.8478 | 0.11476 | 6.63746 | 0.8478 | 0.6638 |
| FEV1 | Girls | 3.75 | 100 | BCPE | 2.61715 | 0.8648 | 0.11476 | 6.63746 | 0.8648 | 0.6771 |
| FEV1 | Girls | 3.75 | 101 | BCPE | 2.61715 | 0.8821 | 0.11476 | 6.63746 | 0.8821 | 0.6906 |
| FEV1 | Girls | 3.75 | 102 | BCPE | 2.61715 | 0.8995 | 0.11476 | 6.63746 | 0.8995 | 0.7043 |
| FEV1 | Girls | 3.75 | 103 | BCPE | 2.61715 | 0.9171 | 0.11476 | 6.63746 | 0.9171 | 0.7181 |
| FEV1 | Girls | 3.75 | 104 | BCPE | 2.61715 | 0.9349 | 0.11476 | 6.63746 | 0.9349 | 0.7320 |
| FEV1 | Girls | 3.75 | 105 | BCPE | 2.61715 | 0.9528 | 0.11476 | 6.63746 | 0.9528 | 0.7460 |
| FEV1 | Girls | 3.75 | 106 | BCPE | 2.61715 | 0.9709 | 0.11476 | 6.63746 | 0.9709 | 0.7602 |
| FEV1 | Girls | 3.75 | 107 | BCPE | 2.61715 | 0.9892 | 0.11476 | 6.63746 | 0.9892 | 0.7745 |
| FEV1 | Girls | 3.75 | 108 | BCPE | 2.61715 | 1.0077 | 0.11476 | 6.63746 | 1.0077 | 0.7890 |
| FEV1 | Girls | 3.75 | 109 | BCPE | 2.61715 | 1.0263 | 0.11476 | 6.63746 | 1.0263 | 0.8035 |
| FEV1 | Girls | 3.75 | 110 | BCPE | 2.61715 | 1.0451 | 0.11476 | 6.63746 | 1.0451 | 0.8182 |
| FEV1 | Girls | 3.75 | 111 | BCPE | 2.61715 | 1.0640 | 0.11476 | 6.63746 | 1.0640 | 0.8331 |
| FEV1 | Girls | 3.75 | 112 | BCPE | 2.61715 | 1.0831 | 0.11476 | 6.63746 | 1.0831 | 0.8480 |
| FEV1 | Girls | 3.75 | 113 | BCPE | 2.61715 | 1.1024 | 0.11476 | 6.63746 | 1.1024 | 0.8631 |
| FEV1 | Girls | 3.75 | 114 | BCPE | 2.61715 | 1.1219 | 0.11476 | 6.63746 | 1.1219 | 0.8784 |
| FEV1 | Girls | 3.75 | 115 | BCPE | 2.61715 | 1.1415 | 0.11476 | 6.63746 | 1.1415 | 0.8938 |
| FEV1 | Girls | 3.75 | 116 | BCPE | 2.61715 | 1.1613 | 0.11476 | 6.63746 | 1.1613 | 0.9093 |
| FEV1 | Girls | 3.75 | 117 | BCPE | 2.61715 | 1.1813 | 0.11476 | 6.63746 | 1.1813 | 0.9249 |
| FEV1 | Girls | 3.75 | 118 | BCPE | 2.61715 | 1.2014 | 0.11476 | 6.63746 | 1.2014 | 0.9406 |
| FEV1 | Girls | 3.75 | 119 | BCPE | 2.61715 | 1.2217 | 0.11476 | 6.63746 | 1.2217 | 0.9565 |
| FEV1 | Girls | 3.75 | 120 | BCPE | 2.61715 | 1.2422 | 0.11476 | 6.63746 | 1.2422 | 0.9726 |
| FEV1 | Girls | 3.75 | 121 | BCPE | 2.61715 | 1.2628 | 0.11476 | 6.63746 | 1.2628 | 0.9887 |
| FEV1 | Girls | 3.75 | 122 | BCPE | 2.61715 | 1.2836 | 0.11476 | 6.63746 | 1.2836 | 1.0050 |
| FEV1 | Girls | 3.75 | 123 | BCPE | 2.61715 | 1.3046 | 0.11476 | 6.63746 | 1.3046 | 1.0215 |
| FEV1 | Girls | 3.75 | 124 | BCPE | 2.61715 | 1.3258 | 0.11476 | 6.63746 | 1.3258 | 1.0380 |
| FEV1 | Girls | 4.00 | 88 | BCPE | 2.63624 | 0.6776 | 0.11495 | 6.65059 | 0.6776 | 0.5298 |
| FEV1 | Girls | 4.00 | 89 | BCPE | 2.63624 | 0.6930 | 0.11495 | 6.65059 | 0.6930 | 0.5419 |
| FEV1 | Girls | 4.00 | 90 | BCPE | 2.63624 | 0.7085 | 0.11495 | 6.65059 | 0.7085 | 0.5540 |
| FEV1 | Girls | 4.00 | 91 | BCPE | 2.63624 | 0.7242 | 0.11495 | 6.65059 | 0.7242 | 0.5663 |
| FEV1 | Girls | 4.00 | 92 | BCPE | 2.63624 | 0.7401 | 0.11495 | 6.65059 | 0.7401 | 0.5787 |
| FEV1 | Girls | 4.00 | 93 | BCPE | 2.63624 | 0.7562 | 0.11495 | 6.65059 | 0.7562 | 0.5913 |
| FEV1 | Girls | 4.00 | 94 | BCPE | 2.63624 | 0.7724 | 0.11495 | 6.65059 | 0.7724 | 0.6040 |
| FEV1 | Girls | 4.00 | 95 | BCPE | 2.63624 | 0.7888 | 0.11495 | 6.65059 | 0.7888 | 0.6168 |
| FEV1 | Girls | 4.00 | 96 | BCPE | 2.63624 | 0.8054 | 0.11495 | 6.65059 | 0.8054 | 0.6298 |
| FEV1 | Girls | 4.00 | 97 | BCPE | 2.63624 | 0.8222 | 0.11495 | 6.65059 | 0.8222 | 0.6429 |
| FEV1 | Girls | 4.00 | 98 | BCPE | 2.63624 | 0.8391 | 0.11495 | 6.65059 | 0.8391 | 0.6561 |
| FEV1 | Girls | 4.00 | 99 | BCPE | 2.63624 | 0.8562 | 0.11495 | 6.65059 | 0.8562 | 0.6695 |
| FEV1 | Girls | 4.00 | 100 | BCPE | 2.63624 | 0.8734 | 0.11495 | 6.65059 | 0.8734 | 0.6830 |
| FEV1 | Girls | 4.00 | 101 | BCPE | 2.63624 | 0.8909 | 0.11495 | 6.65059 | 0.8909 | 0.6966 |
| FEV1 | Girls | 4.00 | 102 | BCPE | 2.63624 | 0.9085 | 0.11495 | 6.65059 | 0.9085 | 0.7104 |
| FEV1 | Girls | 4.00 | 103 | BCPE | 2.63624 | 0.9262 | 0.11495 | 6.65059 | 0.9262 | 0.7243 |
| FEV1 | Girls | 4.00 | 104 | BCPE | 2.63624 | 0.9442 | 0.11495 | 6.65059 | 0.9442 | 0.7383 |
| FEV1 | Girls | 4.00 | 105 | BCPE | 2.63624 | 0.9623 | 0.11495 | 6.65059 | 0.9623 | 0.7525 |
| FEV1 | Girls | 4.00 | 106 | BCPE | 2.63624 | 0.9806 | 0.11495 | 6.65059 | 0.9806 | 0.7668 |
| FEV1 | Girls | 4.00 | 107 | BCPE | 2.63624 | 0.9990 | 0.11495 | 6.65059 | 0.9990 | 0.7812 |
| FEV1 | Girls | 4.00 | 108 | BCPE | 2.63624 | 1.0177 | 0.11495 | 6.65059 | 1.0177 | 0.7958 |
| FEV1 | Girls | 4.00 | 109 | BCPE | 2.63624 | 1.0365 | 0.11495 | 6.65059 | 1.0365 | 0.8105 |
| FEV1 | Girls | 4.00 | 110 | BCPE | 2.63624 | 1.0554 | 0.11495 | 6.65059 | 1.0554 | 0.8253 |
| FEV1 | Girls | 4.00 | 111 | BCPE | 2.63624 | 1.0746 | 0.11495 | 6.65059 | 1.0746 | 0.8403 |
| FEV1 | Girls | 4.00 | 112 | BCPE | 2.63624 | 1.0939 | 0.11495 | 6.65059 | 1.0939 | 0.8554 |
| FEV1 | Girls | 4.00 | 113 | BCPE | 2.63624 | 1.1134 | 0.11495 | 6.65059 | 1.1134 | 0.8706 |
| FEV1 | Girls | 4.00 | 114 | BCPE | 2.63624 | 1.1330 | 0.11495 | 6.65059 | 1.1330 | 0.8860 |
| FEV1 | Girls | 4.00 | 115 | BCPE | 2.63624 | 1.1528 | 0.11495 | 6.65059 | 1.1528 | 0.9015 |
| FEV1 | Girls | 4.00 | 116 | BCPE | 2.63624 | 1.1728 | 0.11495 | 6.65059 | 1.1728 | 0.9171 |
| FEV1 | Girls | 4.00 | 117 | BCPE | 2.63624 | 1.1930 | 0.11495 | 6.65059 | 1.1930 | 0.9329 |
| FEV1 | Girls | 4.00 | 118 | BCPE | 2.63624 | 1.2133 | 0.11495 | 6.65059 | 1.2133 | 0.9488 |
| FEV1 | Girls | 4.00 | 119 | BCPE | 2.63624 | 1.2338 | 0.11495 | 6.65059 | 1.2338 | 0.9648 |
| FEV1 | Girls | 4.00 | 120 | BCPE | 2.63624 | 1.2545 | 0.11495 | 6.65059 | 1.2545 | 0.9810 |
| FEV1 | Girls | 4.00 | 121 | BCPE | 2.63624 | 1.2754 | 0.11495 | 6.65059 | 1.2754 | 0.9973 |
| FEV1 | Girls | 4.00 | 122 | BCPE | 2.63624 | 1.2964 | 0.11495 | 6.65059 | 1.2964 | 1.0137 |
| FEV1 | Girls | 4.00 | 123 | BCPE | 2.63624 | 1.3176 | 0.11495 | 6.65059 | 1.3176 | 1.0303 |
| FEV1 | Girls | 4.00 | 124 | BCPE | 2.63624 | 1.3389 | 0.11495 | 6.65059 | 1.3389 | 1.0470 |
| FEV1 | Girls | 4.25 | 88 | BCPE | 2.65418 | 0.6840 | 0.11513 | 6.66294 | 0.6840 | 0.5342 |
| FEV1 | Girls | 4.25 | 89 | BCPE | 2.65418 | 0.6995 | 0.11513 | 6.66294 | 0.6995 | 0.5463 |
| FEV1 | Girls | 4.25 | 90 | BCPE | 2.65418 | 0.7152 | 0.11513 | 6.66294 | 0.7152 | 0.5585 |
| FEV1 | Girls | 4.25 | 91 | BCPE | 2.65418 | 0.7310 | 0.11513 | 6.66294 | 0.7310 | 0.5709 |
| FEV1 | Girls | 4.25 | 92 | BCPE | 2.65418 | 0.7471 | 0.11513 | 6.66294 | 0.7471 | 0.5835 |
| FEV1 | Girls | 4.25 | 93 | BCPE | 2.65418 | 0.7633 | 0.11513 | 6.66294 | 0.7633 | 0.5961 |
| FEV1 | Girls | 4.25 | 94 | BCPE | 2.65418 | 0.7797 | 0.11513 | 6.66294 | 0.7797 | 0.6089 |
| FEV1 | Girls | 4.25 | 95 | BCPE | 2.65418 | 0.7962 | 0.11513 | 6.66294 | 0.7962 | 0.6219 |
| FEV1 | Girls | 4.25 | 96 | BCPE | 2.65418 | 0.8130 | 0.11513 | 6.66294 | 0.8130 | 0.6349 |
| FEV1 | Girls | 4.25 | 97 | BCPE | 2.65418 | 0.8299 | 0.11513 | 6.66294 | 0.8299 | 0.6481 |
| FEV1 | Girls | 4.25 | 98 | BCPE | 2.65418 | 0.8469 | 0.11513 | 6.66294 | 0.8469 | 0.6615 |
| FEV1 | Girls | 4.25 | 99 | BCPE | 2.65418 | 0.8642 | 0.11513 | 6.66294 | 0.8642 | 0.6749 |
| FEV1 | Girls | 4.25 | 100 | BCPE | 2.65418 | 0.8816 | 0.11513 | 6.66294 | 0.8816 | 0.6885 |
| FEV1 | Girls | 4.25 | 101 | BCPE | 2.65418 | 0.8992 | 0.11513 | 6.66294 | 0.8992 | 0.7023 |
| FEV1 | Girls | 4.25 | 102 | BCPE | 2.65418 | 0.9170 | 0.11513 | 6.66294 | 0.9170 | 0.7162 |
| FEV1 | Girls | 4.25 | 103 | BCPE | 2.65418 | 0.9349 | 0.11513 | 6.66294 | 0.9349 | 0.7302 |
| FEV1 | Girls | 4.25 | 104 | BCPE | 2.65418 | 0.9530 | 0.11513 | 6.66294 | 0.9530 | 0.7443 |
| FEV1 | Girls | 4.25 | 105 | BCPE | 2.65418 | 0.9713 | 0.11513 | 6.66294 | 0.9713 | 0.7586 |
| FEV1 | Girls | 4.25 | 106 | BCPE | 2.65418 | 0.9898 | 0.11513 | 6.66294 | 0.9898 | 0.7730 |
| FEV1 | Girls | 4.25 | 107 | BCPE | 2.65418 | 1.0084 | 0.11513 | 6.66294 | 1.0084 | 0.7876 |
| FEV1 | Girls | 4.25 | 108 | BCPE | 2.65418 | 1.0272 | 0.11513 | 6.66294 | 1.0272 | 0.8023 |
| FEV1 | Girls | 4.25 | 109 | BCPE | 2.65418 | 1.0462 | 0.11513 | 6.66294 | 1.0462 | 0.8171 |
| FEV1 | Girls | 4.25 | 110 | BCPE | 2.65418 | 1.0653 | 0.11513 | 6.66294 | 1.0653 | 0.8320 |
| FEV1 | Girls | 4.25 | 111 | BCPE | 2.65418 | 1.0846 | 0.11513 | 6.66294 | 1.0846 | 0.8471 |
| FEV1 | Girls | 4.25 | 112 | BCPE | 2.65418 | 1.1041 | 0.11513 | 6.66294 | 1.1041 | 0.8623 |
| FEV1 | Girls | 4.25 | 113 | BCPE | 2.65418 | 1.1238 | 0.11513 | 6.66294 | 1.1238 | 0.8777 |
| FEV1 | Girls | 4.25 | 114 | BCPE | 2.65418 | 1.1436 | 0.11513 | 6.66294 | 1.1436 | 0.8932 |
| FEV1 | Girls | 4.25 | 115 | BCPE | 2.65418 | 1.1636 | 0.11513 | 6.66294 | 1.1636 | 0.9088 |
| FEV1 | Girls | 4.25 | 116 | BCPE | 2.65418 | 1.1838 | 0.11513 | 6.66294 | 1.1838 | 0.9246 |
| FEV1 | Girls | 4.25 | 117 | BCPE | 2.65418 | 1.2042 | 0.11513 | 6.66294 | 1.2042 | 0.9405 |
| FEV1 | Girls | 4.25 | 118 | BCPE | 2.65418 | 1.2247 | 0.11513 | 6.66294 | 1.2247 | 0.9565 |
| FEV1 | Girls | 4.25 | 119 | BCPE | 2.65418 | 1.2454 | 0.11513 | 6.66294 | 1.2454 | 0.9727 |
| FEV1 | Girls | 4.25 | 120 | BCPE | 2.65418 | 1.2663 | 0.11513 | 6.66294 | 1.2663 | 0.9890 |
| FEV1 | Girls | 4.25 | 121 | BCPE | 2.65418 | 1.2873 | 0.11513 | 6.66294 | 1.2873 | 1.0054 |
| FEV1 | Girls | 4.25 | 122 | BCPE | 2.65418 | 1.3085 | 0.11513 | 6.66294 | 1.3085 | 1.0220 |
| FEV1 | Girls | 4.25 | 123 | BCPE | 2.65418 | 1.3299 | 0.11513 | 6.66294 | 1.3299 | 1.0387 |
| FEV1 | Girls | 4.25 | 124 | BCPE | 2.65418 | 1.3515 | 0.11513 | 6.66294 | 1.3515 | 1.0555 |
| FEV1 | Girls | 4.50 | 88 | BCPE | 2.67108 | 0.6900 | 0.11530 | 6.67461 | 0.6900 | 0.5383 |
| FEV1 | Girls | 4.50 | 89 | BCPE | 2.67108 | 0.7057 | 0.11530 | 6.67461 | 0.7057 | 0.5505 |
| FEV1 | Girls | 4.50 | 90 | BCPE | 2.67108 | 0.7215 | 0.11530 | 6.67461 | 0.7215 | 0.5629 |
| FEV1 | Girls | 4.50 | 91 | BCPE | 2.67108 | 0.7375 | 0.11530 | 6.67461 | 0.7375 | 0.5753 |
| FEV1 | Girls | 4.50 | 92 | BCPE | 2.67108 | 0.7537 | 0.11530 | 6.67461 | 0.7537 | 0.5880 |
| FEV1 | Girls | 4.50 | 93 | BCPE | 2.67108 | 0.7701 | 0.11530 | 6.67461 | 0.7701 | 0.6007 |
| FEV1 | Girls | 4.50 | 94 | BCPE | 2.67108 | 0.7866 | 0.11530 | 6.67461 | 0.7866 | 0.6136 |
| FEV1 | Girls | 4.50 | 95 | BCPE | 2.67108 | 0.8033 | 0.11530 | 6.67461 | 0.8033 | 0.6267 |
| FEV1 | Girls | 4.50 | 96 | BCPE | 2.67108 | 0.8202 | 0.11530 | 6.67461 | 0.8202 | 0.6398 |
| FEV1 | Girls | 4.50 | 97 | BCPE | 2.67108 | 0.8372 | 0.11530 | 6.67461 | 0.8372 | 0.6531 |
| FEV1 | Girls | 4.50 | 98 | BCPE | 2.67108 | 0.8545 | 0.11530 | 6.67461 | 0.8545 | 0.6666 |
| FEV1 | Girls | 4.50 | 99 | BCPE | 2.67108 | 0.8719 | 0.11530 | 6.67461 | 0.8719 | 0.6801 |
| FEV1 | Girls | 4.50 | 100 | BCPE | 2.67108 | 0.8894 | 0.11530 | 6.67461 | 0.8894 | 0.6939 |
| FEV1 | Girls | 4.50 | 101 | BCPE | 2.67108 | 0.9072 | 0.11530 | 6.67461 | 0.9072 | 0.7077 |
| FEV1 | Girls | 4.50 | 102 | BCPE | 2.67108 | 0.9251 | 0.11530 | 6.67461 | 0.9251 | 0.7217 |
| FEV1 | Girls | 4.50 | 103 | BCPE | 2.67108 | 0.9432 | 0.11530 | 6.67461 | 0.9432 | 0.7358 |
| FEV1 | Girls | 4.50 | 104 | BCPE | 2.67108 | 0.9615 | 0.11530 | 6.67461 | 0.9615 | 0.7501 |
| FEV1 | Girls | 4.50 | 105 | BCPE | 2.67108 | 0.9799 | 0.11530 | 6.67461 | 0.9799 | 0.7645 |
| FEV1 | Girls | 4.50 | 106 | BCPE | 2.67108 | 0.9986 | 0.11530 | 6.67461 | 0.9986 | 0.7790 |
| FEV1 | Girls | 4.50 | 107 | BCPE | 2.67108 | 1.0174 | 0.11530 | 6.67461 | 1.0174 | 0.7936 |
| FEV1 | Girls | 4.50 | 108 | BCPE | 2.67108 | 1.0363 | 0.11530 | 6.67461 | 1.0363 | 0.8084 |
| FEV1 | Girls | 4.50 | 109 | BCPE | 2.67108 | 1.0555 | 0.11530 | 6.67461 | 1.0555 | 0.8234 |
| FEV1 | Girls | 4.50 | 110 | BCPE | 2.67108 | 1.0748 | 0.11530 | 6.67461 | 1.0748 | 0.8384 |
| FEV1 | Girls | 4.50 | 111 | BCPE | 2.67108 | 1.0943 | 0.11530 | 6.67461 | 1.0943 | 0.8536 |
| FEV1 | Girls | 4.50 | 112 | BCPE | 2.67108 | 1.1139 | 0.11530 | 6.67461 | 1.1139 | 0.8690 |
| FEV1 | Girls | 4.50 | 113 | BCPE | 2.67108 | 1.1338 | 0.11530 | 6.67461 | 1.1338 | 0.8845 |
| FEV1 | Girls | 4.50 | 114 | BCPE | 2.67108 | 1.1538 | 0.11530 | 6.67461 | 1.1538 | 0.9001 |
| FEV1 | Girls | 4.50 | 115 | BCPE | 2.67108 | 1.1740 | 0.11530 | 6.67461 | 1.1740 | 0.9158 |
| FEV1 | Girls | 4.50 | 116 | BCPE | 2.67108 | 1.1943 | 0.11530 | 6.67461 | 1.1943 | 0.9317 |
| FEV1 | Girls | 4.50 | 117 | BCPE | 2.67108 | 1.2149 | 0.11530 | 6.67461 | 1.2149 | 0.9477 |
| FEV1 | Girls | 4.50 | 118 | BCPE | 2.67108 | 1.2356 | 0.11530 | 6.67461 | 1.2356 | 0.9639 |
| FEV1 | Girls | 4.50 | 119 | BCPE | 2.67108 | 1.2565 | 0.11530 | 6.67461 | 1.2565 | 0.9802 |
| FEV1 | Girls | 4.50 | 120 | BCPE | 2.67108 | 1.2775 | 0.11530 | 6.67461 | 1.2775 | 0.9966 |
| FEV1 | Girls | 4.50 | 121 | BCPE | 2.67108 | 1.2988 | 0.11530 | 6.67461 | 1.2988 | 1.0132 |
| FEV1 | Girls | 4.50 | 122 | BCPE | 2.67108 | 1.3202 | 0.11530 | 6.67461 | 1.3202 | 1.0299 |
| FEV1 | Girls | 4.50 | 123 | BCPE | 2.67108 | 1.3417 | 0.11530 | 6.67461 | 1.3417 | 1.0467 |
| FEV1 | Girls | 4.50 | 124 | BCPE | 2.67108 | 1.3635 | 0.11530 | 6.67461 | 1.3635 | 1.0637 |
| FEV1 | Girls | 4.75 | 88 | BCPE | 2.68708 | 0.6958 | 0.11547 | 6.68567 | 0.6958 | 0.5422 |
| FEV1 | Girls | 4.75 | 89 | BCPE | 2.68708 | 0.7116 | 0.11547 | 6.68567 | 0.7116 | 0.5545 |
| FEV1 | Girls | 4.75 | 90 | BCPE | 2.68708 | 0.7276 | 0.11547 | 6.68567 | 0.7276 | 0.5670 |
| FEV1 | Girls | 4.75 | 91 | BCPE | 2.68708 | 0.7437 | 0.11547 | 6.68567 | 0.7437 | 0.5795 |
| FEV1 | Girls | 4.75 | 92 | BCPE | 2.68708 | 0.7601 | 0.11547 | 6.68567 | 0.7601 | 0.5923 |
| FEV1 | Girls | 4.75 | 93 | BCPE | 2.68708 | 0.7765 | 0.11547 | 6.68567 | 0.7765 | 0.6051 |
| FEV1 | Girls | 4.75 | 94 | BCPE | 2.68708 | 0.7932 | 0.11547 | 6.68567 | 0.7932 | 0.6181 |
| FEV1 | Girls | 4.75 | 95 | BCPE | 2.68708 | 0.8101 | 0.11547 | 6.68567 | 0.8101 | 0.6312 |
| FEV1 | Girls | 4.75 | 96 | BCPE | 2.68708 | 0.8271 | 0.11547 | 6.68567 | 0.8271 | 0.6445 |
| FEV1 | Girls | 4.75 | 97 | BCPE | 2.68708 | 0.8443 | 0.11547 | 6.68567 | 0.8443 | 0.6579 |
| FEV1 | Girls | 4.75 | 98 | BCPE | 2.68708 | 0.8617 | 0.11547 | 6.68567 | 0.8617 | 0.6714 |
| FEV1 | Girls | 4.75 | 99 | BCPE | 2.68708 | 0.8792 | 0.11547 | 6.68567 | 0.8792 | 0.6851 |
| FEV1 | Girls | 4.75 | 100 | BCPE | 2.68708 | 0.8969 | 0.11547 | 6.68567 | 0.8969 | 0.6989 |
| FEV1 | Girls | 4.75 | 101 | BCPE | 2.68708 | 0.9148 | 0.11547 | 6.68567 | 0.9148 | 0.7129 |
| FEV1 | Girls | 4.75 | 102 | BCPE | 2.68708 | 0.9329 | 0.11547 | 6.68567 | 0.9329 | 0.7270 |
| FEV1 | Girls | 4.75 | 103 | BCPE | 2.68708 | 0.9512 | 0.11547 | 6.68567 | 0.9512 | 0.7412 |
| FEV1 | Girls | 4.75 | 104 | BCPE | 2.68708 | 0.9696 | 0.11547 | 6.68567 | 0.9696 | 0.7555 |
| FEV1 | Girls | 4.75 | 105 | BCPE | 2.68708 | 0.9882 | 0.11547 | 6.68567 | 0.9882 | 0.7700 |
| FEV1 | Girls | 4.75 | 106 | BCPE | 2.68708 | 1.0070 | 0.11547 | 6.68567 | 1.0070 | 0.7847 |
| FEV1 | Girls | 4.75 | 107 | BCPE | 2.68708 | 1.0259 | 0.11547 | 6.68567 | 1.0259 | 0.7994 |
| FEV1 | Girls | 4.75 | 108 | BCPE | 2.68708 | 1.0450 | 0.11547 | 6.68567 | 1.0450 | 0.8143 |
| FEV1 | Girls | 4.75 | 109 | BCPE | 2.68708 | 1.0644 | 0.11547 | 6.68567 | 1.0644 | 0.8294 |
| FEV1 | Girls | 4.75 | 110 | BCPE | 2.68708 | 1.0838 | 0.11547 | 6.68567 | 1.0838 | 0.8446 |
| FEV1 | Girls | 4.75 | 111 | BCPE | 2.68708 | 1.1035 | 0.11547 | 6.68567 | 1.1035 | 0.8599 |
| FEV1 | Girls | 4.75 | 112 | BCPE | 2.68708 | 1.1233 | 0.11547 | 6.68567 | 1.1233 | 0.8753 |
| FEV1 | Girls | 4.75 | 113 | BCPE | 2.68708 | 1.1433 | 0.11547 | 6.68567 | 1.1433 | 0.8909 |
| FEV1 | Girls | 4.75 | 114 | BCPE | 2.68708 | 1.1635 | 0.11547 | 6.68567 | 1.1635 | 0.9066 |
| FEV1 | Girls | 4.75 | 115 | BCPE | 2.68708 | 1.1839 | 0.11547 | 6.68567 | 1.1839 | 0.9225 |
| FEV1 | Girls | 4.75 | 116 | BCPE | 2.68708 | 1.2044 | 0.11547 | 6.68567 | 1.2044 | 0.9385 |
| FEV1 | Girls | 4.75 | 117 | BCPE | 2.68708 | 1.2251 | 0.11547 | 6.68567 | 1.2251 | 0.9546 |
| FEV1 | Girls | 4.75 | 118 | BCPE | 2.68708 | 1.2460 | 0.11547 | 6.68567 | 1.2460 | 0.9709 |
| FEV1 | Girls | 4.75 | 119 | BCPE | 2.68708 | 1.2670 | 0.11547 | 6.68567 | 1.2670 | 0.9873 |
| FEV1 | Girls | 4.75 | 120 | BCPE | 2.68708 | 1.2883 | 0.11547 | 6.68567 | 1.2883 | 1.0039 |
| FEV1 | Girls | 4.75 | 121 | BCPE | 2.68708 | 1.3097 | 0.11547 | 6.68567 | 1.3097 | 1.0205 |
| FEV1 | Girls | 4.75 | 122 | BCPE | 2.68708 | 1.3313 | 0.11547 | 6.68567 | 1.3313 | 1.0374 |
| FEV1 | Girls | 4.75 | 123 | BCPE | 2.68708 | 1.3530 | 0.11547 | 6.68567 | 1.3530 | 1.0543 |
| FEV1 | Girls | 4.75 | 124 | BCPE | 2.68708 | 1.3750 | 0.11547 | 6.68567 | 1.3750 | 1.0714 |
| FEV1 | Girls | 5.00 | 88 | BCPE | 2.70225 | 0.7014 | 0.11562 | 6.69618 | 0.7014 | 0.5460 |
| FEV1 | Girls | 5.00 | 89 | BCPE | 2.70225 | 0.7173 | 0.11562 | 6.69618 | 0.7173 | 0.5584 |
| FEV1 | Girls | 5.00 | 90 | BCPE | 2.70225 | 0.7334 | 0.11562 | 6.69618 | 0.7334 | 0.5709 |
| FEV1 | Girls | 5.00 | 91 | BCPE | 2.70225 | 0.7497 | 0.11562 | 6.69618 | 0.7497 | 0.5836 |
| FEV1 | Girls | 5.00 | 92 | BCPE | 2.70225 | 0.7661 | 0.11562 | 6.69618 | 0.7661 | 0.5964 |
| FEV1 | Girls | 5.00 | 93 | BCPE | 2.70225 | 0.7828 | 0.11562 | 6.69618 | 0.7828 | 0.6093 |
| FEV1 | Girls | 5.00 | 94 | BCPE | 2.70225 | 0.7996 | 0.11562 | 6.69618 | 0.7996 | 0.6224 |
| FEV1 | Girls | 5.00 | 95 | BCPE | 2.70225 | 0.8165 | 0.11562 | 6.69618 | 0.8165 | 0.6356 |
| FEV1 | Girls | 5.00 | 96 | BCPE | 2.70225 | 0.8337 | 0.11562 | 6.69618 | 0.8337 | 0.6490 |
| FEV1 | Girls | 5.00 | 97 | BCPE | 2.70225 | 0.8510 | 0.11562 | 6.69618 | 0.8510 | 0.6625 |
| FEV1 | Girls | 5.00 | 98 | BCPE | 2.70225 | 0.8685 | 0.11562 | 6.69618 | 0.8685 | 0.6761 |
| FEV1 | Girls | 5.00 | 99 | BCPE | 2.70225 | 0.8862 | 0.11562 | 6.69618 | 0.8862 | 0.6899 |
| FEV1 | Girls | 5.00 | 100 | BCPE | 2.70225 | 0.9041 | 0.11562 | 6.69618 | 0.9041 | 0.7038 |
| FEV1 | Girls | 5.00 | 101 | BCPE | 2.70225 | 0.9221 | 0.11562 | 6.69618 | 0.9221 | 0.7178 |
| FEV1 | Girls | 5.00 | 102 | BCPE | 2.70225 | 0.9404 | 0.11562 | 6.69618 | 0.9404 | 0.7320 |
| FEV1 | Girls | 5.00 | 103 | BCPE | 2.70225 | 0.9588 | 0.11562 | 6.69618 | 0.9588 | 0.7463 |
| FEV1 | Girls | 5.00 | 104 | BCPE | 2.70225 | 0.9773 | 0.11562 | 6.69618 | 0.9773 | 0.7608 |
| FEV1 | Girls | 5.00 | 105 | BCPE | 2.70225 | 0.9961 | 0.11562 | 6.69618 | 0.9961 | 0.7754 |
| FEV1 | Girls | 5.00 | 106 | BCPE | 2.70225 | 1.0150 | 0.11562 | 6.69618 | 1.0150 | 0.7901 |
| FEV1 | Girls | 5.00 | 107 | BCPE | 2.70225 | 1.0341 | 0.11562 | 6.69618 | 1.0341 | 0.8050 |
| FEV1 | Girls | 5.00 | 108 | BCPE | 2.70225 | 1.0534 | 0.11562 | 6.69618 | 1.0534 | 0.8200 |
| FEV1 | Girls | 5.00 | 109 | BCPE | 2.70225 | 1.0729 | 0.11562 | 6.69618 | 1.0729 | 0.8351 |
| FEV1 | Girls | 5.00 | 110 | BCPE | 2.70225 | 1.0925 | 0.11562 | 6.69618 | 1.0925 | 0.8504 |
| FEV1 | Girls | 5.00 | 111 | BCPE | 2.70225 | 1.1123 | 0.11562 | 6.69618 | 1.1123 | 0.8658 |
| FEV1 | Girls | 5.00 | 112 | BCPE | 2.70225 | 1.1323 | 0.11562 | 6.69618 | 1.1323 | 0.8814 |
| FEV1 | Girls | 5.00 | 113 | BCPE | 2.70225 | 1.1525 | 0.11562 | 6.69618 | 1.1525 | 0.8971 |
| FEV1 | Girls | 5.00 | 114 | BCPE | 2.70225 | 1.1728 | 0.11562 | 6.69618 | 1.1728 | 0.9129 |
| FEV1 | Girls | 5.00 | 115 | BCPE | 2.70225 | 1.1933 | 0.11562 | 6.69618 | 1.1933 | 0.9289 |
| FEV1 | Girls | 5.00 | 116 | BCPE | 2.70225 | 1.2140 | 0.11562 | 6.69618 | 1.2140 | 0.9450 |
| FEV1 | Girls | 5.00 | 117 | BCPE | 2.70225 | 1.2349 | 0.11562 | 6.69618 | 1.2349 | 0.9613 |
| FEV1 | Girls | 5.00 | 118 | BCPE | 2.70225 | 1.2560 | 0.11562 | 6.69618 | 1.2560 | 0.9776 |
| FEV1 | Girls | 5.00 | 119 | BCPE | 2.70225 | 1.2772 | 0.11562 | 6.69618 | 1.2772 | 0.9942 |
| FEV1 | Girls | 5.00 | 120 | BCPE | 2.70225 | 1.2986 | 0.11562 | 6.69618 | 1.2986 | 1.0108 |
| FEV1 | Girls | 5.00 | 121 | BCPE | 2.70225 | 1.3202 | 0.11562 | 6.69618 | 1.3202 | 1.0276 |
| FEV1 | Girls | 5.00 | 122 | BCPE | 2.70225 | 1.3419 | 0.11562 | 6.69618 | 1.3419 | 1.0446 |
| FEV1 | Girls | 5.00 | 123 | BCPE | 2.70225 | 1.3638 | 0.11562 | 6.69618 | 1.3638 | 1.0616 |
| FEV1 | Girls | 5.00 | 124 | BCPE | 2.70225 | 1.3860 | 0.11562 | 6.69618 | 1.3860 | 1.0788 |
| FEV1 | Girls | 5.25 | 88 | BCPE | 2.71668 | 0.7067 | 0.11577 | 6.70619 | 0.7067 | 0.5496 |
| FEV1 | Girls | 5.25 | 89 | BCPE | 2.71668 | 0.7228 | 0.11577 | 6.70619 | 0.7228 | 0.5620 |
| FEV1 | Girls | 5.25 | 90 | BCPE | 2.71668 | 0.7390 | 0.11577 | 6.70619 | 0.7390 | 0.5747 |
| FEV1 | Girls | 5.25 | 91 | BCPE | 2.71668 | 0.7554 | 0.11577 | 6.70619 | 0.7554 | 0.5874 |
| FEV1 | Girls | 5.25 | 92 | BCPE | 2.71668 | 0.7720 | 0.11577 | 6.70619 | 0.7720 | 0.6003 |
| FEV1 | Girls | 5.25 | 93 | BCPE | 2.71668 | 0.7887 | 0.11577 | 6.70619 | 0.7887 | 0.6133 |
| FEV1 | Girls | 5.25 | 94 | BCPE | 2.71668 | 0.8057 | 0.11577 | 6.70619 | 0.8057 | 0.6265 |
| FEV1 | Girls | 5.25 | 95 | BCPE | 2.71668 | 0.8228 | 0.11577 | 6.70619 | 0.8228 | 0.6398 |
| FEV1 | Girls | 5.25 | 96 | BCPE | 2.71668 | 0.8401 | 0.11577 | 6.70619 | 0.8401 | 0.6532 |
| FEV1 | Girls | 5.25 | 97 | BCPE | 2.71668 | 0.8575 | 0.11577 | 6.70619 | 0.8575 | 0.6668 |
| FEV1 | Girls | 5.25 | 98 | BCPE | 2.71668 | 0.8752 | 0.11577 | 6.70619 | 0.8752 | 0.6805 |
| FEV1 | Girls | 5.25 | 99 | BCPE | 2.71668 | 0.8930 | 0.11577 | 6.70619 | 0.8930 | 0.6944 |
| FEV1 | Girls | 5.25 | 100 | BCPE | 2.71668 | 0.9110 | 0.11577 | 6.70619 | 0.9110 | 0.7084 |
| FEV1 | Girls | 5.25 | 101 | BCPE | 2.71668 | 0.9292 | 0.11577 | 6.70619 | 0.9292 | 0.7225 |
| FEV1 | Girls | 5.25 | 102 | BCPE | 2.71668 | 0.9475 | 0.11577 | 6.70619 | 0.9475 | 0.7368 |
| FEV1 | Girls | 5.25 | 103 | BCPE | 2.71668 | 0.9661 | 0.11577 | 6.70619 | 0.9661 | 0.7512 |
| FEV1 | Girls | 5.25 | 104 | BCPE | 2.71668 | 0.9848 | 0.11577 | 6.70619 | 0.9848 | 0.7658 |
| FEV1 | Girls | 5.25 | 105 | BCPE | 2.71668 | 1.0037 | 0.11577 | 6.70619 | 1.0037 | 0.7805 |
| FEV1 | Girls | 5.25 | 106 | BCPE | 2.71668 | 1.0228 | 0.11577 | 6.70619 | 1.0228 | 0.7953 |
| FEV1 | Girls | 5.25 | 107 | BCPE | 2.71668 | 1.0420 | 0.11577 | 6.70619 | 1.0420 | 0.8103 |
| FEV1 | Girls | 5.25 | 108 | BCPE | 2.71668 | 1.0614 | 0.11577 | 6.70619 | 1.0614 | 0.8254 |
| FEV1 | Girls | 5.25 | 109 | BCPE | 2.71668 | 1.0811 | 0.11577 | 6.70619 | 1.0811 | 0.8406 |
| FEV1 | Girls | 5.25 | 110 | BCPE | 2.71668 | 1.1008 | 0.11577 | 6.70619 | 1.1008 | 0.8560 |
| FEV1 | Girls | 5.25 | 111 | BCPE | 2.71668 | 1.1208 | 0.11577 | 6.70619 | 1.1208 | 0.8715 |
| FEV1 | Girls | 5.25 | 112 | BCPE | 2.71668 | 1.1409 | 0.11577 | 6.70619 | 1.1409 | 0.8872 |
| FEV1 | Girls | 5.25 | 113 | BCPE | 2.71668 | 1.1613 | 0.11577 | 6.70619 | 1.1613 | 0.9030 |
| FEV1 | Girls | 5.25 | 114 | BCPE | 2.71668 | 1.1818 | 0.11577 | 6.70619 | 1.1818 | 0.9189 |
| FEV1 | Girls | 5.25 | 115 | BCPE | 2.71668 | 1.2024 | 0.11577 | 6.70619 | 1.2024 | 0.9350 |
| FEV1 | Girls | 5.25 | 116 | BCPE | 2.71668 | 1.2233 | 0.11577 | 6.70619 | 1.2233 | 0.9512 |
| FEV1 | Girls | 5.25 | 117 | BCPE | 2.71668 | 1.2443 | 0.11577 | 6.70619 | 1.2443 | 0.9676 |
| FEV1 | Girls | 5.25 | 118 | BCPE | 2.71668 | 1.2655 | 0.11577 | 6.70619 | 1.2655 | 0.9841 |
| FEV1 | Girls | 5.25 | 119 | BCPE | 2.71668 | 1.2869 | 0.11577 | 6.70619 | 1.2869 | 1.0007 |
| FEV1 | Girls | 5.25 | 120 | BCPE | 2.71668 | 1.3085 | 0.11577 | 6.70619 | 1.3085 | 1.0175 |
| FEV1 | Girls | 5.25 | 121 | BCPE | 2.71668 | 1.3302 | 0.11577 | 6.70619 | 1.3302 | 1.0344 |
| FEV1 | Girls | 5.25 | 122 | BCPE | 2.71668 | 1.3522 | 0.11577 | 6.70619 | 1.3522 | 1.0514 |
| FEV1 | Girls | 5.25 | 123 | BCPE | 2.71668 | 1.3743 | 0.11577 | 6.70619 | 1.3743 | 1.0686 |
| FEV1 | Girls | 5.25 | 124 | BCPE | 2.71668 | 1.3965 | 0.11577 | 6.70619 | 1.3965 | 1.0860 |
| FEV1 | Girls | 5.50 | 88 | BCPE | 2.73044 | 0.7119 | 0.11591 | 6.71575 | 0.7119 | 0.5530 |
| FEV1 | Girls | 5.50 | 89 | BCPE | 2.73044 | 0.7281 | 0.11591 | 6.71575 | 0.7281 | 0.5656 |
| FEV1 | Girls | 5.50 | 90 | BCPE | 2.73044 | 0.7444 | 0.11591 | 6.71575 | 0.7444 | 0.5783 |
| FEV1 | Girls | 5.50 | 91 | BCPE | 2.73044 | 0.7609 | 0.11591 | 6.71575 | 0.7609 | 0.5911 |
| FEV1 | Girls | 5.50 | 92 | BCPE | 2.73044 | 0.7776 | 0.11591 | 6.71575 | 0.7776 | 0.6041 |
| FEV1 | Girls | 5.50 | 93 | BCPE | 2.73044 | 0.7945 | 0.11591 | 6.71575 | 0.7945 | 0.6172 |
| FEV1 | Girls | 5.50 | 94 | BCPE | 2.73044 | 0.8115 | 0.11591 | 6.71575 | 0.8115 | 0.6304 |
| FEV1 | Girls | 5.50 | 95 | BCPE | 2.73044 | 0.8288 | 0.11591 | 6.71575 | 0.8288 | 0.6438 |
| FEV1 | Girls | 5.50 | 96 | BCPE | 2.73044 | 0.8462 | 0.11591 | 6.71575 | 0.8462 | 0.6574 |
| FEV1 | Girls | 5.50 | 97 | BCPE | 2.73044 | 0.8638 | 0.11591 | 6.71575 | 0.8638 | 0.6710 |
| FEV1 | Girls | 5.50 | 98 | BCPE | 2.73044 | 0.8816 | 0.11591 | 6.71575 | 0.8816 | 0.6848 |
| FEV1 | Girls | 5.50 | 99 | BCPE | 2.73044 | 0.8995 | 0.11591 | 6.71575 | 0.8995 | 0.6988 |
| FEV1 | Girls | 5.50 | 100 | BCPE | 2.73044 | 0.9177 | 0.11591 | 6.71575 | 0.9177 | 0.7129 |
| FEV1 | Girls | 5.50 | 101 | BCPE | 2.73044 | 0.9360 | 0.11591 | 6.71575 | 0.9360 | 0.7271 |
| FEV1 | Girls | 5.50 | 102 | BCPE | 2.73044 | 0.9545 | 0.11591 | 6.71575 | 0.9545 | 0.7415 |
| FEV1 | Girls | 5.50 | 103 | BCPE | 2.73044 | 0.9731 | 0.11591 | 6.71575 | 0.9731 | 0.7560 |
| FEV1 | Girls | 5.50 | 104 | BCPE | 2.73044 | 0.9920 | 0.11591 | 6.71575 | 0.9920 | 0.7706 |
| FEV1 | Girls | 5.50 | 105 | BCPE | 2.73044 | 1.0110 | 0.11591 | 6.71575 | 1.0110 | 0.7854 |
| FEV1 | Girls | 5.50 | 106 | BCPE | 2.73044 | 1.0302 | 0.11591 | 6.71575 | 1.0302 | 0.8003 |
| FEV1 | Girls | 5.50 | 107 | BCPE | 2.73044 | 1.0496 | 0.11591 | 6.71575 | 1.0496 | 0.8154 |
| FEV1 | Girls | 5.50 | 108 | BCPE | 2.73044 | 1.0692 | 0.11591 | 6.71575 | 1.0692 | 0.8306 |
| FEV1 | Girls | 5.50 | 109 | BCPE | 2.73044 | 1.0889 | 0.11591 | 6.71575 | 1.0889 | 0.8459 |
| FEV1 | Girls | 5.50 | 110 | BCPE | 2.73044 | 1.1089 | 0.11591 | 6.71575 | 1.1089 | 0.8614 |
| FEV1 | Girls | 5.50 | 111 | BCPE | 2.73044 | 1.1290 | 0.11591 | 6.71575 | 1.1290 | 0.8770 |
| FEV1 | Girls | 5.50 | 112 | BCPE | 2.73044 | 1.1493 | 0.11591 | 6.71575 | 1.1493 | 0.8928 |
| FEV1 | Girls | 5.50 | 113 | BCPE | 2.73044 | 1.1697 | 0.11591 | 6.71575 | 1.1697 | 0.9087 |
| FEV1 | Girls | 5.50 | 114 | BCPE | 2.73044 | 1.1904 | 0.11591 | 6.71575 | 1.1904 | 0.9247 |
| FEV1 | Girls | 5.50 | 115 | BCPE | 2.73044 | 1.2112 | 0.11591 | 6.71575 | 1.2112 | 0.9409 |
| FEV1 | Girls | 5.50 | 116 | BCPE | 2.73044 | 1.2322 | 0.11591 | 6.71575 | 1.2322 | 0.9572 |
| FEV1 | Girls | 5.50 | 117 | BCPE | 2.73044 | 1.2534 | 0.11591 | 6.71575 | 1.2534 | 0.9737 |
| FEV1 | Girls | 5.50 | 118 | BCPE | 2.73044 | 1.2748 | 0.11591 | 6.71575 | 1.2748 | 0.9903 |
| FEV1 | Girls | 5.50 | 119 | BCPE | 2.73044 | 1.2963 | 0.11591 | 6.71575 | 1.2963 | 1.0070 |
| FEV1 | Girls | 5.50 | 120 | BCPE | 2.73044 | 1.3180 | 0.11591 | 6.71575 | 1.3180 | 1.0239 |
| FEV1 | Girls | 5.50 | 121 | BCPE | 2.73044 | 1.3399 | 0.11591 | 6.71575 | 1.3399 | 1.0409 |
| FEV1 | Girls | 5.50 | 122 | BCPE | 2.73044 | 1.3620 | 0.11591 | 6.71575 | 1.3620 | 1.0581 |
| FEV1 | Girls | 5.50 | 123 | BCPE | 2.73044 | 1.3843 | 0.11591 | 6.71575 | 1.3843 | 1.0754 |
| FEV1 | Girls | 5.50 | 124 | BCPE | 2.73044 | 1.4067 | 0.11591 | 6.71575 | 1.4067 | 1.0928 |
| FEV1 | Girls | 5.75 | 88 | BCPE | 2.74359 | 0.7169 | 0.11605 | 6.72490 | 0.7169 | 0.5564 |
| FEV1 | Girls | 5.75 | 89 | BCPE | 2.74359 | 0.7331 | 0.11605 | 6.72490 | 0.7331 | 0.5690 |
| FEV1 | Girls | 5.75 | 90 | BCPE | 2.74359 | 0.7496 | 0.11605 | 6.72490 | 0.7496 | 0.5818 |
| FEV1 | Girls | 5.75 | 91 | BCPE | 2.74359 | 0.7662 | 0.11605 | 6.72490 | 0.7662 | 0.5947 |
| FEV1 | Girls | 5.75 | 92 | BCPE | 2.74359 | 0.7830 | 0.11605 | 6.72490 | 0.7830 | 0.6077 |
| FEV1 | Girls | 5.75 | 93 | BCPE | 2.74359 | 0.8000 | 0.11605 | 6.72490 | 0.8000 | 0.6209 |
| FEV1 | Girls | 5.75 | 94 | BCPE | 2.74359 | 0.8172 | 0.11605 | 6.72490 | 0.8172 | 0.6342 |
| FEV1 | Girls | 5.75 | 95 | BCPE | 2.74359 | 0.8346 | 0.11605 | 6.72490 | 0.8346 | 0.6477 |
| FEV1 | Girls | 5.75 | 96 | BCPE | 2.74359 | 0.8521 | 0.11605 | 6.72490 | 0.8521 | 0.6613 |
| FEV1 | Girls | 5.75 | 97 | BCPE | 2.74359 | 0.8698 | 0.11605 | 6.72490 | 0.8698 | 0.6751 |
| FEV1 | Girls | 5.75 | 98 | BCPE | 2.74359 | 0.8877 | 0.11605 | 6.72490 | 0.8877 | 0.6890 |
| FEV1 | Girls | 5.75 | 99 | BCPE | 2.74359 | 0.9058 | 0.11605 | 6.72490 | 0.9058 | 0.7030 |
| FEV1 | Girls | 5.75 | 100 | BCPE | 2.74359 | 0.9241 | 0.11605 | 6.72490 | 0.9241 | 0.7172 |
| FEV1 | Girls | 5.75 | 101 | BCPE | 2.74359 | 0.9425 | 0.11605 | 6.72490 | 0.9425 | 0.7315 |
| FEV1 | Girls | 5.75 | 102 | BCPE | 2.74359 | 0.9611 | 0.11605 | 6.72490 | 0.9611 | 0.7459 |
| FEV1 | Girls | 5.75 | 103 | BCPE | 2.74359 | 0.9799 | 0.11605 | 6.72490 | 0.9799 | 0.7605 |
| FEV1 | Girls | 5.75 | 104 | BCPE | 2.74359 | 0.9989 | 0.11605 | 6.72490 | 0.9989 | 0.7753 |
| FEV1 | Girls | 5.75 | 105 | BCPE | 2.74359 | 1.0181 | 0.11605 | 6.72490 | 1.0181 | 0.7901 |
| FEV1 | Girls | 5.75 | 106 | BCPE | 2.74359 | 1.0374 | 0.11605 | 6.72490 | 1.0374 | 0.8051 |
| FEV1 | Girls | 5.75 | 107 | BCPE | 2.74359 | 1.0570 | 0.11605 | 6.72490 | 1.0570 | 0.8203 |
| FEV1 | Girls | 5.75 | 108 | BCPE | 2.74359 | 1.0767 | 0.11605 | 6.72490 | 1.0767 | 0.8356 |
| FEV1 | Girls | 5.75 | 109 | BCPE | 2.74359 | 1.0965 | 0.11605 | 6.72490 | 1.0965 | 0.8510 |
| FEV1 | Girls | 5.75 | 110 | BCPE | 2.74359 | 1.1166 | 0.11605 | 6.72490 | 1.1166 | 0.8666 |
| FEV1 | Girls | 5.75 | 111 | BCPE | 2.74359 | 1.1369 | 0.11605 | 6.72490 | 1.1369 | 0.8823 |
| FEV1 | Girls | 5.75 | 112 | BCPE | 2.74359 | 1.1573 | 0.11605 | 6.72490 | 1.1573 | 0.8982 |
| FEV1 | Girls | 5.75 | 113 | BCPE | 2.74359 | 1.1779 | 0.11605 | 6.72490 | 1.1779 | 0.9142 |
| FEV1 | Girls | 5.75 | 114 | BCPE | 2.74359 | 1.1987 | 0.11605 | 6.72490 | 1.1987 | 0.9303 |
| FEV1 | Girls | 5.75 | 115 | BCPE | 2.74359 | 1.2197 | 0.11605 | 6.72490 | 1.2197 | 0.9466 |
| FEV1 | Girls | 5.75 | 116 | BCPE | 2.74359 | 1.2408 | 0.11605 | 6.72490 | 1.2408 | 0.9630 |
| FEV1 | Girls | 5.75 | 117 | BCPE | 2.74359 | 1.2622 | 0.11605 | 6.72490 | 1.2622 | 0.9796 |
| FEV1 | Girls | 5.75 | 118 | BCPE | 2.74359 | 1.2837 | 0.11605 | 6.72490 | 1.2837 | 0.9963 |
| FEV1 | Girls | 5.75 | 119 | BCPE | 2.74359 | 1.3054 | 0.11605 | 6.72490 | 1.3054 | 1.0131 |
| FEV1 | Girls | 5.75 | 120 | BCPE | 2.74359 | 1.3272 | 0.11605 | 6.72490 | 1.3272 | 1.0301 |
| FEV1 | Girls | 5.75 | 121 | BCPE | 2.74359 | 1.3493 | 0.11605 | 6.72490 | 1.3493 | 1.0472 |
| FEV1 | Girls | 5.75 | 122 | BCPE | 2.74359 | 1.3715 | 0.11605 | 6.72490 | 1.3715 | 1.0645 |
| FEV1 | Girls | 5.75 | 123 | BCPE | 2.74359 | 1.3940 | 0.11605 | 6.72490 | 1.3940 | 1.0818 |
| FEV1 | Girls | 5.75 | 124 | BCPE | 2.74359 | 1.4165 | 0.11605 | 6.72490 | 1.4165 | 1.0994 |
| FEV1 | Girls | 6.00 | 88 | BCPE | 2.75618 | 0.7217 | 0.11618 | 6.73367 | 0.7217 | 0.5596 |
| FEV1 | Girls | 6.00 | 89 | BCPE | 2.75618 | 0.7381 | 0.11618 | 6.73367 | 0.7381 | 0.5723 |
| FEV1 | Girls | 6.00 | 90 | BCPE | 2.75618 | 0.7546 | 0.11618 | 6.73367 | 0.7546 | 0.5851 |
| FEV1 | Girls | 6.00 | 91 | BCPE | 2.75618 | 0.7714 | 0.11618 | 6.73367 | 0.7714 | 0.5981 |
| FEV1 | Girls | 6.00 | 92 | BCPE | 2.75618 | 0.7883 | 0.11618 | 6.73367 | 0.7883 | 0.6112 |
| FEV1 | Girls | 6.00 | 93 | BCPE | 2.75618 | 0.8054 | 0.11618 | 6.73367 | 0.8054 | 0.6245 |
| FEV1 | Girls | 6.00 | 94 | BCPE | 2.75618 | 0.8227 | 0.11618 | 6.73367 | 0.8227 | 0.6379 |
| FEV1 | Girls | 6.00 | 95 | BCPE | 2.75618 | 0.8401 | 0.11618 | 6.73367 | 0.8401 | 0.6514 |
| FEV1 | Girls | 6.00 | 96 | BCPE | 2.75618 | 0.8578 | 0.11618 | 6.73367 | 0.8578 | 0.6651 |
| FEV1 | Girls | 6.00 | 97 | BCPE | 2.75618 | 0.8756 | 0.11618 | 6.73367 | 0.8756 | 0.6790 |
| FEV1 | Girls | 6.00 | 98 | BCPE | 2.75618 | 0.8937 | 0.11618 | 6.73367 | 0.8937 | 0.6929 |
| FEV1 | Girls | 6.00 | 99 | BCPE | 2.75618 | 0.9119 | 0.11618 | 6.73367 | 0.9119 | 0.7070 |
| FEV1 | Girls | 6.00 | 100 | BCPE | 2.75618 | 0.9302 | 0.11618 | 6.73367 | 0.9302 | 0.7213 |
| FEV1 | Girls | 6.00 | 101 | BCPE | 2.75618 | 0.9488 | 0.11618 | 6.73367 | 0.9488 | 0.7357 |
| FEV1 | Girls | 6.00 | 102 | BCPE | 2.75618 | 0.9676 | 0.11618 | 6.73367 | 0.9676 | 0.7502 |
| FEV1 | Girls | 6.00 | 103 | BCPE | 2.75618 | 0.9865 | 0.11618 | 6.73367 | 0.9865 | 0.7649 |
| FEV1 | Girls | 6.00 | 104 | BCPE | 2.75618 | 1.0056 | 0.11618 | 6.73367 | 1.0056 | 0.7797 |
| FEV1 | Girls | 6.00 | 105 | BCPE | 2.75618 | 1.0249 | 0.11618 | 6.73367 | 1.0249 | 0.7947 |
| FEV1 | Girls | 6.00 | 106 | BCPE | 2.75618 | 1.0444 | 0.11618 | 6.73367 | 1.0444 | 0.8098 |
| FEV1 | Girls | 6.00 | 107 | BCPE | 2.75618 | 1.0640 | 0.11618 | 6.73367 | 1.0640 | 0.8250 |
| FEV1 | Girls | 6.00 | 108 | BCPE | 2.75618 | 1.0839 | 0.11618 | 6.73367 | 1.0839 | 0.8404 |
| FEV1 | Girls | 6.00 | 109 | BCPE | 2.75618 | 1.1039 | 0.11618 | 6.73367 | 1.1039 | 0.8559 |
| FEV1 | Girls | 6.00 | 110 | BCPE | 2.75618 | 1.1241 | 0.11618 | 6.73367 | 1.1241 | 0.8716 |
| FEV1 | Girls | 6.00 | 111 | BCPE | 2.75618 | 1.1445 | 0.11618 | 6.73367 | 1.1445 | 0.8874 |
| FEV1 | Girls | 6.00 | 112 | BCPE | 2.75618 | 1.1650 | 0.11618 | 6.73367 | 1.1650 | 0.9034 |
| FEV1 | Girls | 6.00 | 113 | BCPE | 2.75618 | 1.1858 | 0.11618 | 6.73367 | 1.1858 | 0.9194 |
| FEV1 | Girls | 6.00 | 114 | BCPE | 2.75618 | 1.2067 | 0.11618 | 6.73367 | 1.2067 | 0.9357 |
| FEV1 | Girls | 6.00 | 115 | BCPE | 2.75618 | 1.2278 | 0.11618 | 6.73367 | 1.2278 | 0.9520 |
| FEV1 | Girls | 6.00 | 116 | BCPE | 2.75618 | 1.2491 | 0.11618 | 6.73367 | 1.2491 | 0.9686 |
| FEV1 | Girls | 6.00 | 117 | BCPE | 2.75618 | 1.2706 | 0.11618 | 6.73367 | 1.2706 | 0.9852 |
| FEV1 | Girls | 6.00 | 118 | BCPE | 2.75618 | 1.2923 | 0.11618 | 6.73367 | 1.2923 | 1.0020 |
| FEV1 | Girls | 6.00 | 119 | BCPE | 2.75618 | 1.3141 | 0.11618 | 6.73367 | 1.3141 | 1.0189 |
| FEV1 | Girls | 6.00 | 120 | BCPE | 2.75618 | 1.3361 | 0.11618 | 6.73367 | 1.3361 | 1.0360 |
| FEV1 | Girls | 6.00 | 121 | BCPE | 2.75618 | 1.3583 | 0.11618 | 6.73367 | 1.3583 | 1.0532 |
| FEV1 | Girls | 6.00 | 122 | BCPE | 2.75618 | 1.3807 | 0.11618 | 6.73367 | 1.3807 | 1.0706 |
| FEV1 | Girls | 6.00 | 123 | BCPE | 2.75618 | 1.4033 | 0.11618 | 6.73367 | 1.4033 | 1.0881 |
| FEV1 | Girls | 6.00 | 124 | BCPE | 2.75618 | 1.4260 | 0.11618 | 6.73367 | 1.4260 | 1.1057 |

**Table A2. Sex-specific LMS parameters, predicted values, and lower limits of normal (LLN) for FVC across the Phase 1 age and height range.**

| **Outcome** | **Sex** | **Age** | **Height (cm)** | **Family** | **L** | **M** | **S** | **K** | **Pred** | **LLN** |
| --- | --- | --- | --- | --- | --- | --- | --- | --- | --- | --- |
| FVC | Boys | 3.00 | 79 | BCPE | 1.57011 | 0.5192 | 0.12911 | 4.22339 | 0.5192 | 0.4046 |
| FVC | Boys | 3.00 | 80 | BCPE | 1.57011 | 0.5355 | 0.12911 | 4.22339 | 0.5355 | 0.4173 |
| FVC | Boys | 3.00 | 81 | BCPE | 1.57011 | 0.5520 | 0.12911 | 4.22339 | 0.5520 | 0.4302 |
| FVC | Boys | 3.00 | 82 | BCPE | 1.57011 | 0.5689 | 0.12911 | 4.22339 | 0.5689 | 0.4434 |
| FVC | Boys | 3.00 | 83 | BCPE | 1.57011 | 0.5861 | 0.12911 | 4.22339 | 0.5861 | 0.4568 |
| FVC | Boys | 3.00 | 84 | BCPE | 1.57011 | 0.6036 | 0.12911 | 4.22339 | 0.6036 | 0.4704 |
| FVC | Boys | 3.00 | 85 | BCPE | 1.57011 | 0.6214 | 0.12911 | 4.22339 | 0.6214 | 0.4843 |
| FVC | Boys | 3.00 | 86 | BCPE | 1.57011 | 0.6395 | 0.12911 | 4.22339 | 0.6395 | 0.4984 |
| FVC | Boys | 3.00 | 87 | BCPE | 1.57011 | 0.6579 | 0.12911 | 4.22339 | 0.6579 | 0.5127 |
| FVC | Boys | 3.00 | 88 | BCPE | 1.57011 | 0.6766 | 0.12911 | 4.22339 | 0.6766 | 0.5273 |
| FVC | Boys | 3.00 | 89 | BCPE | 1.57011 | 0.6956 | 0.12911 | 4.22339 | 0.6956 | 0.5422 |
| FVC | Boys | 3.00 | 90 | BCPE | 1.57011 | 0.7150 | 0.12911 | 4.22339 | 0.7150 | 0.5572 |
| FVC | Boys | 3.00 | 91 | BCPE | 1.57011 | 0.7346 | 0.12911 | 4.22339 | 0.7346 | 0.5726 |
| FVC | Boys | 3.00 | 92 | BCPE | 1.57011 | 0.7546 | 0.12911 | 4.22339 | 0.7546 | 0.5881 |
| FVC | Boys | 3.00 | 93 | BCPE | 1.57011 | 0.7749 | 0.12911 | 4.22339 | 0.7749 | 0.6040 |
| FVC | Boys | 3.00 | 94 | BCPE | 1.57011 | 0.7955 | 0.12911 | 4.22339 | 0.7955 | 0.6200 |
| FVC | Boys | 3.00 | 95 | BCPE | 1.57011 | 0.8165 | 0.12911 | 4.22339 | 0.8165 | 0.6363 |
| FVC | Boys | 3.00 | 96 | BCPE | 1.57011 | 0.8377 | 0.12911 | 4.22339 | 0.8377 | 0.6529 |
| FVC | Boys | 3.00 | 97 | BCPE | 1.57011 | 0.8593 | 0.12911 | 4.22339 | 0.8593 | 0.6697 |
| FVC | Boys | 3.00 | 98 | BCPE | 1.57011 | 0.8812 | 0.12911 | 4.22339 | 0.8812 | 0.6868 |
| FVC | Boys | 3.00 | 99 | BCPE | 1.57011 | 0.9034 | 0.12911 | 4.22339 | 0.9034 | 0.7041 |
| FVC | Boys | 3.00 | 100 | BCPE | 1.57011 | 0.9260 | 0.12911 | 4.22339 | 0.9260 | 0.7217 |
| FVC | Boys | 3.00 | 101 | BCPE | 1.57011 | 0.9489 | 0.12911 | 4.22339 | 0.9489 | 0.7396 |
| FVC | Boys | 3.00 | 102 | BCPE | 1.57011 | 0.9721 | 0.12911 | 4.22339 | 0.9721 | 0.7577 |
| FVC | Boys | 3.00 | 103 | BCPE | 1.57011 | 0.9957 | 0.12911 | 4.22339 | 0.9957 | 0.7760 |
| FVC | Boys | 3.00 | 104 | BCPE | 1.57011 | 1.0196 | 0.12911 | 4.22339 | 1.0196 | 0.7947 |
| FVC | Boys | 3.00 | 105 | BCPE | 1.57011 | 1.0438 | 0.12911 | 4.22339 | 1.0438 | 0.8136 |
| FVC | Boys | 3.00 | 106 | BCPE | 1.57011 | 1.0684 | 0.12911 | 4.22339 | 1.0684 | 0.8327 |
| FVC | Boys | 3.00 | 107 | BCPE | 1.57011 | 1.0933 | 0.12911 | 4.22339 | 1.0933 | 0.8521 |
| FVC | Boys | 3.00 | 108 | BCPE | 1.57011 | 1.1186 | 0.12911 | 4.22339 | 1.1186 | 0.8718 |
| FVC | Boys | 3.00 | 109 | BCPE | 1.57011 | 1.1442 | 0.12911 | 4.22339 | 1.1442 | 0.8918 |
| FVC | Boys | 3.00 | 110 | BCPE | 1.57011 | 1.1701 | 0.12911 | 4.22339 | 1.1701 | 0.9120 |
| FVC | Boys | 3.00 | 111 | BCPE | 1.57011 | 1.1964 | 0.12911 | 4.22339 | 1.1964 | 0.9325 |
| FVC | Boys | 3.00 | 112 | BCPE | 1.57011 | 1.2230 | 0.12911 | 4.22339 | 1.2230 | 0.9532 |
| FVC | Boys | 3.00 | 113 | BCPE | 1.57011 | 1.2500 | 0.12911 | 4.22339 | 1.2500 | 0.9743 |
| FVC | Boys | 3.00 | 114 | BCPE | 1.57011 | 1.2774 | 0.12911 | 4.22339 | 1.2774 | 0.9956 |
| FVC | Boys | 3.00 | 115 | BCPE | 1.57011 | 1.3050 | 0.12911 | 4.22339 | 1.3050 | 1.0171 |
| FVC | Boys | 3.00 | 116 | BCPE | 1.57011 | 1.3331 | 0.12911 | 4.22339 | 1.3331 | 1.0390 |
| FVC | Boys | 3.00 | 117 | BCPE | 1.57011 | 1.3615 | 0.12911 | 4.22339 | 1.3615 | 1.0611 |
| FVC | Boys | 3.00 | 118 | BCPE | 1.57011 | 1.3902 | 0.12911 | 4.22339 | 1.3902 | 1.0835 |
| FVC | Boys | 3.00 | 119 | BCPE | 1.57011 | 1.4193 | 0.12911 | 4.22339 | 1.4193 | 1.1062 |
| FVC | Boys | 3.00 | 120 | BCPE | 1.57011 | 1.4488 | 0.12911 | 4.22339 | 1.4488 | 1.1292 |
| FVC | Boys | 3.00 | 121 | BCPE | 1.57011 | 1.4786 | 0.12911 | 4.22339 | 1.4786 | 1.1524 |
| FVC | Boys | 3.00 | 122 | BCPE | 1.57011 | 1.5088 | 0.12911 | 4.22339 | 1.5088 | 1.1759 |
| FVC | Boys | 3.00 | 123 | BCPE | 1.57011 | 1.5393 | 0.12911 | 4.22339 | 1.5393 | 1.1997 |
| FVC | Boys | 3.00 | 124 | BCPE | 1.57011 | 1.5702 | 0.12911 | 4.22339 | 1.5702 | 1.2238 |
| FVC | Boys | 3.00 | 125 | BCPE | 1.57011 | 1.6015 | 0.12911 | 4.22339 | 1.6015 | 1.2482 |
| FVC | Boys | 3.00 | 126 | BCPE | 1.57011 | 1.6331 | 0.12911 | 4.22339 | 1.6331 | 1.2728 |
| FVC | Boys | 3.00 | 127 | BCPE | 1.57011 | 1.6651 | 0.12911 | 4.22339 | 1.6651 | 1.2978 |
| FVC | Boys | 3.00 | 128 | BCPE | 1.57011 | 1.6975 | 0.12911 | 4.22339 | 1.6975 | 1.3230 |
| FVC | Boys | 3.00 | 129 | BCPE | 1.57011 | 1.7302 | 0.12911 | 4.22339 | 1.7302 | 1.3485 |
| FVC | Boys | 3.00 | 130 | BCPE | 1.57011 | 1.7633 | 0.12911 | 4.22339 | 1.7633 | 1.3743 |
| FVC | Boys | 3.00 | 131 | BCPE | 1.57011 | 1.7968 | 0.12911 | 4.22339 | 1.7968 | 1.4004 |
| FVC | Boys | 3.00 | 132 | BCPE | 1.57011 | 1.8307 | 0.12911 | 4.22339 | 1.8307 | 1.4268 |
| FVC | Boys | 3.25 | 79 | BCPE | 1.45515 | 0.5209 | 0.12891 | 4.36676 | 0.5209 | 0.4080 |
| FVC | Boys | 3.25 | 80 | BCPE | 1.45515 | 0.5373 | 0.12891 | 4.36676 | 0.5373 | 0.4208 |
| FVC | Boys | 3.25 | 81 | BCPE | 1.45515 | 0.5539 | 0.12891 | 4.36676 | 0.5539 | 0.4338 |
| FVC | Boys | 3.25 | 82 | BCPE | 1.45515 | 0.5708 | 0.12891 | 4.36676 | 0.5708 | 0.4471 |
| FVC | Boys | 3.25 | 83 | BCPE | 1.45515 | 0.5881 | 0.12891 | 4.36676 | 0.5881 | 0.4606 |
| FVC | Boys | 3.25 | 84 | BCPE | 1.45515 | 0.6056 | 0.12891 | 4.36676 | 0.6056 | 0.4743 |
| FVC | Boys | 3.25 | 85 | BCPE | 1.45515 | 0.6235 | 0.12891 | 4.36676 | 0.6235 | 0.4883 |
| FVC | Boys | 3.25 | 86 | BCPE | 1.45515 | 0.6416 | 0.12891 | 4.36676 | 0.6416 | 0.5025 |
| FVC | Boys | 3.25 | 87 | BCPE | 1.45515 | 0.6601 | 0.12891 | 4.36676 | 0.6601 | 0.5170 |
| FVC | Boys | 3.25 | 88 | BCPE | 1.45515 | 0.6789 | 0.12891 | 4.36676 | 0.6789 | 0.5317 |
| FVC | Boys | 3.25 | 89 | BCPE | 1.45515 | 0.6980 | 0.12891 | 4.36676 | 0.6980 | 0.5466 |
| FVC | Boys | 3.25 | 90 | BCPE | 1.45515 | 0.7174 | 0.12891 | 4.36676 | 0.7174 | 0.5618 |
| FVC | Boys | 3.25 | 91 | BCPE | 1.45515 | 0.7371 | 0.12891 | 4.36676 | 0.7371 | 0.5773 |
| FVC | Boys | 3.25 | 92 | BCPE | 1.45515 | 0.7572 | 0.12891 | 4.36676 | 0.7572 | 0.5930 |
| FVC | Boys | 3.25 | 93 | BCPE | 1.45515 | 0.7775 | 0.12891 | 4.36676 | 0.7775 | 0.6089 |
| FVC | Boys | 3.25 | 94 | BCPE | 1.45515 | 0.7982 | 0.12891 | 4.36676 | 0.7982 | 0.6251 |
| FVC | Boys | 3.25 | 95 | BCPE | 1.45515 | 0.8192 | 0.12891 | 4.36676 | 0.8192 | 0.6416 |
| FVC | Boys | 3.25 | 96 | BCPE | 1.45515 | 0.8405 | 0.12891 | 4.36676 | 0.8405 | 0.6583 |
| FVC | Boys | 3.25 | 97 | BCPE | 1.45515 | 0.8622 | 0.12891 | 4.36676 | 0.8622 | 0.6752 |
| FVC | Boys | 3.25 | 98 | BCPE | 1.45515 | 0.8842 | 0.12891 | 4.36676 | 0.8842 | 0.6925 |
| FVC | Boys | 3.25 | 99 | BCPE | 1.45515 | 0.9065 | 0.12891 | 4.36676 | 0.9065 | 0.7099 |
| FVC | Boys | 3.25 | 100 | BCPE | 1.45515 | 0.9291 | 0.12891 | 4.36676 | 0.9291 | 0.7277 |
| FVC | Boys | 3.25 | 101 | BCPE | 1.45515 | 0.9521 | 0.12891 | 4.36676 | 0.9521 | 0.7457 |
| FVC | Boys | 3.25 | 102 | BCPE | 1.45515 | 0.9754 | 0.12891 | 4.36676 | 0.9754 | 0.7639 |
| FVC | Boys | 3.25 | 103 | BCPE | 1.45515 | 0.9991 | 0.12891 | 4.36676 | 0.9991 | 0.7824 |
| FVC | Boys | 3.25 | 104 | BCPE | 1.45515 | 1.0230 | 0.12891 | 4.36676 | 1.0230 | 0.8012 |
| FVC | Boys | 3.25 | 105 | BCPE | 1.45515 | 1.0474 | 0.12891 | 4.36676 | 1.0474 | 0.8203 |
| FVC | Boys | 3.25 | 106 | BCPE | 1.45515 | 1.0720 | 0.12891 | 4.36676 | 1.0720 | 0.8396 |
| FVC | Boys | 3.25 | 107 | BCPE | 1.45515 | 1.0970 | 0.12891 | 4.36676 | 1.0970 | 0.8591 |
| FVC | Boys | 3.25 | 108 | BCPE | 1.45515 | 1.1224 | 0.12891 | 4.36676 | 1.1224 | 0.8790 |
| FVC | Boys | 3.25 | 109 | BCPE | 1.45515 | 1.1480 | 0.12891 | 4.36676 | 1.1480 | 0.8991 |
| FVC | Boys | 3.25 | 110 | BCPE | 1.45515 | 1.1741 | 0.12891 | 4.36676 | 1.1741 | 0.9195 |
| FVC | Boys | 3.25 | 111 | BCPE | 1.45515 | 1.2004 | 0.12891 | 4.36676 | 1.2004 | 0.9401 |
| FVC | Boys | 3.25 | 112 | BCPE | 1.45515 | 1.2272 | 0.12891 | 4.36676 | 1.2272 | 0.9611 |
| FVC | Boys | 3.25 | 113 | BCPE | 1.45515 | 1.2542 | 0.12891 | 4.36676 | 1.2542 | 0.9823 |
| FVC | Boys | 3.25 | 114 | BCPE | 1.45515 | 1.2817 | 0.12891 | 4.36676 | 1.2817 | 1.0038 |
| FVC | Boys | 3.25 | 115 | BCPE | 1.45515 | 1.3094 | 0.12891 | 4.36676 | 1.3094 | 1.0255 |
| FVC | Boys | 3.25 | 116 | BCPE | 1.45515 | 1.3376 | 0.12891 | 4.36676 | 1.3376 | 1.0475 |
| FVC | Boys | 3.25 | 117 | BCPE | 1.45515 | 1.3661 | 0.12891 | 4.36676 | 1.3661 | 1.0698 |
| FVC | Boys | 3.25 | 118 | BCPE | 1.45515 | 1.3949 | 0.12891 | 4.36676 | 1.3949 | 1.0924 |
| FVC | Boys | 3.25 | 119 | BCPE | 1.45515 | 1.4241 | 0.12891 | 4.36676 | 1.4241 | 1.1153 |
| FVC | Boys | 3.25 | 120 | BCPE | 1.45515 | 1.4536 | 0.12891 | 4.36676 | 1.4536 | 1.1384 |
| FVC | Boys | 3.25 | 121 | BCPE | 1.45515 | 1.4836 | 0.12891 | 4.36676 | 1.4836 | 1.1619 |
| FVC | Boys | 3.25 | 122 | BCPE | 1.45515 | 1.5138 | 0.12891 | 4.36676 | 1.5138 | 1.1856 |
| FVC | Boys | 3.25 | 123 | BCPE | 1.45515 | 1.5445 | 0.12891 | 4.36676 | 1.5445 | 1.2096 |
| FVC | Boys | 3.25 | 124 | BCPE | 1.45515 | 1.5755 | 0.12891 | 4.36676 | 1.5755 | 1.2339 |
| FVC | Boys | 3.25 | 125 | BCPE | 1.45515 | 1.6069 | 0.12891 | 4.36676 | 1.6069 | 1.2584 |
| FVC | Boys | 3.25 | 126 | BCPE | 1.45515 | 1.6386 | 0.12891 | 4.36676 | 1.6386 | 1.2833 |
| FVC | Boys | 3.25 | 127 | BCPE | 1.45515 | 1.6707 | 0.12891 | 4.36676 | 1.6707 | 1.3084 |
| FVC | Boys | 3.25 | 128 | BCPE | 1.45515 | 1.7032 | 0.12891 | 4.36676 | 1.7032 | 1.3339 |
| FVC | Boys | 3.25 | 129 | BCPE | 1.45515 | 1.7361 | 0.12891 | 4.36676 | 1.7361 | 1.3596 |
| FVC | Boys | 3.25 | 130 | BCPE | 1.45515 | 1.7693 | 0.12891 | 4.36676 | 1.7693 | 1.3856 |
| FVC | Boys | 3.25 | 131 | BCPE | 1.45515 | 1.8029 | 0.12891 | 4.36676 | 1.8029 | 1.4119 |
| FVC | Boys | 3.25 | 132 | BCPE | 1.45515 | 1.8368 | 0.12891 | 4.36676 | 1.8368 | 1.4385 |
| FVC | Boys | 3.50 | 79 | BCPE | 1.34872 | 0.5226 | 0.12872 | 4.50383 | 0.5226 | 0.4110 |
| FVC | Boys | 3.50 | 80 | BCPE | 1.34872 | 0.5390 | 0.12872 | 4.50383 | 0.5390 | 0.4239 |
| FVC | Boys | 3.50 | 81 | BCPE | 1.34872 | 0.5556 | 0.12872 | 4.50383 | 0.5556 | 0.4370 |
| FVC | Boys | 3.50 | 82 | BCPE | 1.34872 | 0.5726 | 0.12872 | 4.50383 | 0.5726 | 0.4504 |
| FVC | Boys | 3.50 | 83 | BCPE | 1.34872 | 0.5899 | 0.12872 | 4.50383 | 0.5899 | 0.4640 |
| FVC | Boys | 3.50 | 84 | BCPE | 1.34872 | 0.6075 | 0.12872 | 4.50383 | 0.6075 | 0.4778 |
| FVC | Boys | 3.50 | 85 | BCPE | 1.34872 | 0.6254 | 0.12872 | 4.50383 | 0.6254 | 0.4919 |
| FVC | Boys | 3.50 | 86 | BCPE | 1.34872 | 0.6437 | 0.12872 | 4.50383 | 0.6437 | 0.5062 |
| FVC | Boys | 3.50 | 87 | BCPE | 1.34872 | 0.6622 | 0.12872 | 4.50383 | 0.6622 | 0.5208 |
| FVC | Boys | 3.50 | 88 | BCPE | 1.34872 | 0.6810 | 0.12872 | 4.50383 | 0.6810 | 0.5356 |
| FVC | Boys | 3.50 | 89 | BCPE | 1.34872 | 0.7002 | 0.12872 | 4.50383 | 0.7002 | 0.5507 |
| FVC | Boys | 3.50 | 90 | BCPE | 1.34872 | 0.7197 | 0.12872 | 4.50383 | 0.7197 | 0.5660 |
| FVC | Boys | 3.50 | 91 | BCPE | 1.34872 | 0.7394 | 0.12872 | 4.50383 | 0.7394 | 0.5816 |
| FVC | Boys | 3.50 | 92 | BCPE | 1.34872 | 0.7596 | 0.12872 | 4.50383 | 0.7596 | 0.5974 |
| FVC | Boys | 3.50 | 93 | BCPE | 1.34872 | 0.7800 | 0.12872 | 4.50383 | 0.7800 | 0.6135 |
| FVC | Boys | 3.50 | 94 | BCPE | 1.34872 | 0.8007 | 0.12872 | 4.50383 | 0.8007 | 0.6298 |
| FVC | Boys | 3.50 | 95 | BCPE | 1.34872 | 0.8218 | 0.12872 | 4.50383 | 0.8218 | 0.6464 |
| FVC | Boys | 3.50 | 96 | BCPE | 1.34872 | 0.8432 | 0.12872 | 4.50383 | 0.8432 | 0.6632 |
| FVC | Boys | 3.50 | 97 | BCPE | 1.34872 | 0.8649 | 0.12872 | 4.50383 | 0.8649 | 0.6803 |
| FVC | Boys | 3.50 | 98 | BCPE | 1.34872 | 0.8870 | 0.12872 | 4.50383 | 0.8870 | 0.6976 |
| FVC | Boys | 3.50 | 99 | BCPE | 1.34872 | 0.9094 | 0.12872 | 4.50383 | 0.9094 | 0.7152 |
| FVC | Boys | 3.50 | 100 | BCPE | 1.34872 | 0.9321 | 0.12872 | 4.50383 | 0.9321 | 0.7331 |
| FVC | Boys | 3.50 | 101 | BCPE | 1.34872 | 0.9551 | 0.12872 | 4.50383 | 0.9551 | 0.7512 |
| FVC | Boys | 3.50 | 102 | BCPE | 1.34872 | 0.9785 | 0.12872 | 4.50383 | 0.9785 | 0.7696 |
| FVC | Boys | 3.50 | 103 | BCPE | 1.34872 | 1.0022 | 0.12872 | 4.50383 | 1.0022 | 0.7883 |
| FVC | Boys | 3.50 | 104 | BCPE | 1.34872 | 1.0263 | 0.12872 | 4.50383 | 1.0263 | 0.8072 |
| FVC | Boys | 3.50 | 105 | BCPE | 1.34872 | 1.0507 | 0.12872 | 4.50383 | 1.0507 | 0.8264 |
| FVC | Boys | 3.50 | 106 | BCPE | 1.34872 | 1.0754 | 0.12872 | 4.50383 | 1.0754 | 0.8458 |
| FVC | Boys | 3.50 | 107 | BCPE | 1.34872 | 1.1005 | 0.12872 | 4.50383 | 1.1005 | 0.8655 |
| FVC | Boys | 3.50 | 108 | BCPE | 1.34872 | 1.1259 | 0.12872 | 4.50383 | 1.1259 | 0.8855 |
| FVC | Boys | 3.50 | 109 | BCPE | 1.34872 | 1.1517 | 0.12872 | 4.50383 | 1.1517 | 0.9058 |
| FVC | Boys | 3.50 | 110 | BCPE | 1.34872 | 1.1778 | 0.12872 | 4.50383 | 1.1778 | 0.9263 |
| FVC | Boys | 3.50 | 111 | BCPE | 1.34872 | 1.2042 | 0.12872 | 4.50383 | 1.2042 | 0.9471 |
| FVC | Boys | 3.50 | 112 | BCPE | 1.34872 | 1.2311 | 0.12872 | 4.50383 | 1.2311 | 0.9682 |
| FVC | Boys | 3.50 | 113 | BCPE | 1.34872 | 1.2582 | 0.12872 | 4.50383 | 1.2582 | 0.9896 |
| FVC | Boys | 3.50 | 114 | BCPE | 1.34872 | 1.2857 | 0.12872 | 4.50383 | 1.2857 | 1.0112 |
| FVC | Boys | 3.50 | 115 | BCPE | 1.34872 | 1.3136 | 0.12872 | 4.50383 | 1.3136 | 1.0331 |
| FVC | Boys | 3.50 | 116 | BCPE | 1.34872 | 1.3418 | 0.12872 | 4.50383 | 1.3418 | 1.0553 |
| FVC | Boys | 3.50 | 117 | BCPE | 1.34872 | 1.3704 | 0.12872 | 4.50383 | 1.3704 | 1.0778 |
| FVC | Boys | 3.50 | 118 | BCPE | 1.34872 | 1.3993 | 0.12872 | 4.50383 | 1.3993 | 1.1006 |
| FVC | Boys | 3.50 | 119 | BCPE | 1.34872 | 1.4286 | 0.12872 | 4.50383 | 1.4286 | 1.1236 |
| FVC | Boys | 3.50 | 120 | BCPE | 1.34872 | 1.4582 | 0.12872 | 4.50383 | 1.4582 | 1.1469 |
| FVC | Boys | 3.50 | 121 | BCPE | 1.34872 | 1.4883 | 0.12872 | 4.50383 | 1.4883 | 1.1705 |
| FVC | Boys | 3.50 | 122 | BCPE | 1.34872 | 1.5186 | 0.12872 | 4.50383 | 1.5186 | 1.1944 |
| FVC | Boys | 3.50 | 123 | BCPE | 1.34872 | 1.5494 | 0.12872 | 4.50383 | 1.5494 | 1.2186 |
| FVC | Boys | 3.50 | 124 | BCPE | 1.34872 | 1.5805 | 0.12872 | 4.50383 | 1.5805 | 1.2431 |
| FVC | Boys | 3.50 | 125 | BCPE | 1.34872 | 1.6120 | 0.12872 | 4.50383 | 1.6120 | 1.2678 |
| FVC | Boys | 3.50 | 126 | BCPE | 1.34872 | 1.6438 | 0.12872 | 4.50383 | 1.6438 | 1.2929 |
| FVC | Boys | 3.50 | 127 | BCPE | 1.34872 | 1.6760 | 0.12872 | 4.50383 | 1.6760 | 1.3182 |
| FVC | Boys | 3.50 | 128 | BCPE | 1.34872 | 1.7086 | 0.12872 | 4.50383 | 1.7086 | 1.3438 |
| FVC | Boys | 3.50 | 129 | BCPE | 1.34872 | 1.7415 | 0.12872 | 4.50383 | 1.7415 | 1.3697 |
| FVC | Boys | 3.50 | 130 | BCPE | 1.34872 | 1.7749 | 0.12872 | 4.50383 | 1.7749 | 1.3959 |
| FVC | Boys | 3.50 | 131 | BCPE | 1.34872 | 1.8086 | 0.12872 | 4.50383 | 1.8086 | 1.4224 |
| FVC | Boys | 3.50 | 132 | BCPE | 1.34872 | 1.8427 | 0.12872 | 4.50383 | 1.8427 | 1.4493 |
| FVC | Boys | 3.75 | 79 | BCPE | 1.24963 | 0.5241 | 0.12855 | 4.63531 | 0.5241 | 0.4138 |
| FVC | Boys | 3.75 | 80 | BCPE | 1.24963 | 0.5406 | 0.12855 | 4.63531 | 0.5406 | 0.4268 |
| FVC | Boys | 3.75 | 81 | BCPE | 1.24963 | 0.5573 | 0.12855 | 4.63531 | 0.5573 | 0.4400 |
| FVC | Boys | 3.75 | 82 | BCPE | 1.24963 | 0.5744 | 0.12855 | 4.63531 | 0.5744 | 0.4534 |
| FVC | Boys | 3.75 | 83 | BCPE | 1.24963 | 0.5917 | 0.12855 | 4.63531 | 0.5917 | 0.4671 |
| FVC | Boys | 3.75 | 84 | BCPE | 1.24963 | 0.6094 | 0.12855 | 4.63531 | 0.6094 | 0.4811 |
| FVC | Boys | 3.75 | 85 | BCPE | 1.24963 | 0.6273 | 0.12855 | 4.63531 | 0.6273 | 0.4953 |
| FVC | Boys | 3.75 | 86 | BCPE | 1.24963 | 0.6456 | 0.12855 | 4.63531 | 0.6456 | 0.5097 |
| FVC | Boys | 3.75 | 87 | BCPE | 1.24963 | 0.6642 | 0.12855 | 4.63531 | 0.6642 | 0.5244 |
| FVC | Boys | 3.75 | 88 | BCPE | 1.24963 | 0.6831 | 0.12855 | 4.63531 | 0.6831 | 0.5393 |
| FVC | Boys | 3.75 | 89 | BCPE | 1.24963 | 0.7023 | 0.12855 | 4.63531 | 0.7023 | 0.5544 |
| FVC | Boys | 3.75 | 90 | BCPE | 1.24963 | 0.7218 | 0.12855 | 4.63531 | 0.7218 | 0.5699 |
| FVC | Boys | 3.75 | 91 | BCPE | 1.24963 | 0.7417 | 0.12855 | 4.63531 | 0.7417 | 0.5855 |
| FVC | Boys | 3.75 | 92 | BCPE | 1.24963 | 0.7618 | 0.12855 | 4.63531 | 0.7618 | 0.6015 |
| FVC | Boys | 3.75 | 93 | BCPE | 1.24963 | 0.7823 | 0.12855 | 4.63531 | 0.7823 | 0.6176 |
| FVC | Boys | 3.75 | 94 | BCPE | 1.24963 | 0.8031 | 0.12855 | 4.63531 | 0.8031 | 0.6341 |
| FVC | Boys | 3.75 | 95 | BCPE | 1.24963 | 0.8243 | 0.12855 | 4.63531 | 0.8243 | 0.6508 |
| FVC | Boys | 3.75 | 96 | BCPE | 1.24963 | 0.8457 | 0.12855 | 4.63531 | 0.8457 | 0.6677 |
| FVC | Boys | 3.75 | 97 | BCPE | 1.24963 | 0.8675 | 0.12855 | 4.63531 | 0.8675 | 0.6849 |
| FVC | Boys | 3.75 | 98 | BCPE | 1.24963 | 0.8896 | 0.12855 | 4.63531 | 0.8896 | 0.7024 |
| FVC | Boys | 3.75 | 99 | BCPE | 1.24963 | 0.9121 | 0.12855 | 4.63531 | 0.9121 | 0.7201 |
| FVC | Boys | 3.75 | 100 | BCPE | 1.24963 | 0.9349 | 0.12855 | 4.63531 | 0.9349 | 0.7381 |
| FVC | Boys | 3.75 | 101 | BCPE | 1.24963 | 0.9580 | 0.12855 | 4.63531 | 0.9580 | 0.7563 |
| FVC | Boys | 3.75 | 102 | BCPE | 1.24963 | 0.9814 | 0.12855 | 4.63531 | 0.9814 | 0.7748 |
| FVC | Boys | 3.75 | 103 | BCPE | 1.24963 | 1.0052 | 0.12855 | 4.63531 | 1.0052 | 0.7936 |
| FVC | Boys | 3.75 | 104 | BCPE | 1.24963 | 1.0294 | 0.12855 | 4.63531 | 1.0294 | 0.8127 |
| FVC | Boys | 3.75 | 105 | BCPE | 1.24963 | 1.0538 | 0.12855 | 4.63531 | 1.0538 | 0.8320 |
| FVC | Boys | 3.75 | 106 | BCPE | 1.24963 | 1.0786 | 0.12855 | 4.63531 | 1.0786 | 0.8516 |
| FVC | Boys | 3.75 | 107 | BCPE | 1.24963 | 1.1038 | 0.12855 | 4.63531 | 1.1038 | 0.8714 |
| FVC | Boys | 3.75 | 108 | BCPE | 1.24963 | 1.1293 | 0.12855 | 4.63531 | 1.1293 | 0.8916 |
| FVC | Boys | 3.75 | 109 | BCPE | 1.24963 | 1.1551 | 0.12855 | 4.63531 | 1.1551 | 0.9120 |
| FVC | Boys | 3.75 | 110 | BCPE | 1.24963 | 1.1813 | 0.12855 | 4.63531 | 1.1813 | 0.9326 |
| FVC | Boys | 3.75 | 111 | BCPE | 1.24963 | 1.2078 | 0.12855 | 4.63531 | 1.2078 | 0.9536 |
| FVC | Boys | 3.75 | 112 | BCPE | 1.24963 | 1.2347 | 0.12855 | 4.63531 | 1.2347 | 0.9748 |
| FVC | Boys | 3.75 | 113 | BCPE | 1.24963 | 1.2620 | 0.12855 | 4.63531 | 1.2620 | 0.9963 |
| FVC | Boys | 3.75 | 114 | BCPE | 1.24963 | 1.2896 | 0.12855 | 4.63531 | 1.2896 | 1.0181 |
| FVC | Boys | 3.75 | 115 | BCPE | 1.24963 | 1.3175 | 0.12855 | 4.63531 | 1.3175 | 1.0402 |
| FVC | Boys | 3.75 | 116 | BCPE | 1.24963 | 1.3458 | 0.12855 | 4.63531 | 1.3458 | 1.0625 |
| FVC | Boys | 3.75 | 117 | BCPE | 1.24963 | 1.3745 | 0.12855 | 4.63531 | 1.3745 | 1.0851 |
| FVC | Boys | 3.75 | 118 | BCPE | 1.24963 | 1.4035 | 0.12855 | 4.63531 | 1.4035 | 1.1080 |
| FVC | Boys | 3.75 | 119 | BCPE | 1.24963 | 1.4329 | 0.12855 | 4.63531 | 1.4329 | 1.1312 |
| FVC | Boys | 3.75 | 120 | BCPE | 1.24963 | 1.4626 | 0.12855 | 4.63531 | 1.4626 | 1.1547 |
| FVC | Boys | 3.75 | 121 | BCPE | 1.24963 | 1.4927 | 0.12855 | 4.63531 | 1.4927 | 1.1785 |
| FVC | Boys | 3.75 | 122 | BCPE | 1.24963 | 1.5232 | 0.12855 | 4.63531 | 1.5232 | 1.2025 |
| FVC | Boys | 3.75 | 123 | BCPE | 1.24963 | 1.5540 | 0.12855 | 4.63531 | 1.5540 | 1.2269 |
| FVC | Boys | 3.75 | 124 | BCPE | 1.24963 | 1.5852 | 0.12855 | 4.63531 | 1.5852 | 1.2515 |
| FVC | Boys | 3.75 | 125 | BCPE | 1.24963 | 1.6168 | 0.12855 | 4.63531 | 1.6168 | 1.2764 |
| FVC | Boys | 3.75 | 126 | BCPE | 1.24963 | 1.6487 | 0.12855 | 4.63531 | 1.6487 | 1.3016 |
| FVC | Boys | 3.75 | 127 | BCPE | 1.24963 | 1.6810 | 0.12855 | 4.63531 | 1.6810 | 1.3272 |
| FVC | Boys | 3.75 | 128 | BCPE | 1.24963 | 1.7137 | 0.12855 | 4.63531 | 1.7137 | 1.3530 |
| FVC | Boys | 3.75 | 129 | BCPE | 1.24963 | 1.7467 | 0.12855 | 4.63531 | 1.7467 | 1.3790 |
| FVC | Boys | 3.75 | 130 | BCPE | 1.24963 | 1.7802 | 0.12855 | 4.63531 | 1.7802 | 1.4054 |
| FVC | Boys | 3.75 | 131 | BCPE | 1.24963 | 1.8140 | 0.12855 | 4.63531 | 1.8140 | 1.4321 |
| FVC | Boys | 3.75 | 132 | BCPE | 1.24963 | 1.8482 | 0.12855 | 4.63531 | 1.8482 | 1.4591 |
| FVC | Boys | 4.00 | 79 | BCPE | 1.15694 | 0.5256 | 0.12838 | 4.76177 | 0.5256 | 0.4164 |
| FVC | Boys | 4.00 | 80 | BCPE | 1.15694 | 0.5421 | 0.12838 | 4.76177 | 0.5421 | 0.4294 |
| FVC | Boys | 4.00 | 81 | BCPE | 1.15694 | 0.5589 | 0.12838 | 4.76177 | 0.5589 | 0.4427 |
| FVC | Boys | 4.00 | 82 | BCPE | 1.15694 | 0.5760 | 0.12838 | 4.76177 | 0.5760 | 0.4563 |
| FVC | Boys | 4.00 | 83 | BCPE | 1.15694 | 0.5934 | 0.12838 | 4.76177 | 0.5934 | 0.4701 |
| FVC | Boys | 4.00 | 84 | BCPE | 1.15694 | 0.6111 | 0.12838 | 4.76177 | 0.6111 | 0.4841 |
| FVC | Boys | 4.00 | 85 | BCPE | 1.15694 | 0.6291 | 0.12838 | 4.76177 | 0.6291 | 0.4983 |
| FVC | Boys | 4.00 | 86 | BCPE | 1.15694 | 0.6474 | 0.12838 | 4.76177 | 0.6474 | 0.5129 |
| FVC | Boys | 4.00 | 87 | BCPE | 1.15694 | 0.6660 | 0.12838 | 4.76177 | 0.6660 | 0.5276 |
| FVC | Boys | 4.00 | 88 | BCPE | 1.15694 | 0.6850 | 0.12838 | 4.76177 | 0.6850 | 0.5426 |
| FVC | Boys | 4.00 | 89 | BCPE | 1.15694 | 0.7043 | 0.12838 | 4.76177 | 0.7043 | 0.5579 |
| FVC | Boys | 4.00 | 90 | BCPE | 1.15694 | 0.7238 | 0.12838 | 4.76177 | 0.7238 | 0.5734 |
| FVC | Boys | 4.00 | 91 | BCPE | 1.15694 | 0.7437 | 0.12838 | 4.76177 | 0.7437 | 0.5892 |
| FVC | Boys | 4.00 | 92 | BCPE | 1.15694 | 0.7640 | 0.12838 | 4.76177 | 0.7640 | 0.6052 |
| FVC | Boys | 4.00 | 93 | BCPE | 1.15694 | 0.7845 | 0.12838 | 4.76177 | 0.7845 | 0.6215 |
| FVC | Boys | 4.00 | 94 | BCPE | 1.15694 | 0.8054 | 0.12838 | 4.76177 | 0.8054 | 0.6380 |
| FVC | Boys | 4.00 | 95 | BCPE | 1.15694 | 0.8266 | 0.12838 | 4.76177 | 0.8266 | 0.6548 |
| FVC | Boys | 4.00 | 96 | BCPE | 1.15694 | 0.8481 | 0.12838 | 4.76177 | 0.8481 | 0.6719 |
| FVC | Boys | 4.00 | 97 | BCPE | 1.15694 | 0.8700 | 0.12838 | 4.76177 | 0.8700 | 0.6892 |
| FVC | Boys | 4.00 | 98 | BCPE | 1.15694 | 0.8921 | 0.12838 | 4.76177 | 0.8921 | 0.7067 |
| FVC | Boys | 4.00 | 99 | BCPE | 1.15694 | 0.9146 | 0.12838 | 4.76177 | 0.9146 | 0.7246 |
| FVC | Boys | 4.00 | 100 | BCPE | 1.15694 | 0.9375 | 0.12838 | 4.76177 | 0.9375 | 0.7427 |
| FVC | Boys | 4.00 | 101 | BCPE | 1.15694 | 0.9607 | 0.12838 | 4.76177 | 0.9607 | 0.7610 |
| FVC | Boys | 4.00 | 102 | BCPE | 1.15694 | 0.9842 | 0.12838 | 4.76177 | 0.9842 | 0.7797 |
| FVC | Boys | 4.00 | 103 | BCPE | 1.15694 | 1.0080 | 0.12838 | 4.76177 | 1.0080 | 0.7986 |
| FVC | Boys | 4.00 | 104 | BCPE | 1.15694 | 1.0322 | 0.12838 | 4.76177 | 1.0322 | 0.8177 |
| FVC | Boys | 4.00 | 105 | BCPE | 1.15694 | 1.0568 | 0.12838 | 4.76177 | 1.0568 | 0.8372 |
| FVC | Boys | 4.00 | 106 | BCPE | 1.15694 | 1.0817 | 0.12838 | 4.76177 | 1.0817 | 0.8569 |
| FVC | Boys | 4.00 | 107 | BCPE | 1.15694 | 1.1069 | 0.12838 | 4.76177 | 1.1069 | 0.8769 |
| FVC | Boys | 4.00 | 108 | BCPE | 1.15694 | 1.1324 | 0.12838 | 4.76177 | 1.1324 | 0.8971 |
| FVC | Boys | 4.00 | 109 | BCPE | 1.15694 | 1.1584 | 0.12838 | 4.76177 | 1.1584 | 0.9176 |
| FVC | Boys | 4.00 | 110 | BCPE | 1.15694 | 1.1846 | 0.12838 | 4.76177 | 1.1846 | 0.9385 |
| FVC | Boys | 4.00 | 111 | BCPE | 1.15694 | 1.2112 | 0.12838 | 4.76177 | 1.2112 | 0.9595 |
| FVC | Boys | 4.00 | 112 | BCPE | 1.15694 | 1.2382 | 0.12838 | 4.76177 | 1.2382 | 0.9809 |
| FVC | Boys | 4.00 | 113 | BCPE | 1.15694 | 1.2655 | 0.12838 | 4.76177 | 1.2655 | 1.0025 |
| FVC | Boys | 4.00 | 114 | BCPE | 1.15694 | 1.2932 | 0.12838 | 4.76177 | 1.2932 | 1.0245 |
| FVC | Boys | 4.00 | 115 | BCPE | 1.15694 | 1.3212 | 0.12838 | 4.76177 | 1.3212 | 1.0467 |
| FVC | Boys | 4.00 | 116 | BCPE | 1.15694 | 1.3496 | 0.12838 | 4.76177 | 1.3496 | 1.0691 |
| FVC | Boys | 4.00 | 117 | BCPE | 1.15694 | 1.3783 | 0.12838 | 4.76177 | 1.3783 | 1.0919 |
| FVC | Boys | 4.00 | 118 | BCPE | 1.15694 | 1.4074 | 0.12838 | 4.76177 | 1.4074 | 1.1150 |
| FVC | Boys | 4.00 | 119 | BCPE | 1.15694 | 1.4369 | 0.12838 | 4.76177 | 1.4369 | 1.1383 |
| FVC | Boys | 4.00 | 120 | BCPE | 1.15694 | 1.4667 | 0.12838 | 4.76177 | 1.4667 | 1.1619 |
| FVC | Boys | 4.00 | 121 | BCPE | 1.15694 | 1.4969 | 0.12838 | 4.76177 | 1.4969 | 1.1858 |
| FVC | Boys | 4.00 | 122 | BCPE | 1.15694 | 1.5275 | 0.12838 | 4.76177 | 1.5275 | 1.2100 |
| FVC | Boys | 4.00 | 123 | BCPE | 1.15694 | 1.5584 | 0.12838 | 4.76177 | 1.5584 | 1.2345 |
| FVC | Boys | 4.00 | 124 | BCPE | 1.15694 | 1.5897 | 0.12838 | 4.76177 | 1.5897 | 1.2593 |
| FVC | Boys | 4.00 | 125 | BCPE | 1.15694 | 1.6213 | 0.12838 | 4.76177 | 1.6213 | 1.2844 |
| FVC | Boys | 4.00 | 126 | BCPE | 1.15694 | 1.6533 | 0.12838 | 4.76177 | 1.6533 | 1.3098 |
| FVC | Boys | 4.00 | 127 | BCPE | 1.15694 | 1.6857 | 0.12838 | 4.76177 | 1.6857 | 1.3354 |
| FVC | Boys | 4.00 | 128 | BCPE | 1.15694 | 1.7185 | 0.12838 | 4.76177 | 1.7185 | 1.3614 |
| FVC | Boys | 4.00 | 129 | BCPE | 1.15694 | 1.7517 | 0.12838 | 4.76177 | 1.7517 | 1.3876 |
| FVC | Boys | 4.00 | 130 | BCPE | 1.15694 | 1.7852 | 0.12838 | 4.76177 | 1.7852 | 1.4142 |
| FVC | Boys | 4.00 | 131 | BCPE | 1.15694 | 1.8191 | 0.12838 | 4.76177 | 1.8191 | 1.4411 |
| FVC | Boys | 4.00 | 132 | BCPE | 1.15694 | 1.8534 | 0.12838 | 4.76177 | 1.8534 | 1.4682 |
| FVC | Boys | 4.25 | 79 | BCPE | 1.06987 | 0.5270 | 0.12823 | 4.88371 | 0.5270 | 0.4188 |
| FVC | Boys | 4.25 | 80 | BCPE | 1.06987 | 0.5435 | 0.12823 | 4.88371 | 0.5435 | 0.4319 |
| FVC | Boys | 4.25 | 81 | BCPE | 1.06987 | 0.5603 | 0.12823 | 4.88371 | 0.5603 | 0.4453 |
| FVC | Boys | 4.25 | 82 | BCPE | 1.06987 | 0.5775 | 0.12823 | 4.88371 | 0.5775 | 0.4589 |
| FVC | Boys | 4.25 | 83 | BCPE | 1.06987 | 0.5949 | 0.12823 | 4.88371 | 0.5949 | 0.4727 |
| FVC | Boys | 4.25 | 84 | BCPE | 1.06987 | 0.6127 | 0.12823 | 4.88371 | 0.6127 | 0.4869 |
| FVC | Boys | 4.25 | 85 | BCPE | 1.06987 | 0.6307 | 0.12823 | 4.88371 | 0.6307 | 0.5012 |
| FVC | Boys | 4.25 | 86 | BCPE | 1.06987 | 0.6491 | 0.12823 | 4.88371 | 0.6491 | 0.5158 |
| FVC | Boys | 4.25 | 87 | BCPE | 1.06987 | 0.6678 | 0.12823 | 4.88371 | 0.6678 | 0.5307 |
| FVC | Boys | 4.25 | 88 | BCPE | 1.06987 | 0.6868 | 0.12823 | 4.88371 | 0.6868 | 0.5458 |
| FVC | Boys | 4.25 | 89 | BCPE | 1.06987 | 0.7061 | 0.12823 | 4.88371 | 0.7061 | 0.5611 |
| FVC | Boys | 4.25 | 90 | BCPE | 1.06987 | 0.7257 | 0.12823 | 4.88371 | 0.7257 | 0.5767 |
| FVC | Boys | 4.25 | 91 | BCPE | 1.06987 | 0.7457 | 0.12823 | 4.88371 | 0.7457 | 0.5926 |
| FVC | Boys | 4.25 | 92 | BCPE | 1.06987 | 0.7660 | 0.12823 | 4.88371 | 0.7660 | 0.6087 |
| FVC | Boys | 4.25 | 93 | BCPE | 1.06987 | 0.7866 | 0.12823 | 4.88371 | 0.7866 | 0.6250 |
| FVC | Boys | 4.25 | 94 | BCPE | 1.06987 | 0.8075 | 0.12823 | 4.88371 | 0.8075 | 0.6417 |
| FVC | Boys | 4.25 | 95 | BCPE | 1.06987 | 0.8287 | 0.12823 | 4.88371 | 0.8287 | 0.6586 |
| FVC | Boys | 4.25 | 96 | BCPE | 1.06987 | 0.8503 | 0.12823 | 4.88371 | 0.8503 | 0.6757 |
| FVC | Boys | 4.25 | 97 | BCPE | 1.06987 | 0.8722 | 0.12823 | 4.88371 | 0.8722 | 0.6931 |
| FVC | Boys | 4.25 | 98 | BCPE | 1.06987 | 0.8945 | 0.12823 | 4.88371 | 0.8945 | 0.7108 |
| FVC | Boys | 4.25 | 99 | BCPE | 1.06987 | 0.9170 | 0.12823 | 4.88371 | 0.9170 | 0.7287 |
| FVC | Boys | 4.25 | 100 | BCPE | 1.06987 | 0.9400 | 0.12823 | 4.88371 | 0.9400 | 0.7469 |
| FVC | Boys | 4.25 | 101 | BCPE | 1.06987 | 0.9632 | 0.12823 | 4.88371 | 0.9632 | 0.7654 |
| FVC | Boys | 4.25 | 102 | BCPE | 1.06987 | 0.9868 | 0.12823 | 4.88371 | 0.9868 | 0.7841 |
| FVC | Boys | 4.25 | 103 | BCPE | 1.06987 | 1.0107 | 0.12823 | 4.88371 | 1.0107 | 0.8031 |
| FVC | Boys | 4.25 | 104 | BCPE | 1.06987 | 1.0350 | 0.12823 | 4.88371 | 1.0350 | 0.8224 |
| FVC | Boys | 4.25 | 105 | BCPE | 1.06987 | 1.0596 | 0.12823 | 4.88371 | 1.0596 | 0.8420 |
| FVC | Boys | 4.25 | 106 | BCPE | 1.06987 | 1.0845 | 0.12823 | 4.88371 | 1.0845 | 0.8618 |
| FVC | Boys | 4.25 | 107 | BCPE | 1.06987 | 1.1098 | 0.12823 | 4.88371 | 1.1098 | 0.8819 |
| FVC | Boys | 4.25 | 108 | BCPE | 1.06987 | 1.1354 | 0.12823 | 4.88371 | 1.1354 | 0.9023 |
| FVC | Boys | 4.25 | 109 | BCPE | 1.06987 | 1.1614 | 0.12823 | 4.88371 | 1.1614 | 0.9229 |
| FVC | Boys | 4.25 | 110 | BCPE | 1.06987 | 1.1877 | 0.12823 | 4.88371 | 1.1877 | 0.9438 |
| FVC | Boys | 4.25 | 111 | BCPE | 1.06987 | 1.2144 | 0.12823 | 4.88371 | 1.2144 | 0.9650 |
| FVC | Boys | 4.25 | 112 | BCPE | 1.06987 | 1.2414 | 0.12823 | 4.88371 | 1.2414 | 0.9865 |
| FVC | Boys | 4.25 | 113 | BCPE | 1.06987 | 1.2688 | 0.12823 | 4.88371 | 1.2688 | 1.0083 |
| FVC | Boys | 4.25 | 114 | BCPE | 1.06987 | 1.2966 | 0.12823 | 4.88371 | 1.2966 | 1.0303 |
| FVC | Boys | 4.25 | 115 | BCPE | 1.06987 | 1.3247 | 0.12823 | 4.88371 | 1.3247 | 1.0526 |
| FVC | Boys | 4.25 | 116 | BCPE | 1.06987 | 1.3531 | 0.12823 | 4.88371 | 1.3531 | 1.0753 |
| FVC | Boys | 4.25 | 117 | BCPE | 1.06987 | 1.3819 | 0.12823 | 4.88371 | 1.3819 | 1.0982 |
| FVC | Boys | 4.25 | 118 | BCPE | 1.06987 | 1.4111 | 0.12823 | 4.88371 | 1.4111 | 1.1213 |
| FVC | Boys | 4.25 | 119 | BCPE | 1.06987 | 1.4407 | 0.12823 | 4.88371 | 1.4407 | 1.1448 |
| FVC | Boys | 4.25 | 120 | BCPE | 1.06987 | 1.4706 | 0.12823 | 4.88371 | 1.4706 | 1.1686 |
| FVC | Boys | 4.25 | 121 | BCPE | 1.06987 | 1.5008 | 0.12823 | 4.88371 | 1.5008 | 1.1926 |
| FVC | Boys | 4.25 | 122 | BCPE | 1.06987 | 1.5315 | 0.12823 | 4.88371 | 1.5315 | 1.2170 |
| FVC | Boys | 4.25 | 123 | BCPE | 1.06987 | 1.5625 | 0.12823 | 4.88371 | 1.5625 | 1.2416 |
| FVC | Boys | 4.25 | 124 | BCPE | 1.06987 | 1.5938 | 0.12823 | 4.88371 | 1.5938 | 1.2665 |
| FVC | Boys | 4.25 | 125 | BCPE | 1.06987 | 1.6256 | 0.12823 | 4.88371 | 1.6256 | 1.2918 |
| FVC | Boys | 4.25 | 126 | BCPE | 1.06987 | 1.6577 | 0.12823 | 4.88371 | 1.6577 | 1.3173 |
| FVC | Boys | 4.25 | 127 | BCPE | 1.06987 | 1.6902 | 0.12823 | 4.88371 | 1.6902 | 1.3431 |
| FVC | Boys | 4.25 | 128 | BCPE | 1.06987 | 1.7230 | 0.12823 | 4.88371 | 1.7230 | 1.3692 |
| FVC | Boys | 4.25 | 129 | BCPE | 1.06987 | 1.7563 | 0.12823 | 4.88371 | 1.7563 | 1.3956 |
| FVC | Boys | 4.25 | 130 | BCPE | 1.06987 | 1.7899 | 0.12823 | 4.88371 | 1.7899 | 1.4223 |
| FVC | Boys | 4.25 | 131 | BCPE | 1.06987 | 1.8238 | 0.12823 | 4.88371 | 1.8238 | 1.4493 |
| FVC | Boys | 4.25 | 132 | BCPE | 1.06987 | 1.8582 | 0.12823 | 4.88371 | 1.8582 | 1.4766 |
| FVC | Boys | 4.50 | 79 | BCPE | 0.98778 | 0.5283 | 0.12809 | 5.00153 | 0.5283 | 0.4210 |
| FVC | Boys | 4.50 | 80 | BCPE | 0.98778 | 0.5448 | 0.12809 | 5.00153 | 0.5448 | 0.4342 |
| FVC | Boys | 4.50 | 81 | BCPE | 0.98778 | 0.5617 | 0.12809 | 5.00153 | 0.5617 | 0.4476 |
| FVC | Boys | 4.50 | 82 | BCPE | 0.98778 | 0.5789 | 0.12809 | 5.00153 | 0.5789 | 0.4613 |
| FVC | Boys | 4.50 | 83 | BCPE | 0.98778 | 0.5964 | 0.12809 | 5.00153 | 0.5964 | 0.4752 |
| FVC | Boys | 4.50 | 84 | BCPE | 0.98778 | 0.6142 | 0.12809 | 5.00153 | 0.6142 | 0.4894 |
| FVC | Boys | 4.50 | 85 | BCPE | 0.98778 | 0.6323 | 0.12809 | 5.00153 | 0.6323 | 0.5039 |
| FVC | Boys | 4.50 | 86 | BCPE | 0.98778 | 0.6507 | 0.12809 | 5.00153 | 0.6507 | 0.5185 |
| FVC | Boys | 4.50 | 87 | BCPE | 0.98778 | 0.6694 | 0.12809 | 5.00153 | 0.6694 | 0.5335 |
| FVC | Boys | 4.50 | 88 | BCPE | 0.98778 | 0.6885 | 0.12809 | 5.00153 | 0.6885 | 0.5486 |
| FVC | Boys | 4.50 | 89 | BCPE | 0.98778 | 0.7078 | 0.12809 | 5.00153 | 0.7078 | 0.5641 |
| FVC | Boys | 4.50 | 90 | BCPE | 0.98778 | 0.7275 | 0.12809 | 5.00153 | 0.7275 | 0.5797 |
| FVC | Boys | 4.50 | 91 | BCPE | 0.98778 | 0.7475 | 0.12809 | 5.00153 | 0.7475 | 0.5957 |
| FVC | Boys | 4.50 | 92 | BCPE | 0.98778 | 0.7678 | 0.12809 | 5.00153 | 0.7678 | 0.6119 |
| FVC | Boys | 4.50 | 93 | BCPE | 0.98778 | 0.7885 | 0.12809 | 5.00153 | 0.7885 | 0.6283 |
| FVC | Boys | 4.50 | 94 | BCPE | 0.98778 | 0.8095 | 0.12809 | 5.00153 | 0.8095 | 0.6451 |
| FVC | Boys | 4.50 | 95 | BCPE | 0.98778 | 0.8308 | 0.12809 | 5.00153 | 0.8308 | 0.6620 |
| FVC | Boys | 4.50 | 96 | BCPE | 0.98778 | 0.8524 | 0.12809 | 5.00153 | 0.8524 | 0.6793 |
| FVC | Boys | 4.50 | 97 | BCPE | 0.98778 | 0.8744 | 0.12809 | 5.00153 | 0.8744 | 0.6968 |
| FVC | Boys | 4.50 | 98 | BCPE | 0.98778 | 0.8967 | 0.12809 | 5.00153 | 0.8967 | 0.7145 |
| FVC | Boys | 4.50 | 99 | BCPE | 0.98778 | 0.9193 | 0.12809 | 5.00153 | 0.9193 | 0.7326 |
| FVC | Boys | 4.50 | 100 | BCPE | 0.98778 | 0.9423 | 0.12809 | 5.00153 | 0.9423 | 0.7509 |
| FVC | Boys | 4.50 | 101 | BCPE | 0.98778 | 0.9656 | 0.12809 | 5.00153 | 0.9656 | 0.7694 |
| FVC | Boys | 4.50 | 102 | BCPE | 0.98778 | 0.9892 | 0.12809 | 5.00153 | 0.9892 | 0.7883 |
| FVC | Boys | 4.50 | 103 | BCPE | 0.98778 | 1.0132 | 0.12809 | 5.00153 | 1.0132 | 0.8074 |
| FVC | Boys | 4.50 | 104 | BCPE | 0.98778 | 1.0375 | 0.12809 | 5.00153 | 1.0375 | 0.8268 |
| FVC | Boys | 4.50 | 105 | BCPE | 0.98778 | 1.0622 | 0.12809 | 5.00153 | 1.0622 | 0.8464 |
| FVC | Boys | 4.50 | 106 | BCPE | 0.98778 | 1.0872 | 0.12809 | 5.00153 | 1.0872 | 0.8663 |
| FVC | Boys | 4.50 | 107 | BCPE | 0.98778 | 1.1125 | 0.12809 | 5.00153 | 1.1125 | 0.8865 |
| FVC | Boys | 4.50 | 108 | BCPE | 0.98778 | 1.1382 | 0.12809 | 5.00153 | 1.1382 | 0.9070 |
| FVC | Boys | 4.50 | 109 | BCPE | 0.98778 | 1.1642 | 0.12809 | 5.00153 | 1.1642 | 0.9278 |
| FVC | Boys | 4.50 | 110 | BCPE | 0.98778 | 1.1906 | 0.12809 | 5.00153 | 1.1906 | 0.9488 |
| FVC | Boys | 4.50 | 111 | BCPE | 0.98778 | 1.2174 | 0.12809 | 5.00153 | 1.2174 | 0.9701 |
| FVC | Boys | 4.50 | 112 | BCPE | 0.98778 | 1.2445 | 0.12809 | 5.00153 | 1.2445 | 0.9917 |
| FVC | Boys | 4.50 | 113 | BCPE | 0.98778 | 1.2719 | 0.12809 | 5.00153 | 1.2719 | 1.0136 |
| FVC | Boys | 4.50 | 114 | BCPE | 0.98778 | 1.2998 | 0.12809 | 5.00153 | 1.2998 | 1.0358 |
| FVC | Boys | 4.50 | 115 | BCPE | 0.98778 | 1.3279 | 0.12809 | 5.00153 | 1.3279 | 1.0582 |
| FVC | Boys | 4.50 | 116 | BCPE | 0.98778 | 1.3565 | 0.12809 | 5.00153 | 1.3565 | 1.0809 |
| FVC | Boys | 4.50 | 117 | BCPE | 0.98778 | 1.3853 | 0.12809 | 5.00153 | 1.3853 | 1.1040 |
| FVC | Boys | 4.50 | 118 | BCPE | 0.98778 | 1.4146 | 0.12809 | 5.00153 | 1.4146 | 1.1273 |
| FVC | Boys | 4.50 | 119 | BCPE | 0.98778 | 1.4442 | 0.12809 | 5.00153 | 1.4442 | 1.1509 |
| FVC | Boys | 4.50 | 120 | BCPE | 0.98778 | 1.4742 | 0.12809 | 5.00153 | 1.4742 | 1.1747 |
| FVC | Boys | 4.50 | 121 | BCPE | 0.98778 | 1.5045 | 0.12809 | 5.00153 | 1.5045 | 1.1989 |
| FVC | Boys | 4.50 | 122 | BCPE | 0.98778 | 1.5352 | 0.12809 | 5.00153 | 1.5352 | 1.2234 |
| FVC | Boys | 4.50 | 123 | BCPE | 0.98778 | 1.5663 | 0.12809 | 5.00153 | 1.5663 | 1.2482 |
| FVC | Boys | 4.50 | 124 | BCPE | 0.98778 | 1.5977 | 0.12809 | 5.00153 | 1.5977 | 1.2732 |
| FVC | Boys | 4.50 | 125 | BCPE | 0.98778 | 1.6296 | 0.12809 | 5.00153 | 1.6296 | 1.2986 |
| FVC | Boys | 4.50 | 126 | BCPE | 0.98778 | 1.6617 | 0.12809 | 5.00153 | 1.6617 | 1.3242 |
| FVC | Boys | 4.50 | 127 | BCPE | 0.98778 | 1.6943 | 0.12809 | 5.00153 | 1.6943 | 1.3502 |
| FVC | Boys | 4.50 | 128 | BCPE | 0.98778 | 1.7272 | 0.12809 | 5.00153 | 1.7272 | 1.3764 |
| FVC | Boys | 4.50 | 129 | BCPE | 0.98778 | 1.7606 | 0.12809 | 5.00153 | 1.7606 | 1.4030 |
| FVC | Boys | 4.50 | 130 | BCPE | 0.98778 | 1.7942 | 0.12809 | 5.00153 | 1.7942 | 1.4298 |
| FVC | Boys | 4.50 | 131 | BCPE | 0.98778 | 1.8283 | 0.12809 | 5.00153 | 1.8283 | 1.4570 |
| FVC | Boys | 4.50 | 132 | BCPE | 0.98778 | 1.8628 | 0.12809 | 5.00153 | 1.8628 | 1.4844 |
| FVC | Boys | 4.75 | 79 | BCPE | 0.91013 | 0.5295 | 0.12795 | 5.11559 | 0.5295 | 0.4230 |
| FVC | Boys | 4.75 | 80 | BCPE | 0.91013 | 0.5461 | 0.12795 | 5.11559 | 0.5461 | 0.4363 |
| FVC | Boys | 4.75 | 81 | BCPE | 0.91013 | 0.5630 | 0.12795 | 5.11559 | 0.5630 | 0.4498 |
| FVC | Boys | 4.75 | 82 | BCPE | 0.91013 | 0.5802 | 0.12795 | 5.11559 | 0.5802 | 0.4636 |
| FVC | Boys | 4.75 | 83 | BCPE | 0.91013 | 0.5977 | 0.12795 | 5.11559 | 0.5977 | 0.4776 |
| FVC | Boys | 4.75 | 84 | BCPE | 0.91013 | 0.6156 | 0.12795 | 5.11559 | 0.6156 | 0.4918 |
| FVC | Boys | 4.75 | 85 | BCPE | 0.91013 | 0.6337 | 0.12795 | 5.11559 | 0.6337 | 0.5063 |
| FVC | Boys | 4.75 | 86 | BCPE | 0.91013 | 0.6522 | 0.12795 | 5.11559 | 0.6522 | 0.5211 |
| FVC | Boys | 4.75 | 87 | BCPE | 0.91013 | 0.6710 | 0.12795 | 5.11559 | 0.6710 | 0.5361 |
| FVC | Boys | 4.75 | 88 | BCPE | 0.91013 | 0.6901 | 0.12795 | 5.11559 | 0.6901 | 0.5513 |
| FVC | Boys | 4.75 | 89 | BCPE | 0.91013 | 0.7095 | 0.12795 | 5.11559 | 0.7095 | 0.5668 |
| FVC | Boys | 4.75 | 90 | BCPE | 0.91013 | 0.7292 | 0.12795 | 5.11559 | 0.7292 | 0.5826 |
| FVC | Boys | 4.75 | 91 | BCPE | 0.91013 | 0.7492 | 0.12795 | 5.11559 | 0.7492 | 0.5986 |
| FVC | Boys | 4.75 | 92 | BCPE | 0.91013 | 0.7696 | 0.12795 | 5.11559 | 0.7696 | 0.6149 |
| FVC | Boys | 4.75 | 93 | BCPE | 0.91013 | 0.7903 | 0.12795 | 5.11559 | 0.7903 | 0.6314 |
| FVC | Boys | 4.75 | 94 | BCPE | 0.91013 | 0.8113 | 0.12795 | 5.11559 | 0.8113 | 0.6482 |
| FVC | Boys | 4.75 | 95 | BCPE | 0.91013 | 0.8327 | 0.12795 | 5.11559 | 0.8327 | 0.6653 |
| FVC | Boys | 4.75 | 96 | BCPE | 0.91013 | 0.8544 | 0.12795 | 5.11559 | 0.8544 | 0.6826 |
| FVC | Boys | 4.75 | 97 | BCPE | 0.91013 | 0.8764 | 0.12795 | 5.11559 | 0.8764 | 0.7002 |
| FVC | Boys | 4.75 | 98 | BCPE | 0.91013 | 0.8987 | 0.12795 | 5.11559 | 0.8987 | 0.7180 |
| FVC | Boys | 4.75 | 99 | BCPE | 0.91013 | 0.9214 | 0.12795 | 5.11559 | 0.9214 | 0.7362 |
| FVC | Boys | 4.75 | 100 | BCPE | 0.91013 | 0.9444 | 0.12795 | 5.11559 | 0.9444 | 0.7545 |
| FVC | Boys | 4.75 | 101 | BCPE | 0.91013 | 0.9678 | 0.12795 | 5.11559 | 0.9678 | 0.7732 |
| FVC | Boys | 4.75 | 102 | BCPE | 0.91013 | 0.9915 | 0.12795 | 5.11559 | 0.9915 | 0.7921 |
| FVC | Boys | 4.75 | 103 | BCPE | 0.91013 | 1.0155 | 0.12795 | 5.11559 | 1.0155 | 0.8113 |
| FVC | Boys | 4.75 | 104 | BCPE | 0.91013 | 1.0399 | 0.12795 | 5.11559 | 1.0399 | 0.8308 |
| FVC | Boys | 4.75 | 105 | BCPE | 0.91013 | 1.0646 | 0.12795 | 5.11559 | 1.0646 | 0.8506 |
| FVC | Boys | 4.75 | 106 | BCPE | 0.91013 | 1.0897 | 0.12795 | 5.11559 | 1.0897 | 0.8706 |
| FVC | Boys | 4.75 | 107 | BCPE | 0.91013 | 1.1151 | 0.12795 | 5.11559 | 1.1151 | 0.8909 |
| FVC | Boys | 4.75 | 108 | BCPE | 0.91013 | 1.1408 | 0.12795 | 5.11559 | 1.1408 | 0.9115 |
| FVC | Boys | 4.75 | 109 | BCPE | 0.91013 | 1.1669 | 0.12795 | 5.11559 | 1.1669 | 0.9323 |
| FVC | Boys | 4.75 | 110 | BCPE | 0.91013 | 1.1934 | 0.12795 | 5.11559 | 1.1934 | 0.9535 |
| FVC | Boys | 4.75 | 111 | BCPE | 0.91013 | 1.2202 | 0.12795 | 5.11559 | 1.2202 | 0.9749 |
| FVC | Boys | 4.75 | 112 | BCPE | 0.91013 | 1.2474 | 0.12795 | 5.11559 | 1.2474 | 0.9966 |
| FVC | Boys | 4.75 | 113 | BCPE | 0.91013 | 1.2749 | 0.12795 | 5.11559 | 1.2749 | 1.0186 |
| FVC | Boys | 4.75 | 114 | BCPE | 0.91013 | 1.3027 | 0.12795 | 5.11559 | 1.3027 | 1.0408 |
| FVC | Boys | 4.75 | 115 | BCPE | 0.91013 | 1.3310 | 0.12795 | 5.11559 | 1.3310 | 1.0634 |
| FVC | Boys | 4.75 | 116 | BCPE | 0.91013 | 1.3596 | 0.12795 | 5.11559 | 1.3596 | 1.0862 |
| FVC | Boys | 4.75 | 117 | BCPE | 0.91013 | 1.3885 | 0.12795 | 5.11559 | 1.3885 | 1.1094 |
| FVC | Boys | 4.75 | 118 | BCPE | 0.91013 | 1.4178 | 0.12795 | 5.11559 | 1.4178 | 1.1328 |
| FVC | Boys | 4.75 | 119 | BCPE | 0.91013 | 1.4475 | 0.12795 | 5.11559 | 1.4475 | 1.1565 |
| FVC | Boys | 4.75 | 120 | BCPE | 0.91013 | 1.4776 | 0.12795 | 5.11559 | 1.4776 | 1.1805 |
| FVC | Boys | 4.75 | 121 | BCPE | 0.91013 | 1.5080 | 0.12795 | 5.11559 | 1.5080 | 1.2048 |
| FVC | Boys | 4.75 | 122 | BCPE | 0.91013 | 1.5387 | 0.12795 | 5.11559 | 1.5387 | 1.2294 |
| FVC | Boys | 4.75 | 123 | BCPE | 0.91013 | 1.5699 | 0.12795 | 5.11559 | 1.5699 | 1.2543 |
| FVC | Boys | 4.75 | 124 | BCPE | 0.91013 | 1.6014 | 0.12795 | 5.11559 | 1.6014 | 1.2794 |
| FVC | Boys | 4.75 | 125 | BCPE | 0.91013 | 1.6333 | 0.12795 | 5.11559 | 1.6333 | 1.3049 |
| FVC | Boys | 4.75 | 126 | BCPE | 0.91013 | 1.6656 | 0.12795 | 5.11559 | 1.6656 | 1.3307 |
| FVC | Boys | 4.75 | 127 | BCPE | 0.91013 | 1.6982 | 0.12795 | 5.11559 | 1.6982 | 1.3568 |
| FVC | Boys | 4.75 | 128 | BCPE | 0.91013 | 1.7312 | 0.12795 | 5.11559 | 1.7312 | 1.3832 |
| FVC | Boys | 4.75 | 129 | BCPE | 0.91013 | 1.7646 | 0.12795 | 5.11559 | 1.7646 | 1.4098 |
| FVC | Boys | 4.75 | 130 | BCPE | 0.91013 | 1.7984 | 0.12795 | 5.11559 | 1.7984 | 1.4368 |
| FVC | Boys | 4.75 | 131 | BCPE | 0.91013 | 1.8325 | 0.12795 | 5.11559 | 1.8325 | 1.4641 |
| FVC | Boys | 4.75 | 132 | BCPE | 0.91013 | 1.8671 | 0.12795 | 5.11559 | 1.8671 | 1.4917 |
| FVC | Boys | 5.00 | 79 | BCPE | 0.83646 | 0.5306 | 0.12783 | 5.22621 | 0.5306 | 0.4250 |
| FVC | Boys | 5.00 | 80 | BCPE | 0.83646 | 0.5473 | 0.12783 | 5.22621 | 0.5473 | 0.4383 |
| FVC | Boys | 5.00 | 81 | BCPE | 0.83646 | 0.5642 | 0.12783 | 5.22621 | 0.5642 | 0.4519 |
| FVC | Boys | 5.00 | 82 | BCPE | 0.83646 | 0.5815 | 0.12783 | 5.22621 | 0.5815 | 0.4657 |
| FVC | Boys | 5.00 | 83 | BCPE | 0.83646 | 0.5990 | 0.12783 | 5.22621 | 0.5990 | 0.4797 |
| FVC | Boys | 5.00 | 84 | BCPE | 0.83646 | 0.6169 | 0.12783 | 5.22621 | 0.6169 | 0.4941 |
| FVC | Boys | 5.00 | 85 | BCPE | 0.83646 | 0.6351 | 0.12783 | 5.22621 | 0.6351 | 0.5086 |
| FVC | Boys | 5.00 | 86 | BCPE | 0.83646 | 0.6536 | 0.12783 | 5.22621 | 0.6536 | 0.5234 |
| FVC | Boys | 5.00 | 87 | BCPE | 0.83646 | 0.6724 | 0.12783 | 5.22621 | 0.6724 | 0.5385 |
| FVC | Boys | 5.00 | 88 | BCPE | 0.83646 | 0.6915 | 0.12783 | 5.22621 | 0.6915 | 0.5538 |
| FVC | Boys | 5.00 | 89 | BCPE | 0.83646 | 0.7110 | 0.12783 | 5.22621 | 0.7110 | 0.5694 |
| FVC | Boys | 5.00 | 90 | BCPE | 0.83646 | 0.7308 | 0.12783 | 5.22621 | 0.7308 | 0.5852 |
| FVC | Boys | 5.00 | 91 | BCPE | 0.83646 | 0.7509 | 0.12783 | 5.22621 | 0.7509 | 0.6013 |
| FVC | Boys | 5.00 | 92 | BCPE | 0.83646 | 0.7713 | 0.12783 | 5.22621 | 0.7713 | 0.6177 |
| FVC | Boys | 5.00 | 93 | BCPE | 0.83646 | 0.7920 | 0.12783 | 5.22621 | 0.7920 | 0.6343 |
| FVC | Boys | 5.00 | 94 | BCPE | 0.83646 | 0.8131 | 0.12783 | 5.22621 | 0.8131 | 0.6512 |
| FVC | Boys | 5.00 | 95 | BCPE | 0.83646 | 0.8345 | 0.12783 | 5.22621 | 0.8345 | 0.6683 |
| FVC | Boys | 5.00 | 96 | BCPE | 0.83646 | 0.8562 | 0.12783 | 5.22621 | 0.8562 | 0.6857 |
| FVC | Boys | 5.00 | 97 | BCPE | 0.83646 | 0.8783 | 0.12783 | 5.22621 | 0.8783 | 0.7034 |
| FVC | Boys | 5.00 | 98 | BCPE | 0.83646 | 0.9007 | 0.12783 | 5.22621 | 0.9007 | 0.7213 |
| FVC | Boys | 5.00 | 99 | BCPE | 0.83646 | 0.9234 | 0.12783 | 5.22621 | 0.9234 | 0.7395 |
| FVC | Boys | 5.00 | 100 | BCPE | 0.83646 | 0.9465 | 0.12783 | 5.22621 | 0.9465 | 0.7580 |
| FVC | Boys | 5.00 | 101 | BCPE | 0.83646 | 0.9699 | 0.12783 | 5.22621 | 0.9699 | 0.7767 |
| FVC | Boys | 5.00 | 102 | BCPE | 0.83646 | 0.9936 | 0.12783 | 5.22621 | 0.9936 | 0.7957 |
| FVC | Boys | 5.00 | 103 | BCPE | 0.83646 | 1.0177 | 0.12783 | 5.22621 | 1.0177 | 0.8150 |
| FVC | Boys | 5.00 | 104 | BCPE | 0.83646 | 1.0421 | 0.12783 | 5.22621 | 1.0421 | 0.8346 |
| FVC | Boys | 5.00 | 105 | BCPE | 0.83646 | 1.0669 | 0.12783 | 5.22621 | 1.0669 | 0.8544 |
| FVC | Boys | 5.00 | 106 | BCPE | 0.83646 | 1.0920 | 0.12783 | 5.22621 | 1.0920 | 0.8745 |
| FVC | Boys | 5.00 | 107 | BCPE | 0.83646 | 1.1175 | 0.12783 | 5.22621 | 1.1175 | 0.8949 |
| FVC | Boys | 5.00 | 108 | BCPE | 0.83646 | 1.1433 | 0.12783 | 5.22621 | 1.1433 | 0.9156 |
| FVC | Boys | 5.00 | 109 | BCPE | 0.83646 | 1.1695 | 0.12783 | 5.22621 | 1.1695 | 0.9366 |
| FVC | Boys | 5.00 | 110 | BCPE | 0.83646 | 1.1960 | 0.12783 | 5.22621 | 1.1960 | 0.9578 |
| FVC | Boys | 5.00 | 111 | BCPE | 0.83646 | 1.2228 | 0.12783 | 5.22621 | 1.2228 | 0.9793 |
| FVC | Boys | 5.00 | 112 | BCPE | 0.83646 | 1.2501 | 0.12783 | 5.22621 | 1.2501 | 1.0011 |
| FVC | Boys | 5.00 | 113 | BCPE | 0.83646 | 1.2776 | 0.12783 | 5.22621 | 1.2776 | 1.0232 |
| FVC | Boys | 5.00 | 114 | BCPE | 0.83646 | 1.3056 | 0.12783 | 5.22621 | 1.3056 | 1.0456 |
| FVC | Boys | 5.00 | 115 | BCPE | 0.83646 | 1.3339 | 0.12783 | 5.22621 | 1.3339 | 1.0682 |
| FVC | Boys | 5.00 | 116 | BCPE | 0.83646 | 1.3625 | 0.12783 | 5.22621 | 1.3625 | 1.0912 |
| FVC | Boys | 5.00 | 117 | BCPE | 0.83646 | 1.3915 | 0.12783 | 5.22621 | 1.3915 | 1.1144 |
| FVC | Boys | 5.00 | 118 | BCPE | 0.83646 | 1.4209 | 0.12783 | 5.22621 | 1.4209 | 1.1379 |
| FVC | Boys | 5.00 | 119 | BCPE | 0.83646 | 1.4507 | 0.12783 | 5.22621 | 1.4507 | 1.1618 |
| FVC | Boys | 5.00 | 120 | BCPE | 0.83646 | 1.4808 | 0.12783 | 5.22621 | 1.4808 | 1.1859 |
| FVC | Boys | 5.00 | 121 | BCPE | 0.83646 | 1.5112 | 0.12783 | 5.22621 | 1.5112 | 1.2103 |
| FVC | Boys | 5.00 | 122 | BCPE | 0.83646 | 1.5421 | 0.12783 | 5.22621 | 1.5421 | 1.2350 |
| FVC | Boys | 5.00 | 123 | BCPE | 0.83646 | 1.5733 | 0.12783 | 5.22621 | 1.5733 | 1.2600 |
| FVC | Boys | 5.00 | 124 | BCPE | 0.83646 | 1.6049 | 0.12783 | 5.22621 | 1.6049 | 1.2853 |
| FVC | Boys | 5.00 | 125 | BCPE | 0.83646 | 1.6368 | 0.12783 | 5.22621 | 1.6368 | 1.3109 |
| FVC | Boys | 5.00 | 126 | BCPE | 0.83646 | 1.6692 | 0.12783 | 5.22621 | 1.6692 | 1.3368 |
| FVC | Boys | 5.00 | 127 | BCPE | 0.83646 | 1.7019 | 0.12783 | 5.22621 | 1.7019 | 1.3630 |
| FVC | Boys | 5.00 | 128 | BCPE | 0.83646 | 1.7350 | 0.12783 | 5.22621 | 1.7350 | 1.3895 |
| FVC | Boys | 5.00 | 129 | BCPE | 0.83646 | 1.7684 | 0.12783 | 5.22621 | 1.7684 | 1.4163 |
| FVC | Boys | 5.00 | 130 | BCPE | 0.83646 | 1.8023 | 0.12783 | 5.22621 | 1.8023 | 1.4434 |
| FVC | Boys | 5.00 | 131 | BCPE | 0.83646 | 1.8365 | 0.12783 | 5.22621 | 1.8365 | 1.4708 |
| FVC | Boys | 5.00 | 132 | BCPE | 0.83646 | 1.8711 | 0.12783 | 5.22621 | 1.8711 | 1.4985 |
| FVC | Boys | 5.25 | 79 | BCPE | 0.76639 | 0.5317 | 0.12770 | 5.33365 | 0.5317 | 0.4268 |
| FVC | Boys | 5.25 | 80 | BCPE | 0.76639 | 0.5484 | 0.12770 | 5.33365 | 0.5484 | 0.4402 |
| FVC | Boys | 5.25 | 81 | BCPE | 0.76639 | 0.5654 | 0.12770 | 5.33365 | 0.5654 | 0.4538 |
| FVC | Boys | 5.25 | 82 | BCPE | 0.76639 | 0.5827 | 0.12770 | 5.33365 | 0.5827 | 0.4677 |
| FVC | Boys | 5.25 | 83 | BCPE | 0.76639 | 0.6003 | 0.12770 | 5.33365 | 0.6003 | 0.4818 |
| FVC | Boys | 5.25 | 84 | BCPE | 0.76639 | 0.6182 | 0.12770 | 5.33365 | 0.6182 | 0.4962 |
| FVC | Boys | 5.25 | 85 | BCPE | 0.76639 | 0.6364 | 0.12770 | 5.33365 | 0.6364 | 0.5108 |
| FVC | Boys | 5.25 | 86 | BCPE | 0.76639 | 0.6550 | 0.12770 | 5.33365 | 0.6550 | 0.5257 |
| FVC | Boys | 5.25 | 87 | BCPE | 0.76639 | 0.6738 | 0.12770 | 5.33365 | 0.6738 | 0.5408 |
| FVC | Boys | 5.25 | 88 | BCPE | 0.76639 | 0.6930 | 0.12770 | 5.33365 | 0.6930 | 0.5562 |
| FVC | Boys | 5.25 | 89 | BCPE | 0.76639 | 0.7125 | 0.12770 | 5.33365 | 0.7125 | 0.5718 |
| FVC | Boys | 5.25 | 90 | BCPE | 0.76639 | 0.7323 | 0.12770 | 5.33365 | 0.7323 | 0.5877 |
| FVC | Boys | 5.25 | 91 | BCPE | 0.76639 | 0.7524 | 0.12770 | 5.33365 | 0.7524 | 0.6039 |
| FVC | Boys | 5.25 | 92 | BCPE | 0.76639 | 0.7729 | 0.12770 | 5.33365 | 0.7729 | 0.6203 |
| FVC | Boys | 5.25 | 93 | BCPE | 0.76639 | 0.7937 | 0.12770 | 5.33365 | 0.7937 | 0.6370 |
| FVC | Boys | 5.25 | 94 | BCPE | 0.76639 | 0.8148 | 0.12770 | 5.33365 | 0.8148 | 0.6540 |
| FVC | Boys | 5.25 | 95 | BCPE | 0.76639 | 0.8362 | 0.12770 | 5.33365 | 0.8362 | 0.6712 |
| FVC | Boys | 5.25 | 96 | BCPE | 0.76639 | 0.8580 | 0.12770 | 5.33365 | 0.8580 | 0.6886 |
| FVC | Boys | 5.25 | 97 | BCPE | 0.76639 | 0.8801 | 0.12770 | 5.33365 | 0.8801 | 0.7064 |
| FVC | Boys | 5.25 | 98 | BCPE | 0.76639 | 0.9025 | 0.12770 | 5.33365 | 0.9025 | 0.7244 |
| FVC | Boys | 5.25 | 99 | BCPE | 0.76639 | 0.9253 | 0.12770 | 5.33365 | 0.9253 | 0.7427 |
| FVC | Boys | 5.25 | 100 | BCPE | 0.76639 | 0.9484 | 0.12770 | 5.33365 | 0.9484 | 0.7612 |
| FVC | Boys | 5.25 | 101 | BCPE | 0.76639 | 0.9719 | 0.12770 | 5.33365 | 0.9719 | 0.7801 |
| FVC | Boys | 5.25 | 102 | BCPE | 0.76639 | 0.9957 | 0.12770 | 5.33365 | 0.9957 | 0.7992 |
| FVC | Boys | 5.25 | 103 | BCPE | 0.76639 | 1.0198 | 0.12770 | 5.33365 | 1.0198 | 0.8185 |
| FVC | Boys | 5.25 | 104 | BCPE | 0.76639 | 1.0443 | 0.12770 | 5.33365 | 1.0443 | 0.8382 |
| FVC | Boys | 5.25 | 105 | BCPE | 0.76639 | 1.0691 | 0.12770 | 5.33365 | 1.0691 | 0.8581 |
| FVC | Boys | 5.25 | 106 | BCPE | 0.76639 | 1.0943 | 0.12770 | 5.33365 | 1.0943 | 0.8783 |
| FVC | Boys | 5.25 | 107 | BCPE | 0.76639 | 1.1198 | 0.12770 | 5.33365 | 1.1198 | 0.8988 |
| FVC | Boys | 5.25 | 108 | BCPE | 0.76639 | 1.1457 | 0.12770 | 5.33365 | 1.1457 | 0.9195 |
| FVC | Boys | 5.25 | 109 | BCPE | 0.76639 | 1.1719 | 0.12770 | 5.33365 | 1.1719 | 0.9406 |
| FVC | Boys | 5.25 | 110 | BCPE | 0.76639 | 1.1984 | 0.12770 | 5.33365 | 1.1984 | 0.9619 |
| FVC | Boys | 5.25 | 111 | BCPE | 0.76639 | 1.2254 | 0.12770 | 5.33365 | 1.2254 | 0.9835 |
| FVC | Boys | 5.25 | 112 | BCPE | 0.76639 | 1.2526 | 0.12770 | 5.33365 | 1.2526 | 1.0054 |
| FVC | Boys | 5.25 | 113 | BCPE | 0.76639 | 1.2803 | 0.12770 | 5.33365 | 1.2803 | 1.0276 |
| FVC | Boys | 5.25 | 114 | BCPE | 0.76639 | 1.3083 | 0.12770 | 5.33365 | 1.3083 | 1.0500 |
| FVC | Boys | 5.25 | 115 | BCPE | 0.76639 | 1.3366 | 0.12770 | 5.33365 | 1.3366 | 1.0728 |
| FVC | Boys | 5.25 | 116 | BCPE | 0.76639 | 1.3653 | 0.12770 | 5.33365 | 1.3653 | 1.0959 |
| FVC | Boys | 5.25 | 117 | BCPE | 0.76639 | 1.3944 | 0.12770 | 5.33365 | 1.3944 | 1.1192 |
| FVC | Boys | 5.25 | 118 | BCPE | 0.76639 | 1.4238 | 0.12770 | 5.33365 | 1.4238 | 1.1428 |
| FVC | Boys | 5.25 | 119 | BCPE | 0.76639 | 1.4537 | 0.12770 | 5.33365 | 1.4537 | 1.1667 |
| FVC | Boys | 5.25 | 120 | BCPE | 0.76639 | 1.4838 | 0.12770 | 5.33365 | 1.4838 | 1.1910 |
| FVC | Boys | 5.25 | 121 | BCPE | 0.76639 | 1.5144 | 0.12770 | 5.33365 | 1.5144 | 1.2155 |
| FVC | Boys | 5.25 | 122 | BCPE | 0.76639 | 1.5453 | 0.12770 | 5.33365 | 1.5453 | 1.2403 |
| FVC | Boys | 5.25 | 123 | BCPE | 0.76639 | 1.5766 | 0.12770 | 5.33365 | 1.5766 | 1.2654 |
| FVC | Boys | 5.25 | 124 | BCPE | 0.76639 | 1.6082 | 0.12770 | 5.33365 | 1.6082 | 1.2908 |
| FVC | Boys | 5.25 | 125 | BCPE | 0.76639 | 1.6402 | 0.12770 | 5.33365 | 1.6402 | 1.3165 |
| FVC | Boys | 5.25 | 126 | BCPE | 0.76639 | 1.6726 | 0.12770 | 5.33365 | 1.6726 | 1.3425 |
| FVC | Boys | 5.25 | 127 | BCPE | 0.76639 | 1.7054 | 0.12770 | 5.33365 | 1.7054 | 1.3688 |
| FVC | Boys | 5.25 | 128 | BCPE | 0.76639 | 1.7386 | 0.12770 | 5.33365 | 1.7386 | 1.3954 |
| FVC | Boys | 5.25 | 129 | BCPE | 0.76639 | 1.7721 | 0.12770 | 5.33365 | 1.7721 | 1.4223 |
| FVC | Boys | 5.25 | 130 | BCPE | 0.76639 | 1.8060 | 0.12770 | 5.33365 | 1.8060 | 1.4495 |
| FVC | Boys | 5.25 | 131 | BCPE | 0.76639 | 1.8403 | 0.12770 | 5.33365 | 1.8403 | 1.4771 |
| FVC | Boys | 5.25 | 132 | BCPE | 0.76639 | 1.8750 | 0.12770 | 5.33365 | 1.8750 | 1.5049 |
| FVC | Boys | 5.50 | 79 | BCPE | 0.69958 | 0.5328 | 0.12759 | 5.43814 | 0.5328 | 0.4285 |
| FVC | Boys | 5.50 | 80 | BCPE | 0.69958 | 0.5495 | 0.12759 | 5.43814 | 0.5495 | 0.4419 |
| FVC | Boys | 5.50 | 81 | BCPE | 0.69958 | 0.5665 | 0.12759 | 5.43814 | 0.5665 | 0.4556 |
| FVC | Boys | 5.50 | 82 | BCPE | 0.69958 | 0.5838 | 0.12759 | 5.43814 | 0.5838 | 0.4696 |
| FVC | Boys | 5.50 | 83 | BCPE | 0.69958 | 0.6015 | 0.12759 | 5.43814 | 0.6015 | 0.4837 |
| FVC | Boys | 5.50 | 84 | BCPE | 0.69958 | 0.6194 | 0.12759 | 5.43814 | 0.6194 | 0.4982 |
| FVC | Boys | 5.50 | 85 | BCPE | 0.69958 | 0.6377 | 0.12759 | 5.43814 | 0.6377 | 0.5129 |
| FVC | Boys | 5.50 | 86 | BCPE | 0.69958 | 0.6562 | 0.12759 | 5.43814 | 0.6562 | 0.5278 |
| FVC | Boys | 5.50 | 87 | BCPE | 0.69958 | 0.6751 | 0.12759 | 5.43814 | 0.6751 | 0.5430 |
| FVC | Boys | 5.50 | 88 | BCPE | 0.69958 | 0.6944 | 0.12759 | 5.43814 | 0.6944 | 0.5584 |
| FVC | Boys | 5.50 | 89 | BCPE | 0.69958 | 0.7139 | 0.12759 | 5.43814 | 0.7139 | 0.5742 |
| FVC | Boys | 5.50 | 90 | BCPE | 0.69958 | 0.7337 | 0.12759 | 5.43814 | 0.7337 | 0.5901 |
| FVC | Boys | 5.50 | 91 | BCPE | 0.69958 | 0.7539 | 0.12759 | 5.43814 | 0.7539 | 0.6063 |
| FVC | Boys | 5.50 | 92 | BCPE | 0.69958 | 0.7744 | 0.12759 | 5.43814 | 0.7744 | 0.6228 |
| FVC | Boys | 5.50 | 93 | BCPE | 0.69958 | 0.7952 | 0.12759 | 5.43814 | 0.7952 | 0.6396 |
| FVC | Boys | 5.50 | 94 | BCPE | 0.69958 | 0.8164 | 0.12759 | 5.43814 | 0.8164 | 0.6566 |
| FVC | Boys | 5.50 | 95 | BCPE | 0.69958 | 0.8379 | 0.12759 | 5.43814 | 0.8379 | 0.6739 |
| FVC | Boys | 5.50 | 96 | BCPE | 0.69958 | 0.8597 | 0.12759 | 5.43814 | 0.8597 | 0.6914 |
| FVC | Boys | 5.50 | 97 | BCPE | 0.69958 | 0.8818 | 0.12759 | 5.43814 | 0.8818 | 0.7092 |
| FVC | Boys | 5.50 | 98 | BCPE | 0.69958 | 0.9043 | 0.12759 | 5.43814 | 0.9043 | 0.7273 |
| FVC | Boys | 5.50 | 99 | BCPE | 0.69958 | 0.9272 | 0.12759 | 5.43814 | 0.9272 | 0.7457 |
| FVC | Boys | 5.50 | 100 | BCPE | 0.69958 | 0.9503 | 0.12759 | 5.43814 | 0.9503 | 0.7643 |
| FVC | Boys | 5.50 | 101 | BCPE | 0.69958 | 0.9738 | 0.12759 | 5.43814 | 0.9738 | 0.7832 |
| FVC | Boys | 5.50 | 102 | BCPE | 0.69958 | 0.9976 | 0.12759 | 5.43814 | 0.9976 | 0.8024 |
| FVC | Boys | 5.50 | 103 | BCPE | 0.69958 | 1.0218 | 0.12759 | 5.43814 | 1.0218 | 0.8218 |
| FVC | Boys | 5.50 | 104 | BCPE | 0.69958 | 1.0464 | 0.12759 | 5.43814 | 1.0464 | 0.8416 |
| FVC | Boys | 5.50 | 105 | BCPE | 0.69958 | 1.0712 | 0.12759 | 5.43814 | 1.0712 | 0.8616 |
| FVC | Boys | 5.50 | 106 | BCPE | 0.69958 | 1.0964 | 0.12759 | 5.43814 | 1.0964 | 0.8818 |
| FVC | Boys | 5.50 | 107 | BCPE | 0.69958 | 1.1220 | 0.12759 | 5.43814 | 1.1220 | 0.9024 |
| FVC | Boys | 5.50 | 108 | BCPE | 0.69958 | 1.1479 | 0.12759 | 5.43814 | 1.1479 | 0.9232 |
| FVC | Boys | 5.50 | 109 | BCPE | 0.69958 | 1.1742 | 0.12759 | 5.43814 | 1.1742 | 0.9444 |
| FVC | Boys | 5.50 | 110 | BCPE | 0.69958 | 1.2008 | 0.12759 | 5.43814 | 1.2008 | 0.9658 |
| FVC | Boys | 5.50 | 111 | BCPE | 0.69958 | 1.2278 | 0.12759 | 5.43814 | 1.2278 | 0.9875 |
| FVC | Boys | 5.50 | 112 | BCPE | 0.69958 | 1.2551 | 0.12759 | 5.43814 | 1.2551 | 1.0095 |
| FVC | Boys | 5.50 | 113 | BCPE | 0.69958 | 1.2828 | 0.12759 | 5.43814 | 1.2828 | 1.0317 |
| FVC | Boys | 5.50 | 114 | BCPE | 0.69958 | 1.3109 | 0.12759 | 5.43814 | 1.3109 | 1.0543 |
| FVC | Boys | 5.50 | 115 | BCPE | 0.69958 | 1.3393 | 0.12759 | 5.43814 | 1.3393 | 1.0771 |
| FVC | Boys | 5.50 | 116 | BCPE | 0.69958 | 1.3680 | 0.12759 | 5.43814 | 1.3680 | 1.1003 |
| FVC | Boys | 5.50 | 117 | BCPE | 0.69958 | 1.3972 | 0.12759 | 5.43814 | 1.3972 | 1.1237 |
| FVC | Boys | 5.50 | 118 | BCPE | 0.69958 | 1.4267 | 0.12759 | 5.43814 | 1.4267 | 1.1474 |
| FVC | Boys | 5.50 | 119 | BCPE | 0.69958 | 1.4565 | 0.12759 | 5.43814 | 1.4565 | 1.1715 |
| FVC | Boys | 5.50 | 120 | BCPE | 0.69958 | 1.4868 | 0.12759 | 5.43814 | 1.4868 | 1.1958 |
| FVC | Boys | 5.50 | 121 | BCPE | 0.69958 | 1.5174 | 0.12759 | 5.43814 | 1.5174 | 1.2204 |
| FVC | Boys | 5.50 | 122 | BCPE | 0.69958 | 1.5483 | 0.12759 | 5.43814 | 1.5483 | 1.2453 |
| FVC | Boys | 5.50 | 123 | BCPE | 0.69958 | 1.5797 | 0.12759 | 5.43814 | 1.5797 | 1.2705 |
| FVC | Boys | 5.50 | 124 | BCPE | 0.69958 | 1.6114 | 0.12759 | 5.43814 | 1.6114 | 1.2960 |
| FVC | Boys | 5.50 | 125 | BCPE | 0.69958 | 1.6435 | 0.12759 | 5.43814 | 1.6435 | 1.3218 |
| FVC | Boys | 5.50 | 126 | BCPE | 0.69958 | 1.6759 | 0.12759 | 5.43814 | 1.6759 | 1.3479 |
| FVC | Boys | 5.50 | 127 | BCPE | 0.69958 | 1.7088 | 0.12759 | 5.43814 | 1.7088 | 1.3743 |
| FVC | Boys | 5.50 | 128 | BCPE | 0.69958 | 1.7420 | 0.12759 | 5.43814 | 1.7420 | 1.4010 |
| FVC | Boys | 5.50 | 129 | BCPE | 0.69958 | 1.7756 | 0.12759 | 5.43814 | 1.7756 | 1.4281 |
| FVC | Boys | 5.50 | 130 | BCPE | 0.69958 | 1.8096 | 0.12759 | 5.43814 | 1.8096 | 1.4554 |
| FVC | Boys | 5.50 | 131 | BCPE | 0.69958 | 1.8439 | 0.12759 | 5.43814 | 1.8439 | 1.4830 |
| FVC | Boys | 5.50 | 132 | BCPE | 0.69958 | 1.8787 | 0.12759 | 5.43814 | 1.8787 | 1.5110 |
| FVC | Boys | 5.75 | 79 | BCPE | 0.63574 | 0.5338 | 0.12748 | 5.53990 | 0.5338 | 0.4301 |
| FVC | Boys | 5.75 | 80 | BCPE | 0.63574 | 0.5505 | 0.12748 | 5.53990 | 0.5505 | 0.4436 |
| FVC | Boys | 5.75 | 81 | BCPE | 0.63574 | 0.5676 | 0.12748 | 5.53990 | 0.5676 | 0.4574 |
| FVC | Boys | 5.75 | 82 | BCPE | 0.63574 | 0.5849 | 0.12748 | 5.53990 | 0.5849 | 0.4714 |
| FVC | Boys | 5.75 | 83 | BCPE | 0.63574 | 0.6026 | 0.12748 | 5.53990 | 0.6026 | 0.4856 |
| FVC | Boys | 5.75 | 84 | BCPE | 0.63574 | 0.6206 | 0.12748 | 5.53990 | 0.6206 | 0.5001 |
| FVC | Boys | 5.75 | 85 | BCPE | 0.63574 | 0.6389 | 0.12748 | 5.53990 | 0.6389 | 0.5148 |
| FVC | Boys | 5.75 | 86 | BCPE | 0.63574 | 0.6575 | 0.12748 | 5.53990 | 0.6575 | 0.5298 |
| FVC | Boys | 5.75 | 87 | BCPE | 0.63574 | 0.6764 | 0.12748 | 5.53990 | 0.6764 | 0.5451 |
| FVC | Boys | 5.75 | 88 | BCPE | 0.63574 | 0.6957 | 0.12748 | 5.53990 | 0.6957 | 0.5606 |
| FVC | Boys | 5.75 | 89 | BCPE | 0.63574 | 0.7152 | 0.12748 | 5.53990 | 0.7152 | 0.5763 |
| FVC | Boys | 5.75 | 90 | BCPE | 0.63574 | 0.7351 | 0.12748 | 5.53990 | 0.7351 | 0.5924 |
| FVC | Boys | 5.75 | 91 | BCPE | 0.63574 | 0.7553 | 0.12748 | 5.53990 | 0.7553 | 0.6087 |
| FVC | Boys | 5.75 | 92 | BCPE | 0.63574 | 0.7759 | 0.12748 | 5.53990 | 0.7759 | 0.6252 |
| FVC | Boys | 5.75 | 93 | BCPE | 0.63574 | 0.7967 | 0.12748 | 5.53990 | 0.7967 | 0.6420 |
| FVC | Boys | 5.75 | 94 | BCPE | 0.63574 | 0.8179 | 0.12748 | 5.53990 | 0.8179 | 0.6591 |
| FVC | Boys | 5.75 | 95 | BCPE | 0.63574 | 0.8395 | 0.12748 | 5.53990 | 0.8395 | 0.6765 |
| FVC | Boys | 5.75 | 96 | BCPE | 0.63574 | 0.8613 | 0.12748 | 5.53990 | 0.8613 | 0.6941 |
| FVC | Boys | 5.75 | 97 | BCPE | 0.63574 | 0.8835 | 0.12748 | 5.53990 | 0.8835 | 0.7120 |
| FVC | Boys | 5.75 | 98 | BCPE | 0.63574 | 0.9060 | 0.12748 | 5.53990 | 0.9060 | 0.7301 |
| FVC | Boys | 5.75 | 99 | BCPE | 0.63574 | 0.9289 | 0.12748 | 5.53990 | 0.9289 | 0.7485 |
| FVC | Boys | 5.75 | 100 | BCPE | 0.63574 | 0.9521 | 0.12748 | 5.53990 | 0.9521 | 0.7672 |
| FVC | Boys | 5.75 | 101 | BCPE | 0.63574 | 0.9757 | 0.12748 | 5.53990 | 0.9757 | 0.7862 |
| FVC | Boys | 5.75 | 102 | BCPE | 0.63574 | 0.9995 | 0.12748 | 5.53990 | 0.9995 | 0.8054 |
| FVC | Boys | 5.75 | 103 | BCPE | 0.63574 | 1.0238 | 0.12748 | 5.53990 | 1.0238 | 0.8250 |
| FVC | Boys | 5.75 | 104 | BCPE | 0.63574 | 1.0483 | 0.12748 | 5.53990 | 1.0483 | 0.8448 |
| FVC | Boys | 5.75 | 105 | BCPE | 0.63574 | 1.0733 | 0.12748 | 5.53990 | 1.0733 | 0.8649 |
| FVC | Boys | 5.75 | 106 | BCPE | 0.63574 | 1.0985 | 0.12748 | 5.53990 | 1.0985 | 0.8852 |
| FVC | Boys | 5.75 | 107 | BCPE | 0.63574 | 1.1241 | 0.12748 | 5.53990 | 1.1241 | 0.9059 |
| FVC | Boys | 5.75 | 108 | BCPE | 0.63574 | 1.1501 | 0.12748 | 5.53990 | 1.1501 | 0.9268 |
| FVC | Boys | 5.75 | 109 | BCPE | 0.63574 | 1.1764 | 0.12748 | 5.53990 | 1.1764 | 0.9480 |
| FVC | Boys | 5.75 | 110 | BCPE | 0.63574 | 1.2031 | 0.12748 | 5.53990 | 1.2031 | 0.9695 |
| FVC | Boys | 5.75 | 111 | BCPE | 0.63574 | 1.2301 | 0.12748 | 5.53990 | 1.2301 | 0.9913 |
| FVC | Boys | 5.75 | 112 | BCPE | 0.63574 | 1.2575 | 0.12748 | 5.53990 | 1.2575 | 1.0133 |
| FVC | Boys | 5.75 | 113 | BCPE | 0.63574 | 1.2853 | 0.12748 | 5.53990 | 1.2853 | 1.0357 |
| FVC | Boys | 5.75 | 114 | BCPE | 0.63574 | 1.3134 | 0.12748 | 5.53990 | 1.3134 | 1.0583 |
| FVC | Boys | 5.75 | 115 | BCPE | 0.63574 | 1.3418 | 0.12748 | 5.53990 | 1.3418 | 1.0813 |
| FVC | Boys | 5.75 | 116 | BCPE | 0.63574 | 1.3706 | 0.12748 | 5.53990 | 1.3706 | 1.1045 |
| FVC | Boys | 5.75 | 117 | BCPE | 0.63574 | 1.3998 | 0.12748 | 5.53990 | 1.3998 | 1.1280 |
| FVC | Boys | 5.75 | 118 | BCPE | 0.63574 | 1.4294 | 0.12748 | 5.53990 | 1.4294 | 1.1518 |
| FVC | Boys | 5.75 | 119 | BCPE | 0.63574 | 1.4593 | 0.12748 | 5.53990 | 1.4593 | 1.1759 |
| FVC | Boys | 5.75 | 120 | BCPE | 0.63574 | 1.4896 | 0.12748 | 5.53990 | 1.4896 | 1.2003 |
| FVC | Boys | 5.75 | 121 | BCPE | 0.63574 | 1.5202 | 0.12748 | 5.53990 | 1.5202 | 1.2250 |
| FVC | Boys | 5.75 | 122 | BCPE | 0.63574 | 1.5513 | 0.12748 | 5.53990 | 1.5513 | 1.2500 |
| FVC | Boys | 5.75 | 123 | BCPE | 0.63574 | 1.5827 | 0.12748 | 5.53990 | 1.5827 | 1.2753 |
| FVC | Boys | 5.75 | 124 | BCPE | 0.63574 | 1.6145 | 0.12748 | 5.53990 | 1.6145 | 1.3010 |
| FVC | Boys | 5.75 | 125 | BCPE | 0.63574 | 1.6466 | 0.12748 | 5.53990 | 1.6466 | 1.3269 |
| FVC | Boys | 5.75 | 126 | BCPE | 0.63574 | 1.6791 | 0.12748 | 5.53990 | 1.6791 | 1.3531 |
| FVC | Boys | 5.75 | 127 | BCPE | 0.63574 | 1.7120 | 0.12748 | 5.53990 | 1.7120 | 1.3796 |
| FVC | Boys | 5.75 | 128 | BCPE | 0.63574 | 1.7453 | 0.12748 | 5.53990 | 1.7453 | 1.4064 |
| FVC | Boys | 5.75 | 129 | BCPE | 0.63574 | 1.7790 | 0.12748 | 5.53990 | 1.7790 | 1.4335 |
| FVC | Boys | 5.75 | 130 | BCPE | 0.63574 | 1.8130 | 0.12748 | 5.53990 | 1.8130 | 1.4610 |
| FVC | Boys | 5.75 | 131 | BCPE | 0.63574 | 1.8474 | 0.12748 | 5.53990 | 1.8474 | 1.4887 |
| FVC | Boys | 5.75 | 132 | BCPE | 0.63574 | 1.8823 | 0.12748 | 5.53990 | 1.8823 | 1.5168 |
| FVC | Boys | 6.00 | 79 | BCPE | 0.57462 | 0.5348 | 0.12737 | 5.63911 | 0.5348 | 0.4317 |
| FVC | Boys | 6.00 | 80 | BCPE | 0.57462 | 0.5515 | 0.12737 | 5.63911 | 0.5515 | 0.4452 |
| FVC | Boys | 6.00 | 81 | BCPE | 0.57462 | 0.5686 | 0.12737 | 5.63911 | 0.5686 | 0.4590 |
| FVC | Boys | 6.00 | 82 | BCPE | 0.57462 | 0.5860 | 0.12737 | 5.63911 | 0.5860 | 0.4731 |
| FVC | Boys | 6.00 | 83 | BCPE | 0.57462 | 0.6037 | 0.12737 | 5.63911 | 0.6037 | 0.4874 |
| FVC | Boys | 6.00 | 84 | BCPE | 0.57462 | 0.6217 | 0.12737 | 5.63911 | 0.6217 | 0.5019 |
| FVC | Boys | 6.00 | 85 | BCPE | 0.57462 | 0.6401 | 0.12737 | 5.63911 | 0.6401 | 0.5167 |
| FVC | Boys | 6.00 | 86 | BCPE | 0.57462 | 0.6587 | 0.12737 | 5.63911 | 0.6587 | 0.5317 |
| FVC | Boys | 6.00 | 87 | BCPE | 0.57462 | 0.6777 | 0.12737 | 5.63911 | 0.6777 | 0.5470 |
| FVC | Boys | 6.00 | 88 | BCPE | 0.57462 | 0.6969 | 0.12737 | 5.63911 | 0.6969 | 0.5626 |
| FVC | Boys | 6.00 | 89 | BCPE | 0.57462 | 0.7165 | 0.12737 | 5.63911 | 0.7165 | 0.5784 |
| FVC | Boys | 6.00 | 90 | BCPE | 0.57462 | 0.7365 | 0.12737 | 5.63911 | 0.7365 | 0.5945 |
| FVC | Boys | 6.00 | 91 | BCPE | 0.57462 | 0.7567 | 0.12737 | 5.63911 | 0.7567 | 0.6109 |
| FVC | Boys | 6.00 | 92 | BCPE | 0.57462 | 0.7773 | 0.12737 | 5.63911 | 0.7773 | 0.6275 |
| FVC | Boys | 6.00 | 93 | BCPE | 0.57462 | 0.7982 | 0.12737 | 5.63911 | 0.7982 | 0.6444 |
| FVC | Boys | 6.00 | 94 | BCPE | 0.57462 | 0.8194 | 0.12737 | 5.63911 | 0.8194 | 0.6615 |
| FVC | Boys | 6.00 | 95 | BCPE | 0.57462 | 0.8410 | 0.12737 | 5.63911 | 0.8410 | 0.6789 |
| FVC | Boys | 6.00 | 96 | BCPE | 0.57462 | 0.8629 | 0.12737 | 5.63911 | 0.8629 | 0.6966 |
| FVC | Boys | 6.00 | 97 | BCPE | 0.57462 | 0.8851 | 0.12737 | 5.63911 | 0.8851 | 0.7145 |
| FVC | Boys | 6.00 | 98 | BCPE | 0.57462 | 0.9077 | 0.12737 | 5.63911 | 0.9077 | 0.7328 |
| FVC | Boys | 6.00 | 99 | BCPE | 0.57462 | 0.9306 | 0.12737 | 5.63911 | 0.9306 | 0.7512 |
| FVC | Boys | 6.00 | 100 | BCPE | 0.57462 | 0.9538 | 0.12737 | 5.63911 | 0.9538 | 0.7700 |
| FVC | Boys | 6.00 | 101 | BCPE | 0.57462 | 0.9774 | 0.12737 | 5.63911 | 0.9774 | 0.7890 |
| FVC | Boys | 6.00 | 102 | BCPE | 0.57462 | 1.0014 | 0.12737 | 5.63911 | 1.0014 | 0.8084 |
| FVC | Boys | 6.00 | 103 | BCPE | 0.57462 | 1.0256 | 0.12737 | 5.63911 | 1.0256 | 0.8280 |
| FVC | Boys | 6.00 | 104 | BCPE | 0.57462 | 1.0503 | 0.12737 | 5.63911 | 1.0503 | 0.8478 |
| FVC | Boys | 6.00 | 105 | BCPE | 0.57462 | 1.0752 | 0.12737 | 5.63911 | 1.0752 | 0.8680 |
| FVC | Boys | 6.00 | 106 | BCPE | 0.57462 | 1.1005 | 0.12737 | 5.63911 | 1.1005 | 0.8884 |
| FVC | Boys | 6.00 | 107 | BCPE | 0.57462 | 1.1262 | 0.12737 | 5.63911 | 1.1262 | 0.9091 |
| FVC | Boys | 6.00 | 108 | BCPE | 0.57462 | 1.1522 | 0.12737 | 5.63911 | 1.1522 | 0.9301 |
| FVC | Boys | 6.00 | 109 | BCPE | 0.57462 | 1.1786 | 0.12737 | 5.63911 | 1.1786 | 0.9514 |
| FVC | Boys | 6.00 | 110 | BCPE | 0.57462 | 1.2053 | 0.12737 | 5.63911 | 1.2053 | 0.9730 |
| FVC | Boys | 6.00 | 111 | BCPE | 0.57462 | 1.2324 | 0.12737 | 5.63911 | 1.2324 | 0.9948 |
| FVC | Boys | 6.00 | 112 | BCPE | 0.57462 | 1.2598 | 0.12737 | 5.63911 | 1.2598 | 1.0170 |
| FVC | Boys | 6.00 | 113 | BCPE | 0.57462 | 1.2876 | 0.12737 | 5.63911 | 1.2876 | 1.0394 |
| FVC | Boys | 6.00 | 114 | BCPE | 0.57462 | 1.3157 | 0.12737 | 5.63911 | 1.3157 | 1.0622 |
| FVC | Boys | 6.00 | 115 | BCPE | 0.57462 | 1.3443 | 0.12737 | 5.63911 | 1.3443 | 1.0852 |
| FVC | Boys | 6.00 | 116 | BCPE | 0.57462 | 1.3731 | 0.12737 | 5.63911 | 1.3731 | 1.1085 |
| FVC | Boys | 6.00 | 117 | BCPE | 0.57462 | 1.4024 | 0.12737 | 5.63911 | 1.4024 | 1.1321 |
| FVC | Boys | 6.00 | 118 | BCPE | 0.57462 | 1.4320 | 0.12737 | 5.63911 | 1.4320 | 1.1560 |
| FVC | Boys | 6.00 | 119 | BCPE | 0.57462 | 1.4620 | 0.12737 | 5.63911 | 1.4620 | 1.1802 |
| FVC | Boys | 6.00 | 120 | BCPE | 0.57462 | 1.4923 | 0.12737 | 5.63911 | 1.4923 | 1.2047 |
| FVC | Boys | 6.00 | 121 | BCPE | 0.57462 | 1.5230 | 0.12737 | 5.63911 | 1.5230 | 1.2295 |
| FVC | Boys | 6.00 | 122 | BCPE | 0.57462 | 1.5541 | 0.12737 | 5.63911 | 1.5541 | 1.2546 |
| FVC | Boys | 6.00 | 123 | BCPE | 0.57462 | 1.5856 | 0.12737 | 5.63911 | 1.5856 | 1.2800 |
| FVC | Boys | 6.00 | 124 | BCPE | 0.57462 | 1.6174 | 0.12737 | 5.63911 | 1.6174 | 1.3057 |
| FVC | Boys | 6.00 | 125 | BCPE | 0.57462 | 1.6496 | 0.12737 | 5.63911 | 1.6496 | 1.3317 |
| FVC | Boys | 6.00 | 126 | BCPE | 0.57462 | 1.6822 | 0.12737 | 5.63911 | 1.6822 | 1.3580 |
| FVC | Boys | 6.00 | 127 | BCPE | 0.57462 | 1.7151 | 0.12737 | 5.63911 | 1.7151 | 1.3846 |
| FVC | Boys | 6.00 | 128 | BCPE | 0.57462 | 1.7485 | 0.12737 | 5.63911 | 1.7485 | 1.4115 |
| FVC | Boys | 6.00 | 129 | BCPE | 0.57462 | 1.7822 | 0.12737 | 5.63911 | 1.7822 | 1.4387 |
| FVC | Boys | 6.00 | 130 | BCPE | 0.57462 | 1.8163 | 0.12737 | 5.63911 | 1.8163 | 1.4662 |
| FVC | Boys | 6.00 | 131 | BCPE | 0.57462 | 1.8508 | 0.12737 | 5.63911 | 1.8508 | 1.4941 |
| FVC | Boys | 6.00 | 132 | BCPE | 0.57462 | 1.8857 | 0.12737 | 5.63911 | 1.8857 | 1.5222 |
| FVC | Girls | 3.00 | 88 | BCPE | 0.56068 | 0.6781 | 0.13772 | 2.57051 | 0.6781 | 0.5335 |
| FVC | Girls | 3.00 | 89 | BCPE | 0.56068 | 0.6941 | 0.13772 | 2.57051 | 0.6941 | 0.5460 |
| FVC | Girls | 3.00 | 90 | BCPE | 0.56068 | 0.7103 | 0.13772 | 2.57051 | 0.7103 | 0.5588 |
| FVC | Girls | 3.00 | 91 | BCPE | 0.56068 | 0.7267 | 0.13772 | 2.57051 | 0.7267 | 0.5717 |
| FVC | Girls | 3.00 | 92 | BCPE | 0.56068 | 0.7433 | 0.13772 | 2.57051 | 0.7433 | 0.5847 |
| FVC | Girls | 3.00 | 93 | BCPE | 0.56068 | 0.7600 | 0.13772 | 2.57051 | 0.7600 | 0.5979 |
| FVC | Girls | 3.00 | 94 | BCPE | 0.56068 | 0.7770 | 0.13772 | 2.57051 | 0.7770 | 0.6112 |
| FVC | Girls | 3.00 | 95 | BCPE | 0.56068 | 0.7942 | 0.13772 | 2.57051 | 0.7942 | 0.6247 |
| FVC | Girls | 3.00 | 96 | BCPE | 0.56068 | 0.8115 | 0.13772 | 2.57051 | 0.8115 | 0.6384 |
| FVC | Girls | 3.00 | 97 | BCPE | 0.56068 | 0.8290 | 0.13772 | 2.57051 | 0.8290 | 0.6522 |
| FVC | Girls | 3.00 | 98 | BCPE | 0.56068 | 0.8468 | 0.13772 | 2.57051 | 0.8468 | 0.6661 |
| FVC | Girls | 3.00 | 99 | BCPE | 0.56068 | 0.8647 | 0.13772 | 2.57051 | 0.8647 | 0.6802 |
| FVC | Girls | 3.00 | 100 | BCPE | 0.56068 | 0.8828 | 0.13772 | 2.57051 | 0.8828 | 0.6945 |
| FVC | Girls | 3.00 | 101 | BCPE | 0.56068 | 0.9011 | 0.13772 | 2.57051 | 0.9011 | 0.7089 |
| FVC | Girls | 3.00 | 102 | BCPE | 0.56068 | 0.9196 | 0.13772 | 2.57051 | 0.9196 | 0.7234 |
| FVC | Girls | 3.00 | 103 | BCPE | 0.56068 | 0.9383 | 0.13772 | 2.57051 | 0.9383 | 0.7381 |
| FVC | Girls | 3.00 | 104 | BCPE | 0.56068 | 0.9572 | 0.13772 | 2.57051 | 0.9572 | 0.7530 |
| FVC | Girls | 3.00 | 105 | BCPE | 0.56068 | 0.9763 | 0.13772 | 2.57051 | 0.9763 | 0.7680 |
| FVC | Girls | 3.00 | 106 | BCPE | 0.56068 | 0.9956 | 0.13772 | 2.57051 | 0.9956 | 0.7832 |
| FVC | Girls | 3.00 | 107 | BCPE | 0.56068 | 1.0151 | 0.13772 | 2.57051 | 1.0151 | 0.7985 |
| FVC | Girls | 3.00 | 108 | BCPE | 0.56068 | 1.0347 | 0.13772 | 2.57051 | 1.0347 | 0.8140 |
| FVC | Girls | 3.00 | 109 | BCPE | 0.56068 | 1.0546 | 0.13772 | 2.57051 | 1.0546 | 0.8296 |
| FVC | Girls | 3.00 | 110 | BCPE | 0.56068 | 1.0747 | 0.13772 | 2.57051 | 1.0747 | 0.8454 |
| FVC | Girls | 3.00 | 111 | BCPE | 0.56068 | 1.0949 | 0.13772 | 2.57051 | 1.0949 | 0.8613 |
| FVC | Girls | 3.00 | 112 | BCPE | 0.56068 | 1.1154 | 0.13772 | 2.57051 | 1.1154 | 0.8774 |
| FVC | Girls | 3.00 | 113 | BCPE | 0.56068 | 1.1360 | 0.13772 | 2.57051 | 1.1360 | 0.8936 |
| FVC | Girls | 3.00 | 114 | BCPE | 0.56068 | 1.1568 | 0.13772 | 2.57051 | 1.1568 | 0.9100 |
| FVC | Girls | 3.00 | 115 | BCPE | 0.56068 | 1.1779 | 0.13772 | 2.57051 | 1.1779 | 0.9266 |
| FVC | Girls | 3.00 | 116 | BCPE | 0.56068 | 1.1991 | 0.13772 | 2.57051 | 1.1991 | 0.9433 |
| FVC | Girls | 3.00 | 117 | BCPE | 0.56068 | 1.2205 | 0.13772 | 2.57051 | 1.2205 | 0.9601 |
| FVC | Girls | 3.00 | 118 | BCPE | 0.56068 | 1.2422 | 0.13772 | 2.57051 | 1.2422 | 0.9771 |
| FVC | Girls | 3.00 | 119 | BCPE | 0.56068 | 1.2640 | 0.13772 | 2.57051 | 1.2640 | 0.9943 |
| FVC | Girls | 3.00 | 120 | BCPE | 0.56068 | 1.2860 | 0.13772 | 2.57051 | 1.2860 | 1.0116 |
| FVC | Girls | 3.00 | 121 | BCPE | 0.56068 | 1.3082 | 0.13772 | 2.57051 | 1.3082 | 1.0291 |
| FVC | Girls | 3.00 | 122 | BCPE | 0.56068 | 1.3306 | 0.13772 | 2.57051 | 1.3306 | 1.0467 |
| FVC | Girls | 3.00 | 123 | BCPE | 0.56068 | 1.3532 | 0.13772 | 2.57051 | 1.3532 | 1.0645 |
| FVC | Girls | 3.00 | 124 | BCPE | 0.56068 | 1.3760 | 0.13772 | 2.57051 | 1.3760 | 1.0824 |
| FVC | Girls | 3.25 | 88 | BCPE | 0.62385 | 0.6860 | 0.13664 | 2.83372 | 0.6860 | 0.5403 |
| FVC | Girls | 3.25 | 89 | BCPE | 0.62385 | 0.7022 | 0.13664 | 2.83372 | 0.7022 | 0.5530 |
| FVC | Girls | 3.25 | 90 | BCPE | 0.62385 | 0.7186 | 0.13664 | 2.83372 | 0.7186 | 0.5659 |
| FVC | Girls | 3.25 | 91 | BCPE | 0.62385 | 0.7351 | 0.13664 | 2.83372 | 0.7351 | 0.5790 |
| FVC | Girls | 3.25 | 92 | BCPE | 0.62385 | 0.7519 | 0.13664 | 2.83372 | 0.7519 | 0.5922 |
| FVC | Girls | 3.25 | 93 | BCPE | 0.62385 | 0.7689 | 0.13664 | 2.83372 | 0.7689 | 0.6055 |
| FVC | Girls | 3.25 | 94 | BCPE | 0.62385 | 0.7860 | 0.13664 | 2.83372 | 0.7860 | 0.6190 |
| FVC | Girls | 3.25 | 95 | BCPE | 0.62385 | 0.8034 | 0.13664 | 2.83372 | 0.8034 | 0.6327 |
| FVC | Girls | 3.25 | 96 | BCPE | 0.62385 | 0.8209 | 0.13664 | 2.83372 | 0.8209 | 0.6465 |
| FVC | Girls | 3.25 | 97 | BCPE | 0.62385 | 0.8387 | 0.13664 | 2.83372 | 0.8387 | 0.6605 |
| FVC | Girls | 3.25 | 98 | BCPE | 0.62385 | 0.8566 | 0.13664 | 2.83372 | 0.8566 | 0.6746 |
| FVC | Girls | 3.25 | 99 | BCPE | 0.62385 | 0.8747 | 0.13664 | 2.83372 | 0.8747 | 0.6889 |
| FVC | Girls | 3.25 | 100 | BCPE | 0.62385 | 0.8931 | 0.13664 | 2.83372 | 0.8931 | 0.7033 |
| FVC | Girls | 3.25 | 101 | BCPE | 0.62385 | 0.9116 | 0.13664 | 2.83372 | 0.9116 | 0.7179 |
| FVC | Girls | 3.25 | 102 | BCPE | 0.62385 | 0.9303 | 0.13664 | 2.83372 | 0.9303 | 0.7327 |
| FVC | Girls | 3.25 | 103 | BCPE | 0.62385 | 0.9492 | 0.13664 | 2.83372 | 0.9492 | 0.7476 |
| FVC | Girls | 3.25 | 104 | BCPE | 0.62385 | 0.9683 | 0.13664 | 2.83372 | 0.9683 | 0.7626 |
| FVC | Girls | 3.25 | 105 | BCPE | 0.62385 | 0.9876 | 0.13664 | 2.83372 | 0.9876 | 0.7778 |
| FVC | Girls | 3.25 | 106 | BCPE | 0.62385 | 1.0072 | 0.13664 | 2.83372 | 1.0072 | 0.7932 |
| FVC | Girls | 3.25 | 107 | BCPE | 0.62385 | 1.0269 | 0.13664 | 2.83372 | 1.0269 | 0.8087 |
| FVC | Girls | 3.25 | 108 | BCPE | 0.62385 | 1.0468 | 0.13664 | 2.83372 | 1.0468 | 0.8244 |
| FVC | Girls | 3.25 | 109 | BCPE | 0.62385 | 1.0668 | 0.13664 | 2.83372 | 1.0668 | 0.8402 |
| FVC | Girls | 3.25 | 110 | BCPE | 0.62385 | 1.0871 | 0.13664 | 2.83372 | 1.0871 | 0.8562 |
| FVC | Girls | 3.25 | 111 | BCPE | 0.62385 | 1.1076 | 0.13664 | 2.83372 | 1.1076 | 0.8723 |
| FVC | Girls | 3.25 | 112 | BCPE | 0.62385 | 1.1283 | 0.13664 | 2.83372 | 1.1283 | 0.8886 |
| FVC | Girls | 3.25 | 113 | BCPE | 0.62385 | 1.1492 | 0.13664 | 2.83372 | 1.1492 | 0.9051 |
| FVC | Girls | 3.25 | 114 | BCPE | 0.62385 | 1.1703 | 0.13664 | 2.83372 | 1.1703 | 0.9217 |
| FVC | Girls | 3.25 | 115 | BCPE | 0.62385 | 1.1916 | 0.13664 | 2.83372 | 1.1916 | 0.9384 |
| FVC | Girls | 3.25 | 116 | BCPE | 0.62385 | 1.2130 | 0.13664 | 2.83372 | 1.2130 | 0.9553 |
| FVC | Girls | 3.25 | 117 | BCPE | 0.62385 | 1.2347 | 0.13664 | 2.83372 | 1.2347 | 0.9724 |
| FVC | Girls | 3.25 | 118 | BCPE | 0.62385 | 1.2566 | 0.13664 | 2.83372 | 1.2566 | 0.9896 |
| FVC | Girls | 3.25 | 119 | BCPE | 0.62385 | 1.2787 | 0.13664 | 2.83372 | 1.2787 | 1.0070 |
| FVC | Girls | 3.25 | 120 | BCPE | 0.62385 | 1.3009 | 0.13664 | 2.83372 | 1.3009 | 1.0246 |
| FVC | Girls | 3.25 | 121 | BCPE | 0.62385 | 1.3234 | 0.13664 | 2.83372 | 1.3234 | 1.0422 |
| FVC | Girls | 3.25 | 122 | BCPE | 0.62385 | 1.3461 | 0.13664 | 2.83372 | 1.3461 | 1.0601 |
| FVC | Girls | 3.25 | 123 | BCPE | 0.62385 | 1.3689 | 0.13664 | 2.83372 | 1.3689 | 1.0781 |
| FVC | Girls | 3.25 | 124 | BCPE | 0.62385 | 1.3920 | 0.13664 | 2.83372 | 1.3920 | 1.0963 |
| FVC | Girls | 3.50 | 88 | BCPE | 0.68233 | 0.6934 | 0.13565 | 3.10139 | 0.6934 | 0.5467 |
| FVC | Girls | 3.50 | 89 | BCPE | 0.68233 | 0.7098 | 0.13565 | 3.10139 | 0.7098 | 0.5596 |
| FVC | Girls | 3.50 | 90 | BCPE | 0.68233 | 0.7263 | 0.13565 | 3.10139 | 0.7263 | 0.5726 |
| FVC | Girls | 3.50 | 91 | BCPE | 0.68233 | 0.7431 | 0.13565 | 3.10139 | 0.7431 | 0.5858 |
| FVC | Girls | 3.50 | 92 | BCPE | 0.68233 | 0.7600 | 0.13565 | 3.10139 | 0.7600 | 0.5992 |
| FVC | Girls | 3.50 | 93 | BCPE | 0.68233 | 0.7772 | 0.13565 | 3.10139 | 0.7772 | 0.6127 |
| FVC | Girls | 3.50 | 94 | BCPE | 0.68233 | 0.7945 | 0.13565 | 3.10139 | 0.7945 | 0.6264 |
| FVC | Girls | 3.50 | 95 | BCPE | 0.68233 | 0.8120 | 0.13565 | 3.10139 | 0.8120 | 0.6402 |
| FVC | Girls | 3.50 | 96 | BCPE | 0.68233 | 0.8298 | 0.13565 | 3.10139 | 0.8298 | 0.6542 |
| FVC | Girls | 3.50 | 97 | BCPE | 0.68233 | 0.8477 | 0.13565 | 3.10139 | 0.8477 | 0.6683 |
| FVC | Girls | 3.50 | 98 | BCPE | 0.68233 | 0.8658 | 0.13565 | 3.10139 | 0.8658 | 0.6826 |
| FVC | Girls | 3.50 | 99 | BCPE | 0.68233 | 0.8842 | 0.13565 | 3.10139 | 0.8842 | 0.6971 |
| FVC | Girls | 3.50 | 100 | BCPE | 0.68233 | 0.9027 | 0.13565 | 3.10139 | 0.9027 | 0.7117 |
| FVC | Girls | 3.50 | 101 | BCPE | 0.68233 | 0.9214 | 0.13565 | 3.10139 | 0.9214 | 0.7265 |
| FVC | Girls | 3.50 | 102 | BCPE | 0.68233 | 0.9403 | 0.13565 | 3.10139 | 0.9403 | 0.7414 |
| FVC | Girls | 3.50 | 103 | BCPE | 0.68233 | 0.9595 | 0.13565 | 3.10139 | 0.9595 | 0.7565 |
| FVC | Girls | 3.50 | 104 | BCPE | 0.68233 | 0.9788 | 0.13565 | 3.10139 | 0.9788 | 0.7717 |
| FVC | Girls | 3.50 | 105 | BCPE | 0.68233 | 0.9983 | 0.13565 | 3.10139 | 0.9983 | 0.7871 |
| FVC | Girls | 3.50 | 106 | BCPE | 0.68233 | 1.0180 | 0.13565 | 3.10139 | 1.0180 | 0.8026 |
| FVC | Girls | 3.50 | 107 | BCPE | 0.68233 | 1.0379 | 0.13565 | 3.10139 | 1.0379 | 0.8183 |
| FVC | Girls | 3.50 | 108 | BCPE | 0.68233 | 1.0580 | 0.13565 | 3.10139 | 1.0580 | 0.8342 |
| FVC | Girls | 3.50 | 109 | BCPE | 0.68233 | 1.0784 | 0.13565 | 3.10139 | 1.0784 | 0.8502 |
| FVC | Girls | 3.50 | 110 | BCPE | 0.68233 | 1.0989 | 0.13565 | 3.10139 | 1.0989 | 0.8664 |
| FVC | Girls | 3.50 | 111 | BCPE | 0.68233 | 1.1196 | 0.13565 | 3.10139 | 1.1196 | 0.8827 |
| FVC | Girls | 3.50 | 112 | BCPE | 0.68233 | 1.1405 | 0.13565 | 3.10139 | 1.1405 | 0.8992 |
| FVC | Girls | 3.50 | 113 | BCPE | 0.68233 | 1.1616 | 0.13565 | 3.10139 | 1.1616 | 0.9158 |
| FVC | Girls | 3.50 | 114 | BCPE | 0.68233 | 1.1829 | 0.13565 | 3.10139 | 1.1829 | 0.9326 |
| FVC | Girls | 3.50 | 115 | BCPE | 0.68233 | 1.2044 | 0.13565 | 3.10139 | 1.2044 | 0.9496 |
| FVC | Girls | 3.50 | 116 | BCPE | 0.68233 | 1.2261 | 0.13565 | 3.10139 | 1.2261 | 0.9667 |
| FVC | Girls | 3.50 | 117 | BCPE | 0.68233 | 1.2480 | 0.13565 | 3.10139 | 1.2480 | 0.9840 |
| FVC | Girls | 3.50 | 118 | BCPE | 0.68233 | 1.2701 | 0.13565 | 3.10139 | 1.2701 | 1.0014 |
| FVC | Girls | 3.50 | 119 | BCPE | 0.68233 | 1.2924 | 0.13565 | 3.10139 | 1.2924 | 1.0190 |
| FVC | Girls | 3.50 | 120 | BCPE | 0.68233 | 1.3150 | 0.13565 | 3.10139 | 1.3150 | 1.0367 |
| FVC | Girls | 3.50 | 121 | BCPE | 0.68233 | 1.3377 | 0.13565 | 3.10139 | 1.3377 | 1.0546 |
| FVC | Girls | 3.50 | 122 | BCPE | 0.68233 | 1.3606 | 0.13565 | 3.10139 | 1.3606 | 1.0727 |
| FVC | Girls | 3.50 | 123 | BCPE | 0.68233 | 1.3837 | 0.13565 | 3.10139 | 1.3837 | 1.0909 |
| FVC | Girls | 3.50 | 124 | BCPE | 0.68233 | 1.4070 | 0.13565 | 3.10139 | 1.4070 | 1.1093 |
| FVC | Girls | 3.75 | 88 | BCPE | 0.73678 | 0.7004 | 0.13474 | 3.37327 | 0.7004 | 0.5528 |
| FVC | Girls | 3.75 | 89 | BCPE | 0.73678 | 0.7169 | 0.13474 | 3.37327 | 0.7169 | 0.5658 |
| FVC | Girls | 3.75 | 90 | BCPE | 0.73678 | 0.7336 | 0.13474 | 3.37327 | 0.7336 | 0.5790 |
| FVC | Girls | 3.75 | 91 | BCPE | 0.73678 | 0.7506 | 0.13474 | 3.37327 | 0.7506 | 0.5924 |
| FVC | Girls | 3.75 | 92 | BCPE | 0.73678 | 0.7677 | 0.13474 | 3.37327 | 0.7677 | 0.6059 |
| FVC | Girls | 3.75 | 93 | BCPE | 0.73678 | 0.7850 | 0.13474 | 3.37327 | 0.7850 | 0.6195 |
| FVC | Girls | 3.75 | 94 | BCPE | 0.73678 | 0.8025 | 0.13474 | 3.37327 | 0.8025 | 0.6334 |
| FVC | Girls | 3.75 | 95 | BCPE | 0.73678 | 0.8202 | 0.13474 | 3.37327 | 0.8202 | 0.6473 |
| FVC | Girls | 3.75 | 96 | BCPE | 0.73678 | 0.8381 | 0.13474 | 3.37327 | 0.8381 | 0.6615 |
| FVC | Girls | 3.75 | 97 | BCPE | 0.73678 | 0.8563 | 0.13474 | 3.37327 | 0.8563 | 0.6758 |
| FVC | Girls | 3.75 | 98 | BCPE | 0.73678 | 0.8746 | 0.13474 | 3.37327 | 0.8746 | 0.6902 |
| FVC | Girls | 3.75 | 99 | BCPE | 0.73678 | 0.8931 | 0.13474 | 3.37327 | 0.8931 | 0.7048 |
| FVC | Girls | 3.75 | 100 | BCPE | 0.73678 | 0.9118 | 0.13474 | 3.37327 | 0.9118 | 0.7196 |
| FVC | Girls | 3.75 | 101 | BCPE | 0.73678 | 0.9307 | 0.13474 | 3.37327 | 0.9307 | 0.7345 |
| FVC | Girls | 3.75 | 102 | BCPE | 0.73678 | 0.9498 | 0.13474 | 3.37327 | 0.9498 | 0.7496 |
| FVC | Girls | 3.75 | 103 | BCPE | 0.73678 | 0.9691 | 0.13474 | 3.37327 | 0.9691 | 0.7649 |
| FVC | Girls | 3.75 | 104 | BCPE | 0.73678 | 0.9886 | 0.13474 | 3.37327 | 0.9886 | 0.7803 |
| FVC | Girls | 3.75 | 105 | BCPE | 0.73678 | 1.0084 | 0.13474 | 3.37327 | 1.0084 | 0.7958 |
| FVC | Girls | 3.75 | 106 | BCPE | 0.73678 | 1.0283 | 0.13474 | 3.37327 | 1.0283 | 0.8115 |
| FVC | Girls | 3.75 | 107 | BCPE | 0.73678 | 1.0484 | 0.13474 | 3.37327 | 1.0484 | 0.8274 |
| FVC | Girls | 3.75 | 108 | BCPE | 0.73678 | 1.0687 | 0.13474 | 3.37327 | 1.0687 | 0.8435 |
| FVC | Girls | 3.75 | 109 | BCPE | 0.73678 | 1.0892 | 0.13474 | 3.37327 | 1.0892 | 0.8596 |
| FVC | Girls | 3.75 | 110 | BCPE | 0.73678 | 1.1099 | 0.13474 | 3.37327 | 1.1099 | 0.8760 |
| FVC | Girls | 3.75 | 111 | BCPE | 0.73678 | 1.1309 | 0.13474 | 3.37327 | 1.1309 | 0.8925 |
| FVC | Girls | 3.75 | 112 | BCPE | 0.73678 | 1.1520 | 0.13474 | 3.37327 | 1.1520 | 0.9092 |
| FVC | Girls | 3.75 | 113 | BCPE | 0.73678 | 1.1733 | 0.13474 | 3.37327 | 1.1733 | 0.9260 |
| FVC | Girls | 3.75 | 114 | BCPE | 0.73678 | 1.1948 | 0.13474 | 3.37327 | 1.1948 | 0.9430 |
| FVC | Girls | 3.75 | 115 | BCPE | 0.73678 | 1.2166 | 0.13474 | 3.37327 | 1.2166 | 0.9601 |
| FVC | Girls | 3.75 | 116 | BCPE | 0.73678 | 1.2385 | 0.13474 | 3.37327 | 1.2385 | 0.9774 |
| FVC | Girls | 3.75 | 117 | BCPE | 0.73678 | 1.2606 | 0.13474 | 3.37327 | 1.2606 | 0.9949 |
| FVC | Girls | 3.75 | 118 | BCPE | 0.73678 | 1.2829 | 0.13474 | 3.37327 | 1.2829 | 1.0125 |
| FVC | Girls | 3.75 | 119 | BCPE | 0.73678 | 1.3055 | 0.13474 | 3.37327 | 1.3055 | 1.0303 |
| FVC | Girls | 3.75 | 120 | BCPE | 0.73678 | 1.3282 | 0.13474 | 3.37327 | 1.3282 | 1.0483 |
| FVC | Girls | 3.75 | 121 | BCPE | 0.73678 | 1.3512 | 0.13474 | 3.37327 | 1.3512 | 1.0664 |
| FVC | Girls | 3.75 | 122 | BCPE | 0.73678 | 1.3743 | 0.13474 | 3.37327 | 1.3743 | 1.0846 |
| FVC | Girls | 3.75 | 123 | BCPE | 0.73678 | 1.3976 | 0.13474 | 3.37327 | 1.3976 | 1.1031 |
| FVC | Girls | 3.75 | 124 | BCPE | 0.73678 | 1.4212 | 0.13474 | 3.37327 | 1.4212 | 1.1216 |
| FVC | Girls | 4.00 | 88 | BCPE | 0.78771 | 0.7070 | 0.13389 | 3.64912 | 0.7070 | 0.5586 |
| FVC | Girls | 4.00 | 89 | BCPE | 0.78771 | 0.7237 | 0.13389 | 3.64912 | 0.7237 | 0.5717 |
| FVC | Girls | 4.00 | 90 | BCPE | 0.78771 | 0.7406 | 0.13389 | 3.64912 | 0.7406 | 0.5851 |
| FVC | Girls | 4.00 | 91 | BCPE | 0.78771 | 0.7577 | 0.13389 | 3.64912 | 0.7577 | 0.5986 |
| FVC | Girls | 4.00 | 92 | BCPE | 0.78771 | 0.7750 | 0.13389 | 3.64912 | 0.7750 | 0.6122 |
| FVC | Girls | 4.00 | 93 | BCPE | 0.78771 | 0.7924 | 0.13389 | 3.64912 | 0.7924 | 0.6260 |
| FVC | Girls | 4.00 | 94 | BCPE | 0.78771 | 0.8101 | 0.13389 | 3.64912 | 0.8101 | 0.6400 |
| FVC | Girls | 4.00 | 95 | BCPE | 0.78771 | 0.8280 | 0.13389 | 3.64912 | 0.8280 | 0.6541 |
| FVC | Girls | 4.00 | 96 | BCPE | 0.78771 | 0.8461 | 0.13389 | 3.64912 | 0.8461 | 0.6684 |
| FVC | Girls | 4.00 | 97 | BCPE | 0.78771 | 0.8644 | 0.13389 | 3.64912 | 0.8644 | 0.6829 |
| FVC | Girls | 4.00 | 98 | BCPE | 0.78771 | 0.8829 | 0.13389 | 3.64912 | 0.8829 | 0.6975 |
| FVC | Girls | 4.00 | 99 | BCPE | 0.78771 | 0.9016 | 0.13389 | 3.64912 | 0.9016 | 0.7122 |
| FVC | Girls | 4.00 | 100 | BCPE | 0.78771 | 0.9204 | 0.13389 | 3.64912 | 0.9204 | 0.7271 |
| FVC | Girls | 4.00 | 101 | BCPE | 0.78771 | 0.9395 | 0.13389 | 3.64912 | 0.9395 | 0.7422 |
| FVC | Girls | 4.00 | 102 | BCPE | 0.78771 | 0.9588 | 0.13389 | 3.64912 | 0.9588 | 0.7575 |
| FVC | Girls | 4.00 | 103 | BCPE | 0.78771 | 0.9783 | 0.13389 | 3.64912 | 0.9783 | 0.7729 |
| FVC | Girls | 4.00 | 104 | BCPE | 0.78771 | 0.9980 | 0.13389 | 3.64912 | 0.9980 | 0.7884 |
| FVC | Girls | 4.00 | 105 | BCPE | 0.78771 | 1.0179 | 0.13389 | 3.64912 | 1.0179 | 0.8042 |
| FVC | Girls | 4.00 | 106 | BCPE | 0.78771 | 1.0380 | 0.13389 | 3.64912 | 1.0380 | 0.8200 |
| FVC | Girls | 4.00 | 107 | BCPE | 0.78771 | 1.0583 | 0.13389 | 3.64912 | 1.0583 | 0.8361 |
| FVC | Girls | 4.00 | 108 | BCPE | 0.78771 | 1.0788 | 0.13389 | 3.64912 | 1.0788 | 0.8523 |
| FVC | Girls | 4.00 | 109 | BCPE | 0.78771 | 1.0996 | 0.13389 | 3.64912 | 1.0996 | 0.8686 |
| FVC | Girls | 4.00 | 110 | BCPE | 0.78771 | 1.1205 | 0.13389 | 3.64912 | 1.1205 | 0.8852 |
| FVC | Girls | 4.00 | 111 | BCPE | 0.78771 | 1.1416 | 0.13389 | 3.64912 | 1.1416 | 0.9019 |
| FVC | Girls | 4.00 | 112 | BCPE | 0.78771 | 1.1629 | 0.13389 | 3.64912 | 1.1629 | 0.9187 |
| FVC | Girls | 4.00 | 113 | BCPE | 0.78771 | 1.1844 | 0.13389 | 3.64912 | 1.1844 | 0.9357 |
| FVC | Girls | 4.00 | 114 | BCPE | 0.78771 | 1.2062 | 0.13389 | 3.64912 | 1.2062 | 0.9529 |
| FVC | Girls | 4.00 | 115 | BCPE | 0.78771 | 1.2281 | 0.13389 | 3.64912 | 1.2281 | 0.9702 |
| FVC | Girls | 4.00 | 116 | BCPE | 0.78771 | 1.2502 | 0.13389 | 3.64912 | 1.2502 | 0.9877 |
| FVC | Girls | 4.00 | 117 | BCPE | 0.78771 | 1.2726 | 0.13389 | 3.64912 | 1.2726 | 1.0053 |
| FVC | Girls | 4.00 | 118 | BCPE | 0.78771 | 1.2951 | 0.13389 | 3.64912 | 1.2951 | 1.0231 |
| FVC | Girls | 4.00 | 119 | BCPE | 0.78771 | 1.3179 | 0.13389 | 3.64912 | 1.3179 | 1.0411 |
| FVC | Girls | 4.00 | 120 | BCPE | 0.78771 | 1.3408 | 0.13389 | 3.64912 | 1.3408 | 1.0592 |
| FVC | Girls | 4.00 | 121 | BCPE | 0.78771 | 1.3640 | 0.13389 | 3.64912 | 1.3640 | 1.0775 |
| FVC | Girls | 4.00 | 122 | BCPE | 0.78771 | 1.3873 | 0.13389 | 3.64912 | 1.3873 | 1.0960 |
| FVC | Girls | 4.00 | 123 | BCPE | 0.78771 | 1.4109 | 0.13389 | 3.64912 | 1.4109 | 1.1146 |
| FVC | Girls | 4.00 | 124 | BCPE | 0.78771 | 1.4347 | 0.13389 | 3.64912 | 1.4347 | 1.1334 |
| FVC | Girls | 4.25 | 88 | BCPE | 0.83555 | 0.7134 | 0.13310 | 3.92877 | 0.7134 | 0.5641 |
| FVC | Girls | 4.25 | 89 | BCPE | 0.83555 | 0.7302 | 0.13310 | 3.92877 | 0.7302 | 0.5774 |
| FVC | Girls | 4.25 | 90 | BCPE | 0.83555 | 0.7472 | 0.13310 | 3.92877 | 0.7472 | 0.5908 |
| FVC | Girls | 4.25 | 91 | BCPE | 0.83555 | 0.7645 | 0.13310 | 3.92877 | 0.7645 | 0.6045 |
| FVC | Girls | 4.25 | 92 | BCPE | 0.83555 | 0.7819 | 0.13310 | 3.92877 | 0.7819 | 0.6183 |
| FVC | Girls | 4.25 | 93 | BCPE | 0.83555 | 0.7995 | 0.13310 | 3.92877 | 0.7995 | 0.6322 |
| FVC | Girls | 4.25 | 94 | BCPE | 0.83555 | 0.8174 | 0.13310 | 3.92877 | 0.8174 | 0.6463 |
| FVC | Girls | 4.25 | 95 | BCPE | 0.83555 | 0.8354 | 0.13310 | 3.92877 | 0.8354 | 0.6606 |
| FVC | Girls | 4.25 | 96 | BCPE | 0.83555 | 0.8537 | 0.13310 | 3.92877 | 0.8537 | 0.6750 |
| FVC | Girls | 4.25 | 97 | BCPE | 0.83555 | 0.8721 | 0.13310 | 3.92877 | 0.8721 | 0.6896 |
| FVC | Girls | 4.25 | 98 | BCPE | 0.83555 | 0.8908 | 0.13310 | 3.92877 | 0.8908 | 0.7043 |
| FVC | Girls | 4.25 | 99 | BCPE | 0.83555 | 0.9096 | 0.13310 | 3.92877 | 0.9096 | 0.7192 |
| FVC | Girls | 4.25 | 100 | BCPE | 0.83555 | 0.9287 | 0.13310 | 3.92877 | 0.9287 | 0.7343 |
| FVC | Girls | 4.25 | 101 | BCPE | 0.83555 | 0.9480 | 0.13310 | 3.92877 | 0.9480 | 0.7496 |
| FVC | Girls | 4.25 | 102 | BCPE | 0.83555 | 0.9674 | 0.13310 | 3.92877 | 0.9674 | 0.7649 |
| FVC | Girls | 4.25 | 103 | BCPE | 0.83555 | 0.9871 | 0.13310 | 3.92877 | 0.9871 | 0.7805 |
| FVC | Girls | 4.25 | 104 | BCPE | 0.83555 | 1.0070 | 0.13310 | 3.92877 | 1.0070 | 0.7962 |
| FVC | Girls | 4.25 | 105 | BCPE | 0.83555 | 1.0271 | 0.13310 | 3.92877 | 1.0271 | 0.8121 |
| FVC | Girls | 4.25 | 106 | BCPE | 0.83555 | 1.0473 | 0.13310 | 3.92877 | 1.0473 | 0.8281 |
| FVC | Girls | 4.25 | 107 | BCPE | 0.83555 | 1.0678 | 0.13310 | 3.92877 | 1.0678 | 0.8443 |
| FVC | Girls | 4.25 | 108 | BCPE | 0.83555 | 1.0885 | 0.13310 | 3.92877 | 1.0885 | 0.8607 |
| FVC | Girls | 4.25 | 109 | BCPE | 0.83555 | 1.1094 | 0.13310 | 3.92877 | 1.1094 | 0.8772 |
| FVC | Girls | 4.25 | 110 | BCPE | 0.83555 | 1.1305 | 0.13310 | 3.92877 | 1.1305 | 0.8939 |
| FVC | Girls | 4.25 | 111 | BCPE | 0.83555 | 1.1518 | 0.13310 | 3.92877 | 1.1518 | 0.9108 |
| FVC | Girls | 4.25 | 112 | BCPE | 0.83555 | 1.1733 | 0.13310 | 3.92877 | 1.1733 | 0.9278 |
| FVC | Girls | 4.25 | 113 | BCPE | 0.83555 | 1.1951 | 0.13310 | 3.92877 | 1.1951 | 0.9449 |
| FVC | Girls | 4.25 | 114 | BCPE | 0.83555 | 1.2170 | 0.13310 | 3.92877 | 1.2170 | 0.9623 |
| FVC | Girls | 4.25 | 115 | BCPE | 0.83555 | 1.2391 | 0.13310 | 3.92877 | 1.2391 | 0.9798 |
| FVC | Girls | 4.25 | 116 | BCPE | 0.83555 | 1.2614 | 0.13310 | 3.92877 | 1.2614 | 0.9974 |
| FVC | Girls | 4.25 | 117 | BCPE | 0.83555 | 1.2840 | 0.13310 | 3.92877 | 1.2840 | 1.0152 |
| FVC | Girls | 4.25 | 118 | BCPE | 0.83555 | 1.3067 | 0.13310 | 3.92877 | 1.3067 | 1.0332 |
| FVC | Girls | 4.25 | 119 | BCPE | 0.83555 | 1.3297 | 0.13310 | 3.92877 | 1.3297 | 1.0514 |
| FVC | Girls | 4.25 | 120 | BCPE | 0.83555 | 1.3528 | 0.13310 | 3.92877 | 1.3528 | 1.0697 |
| FVC | Girls | 4.25 | 121 | BCPE | 0.83555 | 1.3762 | 0.13310 | 3.92877 | 1.3762 | 1.0882 |
| FVC | Girls | 4.25 | 122 | BCPE | 0.83555 | 1.3998 | 0.13310 | 3.92877 | 1.3998 | 1.1068 |
| FVC | Girls | 4.25 | 123 | BCPE | 0.83555 | 1.4235 | 0.13310 | 3.92877 | 1.4235 | 1.1256 |
| FVC | Girls | 4.25 | 124 | BCPE | 0.83555 | 1.4475 | 0.13310 | 3.92877 | 1.4475 | 1.1446 |
| FVC | Girls | 4.50 | 88 | BCPE | 0.88065 | 0.7194 | 0.13236 | 4.21202 | 0.7194 | 0.5693 |
| FVC | Girls | 4.50 | 89 | BCPE | 0.88065 | 0.7364 | 0.13236 | 4.21202 | 0.7364 | 0.5828 |
| FVC | Girls | 4.50 | 90 | BCPE | 0.88065 | 0.7536 | 0.13236 | 4.21202 | 0.7536 | 0.5963 |
| FVC | Girls | 4.50 | 91 | BCPE | 0.88065 | 0.7710 | 0.13236 | 4.21202 | 0.7710 | 0.6101 |
| FVC | Girls | 4.50 | 92 | BCPE | 0.88065 | 0.7885 | 0.13236 | 4.21202 | 0.7885 | 0.6240 |
| FVC | Girls | 4.50 | 93 | BCPE | 0.88065 | 0.8063 | 0.13236 | 4.21202 | 0.8063 | 0.6381 |
| FVC | Girls | 4.50 | 94 | BCPE | 0.88065 | 0.8243 | 0.13236 | 4.21202 | 0.8243 | 0.6523 |
| FVC | Girls | 4.50 | 95 | BCPE | 0.88065 | 0.8425 | 0.13236 | 4.21202 | 0.8425 | 0.6667 |
| FVC | Girls | 4.50 | 96 | BCPE | 0.88065 | 0.8609 | 0.13236 | 4.21202 | 0.8609 | 0.6813 |
| FVC | Girls | 4.50 | 97 | BCPE | 0.88065 | 0.8795 | 0.13236 | 4.21202 | 0.8795 | 0.6960 |
| FVC | Girls | 4.50 | 98 | BCPE | 0.88065 | 0.8983 | 0.13236 | 4.21202 | 0.8983 | 0.7109 |
| FVC | Girls | 4.50 | 99 | BCPE | 0.88065 | 0.9173 | 0.13236 | 4.21202 | 0.9173 | 0.7259 |
| FVC | Girls | 4.50 | 100 | BCPE | 0.88065 | 0.9366 | 0.13236 | 4.21202 | 0.9366 | 0.7412 |
| FVC | Girls | 4.50 | 101 | BCPE | 0.88065 | 0.9560 | 0.13236 | 4.21202 | 0.9560 | 0.7565 |
| FVC | Girls | 4.50 | 102 | BCPE | 0.88065 | 0.9756 | 0.13236 | 4.21202 | 0.9756 | 0.7721 |
| FVC | Girls | 4.50 | 103 | BCPE | 0.88065 | 0.9955 | 0.13236 | 4.21202 | 0.9955 | 0.7878 |
| FVC | Girls | 4.50 | 104 | BCPE | 0.88065 | 1.0155 | 0.13236 | 4.21202 | 1.0155 | 0.8036 |
| FVC | Girls | 4.50 | 105 | BCPE | 0.88065 | 1.0358 | 0.13236 | 4.21202 | 1.0358 | 0.8197 |
| FVC | Girls | 4.50 | 106 | BCPE | 0.88065 | 1.0562 | 0.13236 | 4.21202 | 1.0562 | 0.8358 |
| FVC | Girls | 4.50 | 107 | BCPE | 0.88065 | 1.0769 | 0.13236 | 4.21202 | 1.0769 | 0.8522 |
| FVC | Girls | 4.50 | 108 | BCPE | 0.88065 | 1.0977 | 0.13236 | 4.21202 | 1.0977 | 0.8687 |
| FVC | Girls | 4.50 | 109 | BCPE | 0.88065 | 1.1188 | 0.13236 | 4.21202 | 1.1188 | 0.8854 |
| FVC | Girls | 4.50 | 110 | BCPE | 0.88065 | 1.1401 | 0.13236 | 4.21202 | 1.1401 | 0.9022 |
| FVC | Girls | 4.50 | 111 | BCPE | 0.88065 | 1.1616 | 0.13236 | 4.21202 | 1.1616 | 0.9192 |
| FVC | Girls | 4.50 | 112 | BCPE | 0.88065 | 1.1833 | 0.13236 | 4.21202 | 1.1833 | 0.9364 |
| FVC | Girls | 4.50 | 113 | BCPE | 0.88065 | 1.2052 | 0.13236 | 4.21202 | 1.2052 | 0.9537 |
| FVC | Girls | 4.50 | 114 | BCPE | 0.88065 | 1.2273 | 0.13236 | 4.21202 | 1.2273 | 0.9712 |
| FVC | Girls | 4.50 | 115 | BCPE | 0.88065 | 1.2496 | 0.13236 | 4.21202 | 1.2496 | 0.9889 |
| FVC | Girls | 4.50 | 116 | BCPE | 0.88065 | 1.2721 | 0.13236 | 4.21202 | 1.2721 | 1.0067 |
| FVC | Girls | 4.50 | 117 | BCPE | 0.88065 | 1.2949 | 0.13236 | 4.21202 | 1.2949 | 1.0247 |
| FVC | Girls | 4.50 | 118 | BCPE | 0.88065 | 1.3178 | 0.13236 | 4.21202 | 1.3178 | 1.0429 |
| FVC | Girls | 4.50 | 119 | BCPE | 0.88065 | 1.3410 | 0.13236 | 4.21202 | 1.3410 | 1.0612 |
| FVC | Girls | 4.50 | 120 | BCPE | 0.88065 | 1.3643 | 0.13236 | 4.21202 | 1.3643 | 1.0796 |
| FVC | Girls | 4.50 | 121 | BCPE | 0.88065 | 1.3879 | 0.13236 | 4.21202 | 1.3879 | 1.0983 |
| FVC | Girls | 4.50 | 122 | BCPE | 0.88065 | 1.4116 | 0.13236 | 4.21202 | 1.4116 | 1.1171 |
| FVC | Girls | 4.50 | 123 | BCPE | 0.88065 | 1.4356 | 0.13236 | 4.21202 | 1.4356 | 1.1361 |
| FVC | Girls | 4.50 | 124 | BCPE | 0.88065 | 1.4598 | 0.13236 | 4.21202 | 1.4598 | 1.1552 |
| FVC | Girls | 4.75 | 88 | BCPE | 0.92332 | 0.7252 | 0.13166 | 4.49872 | 0.7252 | 0.5743 |
| FVC | Girls | 4.75 | 89 | BCPE | 0.92332 | 0.7423 | 0.13166 | 4.49872 | 0.7423 | 0.5879 |
| FVC | Girls | 4.75 | 90 | BCPE | 0.92332 | 0.7596 | 0.13166 | 4.49872 | 0.7596 | 0.6016 |
| FVC | Girls | 4.75 | 91 | BCPE | 0.92332 | 0.7771 | 0.13166 | 4.49872 | 0.7771 | 0.6155 |
| FVC | Girls | 4.75 | 92 | BCPE | 0.92332 | 0.7949 | 0.13166 | 4.49872 | 0.7949 | 0.6295 |
| FVC | Girls | 4.75 | 93 | BCPE | 0.92332 | 0.8128 | 0.13166 | 4.49872 | 0.8128 | 0.6437 |
| FVC | Girls | 4.75 | 94 | BCPE | 0.92332 | 0.8309 | 0.13166 | 4.49872 | 0.8309 | 0.6581 |
| FVC | Girls | 4.75 | 95 | BCPE | 0.92332 | 0.8493 | 0.13166 | 4.49872 | 0.8493 | 0.6726 |
| FVC | Girls | 4.75 | 96 | BCPE | 0.92332 | 0.8678 | 0.13166 | 4.49872 | 0.8678 | 0.6873 |
| FVC | Girls | 4.75 | 97 | BCPE | 0.92332 | 0.8866 | 0.13166 | 4.49872 | 0.8866 | 0.7021 |
| FVC | Girls | 4.75 | 98 | BCPE | 0.92332 | 0.9055 | 0.13166 | 4.49872 | 0.9055 | 0.7171 |
| FVC | Girls | 4.75 | 99 | BCPE | 0.92332 | 0.9247 | 0.13166 | 4.49872 | 0.9247 | 0.7323 |
| FVC | Girls | 4.75 | 100 | BCPE | 0.92332 | 0.9441 | 0.13166 | 4.49872 | 0.9441 | 0.7477 |
| FVC | Girls | 4.75 | 101 | BCPE | 0.92332 | 0.9637 | 0.13166 | 4.49872 | 0.9637 | 0.7632 |
| FVC | Girls | 4.75 | 102 | BCPE | 0.92332 | 0.9834 | 0.13166 | 4.49872 | 0.9834 | 0.7788 |
| FVC | Girls | 4.75 | 103 | BCPE | 0.92332 | 1.0034 | 0.13166 | 4.49872 | 1.0034 | 0.7947 |
| FVC | Girls | 4.75 | 104 | BCPE | 0.92332 | 1.0236 | 0.13166 | 4.49872 | 1.0236 | 0.8107 |
| FVC | Girls | 4.75 | 105 | BCPE | 0.92332 | 1.0441 | 0.13166 | 4.49872 | 1.0441 | 0.8268 |
| FVC | Girls | 4.75 | 106 | BCPE | 0.92332 | 1.0647 | 0.13166 | 4.49872 | 1.0647 | 0.8432 |
| FVC | Girls | 4.75 | 107 | BCPE | 0.92332 | 1.0855 | 0.13166 | 4.49872 | 1.0855 | 0.8597 |
| FVC | Girls | 4.75 | 108 | BCPE | 0.92332 | 1.1065 | 0.13166 | 4.49872 | 1.1065 | 0.8763 |
| FVC | Girls | 4.75 | 109 | BCPE | 0.92332 | 1.1278 | 0.13166 | 4.49872 | 1.1278 | 0.8932 |
| FVC | Girls | 4.75 | 110 | BCPE | 0.92332 | 1.1492 | 0.13166 | 4.49872 | 1.1492 | 0.9101 |
| FVC | Girls | 4.75 | 111 | BCPE | 0.92332 | 1.1709 | 0.13166 | 4.49872 | 1.1709 | 0.9273 |
| FVC | Girls | 4.75 | 112 | BCPE | 0.92332 | 1.1928 | 0.13166 | 4.49872 | 1.1928 | 0.9446 |
| FVC | Girls | 4.75 | 113 | BCPE | 0.92332 | 1.2148 | 0.13166 | 4.49872 | 1.2148 | 0.9621 |
| FVC | Girls | 4.75 | 114 | BCPE | 0.92332 | 1.2371 | 0.13166 | 4.49872 | 1.2371 | 0.9798 |
| FVC | Girls | 4.75 | 115 | BCPE | 0.92332 | 1.2596 | 0.13166 | 4.49872 | 1.2596 | 0.9976 |
| FVC | Girls | 4.75 | 116 | BCPE | 0.92332 | 1.2823 | 0.13166 | 4.49872 | 1.2823 | 1.0156 |
| FVC | Girls | 4.75 | 117 | BCPE | 0.92332 | 1.3052 | 0.13166 | 4.49872 | 1.3052 | 1.0337 |
| FVC | Girls | 4.75 | 118 | BCPE | 0.92332 | 1.3284 | 0.13166 | 4.49872 | 1.3284 | 1.0520 |
| FVC | Girls | 4.75 | 119 | BCPE | 0.92332 | 1.3517 | 0.13166 | 4.49872 | 1.3517 | 1.0705 |
| FVC | Girls | 4.75 | 120 | BCPE | 0.92332 | 1.3752 | 0.13166 | 4.49872 | 1.3752 | 1.0891 |
| FVC | Girls | 4.75 | 121 | BCPE | 0.92332 | 1.3990 | 0.13166 | 4.49872 | 1.3990 | 1.1079 |
| FVC | Girls | 4.75 | 122 | BCPE | 0.92332 | 1.4230 | 0.13166 | 4.49872 | 1.4230 | 1.1269 |
| FVC | Girls | 4.75 | 123 | BCPE | 0.92332 | 1.4471 | 0.13166 | 4.49872 | 1.4471 | 1.1461 |
| FVC | Girls | 4.75 | 124 | BCPE | 0.92332 | 1.4715 | 0.13166 | 4.49872 | 1.4715 | 1.1654 |
| FVC | Girls | 5.00 | 88 | BCPE | 0.96380 | 0.7307 | 0.13101 | 4.78874 | 0.7307 | 0.5791 |
| FVC | Girls | 5.00 | 89 | BCPE | 0.96380 | 0.7479 | 0.13101 | 4.78874 | 0.7479 | 0.5928 |
| FVC | Girls | 5.00 | 90 | BCPE | 0.96380 | 0.7654 | 0.13101 | 4.78874 | 0.7654 | 0.6066 |
| FVC | Girls | 5.00 | 91 | BCPE | 0.96380 | 0.7830 | 0.13101 | 4.78874 | 0.7830 | 0.6206 |
| FVC | Girls | 5.00 | 92 | BCPE | 0.96380 | 0.8009 | 0.13101 | 4.78874 | 0.8009 | 0.6347 |
| FVC | Girls | 5.00 | 93 | BCPE | 0.96380 | 0.8190 | 0.13101 | 4.78874 | 0.8190 | 0.6490 |
| FVC | Girls | 5.00 | 94 | BCPE | 0.96380 | 0.8372 | 0.13101 | 4.78874 | 0.8372 | 0.6635 |
| FVC | Girls | 5.00 | 95 | BCPE | 0.96380 | 0.8557 | 0.13101 | 4.78874 | 0.8557 | 0.6782 |
| FVC | Girls | 5.00 | 96 | BCPE | 0.96380 | 0.8744 | 0.13101 | 4.78874 | 0.8744 | 0.6930 |
| FVC | Girls | 5.00 | 97 | BCPE | 0.96380 | 0.8933 | 0.13101 | 4.78874 | 0.8933 | 0.7079 |
| FVC | Girls | 5.00 | 98 | BCPE | 0.96380 | 0.9124 | 0.13101 | 4.78874 | 0.9124 | 0.7231 |
| FVC | Girls | 5.00 | 99 | BCPE | 0.96380 | 0.9317 | 0.13101 | 4.78874 | 0.9317 | 0.7384 |
| FVC | Girls | 5.00 | 100 | BCPE | 0.96380 | 0.9512 | 0.13101 | 4.78874 | 0.9512 | 0.7539 |
| FVC | Girls | 5.00 | 101 | BCPE | 0.96380 | 0.9710 | 0.13101 | 4.78874 | 0.9710 | 0.7695 |
| FVC | Girls | 5.00 | 102 | BCPE | 0.96380 | 0.9909 | 0.13101 | 4.78874 | 0.9909 | 0.7853 |
| FVC | Girls | 5.00 | 103 | BCPE | 0.96380 | 1.0111 | 0.13101 | 4.78874 | 1.0111 | 0.8013 |
| FVC | Girls | 5.00 | 104 | BCPE | 0.96380 | 1.0314 | 0.13101 | 4.78874 | 1.0314 | 0.8174 |
| FVC | Girls | 5.00 | 105 | BCPE | 0.96380 | 1.0520 | 0.13101 | 4.78874 | 1.0520 | 0.8337 |
| FVC | Girls | 5.00 | 106 | BCPE | 0.96380 | 1.0728 | 0.13101 | 4.78874 | 1.0728 | 0.8502 |
| FVC | Girls | 5.00 | 107 | BCPE | 0.96380 | 1.0938 | 0.13101 | 4.78874 | 1.0938 | 0.8668 |
| FVC | Girls | 5.00 | 108 | BCPE | 0.96380 | 1.1149 | 0.13101 | 4.78874 | 1.1149 | 0.8836 |
| FVC | Girls | 5.00 | 109 | BCPE | 0.96380 | 1.1364 | 0.13101 | 4.78874 | 1.1364 | 0.9006 |
| FVC | Girls | 5.00 | 110 | BCPE | 0.96380 | 1.1580 | 0.13101 | 4.78874 | 1.1580 | 0.9177 |
| FVC | Girls | 5.00 | 111 | BCPE | 0.96380 | 1.1798 | 0.13101 | 4.78874 | 1.1798 | 0.9350 |
| FVC | Girls | 5.00 | 112 | BCPE | 0.96380 | 1.2018 | 0.13101 | 4.78874 | 1.2018 | 0.9525 |
| FVC | Girls | 5.00 | 113 | BCPE | 0.96380 | 1.2241 | 0.13101 | 4.78874 | 1.2241 | 0.9701 |
| FVC | Girls | 5.00 | 114 | BCPE | 0.96380 | 1.2465 | 0.13101 | 4.78874 | 1.2465 | 0.9879 |
| FVC | Girls | 5.00 | 115 | BCPE | 0.96380 | 1.2692 | 0.13101 | 4.78874 | 1.2692 | 1.0058 |
| FVC | Girls | 5.00 | 116 | BCPE | 0.96380 | 1.2921 | 0.13101 | 4.78874 | 1.2921 | 1.0240 |
| FVC | Girls | 5.00 | 117 | BCPE | 0.96380 | 1.3152 | 0.13101 | 4.78874 | 1.3152 | 1.0423 |
| FVC | Girls | 5.00 | 118 | BCPE | 0.96380 | 1.3385 | 0.13101 | 4.78874 | 1.3385 | 1.0607 |
| FVC | Girls | 5.00 | 119 | BCPE | 0.96380 | 1.3620 | 0.13101 | 4.78874 | 1.3620 | 1.0794 |
| FVC | Girls | 5.00 | 120 | BCPE | 0.96380 | 1.3857 | 0.13101 | 4.78874 | 1.3857 | 1.0982 |
| FVC | Girls | 5.00 | 121 | BCPE | 0.96380 | 1.4096 | 0.13101 | 4.78874 | 1.4096 | 1.1171 |
| FVC | Girls | 5.00 | 122 | BCPE | 0.96380 | 1.4338 | 0.13101 | 4.78874 | 1.4338 | 1.1363 |
| FVC | Girls | 5.00 | 123 | BCPE | 0.96380 | 1.4581 | 0.13101 | 4.78874 | 1.4581 | 1.1556 |
| FVC | Girls | 5.00 | 124 | BCPE | 0.96380 | 1.4827 | 0.13101 | 4.78874 | 1.4827 | 1.1750 |
| FVC | Girls | 5.25 | 88 | BCPE | 1.00230 | 0.7360 | 0.13039 | 5.08193 | 0.7360 | 0.5836 |
| FVC | Girls | 5.25 | 89 | BCPE | 1.00230 | 0.7533 | 0.13039 | 5.08193 | 0.7533 | 0.5974 |
| FVC | Girls | 5.25 | 90 | BCPE | 1.00230 | 0.7709 | 0.13039 | 5.08193 | 0.7709 | 0.6113 |
| FVC | Girls | 5.25 | 91 | BCPE | 1.00230 | 0.7887 | 0.13039 | 5.08193 | 0.7887 | 0.6254 |
| FVC | Girls | 5.25 | 92 | BCPE | 1.00230 | 0.8067 | 0.13039 | 5.08193 | 0.8067 | 0.6397 |
| FVC | Girls | 5.25 | 93 | BCPE | 1.00230 | 0.8249 | 0.13039 | 5.08193 | 0.8249 | 0.6541 |
| FVC | Girls | 5.25 | 94 | BCPE | 1.00230 | 0.8433 | 0.13039 | 5.08193 | 0.8433 | 0.6687 |
| FVC | Girls | 5.25 | 95 | BCPE | 1.00230 | 0.8619 | 0.13039 | 5.08193 | 0.8619 | 0.6835 |
| FVC | Girls | 5.25 | 96 | BCPE | 1.00230 | 0.8807 | 0.13039 | 5.08193 | 0.8807 | 0.6984 |
| FVC | Girls | 5.25 | 97 | BCPE | 1.00230 | 0.8997 | 0.13039 | 5.08193 | 0.8997 | 0.7135 |
| FVC | Girls | 5.25 | 98 | BCPE | 1.00230 | 0.9190 | 0.13039 | 5.08193 | 0.9190 | 0.7288 |
| FVC | Girls | 5.25 | 99 | BCPE | 1.00230 | 0.9384 | 0.13039 | 5.08193 | 0.9384 | 0.7442 |
| FVC | Girls | 5.25 | 100 | BCPE | 1.00230 | 0.9581 | 0.13039 | 5.08193 | 0.9581 | 0.7598 |
| FVC | Girls | 5.25 | 101 | BCPE | 1.00230 | 0.9780 | 0.13039 | 5.08193 | 0.9780 | 0.7756 |
| FVC | Girls | 5.25 | 102 | BCPE | 1.00230 | 0.9981 | 0.13039 | 5.08193 | 0.9981 | 0.7915 |
| FVC | Girls | 5.25 | 103 | BCPE | 1.00230 | 1.0184 | 0.13039 | 5.08193 | 1.0184 | 0.8076 |
| FVC | Girls | 5.25 | 104 | BCPE | 1.00230 | 1.0389 | 0.13039 | 5.08193 | 1.0389 | 0.8238 |
| FVC | Girls | 5.25 | 105 | BCPE | 1.00230 | 1.0596 | 0.13039 | 5.08193 | 1.0596 | 0.8403 |
| FVC | Girls | 5.25 | 106 | BCPE | 1.00230 | 1.0805 | 0.13039 | 5.08193 | 1.0805 | 0.8569 |
| FVC | Girls | 5.25 | 107 | BCPE | 1.00230 | 1.1016 | 0.13039 | 5.08193 | 1.1016 | 0.8736 |
| FVC | Girls | 5.25 | 108 | BCPE | 1.00230 | 1.1230 | 0.13039 | 5.08193 | 1.1230 | 0.8905 |
| FVC | Girls | 5.25 | 109 | BCPE | 1.00230 | 1.1445 | 0.13039 | 5.08193 | 1.1445 | 0.9076 |
| FVC | Girls | 5.25 | 110 | BCPE | 1.00230 | 1.1663 | 0.13039 | 5.08193 | 1.1663 | 0.9249 |
| FVC | Girls | 5.25 | 111 | BCPE | 1.00230 | 1.1883 | 0.13039 | 5.08193 | 1.1883 | 0.9423 |
| FVC | Girls | 5.25 | 112 | BCPE | 1.00230 | 1.2105 | 0.13039 | 5.08193 | 1.2105 | 0.9599 |
| FVC | Girls | 5.25 | 113 | BCPE | 1.00230 | 1.2329 | 0.13039 | 5.08193 | 1.2329 | 0.9777 |
| FVC | Girls | 5.25 | 114 | BCPE | 1.00230 | 1.2555 | 0.13039 | 5.08193 | 1.2555 | 0.9956 |
| FVC | Girls | 5.25 | 115 | BCPE | 1.00230 | 1.2783 | 0.13039 | 5.08193 | 1.2783 | 1.0138 |
| FVC | Girls | 5.25 | 116 | BCPE | 1.00230 | 1.3014 | 0.13039 | 5.08193 | 1.3014 | 1.0320 |
| FVC | Girls | 5.25 | 117 | BCPE | 1.00230 | 1.3246 | 0.13039 | 5.08193 | 1.3246 | 1.0505 |
| FVC | Girls | 5.25 | 118 | BCPE | 1.00230 | 1.3481 | 0.13039 | 5.08193 | 1.3481 | 1.0691 |
| FVC | Girls | 5.25 | 119 | BCPE | 1.00230 | 1.3718 | 0.13039 | 5.08193 | 1.3718 | 1.0879 |
| FVC | Girls | 5.25 | 120 | BCPE | 1.00230 | 1.3957 | 0.13039 | 5.08193 | 1.3957 | 1.1068 |
| FVC | Girls | 5.25 | 121 | BCPE | 1.00230 | 1.4198 | 0.13039 | 5.08193 | 1.4198 | 1.1259 |
| FVC | Girls | 5.25 | 122 | BCPE | 1.00230 | 1.4441 | 0.13039 | 5.08193 | 1.4441 | 1.1452 |
| FVC | Girls | 5.25 | 123 | BCPE | 1.00230 | 1.4686 | 0.13039 | 5.08193 | 1.4686 | 1.1646 |
| FVC | Girls | 5.25 | 124 | BCPE | 1.00230 | 1.4934 | 0.13039 | 5.08193 | 1.4934 | 1.1843 |
| FVC | Girls | 5.50 | 88 | BCPE | 1.03901 | 0.7410 | 0.12980 | 5.37818 | 0.7410 | 0.5880 |
| FVC | Girls | 5.50 | 89 | BCPE | 1.03901 | 0.7585 | 0.12980 | 5.37818 | 0.7585 | 0.6019 |
| FVC | Girls | 5.50 | 90 | BCPE | 1.03901 | 0.7762 | 0.12980 | 5.37818 | 0.7762 | 0.6159 |
| FVC | Girls | 5.50 | 91 | BCPE | 1.03901 | 0.7941 | 0.12980 | 5.37818 | 0.7941 | 0.6301 |
| FVC | Girls | 5.50 | 92 | BCPE | 1.03901 | 0.8122 | 0.12980 | 5.37818 | 0.8122 | 0.6445 |
| FVC | Girls | 5.50 | 93 | BCPE | 1.03901 | 0.8305 | 0.12980 | 5.37818 | 0.8305 | 0.6590 |
| FVC | Girls | 5.50 | 94 | BCPE | 1.03901 | 0.8491 | 0.12980 | 5.37818 | 0.8491 | 0.6737 |
| FVC | Girls | 5.50 | 95 | BCPE | 1.03901 | 0.8678 | 0.12980 | 5.37818 | 0.8678 | 0.6886 |
| FVC | Girls | 5.50 | 96 | BCPE | 1.03901 | 0.8868 | 0.12980 | 5.37818 | 0.8868 | 0.7036 |
| FVC | Girls | 5.50 | 97 | BCPE | 1.03901 | 0.9059 | 0.12980 | 5.37818 | 0.9059 | 0.7188 |
| FVC | Girls | 5.50 | 98 | BCPE | 1.03901 | 0.9253 | 0.12980 | 5.37818 | 0.9253 | 0.7342 |
| FVC | Girls | 5.50 | 99 | BCPE | 1.03901 | 0.9449 | 0.12980 | 5.37818 | 0.9449 | 0.7498 |
| FVC | Girls | 5.50 | 100 | BCPE | 1.03901 | 0.9647 | 0.12980 | 5.37818 | 0.9647 | 0.7655 |
| FVC | Girls | 5.50 | 101 | BCPE | 1.03901 | 0.9847 | 0.12980 | 5.37818 | 0.9847 | 0.7813 |
| FVC | Girls | 5.50 | 102 | BCPE | 1.03901 | 1.0049 | 0.12980 | 5.37818 | 1.0049 | 0.7974 |
| FVC | Girls | 5.50 | 103 | BCPE | 1.03901 | 1.0253 | 0.12980 | 5.37818 | 1.0253 | 0.8136 |
| FVC | Girls | 5.50 | 104 | BCPE | 1.03901 | 1.0460 | 0.12980 | 5.37818 | 1.0460 | 0.8300 |
| FVC | Girls | 5.50 | 105 | BCPE | 1.03901 | 1.0668 | 0.12980 | 5.37818 | 1.0668 | 0.8465 |
| FVC | Girls | 5.50 | 106 | BCPE | 1.03901 | 1.0879 | 0.12980 | 5.37818 | 1.0879 | 0.8633 |
| FVC | Girls | 5.50 | 107 | BCPE | 1.03901 | 1.1092 | 0.12980 | 5.37818 | 1.1092 | 0.8801 |
| FVC | Girls | 5.50 | 108 | BCPE | 1.03901 | 1.1307 | 0.12980 | 5.37818 | 1.1307 | 0.8972 |
| FVC | Girls | 5.50 | 109 | BCPE | 1.03901 | 1.1524 | 0.12980 | 5.37818 | 1.1524 | 0.9144 |
| FVC | Girls | 5.50 | 110 | BCPE | 1.03901 | 1.1743 | 0.12980 | 5.37818 | 1.1743 | 0.9318 |
| FVC | Girls | 5.50 | 111 | BCPE | 1.03901 | 1.1965 | 0.12980 | 5.37818 | 1.1965 | 0.9494 |
| FVC | Girls | 5.50 | 112 | BCPE | 1.03901 | 1.2188 | 0.12980 | 5.37818 | 1.2188 | 0.9671 |
| FVC | Girls | 5.50 | 113 | BCPE | 1.03901 | 1.2414 | 0.12980 | 5.37818 | 1.2414 | 0.9850 |
| FVC | Girls | 5.50 | 114 | BCPE | 1.03901 | 1.2641 | 0.12980 | 5.37818 | 1.2641 | 1.0031 |
| FVC | Girls | 5.50 | 115 | BCPE | 1.03901 | 1.2871 | 0.12980 | 5.37818 | 1.2871 | 1.0213 |
| FVC | Girls | 5.50 | 116 | BCPE | 1.03901 | 1.3103 | 0.12980 | 5.37818 | 1.3103 | 1.0397 |
| FVC | Girls | 5.50 | 117 | BCPE | 1.03901 | 1.3337 | 0.12980 | 5.37818 | 1.3337 | 1.0583 |
| FVC | Girls | 5.50 | 118 | BCPE | 1.03901 | 1.3574 | 0.12980 | 5.37818 | 1.3574 | 1.0771 |
| FVC | Girls | 5.50 | 119 | BCPE | 1.03901 | 1.3812 | 0.12980 | 5.37818 | 1.3812 | 1.0960 |
| FVC | Girls | 5.50 | 120 | BCPE | 1.03901 | 1.4053 | 0.12980 | 5.37818 | 1.4053 | 1.1151 |
| FVC | Girls | 5.50 | 121 | BCPE | 1.03901 | 1.4295 | 0.12980 | 5.37818 | 1.4295 | 1.1343 |
| FVC | Girls | 5.50 | 122 | BCPE | 1.03901 | 1.4540 | 0.12980 | 5.37818 | 1.4540 | 1.1537 |
| FVC | Girls | 5.50 | 123 | BCPE | 1.03901 | 1.4787 | 0.12980 | 5.37818 | 1.4787 | 1.1733 |
| FVC | Girls | 5.50 | 124 | BCPE | 1.03901 | 1.5036 | 0.12980 | 5.37818 | 1.5036 | 1.1931 |
| FVC | Girls | 5.75 | 88 | BCPE | 1.07409 | 0.7459 | 0.12925 | 5.67739 | 0.7459 | 0.5922 |
| FVC | Girls | 5.75 | 89 | BCPE | 1.07409 | 0.7635 | 0.12925 | 5.67739 | 0.7635 | 0.6061 |
| FVC | Girls | 5.75 | 90 | BCPE | 1.07409 | 0.7813 | 0.12925 | 5.67739 | 0.7813 | 0.6203 |
| FVC | Girls | 5.75 | 91 | BCPE | 1.07409 | 0.7993 | 0.12925 | 5.67739 | 0.7993 | 0.6346 |
| FVC | Girls | 5.75 | 92 | BCPE | 1.07409 | 0.8175 | 0.12925 | 5.67739 | 0.8175 | 0.6491 |
| FVC | Girls | 5.75 | 93 | BCPE | 1.07409 | 0.8360 | 0.12925 | 5.67739 | 0.8360 | 0.6637 |
| FVC | Girls | 5.75 | 94 | BCPE | 1.07409 | 0.8546 | 0.12925 | 5.67739 | 0.8546 | 0.6785 |
| FVC | Girls | 5.75 | 95 | BCPE | 1.07409 | 0.8735 | 0.12925 | 5.67739 | 0.8735 | 0.6935 |
| FVC | Girls | 5.75 | 96 | BCPE | 1.07409 | 0.8926 | 0.12925 | 5.67739 | 0.8926 | 0.7086 |
| FVC | Girls | 5.75 | 97 | BCPE | 1.07409 | 0.9119 | 0.12925 | 5.67739 | 0.9119 | 0.7239 |
| FVC | Girls | 5.75 | 98 | BCPE | 1.07409 | 0.9314 | 0.12925 | 5.67739 | 0.9314 | 0.7394 |
| FVC | Girls | 5.75 | 99 | BCPE | 1.07409 | 0.9511 | 0.12925 | 5.67739 | 0.9511 | 0.7551 |
| FVC | Girls | 5.75 | 100 | BCPE | 1.07409 | 0.9710 | 0.12925 | 5.67739 | 0.9710 | 0.7709 |
| FVC | Girls | 5.75 | 101 | BCPE | 1.07409 | 0.9911 | 0.12925 | 5.67739 | 0.9911 | 0.7869 |
| FVC | Girls | 5.75 | 102 | BCPE | 1.07409 | 1.0115 | 0.12925 | 5.67739 | 1.0115 | 0.8031 |
| FVC | Girls | 5.75 | 103 | BCPE | 1.07409 | 1.0321 | 0.12925 | 5.67739 | 1.0321 | 0.8194 |
| FVC | Girls | 5.75 | 104 | BCPE | 1.07409 | 1.0528 | 0.12925 | 5.67739 | 1.0528 | 0.8359 |
| FVC | Girls | 5.75 | 105 | BCPE | 1.07409 | 1.0738 | 0.12925 | 5.67739 | 1.0738 | 0.8526 |
| FVC | Girls | 5.75 | 106 | BCPE | 1.07409 | 1.0950 | 0.12925 | 5.67739 | 1.0950 | 0.8694 |
| FVC | Girls | 5.75 | 107 | BCPE | 1.07409 | 1.1165 | 0.12925 | 5.67739 | 1.1165 | 0.8864 |
| FVC | Girls | 5.75 | 108 | BCPE | 1.07409 | 1.1381 | 0.12925 | 5.67739 | 1.1381 | 0.9036 |
| FVC | Girls | 5.75 | 109 | BCPE | 1.07409 | 1.1600 | 0.12925 | 5.67739 | 1.1600 | 0.9209 |
| FVC | Girls | 5.75 | 110 | BCPE | 1.07409 | 1.1820 | 0.12925 | 5.67739 | 1.1820 | 0.9384 |
| FVC | Girls | 5.75 | 111 | BCPE | 1.07409 | 1.2043 | 0.12925 | 5.67739 | 1.2043 | 0.9561 |
| FVC | Girls | 5.75 | 112 | BCPE | 1.07409 | 1.2268 | 0.12925 | 5.67739 | 1.2268 | 0.9740 |
| FVC | Girls | 5.75 | 113 | BCPE | 1.07409 | 1.2495 | 0.12925 | 5.67739 | 1.2495 | 0.9920 |
| FVC | Girls | 5.75 | 114 | BCPE | 1.07409 | 1.2724 | 0.12925 | 5.67739 | 1.2724 | 1.0102 |
| FVC | Girls | 5.75 | 115 | BCPE | 1.07409 | 1.2956 | 0.12925 | 5.67739 | 1.2956 | 1.0286 |
| FVC | Girls | 5.75 | 116 | BCPE | 1.07409 | 1.3189 | 0.12925 | 5.67739 | 1.3189 | 1.0471 |
| FVC | Girls | 5.75 | 117 | BCPE | 1.07409 | 1.3425 | 0.12925 | 5.67739 | 1.3425 | 1.0658 |
| FVC | Girls | 5.75 | 118 | BCPE | 1.07409 | 1.3663 | 0.12925 | 5.67739 | 1.3663 | 1.0847 |
| FVC | Girls | 5.75 | 119 | BCPE | 1.07409 | 1.3903 | 0.12925 | 5.67739 | 1.3903 | 1.1038 |
| FVC | Girls | 5.75 | 120 | BCPE | 1.07409 | 1.4145 | 0.12925 | 5.67739 | 1.4145 | 1.1230 |
| FVC | Girls | 5.75 | 121 | BCPE | 1.07409 | 1.4389 | 0.12925 | 5.67739 | 1.4389 | 1.1424 |
| FVC | Girls | 5.75 | 122 | BCPE | 1.07409 | 1.4635 | 0.12925 | 5.67739 | 1.4635 | 1.1619 |
| FVC | Girls | 5.75 | 123 | BCPE | 1.07409 | 1.4884 | 0.12925 | 5.67739 | 1.4884 | 1.1817 |
| FVC | Girls | 5.75 | 124 | BCPE | 1.07409 | 1.5135 | 0.12925 | 5.67739 | 1.5135 | 1.2016 |
| FVC | Girls | 6.00 | 88 | BCPE | 1.10767 | 0.7506 | 0.12871 | 5.97944 | 0.7506 | 0.5962 |
| FVC | Girls | 6.00 | 89 | BCPE | 1.10767 | 0.7683 | 0.12871 | 5.97944 | 0.7683 | 0.6103 |
| FVC | Girls | 6.00 | 90 | BCPE | 1.10767 | 0.7862 | 0.12871 | 5.97944 | 0.7862 | 0.6245 |
| FVC | Girls | 6.00 | 91 | BCPE | 1.10767 | 0.8043 | 0.12871 | 5.97944 | 0.8043 | 0.6389 |
| FVC | Girls | 6.00 | 92 | BCPE | 1.10767 | 0.8227 | 0.12871 | 5.97944 | 0.8227 | 0.6535 |
| FVC | Girls | 6.00 | 93 | BCPE | 1.10767 | 0.8412 | 0.12871 | 5.97944 | 0.8412 | 0.6682 |
| FVC | Girls | 6.00 | 94 | BCPE | 1.10767 | 0.8600 | 0.12871 | 5.97944 | 0.8600 | 0.6831 |
| FVC | Girls | 6.00 | 95 | BCPE | 1.10767 | 0.8790 | 0.12871 | 5.97944 | 0.8790 | 0.6982 |
| FVC | Girls | 6.00 | 96 | BCPE | 1.10767 | 0.8982 | 0.12871 | 5.97944 | 0.8982 | 0.7134 |
| FVC | Girls | 6.00 | 97 | BCPE | 1.10767 | 0.9176 | 0.12871 | 5.97944 | 0.9176 | 0.7289 |
| FVC | Girls | 6.00 | 98 | BCPE | 1.10767 | 0.9372 | 0.12871 | 5.97944 | 0.9372 | 0.7444 |
| FVC | Girls | 6.00 | 99 | BCPE | 1.10767 | 0.9570 | 0.12871 | 5.97944 | 0.9570 | 0.7602 |
| FVC | Girls | 6.00 | 100 | BCPE | 1.10767 | 0.9771 | 0.12871 | 5.97944 | 0.9771 | 0.7761 |
| FVC | Girls | 6.00 | 101 | BCPE | 1.10767 | 0.9974 | 0.12871 | 5.97944 | 0.9974 | 0.7922 |
| FVC | Girls | 6.00 | 102 | BCPE | 1.10767 | 1.0178 | 0.12871 | 5.97944 | 1.0178 | 0.8085 |
| FVC | Girls | 6.00 | 103 | BCPE | 1.10767 | 1.0385 | 0.12871 | 5.97944 | 1.0385 | 0.8249 |
| FVC | Girls | 6.00 | 104 | BCPE | 1.10767 | 1.0594 | 0.12871 | 5.97944 | 1.0594 | 0.8416 |
| FVC | Girls | 6.00 | 105 | BCPE | 1.10767 | 1.0806 | 0.12871 | 5.97944 | 1.0806 | 0.8583 |
| FVC | Girls | 6.00 | 106 | BCPE | 1.10767 | 1.1019 | 0.12871 | 5.97944 | 1.1019 | 0.8753 |
| FVC | Girls | 6.00 | 107 | BCPE | 1.10767 | 1.1235 | 0.12871 | 5.97944 | 1.1235 | 0.8924 |
| FVC | Girls | 6.00 | 108 | BCPE | 1.10767 | 1.1452 | 0.12871 | 5.97944 | 1.1452 | 0.9097 |
| FVC | Girls | 6.00 | 109 | BCPE | 1.10767 | 1.1672 | 0.12871 | 5.97944 | 1.1672 | 0.9272 |
| FVC | Girls | 6.00 | 110 | BCPE | 1.10767 | 1.1894 | 0.12871 | 5.97944 | 1.1894 | 0.9448 |
| FVC | Girls | 6.00 | 111 | BCPE | 1.10767 | 1.2119 | 0.12871 | 5.97944 | 1.2119 | 0.9626 |
| FVC | Girls | 6.00 | 112 | BCPE | 1.10767 | 1.2345 | 0.12871 | 5.97944 | 1.2345 | 0.9806 |
| FVC | Girls | 6.00 | 113 | BCPE | 1.10767 | 1.2573 | 0.12871 | 5.97944 | 1.2573 | 0.9987 |
| FVC | Girls | 6.00 | 114 | BCPE | 1.10767 | 1.2804 | 0.12871 | 5.97944 | 1.2804 | 1.0171 |
| FVC | Girls | 6.00 | 115 | BCPE | 1.10767 | 1.3037 | 0.12871 | 5.97944 | 1.3037 | 1.0356 |
| FVC | Girls | 6.00 | 116 | BCPE | 1.10767 | 1.3272 | 0.12871 | 5.97944 | 1.3272 | 1.0542 |
| FVC | Girls | 6.00 | 117 | BCPE | 1.10767 | 1.3509 | 0.12871 | 5.97944 | 1.3509 | 1.0731 |
| FVC | Girls | 6.00 | 118 | BCPE | 1.10767 | 1.3748 | 0.12871 | 5.97944 | 1.3748 | 1.0921 |
| FVC | Girls | 6.00 | 119 | BCPE | 1.10767 | 1.3990 | 0.12871 | 5.97944 | 1.3990 | 1.1113 |
| FVC | Girls | 6.00 | 120 | BCPE | 1.10767 | 1.4233 | 0.12871 | 5.97944 | 1.4233 | 1.1306 |
| FVC | Girls | 6.00 | 121 | BCPE | 1.10767 | 1.4479 | 0.12871 | 5.97944 | 1.4479 | 1.1501 |
| FVC | Girls | 6.00 | 122 | BCPE | 1.10767 | 1.4727 | 0.12871 | 5.97944 | 1.4727 | 1.1698 |
| FVC | Girls | 6.00 | 123 | BCPE | 1.10767 | 1.4977 | 0.12871 | 5.97944 | 1.4977 | 1.1897 |
| FVC | Girls | 6.00 | 124 | BCPE | 1.10767 | 1.5230 | 0.12871 | 5.97944 | 1.5230 | 1.2097 |

**Table A3. Sex-specific LMS parameters, predicted values, and lower limits of normal (LLN) for FEV_1_/FVC across the Phase 1 age and height range.**

| **Outcome** | **Sex** | **Age** | **Height (cm)** | **Family** | **L** | **M** | **S** | **K** | **Pred** | **LLN** |
| --- | --- | --- | --- | --- | --- | --- | --- | --- | --- | --- |
| FEV1/FVC | Boys | 3.00 | 79 | BCCG | 4.26297 | 0.9870 | 0.05032 |  | 0.9870 | 0.8912 |
| FEV1/FVC | Boys | 3.00 | 80 | BCCG | 4.26297 | 0.9837 | 0.05032 |  | 0.9837 | 0.8883 |
| FEV1/FVC | Boys | 3.00 | 81 | BCCG | 4.26297 | 0.9806 | 0.05032 |  | 0.9806 | 0.8854 |
| FEV1/FVC | Boys | 3.00 | 82 | BCCG | 4.26297 | 0.9775 | 0.05032 |  | 0.9775 | 0.8826 |
| FEV1/FVC | Boys | 3.00 | 83 | BCCG | 4.26297 | 0.9744 | 0.05032 |  | 0.9744 | 0.8799 |
| FEV1/FVC | Boys | 3.00 | 84 | BCCG | 4.26297 | 0.9714 | 0.05032 |  | 0.9714 | 0.8771 |
| FEV1/FVC | Boys | 3.00 | 85 | BCCG | 4.26297 | 0.9684 | 0.05032 |  | 0.9684 | 0.8744 |
| FEV1/FVC | Boys | 3.00 | 86 | BCCG | 4.26297 | 0.9655 | 0.05032 |  | 0.9655 | 0.8718 |
| FEV1/FVC | Boys | 3.00 | 87 | BCCG | 4.26297 | 0.9626 | 0.05032 |  | 0.9626 | 0.8692 |
| FEV1/FVC | Boys | 3.00 | 88 | BCCG | 4.26297 | 0.9597 | 0.05032 |  | 0.9597 | 0.8666 |
| FEV1/FVC | Boys | 3.00 | 89 | BCCG | 4.26297 | 0.9569 | 0.05032 |  | 0.9569 | 0.8641 |
| FEV1/FVC | Boys | 3.00 | 90 | BCCG | 4.26297 | 0.9542 | 0.05032 |  | 0.9542 | 0.8616 |
| FEV1/FVC | Boys | 3.00 | 91 | BCCG | 4.26297 | 0.9514 | 0.05032 |  | 0.9514 | 0.8591 |
| FEV1/FVC | Boys | 3.00 | 92 | BCCG | 4.26297 | 0.9488 | 0.05032 |  | 0.9488 | 0.8567 |
| FEV1/FVC | Boys | 3.00 | 93 | BCCG | 4.26297 | 0.9461 | 0.05032 |  | 0.9461 | 0.8543 |
| FEV1/FVC | Boys | 3.00 | 94 | BCCG | 4.26297 | 0.9435 | 0.05032 |  | 0.9435 | 0.8519 |
| FEV1/FVC | Boys | 3.00 | 95 | BCCG | 4.26297 | 0.9409 | 0.05032 |  | 0.9409 | 0.8496 |
| FEV1/FVC | Boys | 3.00 | 96 | BCCG | 4.26297 | 0.9383 | 0.05032 |  | 0.9383 | 0.8473 |
| FEV1/FVC | Boys | 3.00 | 97 | BCCG | 4.26297 | 0.9358 | 0.05032 |  | 0.9358 | 0.8450 |
| FEV1/FVC | Boys | 3.00 | 98 | BCCG | 4.26297 | 0.9333 | 0.05032 |  | 0.9333 | 0.8428 |
| FEV1/FVC | Boys | 3.00 | 99 | BCCG | 4.26297 | 0.9309 | 0.05032 |  | 0.9309 | 0.8406 |
| FEV1/FVC | Boys | 3.00 | 100 | BCCG | 4.26297 | 0.9285 | 0.05032 |  | 0.9285 | 0.8384 |
| FEV1/FVC | Boys | 3.00 | 101 | BCCG | 4.26297 | 0.9261 | 0.05032 |  | 0.9261 | 0.8362 |
| FEV1/FVC | Boys | 3.00 | 102 | BCCG | 4.26297 | 0.9237 | 0.05032 |  | 0.9237 | 0.8341 |
| FEV1/FVC | Boys | 3.00 | 103 | BCCG | 4.26297 | 0.9214 | 0.05032 |  | 0.9214 | 0.8320 |
| FEV1/FVC | Boys | 3.00 | 104 | BCCG | 4.26297 | 0.9191 | 0.05032 |  | 0.9191 | 0.8299 |
| FEV1/FVC | Boys | 3.00 | 105 | BCCG | 4.26297 | 0.9168 | 0.05032 |  | 0.9168 | 0.8279 |
| FEV1/FVC | Boys | 3.00 | 106 | BCCG | 4.26297 | 0.9146 | 0.05032 |  | 0.9146 | 0.8258 |
| FEV1/FVC | Boys | 3.00 | 107 | BCCG | 4.26297 | 0.9123 | 0.05032 |  | 0.9123 | 0.8238 |
| FEV1/FVC | Boys | 3.00 | 108 | BCCG | 4.26297 | 0.9101 | 0.05032 |  | 0.9101 | 0.8218 |
| FEV1/FVC | Boys | 3.00 | 109 | BCCG | 4.26297 | 0.9080 | 0.05032 |  | 0.9080 | 0.8199 |
| FEV1/FVC | Boys | 3.00 | 110 | BCCG | 4.26297 | 0.9058 | 0.05032 |  | 0.9058 | 0.8179 |
| FEV1/FVC | Boys | 3.00 | 111 | BCCG | 4.26297 | 0.9037 | 0.05032 |  | 0.9037 | 0.8160 |
| FEV1/FVC | Boys | 3.00 | 112 | BCCG | 4.26297 | 0.9016 | 0.05032 |  | 0.9016 | 0.8141 |
| FEV1/FVC | Boys | 3.00 | 113 | BCCG | 4.26297 | 0.8995 | 0.05032 |  | 0.8995 | 0.8122 |
| FEV1/FVC | Boys | 3.00 | 114 | BCCG | 4.26297 | 0.8975 | 0.05032 |  | 0.8975 | 0.8104 |
| FEV1/FVC | Boys | 3.00 | 115 | BCCG | 4.26297 | 0.8955 | 0.05032 |  | 0.8955 | 0.8086 |
| FEV1/FVC | Boys | 3.00 | 116 | BCCG | 4.26297 | 0.8934 | 0.05032 |  | 0.8934 | 0.8068 |
| FEV1/FVC | Boys | 3.00 | 117 | BCCG | 4.26297 | 0.8915 | 0.05032 |  | 0.8915 | 0.8050 |
| FEV1/FVC | Boys | 3.00 | 118 | BCCG | 4.26297 | 0.8895 | 0.05032 |  | 0.8895 | 0.8032 |
| FEV1/FVC | Boys | 3.00 | 119 | BCCG | 4.26297 | 0.8876 | 0.05032 |  | 0.8876 | 0.8014 |
| FEV1/FVC | Boys | 3.00 | 120 | BCCG | 4.26297 | 0.8856 | 0.05032 |  | 0.8856 | 0.7997 |
| FEV1/FVC | Boys | 3.00 | 121 | BCCG | 4.26297 | 0.8837 | 0.05032 |  | 0.8837 | 0.7980 |
| FEV1/FVC | Boys | 3.00 | 122 | BCCG | 4.26297 | 0.8818 | 0.05032 |  | 0.8818 | 0.7963 |
| FEV1/FVC | Boys | 3.00 | 123 | BCCG | 4.26297 | 0.8800 | 0.05032 |  | 0.8800 | 0.7946 |
| FEV1/FVC | Boys | 3.00 | 124 | BCCG | 4.26297 | 0.8781 | 0.05032 |  | 0.8781 | 0.7929 |
| FEV1/FVC | Boys | 3.00 | 125 | BCCG | 4.26297 | 0.8763 | 0.05032 |  | 0.8763 | 0.7913 |
| FEV1/FVC | Boys | 3.00 | 126 | BCCG | 4.26297 | 0.8745 | 0.05032 |  | 0.8745 | 0.7897 |
| FEV1/FVC | Boys | 3.00 | 127 | BCCG | 4.26297 | 0.8727 | 0.05032 |  | 0.8727 | 0.7880 |
| FEV1/FVC | Boys | 3.00 | 128 | BCCG | 4.26297 | 0.8709 | 0.05032 |  | 0.8709 | 0.7864 |
| FEV1/FVC | Boys | 3.00 | 129 | BCCG | 4.26297 | 0.8692 | 0.05032 |  | 0.8692 | 0.7849 |
| FEV1/FVC | Boys | 3.00 | 130 | BCCG | 4.26297 | 0.8675 | 0.05032 |  | 0.8675 | 0.7833 |
| FEV1/FVC | Boys | 3.00 | 131 | BCCG | 4.26297 | 0.8657 | 0.05032 |  | 0.8657 | 0.7817 |
| FEV1/FVC | Boys | 3.00 | 132 | BCCG | 4.26297 | 0.8640 | 0.05032 |  | 0.8640 | 0.7802 |
| FEV1/FVC | Boys | 3.25 | 79 | BCCG | 4.26297 | 0.9918 | 0.05032 |  | 0.9918 | 0.8955 |
| FEV1/FVC | Boys | 3.25 | 80 | BCCG | 4.26297 | 0.9885 | 0.05032 |  | 0.9885 | 0.8926 |
| FEV1/FVC | Boys | 3.25 | 81 | BCCG | 4.26297 | 0.9853 | 0.05032 |  | 0.9853 | 0.8897 |
| FEV1/FVC | Boys | 3.25 | 82 | BCCG | 4.26297 | 0.9822 | 0.05032 |  | 0.9822 | 0.8869 |
| FEV1/FVC | Boys | 3.25 | 83 | BCCG | 4.26297 | 0.9791 | 0.05032 |  | 0.9791 | 0.8841 |
| FEV1/FVC | Boys | 3.25 | 84 | BCCG | 4.26297 | 0.9761 | 0.05032 |  | 0.9761 | 0.8814 |
| FEV1/FVC | Boys | 3.25 | 85 | BCCG | 4.26297 | 0.9731 | 0.05032 |  | 0.9731 | 0.8787 |
| FEV1/FVC | Boys | 3.25 | 86 | BCCG | 4.26297 | 0.9702 | 0.05032 |  | 0.9702 | 0.8760 |
| FEV1/FVC | Boys | 3.25 | 87 | BCCG | 4.26297 | 0.9673 | 0.05032 |  | 0.9673 | 0.8734 |
| FEV1/FVC | Boys | 3.25 | 88 | BCCG | 4.26297 | 0.9644 | 0.05032 |  | 0.9644 | 0.8708 |
| FEV1/FVC | Boys | 3.25 | 89 | BCCG | 4.26297 | 0.9616 | 0.05032 |  | 0.9616 | 0.8683 |
| FEV1/FVC | Boys | 3.25 | 90 | BCCG | 4.26297 | 0.9588 | 0.05032 |  | 0.9588 | 0.8658 |
| FEV1/FVC | Boys | 3.25 | 91 | BCCG | 4.26297 | 0.9561 | 0.05032 |  | 0.9561 | 0.8633 |
| FEV1/FVC | Boys | 3.25 | 92 | BCCG | 4.26297 | 0.9534 | 0.05032 |  | 0.9534 | 0.8609 |
| FEV1/FVC | Boys | 3.25 | 93 | BCCG | 4.26297 | 0.9507 | 0.05032 |  | 0.9507 | 0.8584 |
| FEV1/FVC | Boys | 3.25 | 94 | BCCG | 4.26297 | 0.9481 | 0.05032 |  | 0.9481 | 0.8561 |
| FEV1/FVC | Boys | 3.25 | 95 | BCCG | 4.26297 | 0.9455 | 0.05032 |  | 0.9455 | 0.8537 |
| FEV1/FVC | Boys | 3.25 | 96 | BCCG | 4.26297 | 0.9429 | 0.05032 |  | 0.9429 | 0.8514 |
| FEV1/FVC | Boys | 3.25 | 97 | BCCG | 4.26297 | 0.9404 | 0.05032 |  | 0.9404 | 0.8491 |
| FEV1/FVC | Boys | 3.25 | 98 | BCCG | 4.26297 | 0.9379 | 0.05032 |  | 0.9379 | 0.8469 |
| FEV1/FVC | Boys | 3.25 | 99 | BCCG | 4.26297 | 0.9354 | 0.05032 |  | 0.9354 | 0.8447 |
| FEV1/FVC | Boys | 3.25 | 100 | BCCG | 4.26297 | 0.9330 | 0.05032 |  | 0.9330 | 0.8425 |
| FEV1/FVC | Boys | 3.25 | 101 | BCCG | 4.26297 | 0.9306 | 0.05032 |  | 0.9306 | 0.8403 |
| FEV1/FVC | Boys | 3.25 | 102 | BCCG | 4.26297 | 0.9282 | 0.05032 |  | 0.9282 | 0.8381 |
| FEV1/FVC | Boys | 3.25 | 103 | BCCG | 4.26297 | 0.9259 | 0.05032 |  | 0.9259 | 0.8360 |
| FEV1/FVC | Boys | 3.25 | 104 | BCCG | 4.26297 | 0.9236 | 0.05032 |  | 0.9236 | 0.8339 |
| FEV1/FVC | Boys | 3.25 | 105 | BCCG | 4.26297 | 0.9213 | 0.05032 |  | 0.9213 | 0.8319 |
| FEV1/FVC | Boys | 3.25 | 106 | BCCG | 4.26297 | 0.9190 | 0.05032 |  | 0.9190 | 0.8298 |
| FEV1/FVC | Boys | 3.25 | 107 | BCCG | 4.26297 | 0.9168 | 0.05032 |  | 0.9168 | 0.8278 |
| FEV1/FVC | Boys | 3.25 | 108 | BCCG | 4.26297 | 0.9146 | 0.05032 |  | 0.9146 | 0.8258 |
| FEV1/FVC | Boys | 3.25 | 109 | BCCG | 4.26297 | 0.9124 | 0.05032 |  | 0.9124 | 0.8239 |
| FEV1/FVC | Boys | 3.25 | 110 | BCCG | 4.26297 | 0.9102 | 0.05032 |  | 0.9102 | 0.8219 |
| FEV1/FVC | Boys | 3.25 | 111 | BCCG | 4.26297 | 0.9081 | 0.05032 |  | 0.9081 | 0.8200 |
| FEV1/FVC | Boys | 3.25 | 112 | BCCG | 4.26297 | 0.9060 | 0.05032 |  | 0.9060 | 0.8181 |
| FEV1/FVC | Boys | 3.25 | 113 | BCCG | 4.26297 | 0.9039 | 0.05032 |  | 0.9039 | 0.8162 |
| FEV1/FVC | Boys | 3.25 | 114 | BCCG | 4.26297 | 0.9018 | 0.05032 |  | 0.9018 | 0.8143 |
| FEV1/FVC | Boys | 3.25 | 115 | BCCG | 4.26297 | 0.8998 | 0.05032 |  | 0.8998 | 0.8125 |
| FEV1/FVC | Boys | 3.25 | 116 | BCCG | 4.26297 | 0.8978 | 0.05032 |  | 0.8978 | 0.8107 |
| FEV1/FVC | Boys | 3.25 | 117 | BCCG | 4.26297 | 0.8958 | 0.05032 |  | 0.8958 | 0.8089 |
| FEV1/FVC | Boys | 3.25 | 118 | BCCG | 4.26297 | 0.8938 | 0.05032 |  | 0.8938 | 0.8071 |
| FEV1/FVC | Boys | 3.25 | 119 | BCCG | 4.26297 | 0.8919 | 0.05032 |  | 0.8919 | 0.8053 |
| FEV1/FVC | Boys | 3.25 | 120 | BCCG | 4.26297 | 0.8899 | 0.05032 |  | 0.8899 | 0.8036 |
| FEV1/FVC | Boys | 3.25 | 121 | BCCG | 4.26297 | 0.8880 | 0.05032 |  | 0.8880 | 0.8019 |
| FEV1/FVC | Boys | 3.25 | 122 | BCCG | 4.26297 | 0.8861 | 0.05032 |  | 0.8861 | 0.8001 |
| FEV1/FVC | Boys | 3.25 | 123 | BCCG | 4.26297 | 0.8843 | 0.05032 |  | 0.8843 | 0.7985 |
| FEV1/FVC | Boys | 3.25 | 124 | BCCG | 4.26297 | 0.8824 | 0.05032 |  | 0.8824 | 0.7968 |
| FEV1/FVC | Boys | 3.25 | 125 | BCCG | 4.26297 | 0.8806 | 0.05032 |  | 0.8806 | 0.7951 |
| FEV1/FVC | Boys | 3.25 | 126 | BCCG | 4.26297 | 0.8788 | 0.05032 |  | 0.8788 | 0.7935 |
| FEV1/FVC | Boys | 3.25 | 127 | BCCG | 4.26297 | 0.8770 | 0.05032 |  | 0.8770 | 0.7919 |
| FEV1/FVC | Boys | 3.25 | 128 | BCCG | 4.26297 | 0.8752 | 0.05032 |  | 0.8752 | 0.7903 |
| FEV1/FVC | Boys | 3.25 | 129 | BCCG | 4.26297 | 0.8734 | 0.05032 |  | 0.8734 | 0.7887 |
| FEV1/FVC | Boys | 3.25 | 130 | BCCG | 4.26297 | 0.8717 | 0.05032 |  | 0.8717 | 0.7871 |
| FEV1/FVC | Boys | 3.25 | 131 | BCCG | 4.26297 | 0.8699 | 0.05032 |  | 0.8699 | 0.7855 |
| FEV1/FVC | Boys | 3.25 | 132 | BCCG | 4.26297 | 0.8682 | 0.05032 |  | 0.8682 | 0.7840 |
| FEV1/FVC | Boys | 3.50 | 79 | BCCG | 4.26297 | 0.9962 | 0.05032 |  | 0.9962 | 0.8995 |
| FEV1/FVC | Boys | 3.50 | 80 | BCCG | 4.26297 | 0.9930 | 0.05032 |  | 0.9930 | 0.8966 |
| FEV1/FVC | Boys | 3.50 | 81 | BCCG | 4.26297 | 0.9898 | 0.05032 |  | 0.9898 | 0.8937 |
| FEV1/FVC | Boys | 3.50 | 82 | BCCG | 4.26297 | 0.9866 | 0.05032 |  | 0.9866 | 0.8909 |
| FEV1/FVC | Boys | 3.50 | 83 | BCCG | 4.26297 | 0.9835 | 0.05032 |  | 0.9835 | 0.8881 |
| FEV1/FVC | Boys | 3.50 | 84 | BCCG | 4.26297 | 0.9805 | 0.05032 |  | 0.9805 | 0.8854 |
| FEV1/FVC | Boys | 3.50 | 85 | BCCG | 4.26297 | 0.9775 | 0.05032 |  | 0.9775 | 0.8826 |
| FEV1/FVC | Boys | 3.50 | 86 | BCCG | 4.26297 | 0.9745 | 0.05032 |  | 0.9745 | 0.8800 |
| FEV1/FVC | Boys | 3.50 | 87 | BCCG | 4.26297 | 0.9716 | 0.05032 |  | 0.9716 | 0.8773 |
| FEV1/FVC | Boys | 3.50 | 88 | BCCG | 4.26297 | 0.9687 | 0.05032 |  | 0.9687 | 0.8747 |
| FEV1/FVC | Boys | 3.50 | 89 | BCCG | 4.26297 | 0.9659 | 0.05032 |  | 0.9659 | 0.8722 |
| FEV1/FVC | Boys | 3.50 | 90 | BCCG | 4.26297 | 0.9631 | 0.05032 |  | 0.9631 | 0.8697 |
| FEV1/FVC | Boys | 3.50 | 91 | BCCG | 4.26297 | 0.9604 | 0.05032 |  | 0.9604 | 0.8672 |
| FEV1/FVC | Boys | 3.50 | 92 | BCCG | 4.26297 | 0.9577 | 0.05032 |  | 0.9577 | 0.8647 |
| FEV1/FVC | Boys | 3.50 | 93 | BCCG | 4.26297 | 0.9550 | 0.05032 |  | 0.9550 | 0.8623 |
| FEV1/FVC | Boys | 3.50 | 94 | BCCG | 4.26297 | 0.9523 | 0.05032 |  | 0.9523 | 0.8599 |
| FEV1/FVC | Boys | 3.50 | 95 | BCCG | 4.26297 | 0.9497 | 0.05032 |  | 0.9497 | 0.8576 |
| FEV1/FVC | Boys | 3.50 | 96 | BCCG | 4.26297 | 0.9471 | 0.05032 |  | 0.9471 | 0.8552 |
| FEV1/FVC | Boys | 3.50 | 97 | BCCG | 4.26297 | 0.9446 | 0.05032 |  | 0.9446 | 0.8529 |
| FEV1/FVC | Boys | 3.50 | 98 | BCCG | 4.26297 | 0.9421 | 0.05032 |  | 0.9421 | 0.8507 |
| FEV1/FVC | Boys | 3.50 | 99 | BCCG | 4.26297 | 0.9396 | 0.05032 |  | 0.9396 | 0.8485 |
| FEV1/FVC | Boys | 3.50 | 100 | BCCG | 4.26297 | 0.9372 | 0.05032 |  | 0.9372 | 0.8462 |
| FEV1/FVC | Boys | 3.50 | 101 | BCCG | 4.26297 | 0.9348 | 0.05032 |  | 0.9348 | 0.8441 |
| FEV1/FVC | Boys | 3.50 | 102 | BCCG | 4.26297 | 0.9324 | 0.05032 |  | 0.9324 | 0.8419 |
| FEV1/FVC | Boys | 3.50 | 103 | BCCG | 4.26297 | 0.9300 | 0.05032 |  | 0.9300 | 0.8398 |
| FEV1/FVC | Boys | 3.50 | 104 | BCCG | 4.26297 | 0.9277 | 0.05032 |  | 0.9277 | 0.8377 |
| FEV1/FVC | Boys | 3.50 | 105 | BCCG | 4.26297 | 0.9254 | 0.05032 |  | 0.9254 | 0.8356 |
| FEV1/FVC | Boys | 3.50 | 106 | BCCG | 4.26297 | 0.9231 | 0.05032 |  | 0.9231 | 0.8336 |
| FEV1/FVC | Boys | 3.50 | 107 | BCCG | 4.26297 | 0.9209 | 0.05032 |  | 0.9209 | 0.8315 |
| FEV1/FVC | Boys | 3.50 | 108 | BCCG | 4.26297 | 0.9187 | 0.05032 |  | 0.9187 | 0.8295 |
| FEV1/FVC | Boys | 3.50 | 109 | BCCG | 4.26297 | 0.9165 | 0.05032 |  | 0.9165 | 0.8276 |
| FEV1/FVC | Boys | 3.50 | 110 | BCCG | 4.26297 | 0.9143 | 0.05032 |  | 0.9143 | 0.8256 |
| FEV1/FVC | Boys | 3.50 | 111 | BCCG | 4.26297 | 0.9122 | 0.05032 |  | 0.9122 | 0.8237 |
| FEV1/FVC | Boys | 3.50 | 112 | BCCG | 4.26297 | 0.9101 | 0.05032 |  | 0.9101 | 0.8218 |
| FEV1/FVC | Boys | 3.50 | 113 | BCCG | 4.26297 | 0.9080 | 0.05032 |  | 0.9080 | 0.8199 |
| FEV1/FVC | Boys | 3.50 | 114 | BCCG | 4.26297 | 0.9059 | 0.05032 |  | 0.9059 | 0.8180 |
| FEV1/FVC | Boys | 3.50 | 115 | BCCG | 4.26297 | 0.9038 | 0.05032 |  | 0.9038 | 0.8161 |
| FEV1/FVC | Boys | 3.50 | 116 | BCCG | 4.26297 | 0.9018 | 0.05032 |  | 0.9018 | 0.8143 |
| FEV1/FVC | Boys | 3.50 | 117 | BCCG | 4.26297 | 0.8998 | 0.05032 |  | 0.8998 | 0.8125 |
| FEV1/FVC | Boys | 3.50 | 118 | BCCG | 4.26297 | 0.8978 | 0.05032 |  | 0.8978 | 0.8107 |
| FEV1/FVC | Boys | 3.50 | 119 | BCCG | 4.26297 | 0.8959 | 0.05032 |  | 0.8959 | 0.8089 |
| FEV1/FVC | Boys | 3.50 | 120 | BCCG | 4.26297 | 0.8939 | 0.05032 |  | 0.8939 | 0.8072 |
| FEV1/FVC | Boys | 3.50 | 121 | BCCG | 4.26297 | 0.8920 | 0.05032 |  | 0.8920 | 0.8055 |
| FEV1/FVC | Boys | 3.50 | 122 | BCCG | 4.26297 | 0.8901 | 0.05032 |  | 0.8901 | 0.8037 |
| FEV1/FVC | Boys | 3.50 | 123 | BCCG | 4.26297 | 0.8882 | 0.05032 |  | 0.8882 | 0.8020 |
| FEV1/FVC | Boys | 3.50 | 124 | BCCG | 4.26297 | 0.8864 | 0.05032 |  | 0.8864 | 0.8004 |
| FEV1/FVC | Boys | 3.50 | 125 | BCCG | 4.26297 | 0.8845 | 0.05032 |  | 0.8845 | 0.7987 |
| FEV1/FVC | Boys | 3.50 | 126 | BCCG | 4.26297 | 0.8827 | 0.05032 |  | 0.8827 | 0.7971 |
| FEV1/FVC | Boys | 3.50 | 127 | BCCG | 4.26297 | 0.8809 | 0.05032 |  | 0.8809 | 0.7954 |
| FEV1/FVC | Boys | 3.50 | 128 | BCCG | 4.26297 | 0.8791 | 0.05032 |  | 0.8791 | 0.7938 |
| FEV1/FVC | Boys | 3.50 | 129 | BCCG | 4.26297 | 0.8773 | 0.05032 |  | 0.8773 | 0.7922 |
| FEV1/FVC | Boys | 3.50 | 130 | BCCG | 4.26297 | 0.8756 | 0.05032 |  | 0.8756 | 0.7906 |
| FEV1/FVC | Boys | 3.50 | 131 | BCCG | 4.26297 | 0.8738 | 0.05032 |  | 0.8738 | 0.7891 |
| FEV1/FVC | Boys | 3.50 | 132 | BCCG | 4.26297 | 0.8721 | 0.05032 |  | 0.8721 | 0.7875 |
| FEV1/FVC | Boys | 3.75 | 79 | BCCG | 4.26297 | 1.0004 | 0.05032 |  | 1.0004 | 0.9033 |
| FEV1/FVC | Boys | 3.75 | 80 | BCCG | 4.26297 | 0.9971 | 0.05032 |  | 0.9971 | 0.9004 |
| FEV1/FVC | Boys | 3.75 | 81 | BCCG | 4.26297 | 0.9939 | 0.05032 |  | 0.9939 | 0.8975 |
| FEV1/FVC | Boys | 3.75 | 82 | BCCG | 4.26297 | 0.9908 | 0.05032 |  | 0.9908 | 0.8946 |
| FEV1/FVC | Boys | 3.75 | 83 | BCCG | 4.26297 | 0.9877 | 0.05032 |  | 0.9877 | 0.8918 |
| FEV1/FVC | Boys | 3.75 | 84 | BCCG | 4.26297 | 0.9846 | 0.05032 |  | 0.9846 | 0.8891 |
| FEV1/FVC | Boys | 3.75 | 85 | BCCG | 4.26297 | 0.9816 | 0.05032 |  | 0.9816 | 0.8863 |
| FEV1/FVC | Boys | 3.75 | 86 | BCCG | 4.26297 | 0.9786 | 0.05032 |  | 0.9786 | 0.8837 |
| FEV1/FVC | Boys | 3.75 | 87 | BCCG | 4.26297 | 0.9757 | 0.05032 |  | 0.9757 | 0.8810 |
| FEV1/FVC | Boys | 3.75 | 88 | BCCG | 4.26297 | 0.9728 | 0.05032 |  | 0.9728 | 0.8784 |
| FEV1/FVC | Boys | 3.75 | 89 | BCCG | 4.26297 | 0.9700 | 0.05032 |  | 0.9700 | 0.8758 |
| FEV1/FVC | Boys | 3.75 | 90 | BCCG | 4.26297 | 0.9672 | 0.05032 |  | 0.9672 | 0.8733 |
| FEV1/FVC | Boys | 3.75 | 91 | BCCG | 4.26297 | 0.9644 | 0.05032 |  | 0.9644 | 0.8708 |
| FEV1/FVC | Boys | 3.75 | 92 | BCCG | 4.26297 | 0.9617 | 0.05032 |  | 0.9617 | 0.8683 |
| FEV1/FVC | Boys | 3.75 | 93 | BCCG | 4.26297 | 0.9590 | 0.05032 |  | 0.9590 | 0.8659 |
| FEV1/FVC | Boys | 3.75 | 94 | BCCG | 4.26297 | 0.9563 | 0.05032 |  | 0.9563 | 0.8635 |
| FEV1/FVC | Boys | 3.75 | 95 | BCCG | 4.26297 | 0.9537 | 0.05032 |  | 0.9537 | 0.8612 |
| FEV1/FVC | Boys | 3.75 | 96 | BCCG | 4.26297 | 0.9511 | 0.05032 |  | 0.9511 | 0.8588 |
| FEV1/FVC | Boys | 3.75 | 97 | BCCG | 4.26297 | 0.9486 | 0.05032 |  | 0.9486 | 0.8565 |
| FEV1/FVC | Boys | 3.75 | 98 | BCCG | 4.26297 | 0.9460 | 0.05032 |  | 0.9460 | 0.8542 |
| FEV1/FVC | Boys | 3.75 | 99 | BCCG | 4.26297 | 0.9436 | 0.05032 |  | 0.9436 | 0.8520 |
| FEV1/FVC | Boys | 3.75 | 100 | BCCG | 4.26297 | 0.9411 | 0.05032 |  | 0.9411 | 0.8498 |
| FEV1/FVC | Boys | 3.75 | 101 | BCCG | 4.26297 | 0.9387 | 0.05032 |  | 0.9387 | 0.8476 |
| FEV1/FVC | Boys | 3.75 | 102 | BCCG | 4.26297 | 0.9363 | 0.05032 |  | 0.9363 | 0.8454 |
| FEV1/FVC | Boys | 3.75 | 103 | BCCG | 4.26297 | 0.9339 | 0.05032 |  | 0.9339 | 0.8433 |
| FEV1/FVC | Boys | 3.75 | 104 | BCCG | 4.26297 | 0.9316 | 0.05032 |  | 0.9316 | 0.8412 |
| FEV1/FVC | Boys | 3.75 | 105 | BCCG | 4.26297 | 0.9293 | 0.05032 |  | 0.9293 | 0.8391 |
| FEV1/FVC | Boys | 3.75 | 106 | BCCG | 4.26297 | 0.9270 | 0.05032 |  | 0.9270 | 0.8371 |
| FEV1/FVC | Boys | 3.75 | 107 | BCCG | 4.26297 | 0.9247 | 0.05032 |  | 0.9247 | 0.8350 |
| FEV1/FVC | Boys | 3.75 | 108 | BCCG | 4.26297 | 0.9225 | 0.05032 |  | 0.9225 | 0.8330 |
| FEV1/FVC | Boys | 3.75 | 109 | BCCG | 4.26297 | 0.9203 | 0.05032 |  | 0.9203 | 0.8310 |
| FEV1/FVC | Boys | 3.75 | 110 | BCCG | 4.26297 | 0.9181 | 0.05032 |  | 0.9181 | 0.8291 |
| FEV1/FVC | Boys | 3.75 | 111 | BCCG | 4.26297 | 0.9160 | 0.05032 |  | 0.9160 | 0.8271 |
| FEV1/FVC | Boys | 3.75 | 112 | BCCG | 4.26297 | 0.9139 | 0.05032 |  | 0.9139 | 0.8252 |
| FEV1/FVC | Boys | 3.75 | 113 | BCCG | 4.26297 | 0.9118 | 0.05032 |  | 0.9118 | 0.8233 |
| FEV1/FVC | Boys | 3.75 | 114 | BCCG | 4.26297 | 0.9097 | 0.05032 |  | 0.9097 | 0.8214 |
| FEV1/FVC | Boys | 3.75 | 115 | BCCG | 4.26297 | 0.9076 | 0.05032 |  | 0.9076 | 0.8196 |
| FEV1/FVC | Boys | 3.75 | 116 | BCCG | 4.26297 | 0.9056 | 0.05032 |  | 0.9056 | 0.8177 |
| FEV1/FVC | Boys | 3.75 | 117 | BCCG | 4.26297 | 0.9036 | 0.05032 |  | 0.9036 | 0.8159 |
| FEV1/FVC | Boys | 3.75 | 118 | BCCG | 4.26297 | 0.9016 | 0.05032 |  | 0.9016 | 0.8141 |
| FEV1/FVC | Boys | 3.75 | 119 | BCCG | 4.26297 | 0.8996 | 0.05032 |  | 0.8996 | 0.8123 |
| FEV1/FVC | Boys | 3.75 | 120 | BCCG | 4.26297 | 0.8977 | 0.05032 |  | 0.8977 | 0.8106 |
| FEV1/FVC | Boys | 3.75 | 121 | BCCG | 4.26297 | 0.8957 | 0.05032 |  | 0.8957 | 0.8088 |
| FEV1/FVC | Boys | 3.75 | 122 | BCCG | 4.26297 | 0.8938 | 0.05032 |  | 0.8938 | 0.8071 |
| FEV1/FVC | Boys | 3.75 | 123 | BCCG | 4.26297 | 0.8920 | 0.05032 |  | 0.8920 | 0.8054 |
| FEV1/FVC | Boys | 3.75 | 124 | BCCG | 4.26297 | 0.8901 | 0.05032 |  | 0.8901 | 0.8037 |
| FEV1/FVC | Boys | 3.75 | 125 | BCCG | 4.26297 | 0.8882 | 0.05032 |  | 0.8882 | 0.8020 |
| FEV1/FVC | Boys | 3.75 | 126 | BCCG | 4.26297 | 0.8864 | 0.05032 |  | 0.8864 | 0.8004 |
| FEV1/FVC | Boys | 3.75 | 127 | BCCG | 4.26297 | 0.8846 | 0.05032 |  | 0.8846 | 0.7988 |
| FEV1/FVC | Boys | 3.75 | 128 | BCCG | 4.26297 | 0.8828 | 0.05032 |  | 0.8828 | 0.7971 |
| FEV1/FVC | Boys | 3.75 | 129 | BCCG | 4.26297 | 0.8810 | 0.05032 |  | 0.8810 | 0.7955 |
| FEV1/FVC | Boys | 3.75 | 130 | BCCG | 4.26297 | 0.8793 | 0.05032 |  | 0.8793 | 0.7939 |
| FEV1/FVC | Boys | 3.75 | 131 | BCCG | 4.26297 | 0.8775 | 0.05032 |  | 0.8775 | 0.7924 |
| FEV1/FVC | Boys | 3.75 | 132 | BCCG | 4.26297 | 0.8758 | 0.05032 |  | 0.8758 | 0.7908 |
| FEV1/FVC | Boys | 4.00 | 79 | BCCG | 4.26297 | 1.0043 | 0.05032 |  | 1.0043 | 0.9068 |
| FEV1/FVC | Boys | 4.00 | 80 | BCCG | 4.26297 | 1.0010 | 0.05032 |  | 1.0010 | 0.9039 |
| FEV1/FVC | Boys | 4.00 | 81 | BCCG | 4.26297 | 0.9978 | 0.05032 |  | 0.9978 | 0.9010 |
| FEV1/FVC | Boys | 4.00 | 82 | BCCG | 4.26297 | 0.9946 | 0.05032 |  | 0.9946 | 0.8981 |
| FEV1/FVC | Boys | 4.00 | 83 | BCCG | 4.26297 | 0.9915 | 0.05032 |  | 0.9915 | 0.8953 |
| FEV1/FVC | Boys | 4.00 | 84 | BCCG | 4.26297 | 0.9885 | 0.05032 |  | 0.9885 | 0.8925 |
| FEV1/FVC | Boys | 4.00 | 85 | BCCG | 4.26297 | 0.9854 | 0.05032 |  | 0.9854 | 0.8898 |
| FEV1/FVC | Boys | 4.00 | 86 | BCCG | 4.26297 | 0.9824 | 0.05032 |  | 0.9824 | 0.8871 |
| FEV1/FVC | Boys | 4.00 | 87 | BCCG | 4.26297 | 0.9795 | 0.05032 |  | 0.9795 | 0.8845 |
| FEV1/FVC | Boys | 4.00 | 88 | BCCG | 4.26297 | 0.9766 | 0.05032 |  | 0.9766 | 0.8818 |
| FEV1/FVC | Boys | 4.00 | 89 | BCCG | 4.26297 | 0.9738 | 0.05032 |  | 0.9738 | 0.8793 |
| FEV1/FVC | Boys | 4.00 | 90 | BCCG | 4.26297 | 0.9709 | 0.05032 |  | 0.9709 | 0.8767 |
| FEV1/FVC | Boys | 4.00 | 91 | BCCG | 4.26297 | 0.9682 | 0.05032 |  | 0.9682 | 0.8742 |
| FEV1/FVC | Boys | 4.00 | 92 | BCCG | 4.26297 | 0.9654 | 0.05032 |  | 0.9654 | 0.8717 |
| FEV1/FVC | Boys | 4.00 | 93 | BCCG | 4.26297 | 0.9627 | 0.05032 |  | 0.9627 | 0.8693 |
| FEV1/FVC | Boys | 4.00 | 94 | BCCG | 4.26297 | 0.9601 | 0.05032 |  | 0.9601 | 0.8669 |
| FEV1/FVC | Boys | 4.00 | 95 | BCCG | 4.26297 | 0.9574 | 0.05032 |  | 0.9574 | 0.8645 |
| FEV1/FVC | Boys | 4.00 | 96 | BCCG | 4.26297 | 0.9548 | 0.05032 |  | 0.9548 | 0.8622 |
| FEV1/FVC | Boys | 4.00 | 97 | BCCG | 4.26297 | 0.9523 | 0.05032 |  | 0.9523 | 0.8599 |
| FEV1/FVC | Boys | 4.00 | 98 | BCCG | 4.26297 | 0.9497 | 0.05032 |  | 0.9497 | 0.8576 |
| FEV1/FVC | Boys | 4.00 | 99 | BCCG | 4.26297 | 0.9473 | 0.05032 |  | 0.9473 | 0.8553 |
| FEV1/FVC | Boys | 4.00 | 100 | BCCG | 4.26297 | 0.9448 | 0.05032 |  | 0.9448 | 0.8531 |
| FEV1/FVC | Boys | 4.00 | 101 | BCCG | 4.26297 | 0.9424 | 0.05032 |  | 0.9424 | 0.8509 |
| FEV1/FVC | Boys | 4.00 | 102 | BCCG | 4.26297 | 0.9400 | 0.05032 |  | 0.9400 | 0.8487 |
| FEV1/FVC | Boys | 4.00 | 103 | BCCG | 4.26297 | 0.9376 | 0.05032 |  | 0.9376 | 0.8466 |
| FEV1/FVC | Boys | 4.00 | 104 | BCCG | 4.26297 | 0.9352 | 0.05032 |  | 0.9352 | 0.8445 |
| FEV1/FVC | Boys | 4.00 | 105 | BCCG | 4.26297 | 0.9329 | 0.05032 |  | 0.9329 | 0.8424 |
| FEV1/FVC | Boys | 4.00 | 106 | BCCG | 4.26297 | 0.9306 | 0.05032 |  | 0.9306 | 0.8403 |
| FEV1/FVC | Boys | 4.00 | 107 | BCCG | 4.26297 | 0.9284 | 0.05032 |  | 0.9284 | 0.8383 |
| FEV1/FVC | Boys | 4.00 | 108 | BCCG | 4.26297 | 0.9261 | 0.05032 |  | 0.9261 | 0.8363 |
| FEV1/FVC | Boys | 4.00 | 109 | BCCG | 4.26297 | 0.9239 | 0.05032 |  | 0.9239 | 0.8343 |
| FEV1/FVC | Boys | 4.00 | 110 | BCCG | 4.26297 | 0.9217 | 0.05032 |  | 0.9217 | 0.8323 |
| FEV1/FVC | Boys | 4.00 | 111 | BCCG | 4.26297 | 0.9196 | 0.05032 |  | 0.9196 | 0.8304 |
| FEV1/FVC | Boys | 4.00 | 112 | BCCG | 4.26297 | 0.9174 | 0.05032 |  | 0.9174 | 0.8284 |
| FEV1/FVC | Boys | 4.00 | 113 | BCCG | 4.26297 | 0.9153 | 0.05032 |  | 0.9153 | 0.8265 |
| FEV1/FVC | Boys | 4.00 | 114 | BCCG | 4.26297 | 0.9132 | 0.05032 |  | 0.9132 | 0.8246 |
| FEV1/FVC | Boys | 4.00 | 115 | BCCG | 4.26297 | 0.9112 | 0.05032 |  | 0.9112 | 0.8228 |
| FEV1/FVC | Boys | 4.00 | 116 | BCCG | 4.26297 | 0.9091 | 0.05032 |  | 0.9091 | 0.8209 |
| FEV1/FVC | Boys | 4.00 | 117 | BCCG | 4.26297 | 0.9071 | 0.05032 |  | 0.9071 | 0.8191 |
| FEV1/FVC | Boys | 4.00 | 118 | BCCG | 4.26297 | 0.9051 | 0.05032 |  | 0.9051 | 0.8173 |
| FEV1/FVC | Boys | 4.00 | 119 | BCCG | 4.26297 | 0.9031 | 0.05032 |  | 0.9031 | 0.8155 |
| FEV1/FVC | Boys | 4.00 | 120 | BCCG | 4.26297 | 0.9012 | 0.05032 |  | 0.9012 | 0.8137 |
| FEV1/FVC | Boys | 4.00 | 121 | BCCG | 4.26297 | 0.8993 | 0.05032 |  | 0.8993 | 0.8120 |
| FEV1/FVC | Boys | 4.00 | 122 | BCCG | 4.26297 | 0.8973 | 0.05032 |  | 0.8973 | 0.8103 |
| FEV1/FVC | Boys | 4.00 | 123 | BCCG | 4.26297 | 0.8954 | 0.05032 |  | 0.8954 | 0.8086 |
| FEV1/FVC | Boys | 4.00 | 124 | BCCG | 4.26297 | 0.8936 | 0.05032 |  | 0.8936 | 0.8069 |
| FEV1/FVC | Boys | 4.00 | 125 | BCCG | 4.26297 | 0.8917 | 0.05032 |  | 0.8917 | 0.8052 |
| FEV1/FVC | Boys | 4.00 | 126 | BCCG | 4.26297 | 0.8899 | 0.05032 |  | 0.8899 | 0.8035 |
| FEV1/FVC | Boys | 4.00 | 127 | BCCG | 4.26297 | 0.8880 | 0.05032 |  | 0.8880 | 0.8019 |
| FEV1/FVC | Boys | 4.00 | 128 | BCCG | 4.26297 | 0.8862 | 0.05032 |  | 0.8862 | 0.8003 |
| FEV1/FVC | Boys | 4.00 | 129 | BCCG | 4.26297 | 0.8845 | 0.05032 |  | 0.8845 | 0.7986 |
| FEV1/FVC | Boys | 4.00 | 130 | BCCG | 4.26297 | 0.8827 | 0.05032 |  | 0.8827 | 0.7970 |
| FEV1/FVC | Boys | 4.00 | 131 | BCCG | 4.26297 | 0.8809 | 0.05032 |  | 0.8809 | 0.7955 |
| FEV1/FVC | Boys | 4.00 | 132 | BCCG | 4.26297 | 0.8792 | 0.05032 |  | 0.8792 | 0.7939 |
| FEV1/FVC | Boys | 4.25 | 79 | BCCG | 4.26297 | 1.0080 | 0.05032 |  | 1.0080 | 0.9102 |
| FEV1/FVC | Boys | 4.25 | 80 | BCCG | 4.26297 | 1.0047 | 0.05032 |  | 1.0047 | 0.9072 |
| FEV1/FVC | Boys | 4.25 | 81 | BCCG | 4.26297 | 1.0015 | 0.05032 |  | 1.0015 | 0.9043 |
| FEV1/FVC | Boys | 4.25 | 82 | BCCG | 4.26297 | 0.9983 | 0.05032 |  | 0.9983 | 0.9014 |
| FEV1/FVC | Boys | 4.25 | 83 | BCCG | 4.26297 | 0.9952 | 0.05032 |  | 0.9952 | 0.8986 |
| FEV1/FVC | Boys | 4.25 | 84 | BCCG | 4.26297 | 0.9921 | 0.05032 |  | 0.9921 | 0.8958 |
| FEV1/FVC | Boys | 4.25 | 85 | BCCG | 4.26297 | 0.9890 | 0.05032 |  | 0.9890 | 0.8931 |
| FEV1/FVC | Boys | 4.25 | 86 | BCCG | 4.26297 | 0.9861 | 0.05032 |  | 0.9861 | 0.8904 |
| FEV1/FVC | Boys | 4.25 | 87 | BCCG | 4.26297 | 0.9831 | 0.05032 |  | 0.9831 | 0.8877 |
| FEV1/FVC | Boys | 4.25 | 88 | BCCG | 4.26297 | 0.9802 | 0.05032 |  | 0.9802 | 0.8851 |
| FEV1/FVC | Boys | 4.25 | 89 | BCCG | 4.26297 | 0.9773 | 0.05032 |  | 0.9773 | 0.8825 |
| FEV1/FVC | Boys | 4.25 | 90 | BCCG | 4.26297 | 0.9745 | 0.05032 |  | 0.9745 | 0.8799 |
| FEV1/FVC | Boys | 4.25 | 91 | BCCG | 4.26297 | 0.9717 | 0.05032 |  | 0.9717 | 0.8774 |
| FEV1/FVC | Boys | 4.25 | 92 | BCCG | 4.26297 | 0.9690 | 0.05032 |  | 0.9690 | 0.8750 |
| FEV1/FVC | Boys | 4.25 | 93 | BCCG | 4.26297 | 0.9663 | 0.05032 |  | 0.9663 | 0.8725 |
| FEV1/FVC | Boys | 4.25 | 94 | BCCG | 4.26297 | 0.9636 | 0.05032 |  | 0.9636 | 0.8701 |
| FEV1/FVC | Boys | 4.25 | 95 | BCCG | 4.26297 | 0.9610 | 0.05032 |  | 0.9610 | 0.8677 |
| FEV1/FVC | Boys | 4.25 | 96 | BCCG | 4.26297 | 0.9583 | 0.05032 |  | 0.9583 | 0.8654 |
| FEV1/FVC | Boys | 4.25 | 97 | BCCG | 4.26297 | 0.9558 | 0.05032 |  | 0.9558 | 0.8630 |
| FEV1/FVC | Boys | 4.25 | 98 | BCCG | 4.26297 | 0.9532 | 0.05032 |  | 0.9532 | 0.8607 |
| FEV1/FVC | Boys | 4.25 | 99 | BCCG | 4.26297 | 0.9507 | 0.05032 |  | 0.9507 | 0.8585 |
| FEV1/FVC | Boys | 4.25 | 100 | BCCG | 4.26297 | 0.9483 | 0.05032 |  | 0.9483 | 0.8562 |
| FEV1/FVC | Boys | 4.25 | 101 | BCCG | 4.26297 | 0.9458 | 0.05032 |  | 0.9458 | 0.8540 |
| FEV1/FVC | Boys | 4.25 | 102 | BCCG | 4.26297 | 0.9434 | 0.05032 |  | 0.9434 | 0.8519 |
| FEV1/FVC | Boys | 4.25 | 103 | BCCG | 4.26297 | 0.9410 | 0.05032 |  | 0.9410 | 0.8497 |
| FEV1/FVC | Boys | 4.25 | 104 | BCCG | 4.26297 | 0.9387 | 0.05032 |  | 0.9387 | 0.8476 |
| FEV1/FVC | Boys | 4.25 | 105 | BCCG | 4.26297 | 0.9363 | 0.05032 |  | 0.9363 | 0.8455 |
| FEV1/FVC | Boys | 4.25 | 106 | BCCG | 4.26297 | 0.9341 | 0.05032 |  | 0.9341 | 0.8434 |
| FEV1/FVC | Boys | 4.25 | 107 | BCCG | 4.26297 | 0.9318 | 0.05032 |  | 0.9318 | 0.8414 |
| FEV1/FVC | Boys | 4.25 | 108 | BCCG | 4.26297 | 0.9295 | 0.05032 |  | 0.9295 | 0.8393 |
| FEV1/FVC | Boys | 4.25 | 109 | BCCG | 4.26297 | 0.9273 | 0.05032 |  | 0.9273 | 0.8373 |
| FEV1/FVC | Boys | 4.25 | 110 | BCCG | 4.26297 | 0.9251 | 0.05032 |  | 0.9251 | 0.8354 |
| FEV1/FVC | Boys | 4.25 | 111 | BCCG | 4.26297 | 0.9230 | 0.05032 |  | 0.9230 | 0.8334 |
| FEV1/FVC | Boys | 4.25 | 112 | BCCG | 4.26297 | 0.9208 | 0.05032 |  | 0.9208 | 0.8315 |
| FEV1/FVC | Boys | 4.25 | 113 | BCCG | 4.26297 | 0.9187 | 0.05032 |  | 0.9187 | 0.8296 |
| FEV1/FVC | Boys | 4.25 | 114 | BCCG | 4.26297 | 0.9166 | 0.05032 |  | 0.9166 | 0.8277 |
| FEV1/FVC | Boys | 4.25 | 115 | BCCG | 4.26297 | 0.9145 | 0.05032 |  | 0.9145 | 0.8258 |
| FEV1/FVC | Boys | 4.25 | 116 | BCCG | 4.26297 | 0.9125 | 0.05032 |  | 0.9125 | 0.8239 |
| FEV1/FVC | Boys | 4.25 | 117 | BCCG | 4.26297 | 0.9105 | 0.05032 |  | 0.9105 | 0.8221 |
| FEV1/FVC | Boys | 4.25 | 118 | BCCG | 4.26297 | 0.9085 | 0.05032 |  | 0.9085 | 0.8203 |
| FEV1/FVC | Boys | 4.25 | 119 | BCCG | 4.26297 | 0.9065 | 0.05032 |  | 0.9065 | 0.8185 |
| FEV1/FVC | Boys | 4.25 | 120 | BCCG | 4.26297 | 0.9045 | 0.05032 |  | 0.9045 | 0.8167 |
| FEV1/FVC | Boys | 4.25 | 121 | BCCG | 4.26297 | 0.9026 | 0.05032 |  | 0.9026 | 0.8150 |
| FEV1/FVC | Boys | 4.25 | 122 | BCCG | 4.26297 | 0.9006 | 0.05032 |  | 0.9006 | 0.8132 |
| FEV1/FVC | Boys | 4.25 | 123 | BCCG | 4.26297 | 0.8987 | 0.05032 |  | 0.8987 | 0.8115 |
| FEV1/FVC | Boys | 4.25 | 124 | BCCG | 4.26297 | 0.8969 | 0.05032 |  | 0.8969 | 0.8098 |
| FEV1/FVC | Boys | 4.25 | 125 | BCCG | 4.26297 | 0.8950 | 0.05032 |  | 0.8950 | 0.8081 |
| FEV1/FVC | Boys | 4.25 | 126 | BCCG | 4.26297 | 0.8931 | 0.05032 |  | 0.8931 | 0.8065 |
| FEV1/FVC | Boys | 4.25 | 127 | BCCG | 4.26297 | 0.8913 | 0.05032 |  | 0.8913 | 0.8048 |
| FEV1/FVC | Boys | 4.25 | 128 | BCCG | 4.26297 | 0.8895 | 0.05032 |  | 0.8895 | 0.8032 |
| FEV1/FVC | Boys | 4.25 | 129 | BCCG | 4.26297 | 0.8877 | 0.05032 |  | 0.8877 | 0.8016 |
| FEV1/FVC | Boys | 4.25 | 130 | BCCG | 4.26297 | 0.8859 | 0.05032 |  | 0.8859 | 0.8000 |
| FEV1/FVC | Boys | 4.25 | 131 | BCCG | 4.26297 | 0.8842 | 0.05032 |  | 0.8842 | 0.7984 |
| FEV1/FVC | Boys | 4.25 | 132 | BCCG | 4.26297 | 0.8824 | 0.05032 |  | 0.8824 | 0.7968 |
| FEV1/FVC | Boys | 4.50 | 79 | BCCG | 4.26297 | 1.0115 | 0.05032 |  | 1.0115 | 0.9133 |
| FEV1/FVC | Boys | 4.50 | 80 | BCCG | 4.26297 | 1.0082 | 0.05032 |  | 1.0082 | 0.9104 |
| FEV1/FVC | Boys | 4.50 | 81 | BCCG | 4.26297 | 1.0050 | 0.05032 |  | 1.0050 | 0.9074 |
| FEV1/FVC | Boys | 4.50 | 82 | BCCG | 4.26297 | 1.0018 | 0.05032 |  | 1.0018 | 0.9046 |
| FEV1/FVC | Boys | 4.50 | 83 | BCCG | 4.26297 | 0.9986 | 0.05032 |  | 0.9986 | 0.9017 |
| FEV1/FVC | Boys | 4.50 | 84 | BCCG | 4.26297 | 0.9955 | 0.05032 |  | 0.9955 | 0.8989 |
| FEV1/FVC | Boys | 4.50 | 85 | BCCG | 4.26297 | 0.9925 | 0.05032 |  | 0.9925 | 0.8962 |
| FEV1/FVC | Boys | 4.50 | 86 | BCCG | 4.26297 | 0.9895 | 0.05032 |  | 0.9895 | 0.8935 |
| FEV1/FVC | Boys | 4.50 | 87 | BCCG | 4.26297 | 0.9865 | 0.05032 |  | 0.9865 | 0.8908 |
| FEV1/FVC | Boys | 4.50 | 88 | BCCG | 4.26297 | 0.9836 | 0.05032 |  | 0.9836 | 0.8882 |
| FEV1/FVC | Boys | 4.50 | 89 | BCCG | 4.26297 | 0.9807 | 0.05032 |  | 0.9807 | 0.8856 |
| FEV1/FVC | Boys | 4.50 | 90 | BCCG | 4.26297 | 0.9779 | 0.05032 |  | 0.9779 | 0.8830 |
| FEV1/FVC | Boys | 4.50 | 91 | BCCG | 4.26297 | 0.9751 | 0.05032 |  | 0.9751 | 0.8805 |
| FEV1/FVC | Boys | 4.50 | 92 | BCCG | 4.26297 | 0.9723 | 0.05032 |  | 0.9723 | 0.8780 |
| FEV1/FVC | Boys | 4.50 | 93 | BCCG | 4.26297 | 0.9696 | 0.05032 |  | 0.9696 | 0.8755 |
| FEV1/FVC | Boys | 4.50 | 94 | BCCG | 4.26297 | 0.9669 | 0.05032 |  | 0.9669 | 0.8731 |
| FEV1/FVC | Boys | 4.50 | 95 | BCCG | 4.26297 | 0.9643 | 0.05032 |  | 0.9643 | 0.8707 |
| FEV1/FVC | Boys | 4.50 | 96 | BCCG | 4.26297 | 0.9617 | 0.05032 |  | 0.9617 | 0.8684 |
| FEV1/FVC | Boys | 4.50 | 97 | BCCG | 4.26297 | 0.9591 | 0.05032 |  | 0.9591 | 0.8660 |
| FEV1/FVC | Boys | 4.50 | 98 | BCCG | 4.26297 | 0.9565 | 0.05032 |  | 0.9565 | 0.8637 |
| FEV1/FVC | Boys | 4.50 | 99 | BCCG | 4.26297 | 0.9540 | 0.05032 |  | 0.9540 | 0.8615 |
| FEV1/FVC | Boys | 4.50 | 100 | BCCG | 4.26297 | 0.9515 | 0.05032 |  | 0.9515 | 0.8592 |
| FEV1/FVC | Boys | 4.50 | 101 | BCCG | 4.26297 | 0.9491 | 0.05032 |  | 0.9491 | 0.8570 |
| FEV1/FVC | Boys | 4.50 | 102 | BCCG | 4.26297 | 0.9467 | 0.05032 |  | 0.9467 | 0.8548 |
| FEV1/FVC | Boys | 4.50 | 103 | BCCG | 4.26297 | 0.9443 | 0.05032 |  | 0.9443 | 0.8527 |
| FEV1/FVC | Boys | 4.50 | 104 | BCCG | 4.26297 | 0.9419 | 0.05032 |  | 0.9419 | 0.8505 |
| FEV1/FVC | Boys | 4.50 | 105 | BCCG | 4.26297 | 0.9396 | 0.05032 |  | 0.9396 | 0.8484 |
| FEV1/FVC | Boys | 4.50 | 106 | BCCG | 4.26297 | 0.9373 | 0.05032 |  | 0.9373 | 0.8463 |
| FEV1/FVC | Boys | 4.50 | 107 | BCCG | 4.26297 | 0.9350 | 0.05032 |  | 0.9350 | 0.8443 |
| FEV1/FVC | Boys | 4.50 | 108 | BCCG | 4.26297 | 0.9328 | 0.05032 |  | 0.9328 | 0.8423 |
| FEV1/FVC | Boys | 4.50 | 109 | BCCG | 4.26297 | 0.9305 | 0.05032 |  | 0.9305 | 0.8402 |
| FEV1/FVC | Boys | 4.50 | 110 | BCCG | 4.26297 | 0.9283 | 0.05032 |  | 0.9283 | 0.8383 |
| FEV1/FVC | Boys | 4.50 | 111 | BCCG | 4.26297 | 0.9262 | 0.05032 |  | 0.9262 | 0.8363 |
| FEV1/FVC | Boys | 4.50 | 112 | BCCG | 4.26297 | 0.9240 | 0.05032 |  | 0.9240 | 0.8344 |
| FEV1/FVC | Boys | 4.50 | 113 | BCCG | 4.26297 | 0.9219 | 0.05032 |  | 0.9219 | 0.8324 |
| FEV1/FVC | Boys | 4.50 | 114 | BCCG | 4.26297 | 0.9198 | 0.05032 |  | 0.9198 | 0.8305 |
| FEV1/FVC | Boys | 4.50 | 115 | BCCG | 4.26297 | 0.9177 | 0.05032 |  | 0.9177 | 0.8287 |
| FEV1/FVC | Boys | 4.50 | 116 | BCCG | 4.26297 | 0.9156 | 0.05032 |  | 0.9156 | 0.8268 |
| FEV1/FVC | Boys | 4.50 | 117 | BCCG | 4.26297 | 0.9136 | 0.05032 |  | 0.9136 | 0.8250 |
| FEV1/FVC | Boys | 4.50 | 118 | BCCG | 4.26297 | 0.9116 | 0.05032 |  | 0.9116 | 0.8231 |
| FEV1/FVC | Boys | 4.50 | 119 | BCCG | 4.26297 | 0.9096 | 0.05032 |  | 0.9096 | 0.8213 |
| FEV1/FVC | Boys | 4.50 | 120 | BCCG | 4.26297 | 0.9076 | 0.05032 |  | 0.9076 | 0.8196 |
| FEV1/FVC | Boys | 4.50 | 121 | BCCG | 4.26297 | 0.9057 | 0.05032 |  | 0.9057 | 0.8178 |
| FEV1/FVC | Boys | 4.50 | 122 | BCCG | 4.26297 | 0.9038 | 0.05032 |  | 0.9038 | 0.8161 |
| FEV1/FVC | Boys | 4.50 | 123 | BCCG | 4.26297 | 0.9019 | 0.05032 |  | 0.9019 | 0.8143 |
| FEV1/FVC | Boys | 4.50 | 124 | BCCG | 4.26297 | 0.9000 | 0.05032 |  | 0.9000 | 0.8126 |
| FEV1/FVC | Boys | 4.50 | 125 | BCCG | 4.26297 | 0.8981 | 0.05032 |  | 0.8981 | 0.8109 |
| FEV1/FVC | Boys | 4.50 | 126 | BCCG | 4.26297 | 0.8962 | 0.05032 |  | 0.8962 | 0.8093 |
| FEV1/FVC | Boys | 4.50 | 127 | BCCG | 4.26297 | 0.8944 | 0.05032 |  | 0.8944 | 0.8076 |
| FEV1/FVC | Boys | 4.50 | 128 | BCCG | 4.26297 | 0.8926 | 0.05032 |  | 0.8926 | 0.8060 |
| FEV1/FVC | Boys | 4.50 | 129 | BCCG | 4.26297 | 0.8908 | 0.05032 |  | 0.8908 | 0.8044 |
| FEV1/FVC | Boys | 4.50 | 130 | BCCG | 4.26297 | 0.8890 | 0.05032 |  | 0.8890 | 0.8027 |
| FEV1/FVC | Boys | 4.50 | 131 | BCCG | 4.26297 | 0.8872 | 0.05032 |  | 0.8872 | 0.8012 |
| FEV1/FVC | Boys | 4.50 | 132 | BCCG | 4.26297 | 0.8855 | 0.05032 |  | 0.8855 | 0.7996 |
| FEV1/FVC | Boys | 4.75 | 79 | BCCG | 4.26297 | 1.0148 | 0.05032 |  | 1.0148 | 0.9163 |
| FEV1/FVC | Boys | 4.75 | 80 | BCCG | 4.26297 | 1.0115 | 0.05032 |  | 1.0115 | 0.9133 |
| FEV1/FVC | Boys | 4.75 | 81 | BCCG | 4.26297 | 1.0082 | 0.05032 |  | 1.0082 | 0.9104 |
| FEV1/FVC | Boys | 4.75 | 82 | BCCG | 4.26297 | 1.0050 | 0.05032 |  | 1.0050 | 0.9075 |
| FEV1/FVC | Boys | 4.75 | 83 | BCCG | 4.26297 | 1.0019 | 0.05032 |  | 1.0019 | 0.9047 |
| FEV1/FVC | Boys | 4.75 | 84 | BCCG | 4.26297 | 0.9988 | 0.05032 |  | 0.9988 | 0.9019 |
| FEV1/FVC | Boys | 4.75 | 85 | BCCG | 4.26297 | 0.9957 | 0.05032 |  | 0.9957 | 0.8991 |
| FEV1/FVC | Boys | 4.75 | 86 | BCCG | 4.26297 | 0.9927 | 0.05032 |  | 0.9927 | 0.8964 |
| FEV1/FVC | Boys | 4.75 | 87 | BCCG | 4.26297 | 0.9897 | 0.05032 |  | 0.9897 | 0.8937 |
| FEV1/FVC | Boys | 4.75 | 88 | BCCG | 4.26297 | 0.9868 | 0.05032 |  | 0.9868 | 0.8911 |
| FEV1/FVC | Boys | 4.75 | 89 | BCCG | 4.26297 | 0.9839 | 0.05032 |  | 0.9839 | 0.8885 |
| FEV1/FVC | Boys | 4.75 | 90 | BCCG | 4.26297 | 0.9811 | 0.05032 |  | 0.9811 | 0.8859 |
| FEV1/FVC | Boys | 4.75 | 91 | BCCG | 4.26297 | 0.9783 | 0.05032 |  | 0.9783 | 0.8834 |
| FEV1/FVC | Boys | 4.75 | 92 | BCCG | 4.26297 | 0.9755 | 0.05032 |  | 0.9755 | 0.8809 |
| FEV1/FVC | Boys | 4.75 | 93 | BCCG | 4.26297 | 0.9728 | 0.05032 |  | 0.9728 | 0.8784 |
| FEV1/FVC | Boys | 4.75 | 94 | BCCG | 4.26297 | 0.9701 | 0.05032 |  | 0.9701 | 0.8760 |
| FEV1/FVC | Boys | 4.75 | 95 | BCCG | 4.26297 | 0.9674 | 0.05032 |  | 0.9674 | 0.8736 |
| FEV1/FVC | Boys | 4.75 | 96 | BCCG | 4.26297 | 0.9648 | 0.05032 |  | 0.9648 | 0.8712 |
| FEV1/FVC | Boys | 4.75 | 97 | BCCG | 4.26297 | 0.9622 | 0.05032 |  | 0.9622 | 0.8689 |
| FEV1/FVC | Boys | 4.75 | 98 | BCCG | 4.26297 | 0.9597 | 0.05032 |  | 0.9597 | 0.8666 |
| FEV1/FVC | Boys | 4.75 | 99 | BCCG | 4.26297 | 0.9572 | 0.05032 |  | 0.9572 | 0.8643 |
| FEV1/FVC | Boys | 4.75 | 100 | BCCG | 4.26297 | 0.9547 | 0.05032 |  | 0.9547 | 0.8620 |
| FEV1/FVC | Boys | 4.75 | 101 | BCCG | 4.26297 | 0.9522 | 0.05032 |  | 0.9522 | 0.8598 |
| FEV1/FVC | Boys | 4.75 | 102 | BCCG | 4.26297 | 0.9498 | 0.05032 |  | 0.9498 | 0.8576 |
| FEV1/FVC | Boys | 4.75 | 103 | BCCG | 4.26297 | 0.9474 | 0.05032 |  | 0.9474 | 0.8555 |
| FEV1/FVC | Boys | 4.75 | 104 | BCCG | 4.26297 | 0.9450 | 0.05032 |  | 0.9450 | 0.8533 |
| FEV1/FVC | Boys | 4.75 | 105 | BCCG | 4.26297 | 0.9427 | 0.05032 |  | 0.9427 | 0.8512 |
| FEV1/FVC | Boys | 4.75 | 106 | BCCG | 4.26297 | 0.9404 | 0.05032 |  | 0.9404 | 0.8491 |
| FEV1/FVC | Boys | 4.75 | 107 | BCCG | 4.26297 | 0.9381 | 0.05032 |  | 0.9381 | 0.8471 |
| FEV1/FVC | Boys | 4.75 | 108 | BCCG | 4.26297 | 0.9358 | 0.05032 |  | 0.9358 | 0.8450 |
| FEV1/FVC | Boys | 4.75 | 109 | BCCG | 4.26297 | 0.9336 | 0.05032 |  | 0.9336 | 0.8430 |
| FEV1/FVC | Boys | 4.75 | 110 | BCCG | 4.26297 | 0.9314 | 0.05032 |  | 0.9314 | 0.8410 |
| FEV1/FVC | Boys | 4.75 | 111 | BCCG | 4.26297 | 0.9292 | 0.05032 |  | 0.9292 | 0.8390 |
| FEV1/FVC | Boys | 4.75 | 112 | BCCG | 4.26297 | 0.9270 | 0.05032 |  | 0.9270 | 0.8371 |
| FEV1/FVC | Boys | 4.75 | 113 | BCCG | 4.26297 | 0.9249 | 0.05032 |  | 0.9249 | 0.8352 |
| FEV1/FVC | Boys | 4.75 | 114 | BCCG | 4.26297 | 0.9228 | 0.05032 |  | 0.9228 | 0.8333 |
| FEV1/FVC | Boys | 4.75 | 115 | BCCG | 4.26297 | 0.9207 | 0.05032 |  | 0.9207 | 0.8314 |
| FEV1/FVC | Boys | 4.75 | 116 | BCCG | 4.26297 | 0.9187 | 0.05032 |  | 0.9187 | 0.8295 |
| FEV1/FVC | Boys | 4.75 | 117 | BCCG | 4.26297 | 0.9166 | 0.05032 |  | 0.9166 | 0.8277 |
| FEV1/FVC | Boys | 4.75 | 118 | BCCG | 4.26297 | 0.9146 | 0.05032 |  | 0.9146 | 0.8258 |
| FEV1/FVC | Boys | 4.75 | 119 | BCCG | 4.26297 | 0.9126 | 0.05032 |  | 0.9126 | 0.8240 |
| FEV1/FVC | Boys | 4.75 | 120 | BCCG | 4.26297 | 0.9106 | 0.05032 |  | 0.9106 | 0.8223 |
| FEV1/FVC | Boys | 4.75 | 121 | BCCG | 4.26297 | 0.9087 | 0.05032 |  | 0.9087 | 0.8205 |
| FEV1/FVC | Boys | 4.75 | 122 | BCCG | 4.26297 | 0.9067 | 0.05032 |  | 0.9067 | 0.8187 |
| FEV1/FVC | Boys | 4.75 | 123 | BCCG | 4.26297 | 0.9048 | 0.05032 |  | 0.9048 | 0.8170 |
| FEV1/FVC | Boys | 4.75 | 124 | BCCG | 4.26297 | 0.9029 | 0.05032 |  | 0.9029 | 0.8153 |
| FEV1/FVC | Boys | 4.75 | 125 | BCCG | 4.26297 | 0.9010 | 0.05032 |  | 0.9010 | 0.8136 |
| FEV1/FVC | Boys | 4.75 | 126 | BCCG | 4.26297 | 0.8992 | 0.05032 |  | 0.8992 | 0.8119 |
| FEV1/FVC | Boys | 4.75 | 127 | BCCG | 4.26297 | 0.8973 | 0.05032 |  | 0.8973 | 0.8103 |
| FEV1/FVC | Boys | 4.75 | 128 | BCCG | 4.26297 | 0.8955 | 0.05032 |  | 0.8955 | 0.8086 |
| FEV1/FVC | Boys | 4.75 | 129 | BCCG | 4.26297 | 0.8937 | 0.05032 |  | 0.8937 | 0.8070 |
| FEV1/FVC | Boys | 4.75 | 130 | BCCG | 4.26297 | 0.8919 | 0.05032 |  | 0.8919 | 0.8054 |
| FEV1/FVC | Boys | 4.75 | 131 | BCCG | 4.26297 | 0.8902 | 0.05032 |  | 0.8902 | 0.8038 |
| FEV1/FVC | Boys | 4.75 | 132 | BCCG | 4.26297 | 0.8884 | 0.05032 |  | 0.8884 | 0.8022 |
| FEV1/FVC | Boys | 5.00 | 79 | BCCG | 4.26297 | 1.0180 | 0.05032 |  | 1.0180 | 0.9192 |
| FEV1/FVC | Boys | 5.00 | 80 | BCCG | 4.26297 | 1.0146 | 0.05032 |  | 1.0146 | 0.9162 |
| FEV1/FVC | Boys | 5.00 | 81 | BCCG | 4.26297 | 1.0114 | 0.05032 |  | 1.0114 | 0.9132 |
| FEV1/FVC | Boys | 5.00 | 82 | BCCG | 4.26297 | 1.0082 | 0.05032 |  | 1.0082 | 0.9103 |
| FEV1/FVC | Boys | 5.00 | 83 | BCCG | 4.26297 | 1.0050 | 0.05032 |  | 1.0050 | 0.9075 |
| FEV1/FVC | Boys | 5.00 | 84 | BCCG | 4.26297 | 1.0019 | 0.05032 |  | 1.0019 | 0.9047 |
| FEV1/FVC | Boys | 5.00 | 85 | BCCG | 4.26297 | 0.9988 | 0.05032 |  | 0.9988 | 0.9019 |
| FEV1/FVC | Boys | 5.00 | 86 | BCCG | 4.26297 | 0.9958 | 0.05032 |  | 0.9958 | 0.8992 |
| FEV1/FVC | Boys | 5.00 | 87 | BCCG | 4.26297 | 0.9928 | 0.05032 |  | 0.9928 | 0.8965 |
| FEV1/FVC | Boys | 5.00 | 88 | BCCG | 4.26297 | 0.9899 | 0.05032 |  | 0.9899 | 0.8938 |
| FEV1/FVC | Boys | 5.00 | 89 | BCCG | 4.26297 | 0.9870 | 0.05032 |  | 0.9870 | 0.8912 |
| FEV1/FVC | Boys | 5.00 | 90 | BCCG | 4.26297 | 0.9841 | 0.05032 |  | 0.9841 | 0.8886 |
| FEV1/FVC | Boys | 5.00 | 91 | BCCG | 4.26297 | 0.9813 | 0.05032 |  | 0.9813 | 0.8861 |
| FEV1/FVC | Boys | 5.00 | 92 | BCCG | 4.26297 | 0.9786 | 0.05032 |  | 0.9786 | 0.8836 |
| FEV1/FVC | Boys | 5.00 | 93 | BCCG | 4.26297 | 0.9758 | 0.05032 |  | 0.9758 | 0.8811 |
| FEV1/FVC | Boys | 5.00 | 94 | BCCG | 4.26297 | 0.9731 | 0.05032 |  | 0.9731 | 0.8787 |
| FEV1/FVC | Boys | 5.00 | 95 | BCCG | 4.26297 | 0.9705 | 0.05032 |  | 0.9705 | 0.8763 |
| FEV1/FVC | Boys | 5.00 | 96 | BCCG | 4.26297 | 0.9678 | 0.05032 |  | 0.9678 | 0.8739 |
| FEV1/FVC | Boys | 5.00 | 97 | BCCG | 4.26297 | 0.9652 | 0.05032 |  | 0.9652 | 0.8716 |
| FEV1/FVC | Boys | 5.00 | 98 | BCCG | 4.26297 | 0.9627 | 0.05032 |  | 0.9627 | 0.8693 |
| FEV1/FVC | Boys | 5.00 | 99 | BCCG | 4.26297 | 0.9601 | 0.05032 |  | 0.9601 | 0.8670 |
| FEV1/FVC | Boys | 5.00 | 100 | BCCG | 4.26297 | 0.9576 | 0.05032 |  | 0.9576 | 0.8647 |
| FEV1/FVC | Boys | 5.00 | 101 | BCCG | 4.26297 | 0.9552 | 0.05032 |  | 0.9552 | 0.8625 |
| FEV1/FVC | Boys | 5.00 | 102 | BCCG | 4.26297 | 0.9527 | 0.05032 |  | 0.9527 | 0.8603 |
| FEV1/FVC | Boys | 5.00 | 103 | BCCG | 4.26297 | 0.9503 | 0.05032 |  | 0.9503 | 0.8581 |
| FEV1/FVC | Boys | 5.00 | 104 | BCCG | 4.26297 | 0.9480 | 0.05032 |  | 0.9480 | 0.8560 |
| FEV1/FVC | Boys | 5.00 | 105 | BCCG | 4.26297 | 0.9456 | 0.05032 |  | 0.9456 | 0.8539 |
| FEV1/FVC | Boys | 5.00 | 106 | BCCG | 4.26297 | 0.9433 | 0.05032 |  | 0.9433 | 0.8518 |
| FEV1/FVC | Boys | 5.00 | 107 | BCCG | 4.26297 | 0.9410 | 0.05032 |  | 0.9410 | 0.8497 |
| FEV1/FVC | Boys | 5.00 | 108 | BCCG | 4.26297 | 0.9387 | 0.05032 |  | 0.9387 | 0.8476 |
| FEV1/FVC | Boys | 5.00 | 109 | BCCG | 4.26297 | 0.9365 | 0.05032 |  | 0.9365 | 0.8456 |
| FEV1/FVC | Boys | 5.00 | 110 | BCCG | 4.26297 | 0.9343 | 0.05032 |  | 0.9343 | 0.8436 |
| FEV1/FVC | Boys | 5.00 | 111 | BCCG | 4.26297 | 0.9321 | 0.05032 |  | 0.9321 | 0.8416 |
| FEV1/FVC | Boys | 5.00 | 112 | BCCG | 4.26297 | 0.9299 | 0.05032 |  | 0.9299 | 0.8397 |
| FEV1/FVC | Boys | 5.00 | 113 | BCCG | 4.26297 | 0.9278 | 0.05032 |  | 0.9278 | 0.8378 |
| FEV1/FVC | Boys | 5.00 | 114 | BCCG | 4.26297 | 0.9257 | 0.05032 |  | 0.9257 | 0.8358 |
| FEV1/FVC | Boys | 5.00 | 115 | BCCG | 4.26297 | 0.9236 | 0.05032 |  | 0.9236 | 0.8340 |
| FEV1/FVC | Boys | 5.00 | 116 | BCCG | 4.26297 | 0.9215 | 0.05032 |  | 0.9215 | 0.8321 |
| FEV1/FVC | Boys | 5.00 | 117 | BCCG | 4.26297 | 0.9195 | 0.05032 |  | 0.9195 | 0.8302 |
| FEV1/FVC | Boys | 5.00 | 118 | BCCG | 4.26297 | 0.9174 | 0.05032 |  | 0.9174 | 0.8284 |
| FEV1/FVC | Boys | 5.00 | 119 | BCCG | 4.26297 | 0.9154 | 0.05032 |  | 0.9154 | 0.8266 |
| FEV1/FVC | Boys | 5.00 | 120 | BCCG | 4.26297 | 0.9134 | 0.05032 |  | 0.9134 | 0.8248 |
| FEV1/FVC | Boys | 5.00 | 121 | BCCG | 4.26297 | 0.9115 | 0.05032 |  | 0.9115 | 0.8230 |
| FEV1/FVC | Boys | 5.00 | 122 | BCCG | 4.26297 | 0.9095 | 0.05032 |  | 0.9095 | 0.8213 |
| FEV1/FVC | Boys | 5.00 | 123 | BCCG | 4.26297 | 0.9076 | 0.05032 |  | 0.9076 | 0.8196 |
| FEV1/FVC | Boys | 5.00 | 124 | BCCG | 4.26297 | 0.9057 | 0.05032 |  | 0.9057 | 0.8178 |
| FEV1/FVC | Boys | 5.00 | 125 | BCCG | 4.26297 | 0.9038 | 0.05032 |  | 0.9038 | 0.8161 |
| FEV1/FVC | Boys | 5.00 | 126 | BCCG | 4.26297 | 0.9020 | 0.05032 |  | 0.9020 | 0.8145 |
| FEV1/FVC | Boys | 5.00 | 127 | BCCG | 4.26297 | 0.9001 | 0.05032 |  | 0.9001 | 0.8128 |
| FEV1/FVC | Boys | 5.00 | 128 | BCCG | 4.26297 | 0.8983 | 0.05032 |  | 0.8983 | 0.8111 |
| FEV1/FVC | Boys | 5.00 | 129 | BCCG | 4.26297 | 0.8965 | 0.05032 |  | 0.8965 | 0.8095 |
| FEV1/FVC | Boys | 5.00 | 130 | BCCG | 4.26297 | 0.8947 | 0.05032 |  | 0.8947 | 0.8079 |
| FEV1/FVC | Boys | 5.00 | 131 | BCCG | 4.26297 | 0.8929 | 0.05032 |  | 0.8929 | 0.8063 |
| FEV1/FVC | Boys | 5.00 | 132 | BCCG | 4.26297 | 0.8912 | 0.05032 |  | 0.8912 | 0.8047 |
| FEV1/FVC | Boys | 5.25 | 79 | BCCG | 4.26297 | 1.0210 | 0.05032 |  | 1.0210 | 0.9219 |
| FEV1/FVC | Boys | 5.25 | 80 | BCCG | 4.26297 | 1.0176 | 0.05032 |  | 1.0176 | 0.9189 |
| FEV1/FVC | Boys | 5.25 | 81 | BCCG | 4.26297 | 1.0144 | 0.05032 |  | 1.0144 | 0.9159 |
| FEV1/FVC | Boys | 5.25 | 82 | BCCG | 4.26297 | 1.0112 | 0.05032 |  | 1.0112 | 0.9130 |
| FEV1/FVC | Boys | 5.25 | 83 | BCCG | 4.26297 | 1.0080 | 0.05032 |  | 1.0080 | 0.9102 |
| FEV1/FVC | Boys | 5.25 | 84 | BCCG | 4.26297 | 1.0049 | 0.05032 |  | 1.0049 | 0.9074 |
| FEV1/FVC | Boys | 5.25 | 85 | BCCG | 4.26297 | 1.0018 | 0.05032 |  | 1.0018 | 0.9046 |
| FEV1/FVC | Boys | 5.25 | 86 | BCCG | 4.26297 | 0.9988 | 0.05032 |  | 0.9988 | 0.9018 |
| FEV1/FVC | Boys | 5.25 | 87 | BCCG | 4.26297 | 0.9958 | 0.05032 |  | 0.9958 | 0.8991 |
| FEV1/FVC | Boys | 5.25 | 88 | BCCG | 4.26297 | 0.9928 | 0.05032 |  | 0.9928 | 0.8965 |
| FEV1/FVC | Boys | 5.25 | 89 | BCCG | 4.26297 | 0.9899 | 0.05032 |  | 0.9899 | 0.8939 |
| FEV1/FVC | Boys | 5.25 | 90 | BCCG | 4.26297 | 0.9871 | 0.05032 |  | 0.9871 | 0.8913 |
| FEV1/FVC | Boys | 5.25 | 91 | BCCG | 4.26297 | 0.9842 | 0.05032 |  | 0.9842 | 0.8887 |
| FEV1/FVC | Boys | 5.25 | 92 | BCCG | 4.26297 | 0.9814 | 0.05032 |  | 0.9814 | 0.8862 |
| FEV1/FVC | Boys | 5.25 | 93 | BCCG | 4.26297 | 0.9787 | 0.05032 |  | 0.9787 | 0.8837 |
| FEV1/FVC | Boys | 5.25 | 94 | BCCG | 4.26297 | 0.9760 | 0.05032 |  | 0.9760 | 0.8813 |
| FEV1/FVC | Boys | 5.25 | 95 | BCCG | 4.26297 | 0.9733 | 0.05032 |  | 0.9733 | 0.8789 |
| FEV1/FVC | Boys | 5.25 | 96 | BCCG | 4.26297 | 0.9707 | 0.05032 |  | 0.9707 | 0.8765 |
| FEV1/FVC | Boys | 5.25 | 97 | BCCG | 4.26297 | 0.9681 | 0.05032 |  | 0.9681 | 0.8741 |
| FEV1/FVC | Boys | 5.25 | 98 | BCCG | 4.26297 | 0.9655 | 0.05032 |  | 0.9655 | 0.8718 |
| FEV1/FVC | Boys | 5.25 | 99 | BCCG | 4.26297 | 0.9630 | 0.05032 |  | 0.9630 | 0.8695 |
| FEV1/FVC | Boys | 5.25 | 100 | BCCG | 4.26297 | 0.9605 | 0.05032 |  | 0.9605 | 0.8673 |
| FEV1/FVC | Boys | 5.25 | 101 | BCCG | 4.26297 | 0.9580 | 0.05032 |  | 0.9580 | 0.8650 |
| FEV1/FVC | Boys | 5.25 | 102 | BCCG | 4.26297 | 0.9556 | 0.05032 |  | 0.9556 | 0.8628 |
| FEV1/FVC | Boys | 5.25 | 103 | BCCG | 4.26297 | 0.9531 | 0.05032 |  | 0.9531 | 0.8607 |
| FEV1/FVC | Boys | 5.25 | 104 | BCCG | 4.26297 | 0.9508 | 0.05032 |  | 0.9508 | 0.8585 |
| FEV1/FVC | Boys | 5.25 | 105 | BCCG | 4.26297 | 0.9484 | 0.05032 |  | 0.9484 | 0.8564 |
| FEV1/FVC | Boys | 5.25 | 106 | BCCG | 4.26297 | 0.9461 | 0.05032 |  | 0.9461 | 0.8543 |
| FEV1/FVC | Boys | 5.25 | 107 | BCCG | 4.26297 | 0.9438 | 0.05032 |  | 0.9438 | 0.8522 |
| FEV1/FVC | Boys | 5.25 | 108 | BCCG | 4.26297 | 0.9415 | 0.05032 |  | 0.9415 | 0.8501 |
| FEV1/FVC | Boys | 5.25 | 109 | BCCG | 4.26297 | 0.9393 | 0.05032 |  | 0.9393 | 0.8481 |
| FEV1/FVC | Boys | 5.25 | 110 | BCCG | 4.26297 | 0.9370 | 0.05032 |  | 0.9370 | 0.8461 |
| FEV1/FVC | Boys | 5.25 | 111 | BCCG | 4.26297 | 0.9348 | 0.05032 |  | 0.9348 | 0.8441 |
| FEV1/FVC | Boys | 5.25 | 112 | BCCG | 4.26297 | 0.9327 | 0.05032 |  | 0.9327 | 0.8422 |
| FEV1/FVC | Boys | 5.25 | 113 | BCCG | 4.26297 | 0.9305 | 0.05032 |  | 0.9305 | 0.8402 |
| FEV1/FVC | Boys | 5.25 | 114 | BCCG | 4.26297 | 0.9284 | 0.05032 |  | 0.9284 | 0.8383 |
| FEV1/FVC | Boys | 5.25 | 115 | BCCG | 4.26297 | 0.9263 | 0.05032 |  | 0.9263 | 0.8364 |
| FEV1/FVC | Boys | 5.25 | 116 | BCCG | 4.26297 | 0.9242 | 0.05032 |  | 0.9242 | 0.8346 |
| FEV1/FVC | Boys | 5.25 | 117 | BCCG | 4.26297 | 0.9222 | 0.05032 |  | 0.9222 | 0.8327 |
| FEV1/FVC | Boys | 5.25 | 118 | BCCG | 4.26297 | 0.9201 | 0.05032 |  | 0.9201 | 0.8309 |
| FEV1/FVC | Boys | 5.25 | 119 | BCCG | 4.26297 | 0.9181 | 0.05032 |  | 0.9181 | 0.8290 |
| FEV1/FVC | Boys | 5.25 | 120 | BCCG | 4.26297 | 0.9162 | 0.05032 |  | 0.9162 | 0.8273 |
| FEV1/FVC | Boys | 5.25 | 121 | BCCG | 4.26297 | 0.9142 | 0.05032 |  | 0.9142 | 0.8255 |
| FEV1/FVC | Boys | 5.25 | 122 | BCCG | 4.26297 | 0.9122 | 0.05032 |  | 0.9122 | 0.8237 |
| FEV1/FVC | Boys | 5.25 | 123 | BCCG | 4.26297 | 0.9103 | 0.05032 |  | 0.9103 | 0.8220 |
| FEV1/FVC | Boys | 5.25 | 124 | BCCG | 4.26297 | 0.9084 | 0.05032 |  | 0.9084 | 0.8203 |
| FEV1/FVC | Boys | 5.25 | 125 | BCCG | 4.26297 | 0.9065 | 0.05032 |  | 0.9065 | 0.8185 |
| FEV1/FVC | Boys | 5.25 | 126 | BCCG | 4.26297 | 0.9046 | 0.05032 |  | 0.9046 | 0.8169 |
| FEV1/FVC | Boys | 5.25 | 127 | BCCG | 4.26297 | 0.9028 | 0.05032 |  | 0.9028 | 0.8152 |
| FEV1/FVC | Boys | 5.25 | 128 | BCCG | 4.26297 | 0.9010 | 0.05032 |  | 0.9010 | 0.8135 |
| FEV1/FVC | Boys | 5.25 | 129 | BCCG | 4.26297 | 0.8991 | 0.05032 |  | 0.8991 | 0.8119 |
| FEV1/FVC | Boys | 5.25 | 130 | BCCG | 4.26297 | 0.8973 | 0.05032 |  | 0.8973 | 0.8103 |
| FEV1/FVC | Boys | 5.25 | 131 | BCCG | 4.26297 | 0.8956 | 0.05032 |  | 0.8956 | 0.8087 |
| FEV1/FVC | Boys | 5.25 | 132 | BCCG | 4.26297 | 0.8938 | 0.05032 |  | 0.8938 | 0.8071 |
| FEV1/FVC | Boys | 5.50 | 79 | BCCG | 4.26297 | 1.0238 | 0.05032 |  | 1.0238 | 0.9245 |
| FEV1/FVC | Boys | 5.50 | 80 | BCCG | 4.26297 | 1.0205 | 0.05032 |  | 1.0205 | 0.9215 |
| FEV1/FVC | Boys | 5.50 | 81 | BCCG | 4.26297 | 1.0172 | 0.05032 |  | 1.0172 | 0.9185 |
| FEV1/FVC | Boys | 5.50 | 82 | BCCG | 4.26297 | 1.0140 | 0.05032 |  | 1.0140 | 0.9156 |
| FEV1/FVC | Boys | 5.50 | 83 | BCCG | 4.26297 | 1.0108 | 0.05032 |  | 1.0108 | 0.9127 |
| FEV1/FVC | Boys | 5.50 | 84 | BCCG | 4.26297 | 1.0077 | 0.05032 |  | 1.0077 | 0.9099 |
| FEV1/FVC | Boys | 5.50 | 85 | BCCG | 4.26297 | 1.0046 | 0.05032 |  | 1.0046 | 0.9071 |
| FEV1/FVC | Boys | 5.50 | 86 | BCCG | 4.26297 | 1.0016 | 0.05032 |  | 1.0016 | 0.9044 |
| FEV1/FVC | Boys | 5.50 | 87 | BCCG | 4.26297 | 0.9986 | 0.05032 |  | 0.9986 | 0.9017 |
| FEV1/FVC | Boys | 5.50 | 88 | BCCG | 4.26297 | 0.9956 | 0.05032 |  | 0.9956 | 0.8990 |
| FEV1/FVC | Boys | 5.50 | 89 | BCCG | 4.26297 | 0.9927 | 0.05032 |  | 0.9927 | 0.8964 |
| FEV1/FVC | Boys | 5.50 | 90 | BCCG | 4.26297 | 0.9898 | 0.05032 |  | 0.9898 | 0.8938 |
| FEV1/FVC | Boys | 5.50 | 91 | BCCG | 4.26297 | 0.9870 | 0.05032 |  | 0.9870 | 0.8912 |
| FEV1/FVC | Boys | 5.50 | 92 | BCCG | 4.26297 | 0.9842 | 0.05032 |  | 0.9842 | 0.8887 |
| FEV1/FVC | Boys | 5.50 | 93 | BCCG | 4.26297 | 0.9815 | 0.05032 |  | 0.9815 | 0.8862 |
| FEV1/FVC | Boys | 5.50 | 94 | BCCG | 4.26297 | 0.9787 | 0.05032 |  | 0.9787 | 0.8838 |
| FEV1/FVC | Boys | 5.50 | 95 | BCCG | 4.26297 | 0.9761 | 0.05032 |  | 0.9761 | 0.8814 |
| FEV1/FVC | Boys | 5.50 | 96 | BCCG | 4.26297 | 0.9734 | 0.05032 |  | 0.9734 | 0.8790 |
| FEV1/FVC | Boys | 5.50 | 97 | BCCG | 4.26297 | 0.9708 | 0.05032 |  | 0.9708 | 0.8766 |
| FEV1/FVC | Boys | 5.50 | 98 | BCCG | 4.26297 | 0.9682 | 0.05032 |  | 0.9682 | 0.8743 |
| FEV1/FVC | Boys | 5.50 | 99 | BCCG | 4.26297 | 0.9657 | 0.05032 |  | 0.9657 | 0.8720 |
| FEV1/FVC | Boys | 5.50 | 100 | BCCG | 4.26297 | 0.9632 | 0.05032 |  | 0.9632 | 0.8697 |
| FEV1/FVC | Boys | 5.50 | 101 | BCCG | 4.26297 | 0.9607 | 0.05032 |  | 0.9607 | 0.8675 |
| FEV1/FVC | Boys | 5.50 | 102 | BCCG | 4.26297 | 0.9583 | 0.05032 |  | 0.9583 | 0.8653 |
| FEV1/FVC | Boys | 5.50 | 103 | BCCG | 4.26297 | 0.9558 | 0.05032 |  | 0.9558 | 0.8631 |
| FEV1/FVC | Boys | 5.50 | 104 | BCCG | 4.26297 | 0.9534 | 0.05032 |  | 0.9534 | 0.8609 |
| FEV1/FVC | Boys | 5.50 | 105 | BCCG | 4.26297 | 0.9511 | 0.05032 |  | 0.9511 | 0.8588 |
| FEV1/FVC | Boys | 5.50 | 106 | BCCG | 4.26297 | 0.9487 | 0.05032 |  | 0.9487 | 0.8567 |
| FEV1/FVC | Boys | 5.50 | 107 | BCCG | 4.26297 | 0.9464 | 0.05032 |  | 0.9464 | 0.8546 |
| FEV1/FVC | Boys | 5.50 | 108 | BCCG | 4.26297 | 0.9442 | 0.05032 |  | 0.9442 | 0.8525 |
| FEV1/FVC | Boys | 5.50 | 109 | BCCG | 4.26297 | 0.9419 | 0.05032 |  | 0.9419 | 0.8505 |
| FEV1/FVC | Boys | 5.50 | 110 | BCCG | 4.26297 | 0.9397 | 0.05032 |  | 0.9397 | 0.8485 |
| FEV1/FVC | Boys | 5.50 | 111 | BCCG | 4.26297 | 0.9375 | 0.05032 |  | 0.9375 | 0.8465 |
| FEV1/FVC | Boys | 5.50 | 112 | BCCG | 4.26297 | 0.9353 | 0.05032 |  | 0.9353 | 0.8446 |
| FEV1/FVC | Boys | 5.50 | 113 | BCCG | 4.26297 | 0.9332 | 0.05032 |  | 0.9332 | 0.8426 |
| FEV1/FVC | Boys | 5.50 | 114 | BCCG | 4.26297 | 0.9310 | 0.05032 |  | 0.9310 | 0.8407 |
| FEV1/FVC | Boys | 5.50 | 115 | BCCG | 4.26297 | 0.9289 | 0.05032 |  | 0.9289 | 0.8388 |
| FEV1/FVC | Boys | 5.50 | 116 | BCCG | 4.26297 | 0.9268 | 0.05032 |  | 0.9268 | 0.8369 |
| FEV1/FVC | Boys | 5.50 | 117 | BCCG | 4.26297 | 0.9248 | 0.05032 |  | 0.9248 | 0.8350 |
| FEV1/FVC | Boys | 5.50 | 118 | BCCG | 4.26297 | 0.9227 | 0.05032 |  | 0.9227 | 0.8332 |
| FEV1/FVC | Boys | 5.50 | 119 | BCCG | 4.26297 | 0.9207 | 0.05032 |  | 0.9207 | 0.8314 |
| FEV1/FVC | Boys | 5.50 | 120 | BCCG | 4.26297 | 0.9187 | 0.05032 |  | 0.9187 | 0.8296 |
| FEV1/FVC | Boys | 5.50 | 121 | BCCG | 4.26297 | 0.9168 | 0.05032 |  | 0.9168 | 0.8278 |
| FEV1/FVC | Boys | 5.50 | 122 | BCCG | 4.26297 | 0.9148 | 0.05032 |  | 0.9148 | 0.8260 |
| FEV1/FVC | Boys | 5.50 | 123 | BCCG | 4.26297 | 0.9129 | 0.05032 |  | 0.9129 | 0.8243 |
| FEV1/FVC | Boys | 5.50 | 124 | BCCG | 4.26297 | 0.9110 | 0.05032 |  | 0.9110 | 0.8226 |
| FEV1/FVC | Boys | 5.50 | 125 | BCCG | 4.26297 | 0.9091 | 0.05032 |  | 0.9091 | 0.8209 |
| FEV1/FVC | Boys | 5.50 | 126 | BCCG | 4.26297 | 0.9072 | 0.05032 |  | 0.9072 | 0.8192 |
| FEV1/FVC | Boys | 5.50 | 127 | BCCG | 4.26297 | 0.9053 | 0.05032 |  | 0.9053 | 0.8175 |
| FEV1/FVC | Boys | 5.50 | 128 | BCCG | 4.26297 | 0.9035 | 0.05032 |  | 0.9035 | 0.8158 |
| FEV1/FVC | Boys | 5.50 | 129 | BCCG | 4.26297 | 0.9017 | 0.05032 |  | 0.9017 | 0.8142 |
| FEV1/FVC | Boys | 5.50 | 130 | BCCG | 4.26297 | 0.8999 | 0.05032 |  | 0.8999 | 0.8126 |
| FEV1/FVC | Boys | 5.50 | 131 | BCCG | 4.26297 | 0.8981 | 0.05032 |  | 0.8981 | 0.8109 |
| FEV1/FVC | Boys | 5.50 | 132 | BCCG | 4.26297 | 0.8963 | 0.05032 |  | 0.8963 | 0.8093 |
| FEV1/FVC | Boys | 5.75 | 79 | BCCG | 4.26297 | 1.0266 | 0.05032 |  | 1.0266 | 0.9270 |
| FEV1/FVC | Boys | 5.75 | 80 | BCCG | 4.26297 | 1.0233 | 0.05032 |  | 1.0233 | 0.9240 |
| FEV1/FVC | Boys | 5.75 | 81 | BCCG | 4.26297 | 1.0200 | 0.05032 |  | 1.0200 | 0.9210 |
| FEV1/FVC | Boys | 5.75 | 82 | BCCG | 4.26297 | 1.0167 | 0.05032 |  | 1.0167 | 0.9181 |
| FEV1/FVC | Boys | 5.75 | 83 | BCCG | 4.26297 | 1.0135 | 0.05032 |  | 1.0135 | 0.9152 |
| FEV1/FVC | Boys | 5.75 | 84 | BCCG | 4.26297 | 1.0104 | 0.05032 |  | 1.0104 | 0.9124 |
| FEV1/FVC | Boys | 5.75 | 85 | BCCG | 4.26297 | 1.0073 | 0.05032 |  | 1.0073 | 0.9096 |
| FEV1/FVC | Boys | 5.75 | 86 | BCCG | 4.26297 | 1.0043 | 0.05032 |  | 1.0043 | 0.9068 |
| FEV1/FVC | Boys | 5.75 | 87 | BCCG | 4.26297 | 1.0013 | 0.05032 |  | 1.0013 | 0.9041 |
| FEV1/FVC | Boys | 5.75 | 88 | BCCG | 4.26297 | 0.9983 | 0.05032 |  | 0.9983 | 0.9014 |
| FEV1/FVC | Boys | 5.75 | 89 | BCCG | 4.26297 | 0.9954 | 0.05032 |  | 0.9954 | 0.8988 |
| FEV1/FVC | Boys | 5.75 | 90 | BCCG | 4.26297 | 0.9925 | 0.05032 |  | 0.9925 | 0.8962 |
| FEV1/FVC | Boys | 5.75 | 91 | BCCG | 4.26297 | 0.9897 | 0.05032 |  | 0.9897 | 0.8936 |
| FEV1/FVC | Boys | 5.75 | 92 | BCCG | 4.26297 | 0.9869 | 0.05032 |  | 0.9869 | 0.8911 |
| FEV1/FVC | Boys | 5.75 | 93 | BCCG | 4.26297 | 0.9841 | 0.05032 |  | 0.9841 | 0.8886 |
| FEV1/FVC | Boys | 5.75 | 94 | BCCG | 4.26297 | 0.9814 | 0.05032 |  | 0.9814 | 0.8862 |
| FEV1/FVC | Boys | 5.75 | 95 | BCCG | 4.26297 | 0.9787 | 0.05032 |  | 0.9787 | 0.8837 |
| FEV1/FVC | Boys | 5.75 | 96 | BCCG | 4.26297 | 0.9760 | 0.05032 |  | 0.9760 | 0.8813 |
| FEV1/FVC | Boys | 5.75 | 97 | BCCG | 4.26297 | 0.9734 | 0.05032 |  | 0.9734 | 0.8790 |
| FEV1/FVC | Boys | 5.75 | 98 | BCCG | 4.26297 | 0.9708 | 0.05032 |  | 0.9708 | 0.8766 |
| FEV1/FVC | Boys | 5.75 | 99 | BCCG | 4.26297 | 0.9683 | 0.05032 |  | 0.9683 | 0.8743 |
| FEV1/FVC | Boys | 5.75 | 100 | BCCG | 4.26297 | 0.9658 | 0.05032 |  | 0.9658 | 0.8721 |
| FEV1/FVC | Boys | 5.75 | 101 | BCCG | 4.26297 | 0.9633 | 0.05032 |  | 0.9633 | 0.8698 |
| FEV1/FVC | Boys | 5.75 | 102 | BCCG | 4.26297 | 0.9608 | 0.05032 |  | 0.9608 | 0.8676 |
| FEV1/FVC | Boys | 5.75 | 103 | BCCG | 4.26297 | 0.9584 | 0.05032 |  | 0.9584 | 0.8654 |
| FEV1/FVC | Boys | 5.75 | 104 | BCCG | 4.26297 | 0.9560 | 0.05032 |  | 0.9560 | 0.8632 |
| FEV1/FVC | Boys | 5.75 | 105 | BCCG | 4.26297 | 0.9536 | 0.05032 |  | 0.9536 | 0.8611 |
| FEV1/FVC | Boys | 5.75 | 106 | BCCG | 4.26297 | 0.9513 | 0.05032 |  | 0.9513 | 0.8590 |
| FEV1/FVC | Boys | 5.75 | 107 | BCCG | 4.26297 | 0.9490 | 0.05032 |  | 0.9490 | 0.8569 |
| FEV1/FVC | Boys | 5.75 | 108 | BCCG | 4.26297 | 0.9467 | 0.05032 |  | 0.9467 | 0.8548 |
| FEV1/FVC | Boys | 5.75 | 109 | BCCG | 4.26297 | 0.9444 | 0.05032 |  | 0.9444 | 0.8528 |
| FEV1/FVC | Boys | 5.75 | 110 | BCCG | 4.26297 | 0.9422 | 0.05032 |  | 0.9422 | 0.8508 |
| FEV1/FVC | Boys | 5.75 | 111 | BCCG | 4.26297 | 0.9400 | 0.05032 |  | 0.9400 | 0.8488 |
| FEV1/FVC | Boys | 5.75 | 112 | BCCG | 4.26297 | 0.9378 | 0.05032 |  | 0.9378 | 0.8468 |
| FEV1/FVC | Boys | 5.75 | 113 | BCCG | 4.26297 | 0.9357 | 0.05032 |  | 0.9357 | 0.8449 |
| FEV1/FVC | Boys | 5.75 | 114 | BCCG | 4.26297 | 0.9335 | 0.05032 |  | 0.9335 | 0.8430 |
| FEV1/FVC | Boys | 5.75 | 115 | BCCG | 4.26297 | 0.9314 | 0.05032 |  | 0.9314 | 0.8410 |
| FEV1/FVC | Boys | 5.75 | 116 | BCCG | 4.26297 | 0.9293 | 0.05032 |  | 0.9293 | 0.8392 |
| FEV1/FVC | Boys | 5.75 | 117 | BCCG | 4.26297 | 0.9273 | 0.05032 |  | 0.9273 | 0.8373 |
| FEV1/FVC | Boys | 5.75 | 118 | BCCG | 4.26297 | 0.9252 | 0.05032 |  | 0.9252 | 0.8355 |
| FEV1/FVC | Boys | 5.75 | 119 | BCCG | 4.26297 | 0.9232 | 0.05032 |  | 0.9232 | 0.8336 |
| FEV1/FVC | Boys | 5.75 | 120 | BCCG | 4.26297 | 0.9212 | 0.05032 |  | 0.9212 | 0.8318 |
| FEV1/FVC | Boys | 5.75 | 121 | BCCG | 4.26297 | 0.9192 | 0.05032 |  | 0.9192 | 0.8300 |
| FEV1/FVC | Boys | 5.75 | 122 | BCCG | 4.26297 | 0.9173 | 0.05032 |  | 0.9173 | 0.8283 |
| FEV1/FVC | Boys | 5.75 | 123 | BCCG | 4.26297 | 0.9153 | 0.05032 |  | 0.9153 | 0.8265 |
| FEV1/FVC | Boys | 5.75 | 124 | BCCG | 4.26297 | 0.9134 | 0.05032 |  | 0.9134 | 0.8248 |
| FEV1/FVC | Boys | 5.75 | 125 | BCCG | 4.26297 | 0.9115 | 0.05032 |  | 0.9115 | 0.8231 |
| FEV1/FVC | Boys | 5.75 | 126 | BCCG | 4.26297 | 0.9096 | 0.05032 |  | 0.9096 | 0.8214 |
| FEV1/FVC | Boys | 5.75 | 127 | BCCG | 4.26297 | 0.9078 | 0.05032 |  | 0.9078 | 0.8197 |
| FEV1/FVC | Boys | 5.75 | 128 | BCCG | 4.26297 | 0.9059 | 0.05032 |  | 0.9059 | 0.8180 |
| FEV1/FVC | Boys | 5.75 | 129 | BCCG | 4.26297 | 0.9041 | 0.05032 |  | 0.9041 | 0.8164 |
| FEV1/FVC | Boys | 5.75 | 130 | BCCG | 4.26297 | 0.9023 | 0.05032 |  | 0.9023 | 0.8147 |
| FEV1/FVC | Boys | 5.75 | 131 | BCCG | 4.26297 | 0.9005 | 0.05032 |  | 0.9005 | 0.8131 |
| FEV1/FVC | Boys | 5.75 | 132 | BCCG | 4.26297 | 0.8987 | 0.05032 |  | 0.8987 | 0.8115 |
| FEV1/FVC | Boys | 6.00 | 79 | BCCG | 4.26297 | 1.0293 | 0.05032 |  | 1.0293 | 0.9294 |
| FEV1/FVC | Boys | 6.00 | 80 | BCCG | 4.26297 | 1.0259 | 0.05032 |  | 1.0259 | 0.9264 |
| FEV1/FVC | Boys | 6.00 | 81 | BCCG | 4.26297 | 1.0226 | 0.05032 |  | 1.0226 | 0.9234 |
| FEV1/FVC | Boys | 6.00 | 82 | BCCG | 4.26297 | 1.0194 | 0.05032 |  | 1.0194 | 0.9204 |
| FEV1/FVC | Boys | 6.00 | 83 | BCCG | 4.26297 | 1.0162 | 0.05032 |  | 1.0162 | 0.9176 |
| FEV1/FVC | Boys | 6.00 | 84 | BCCG | 4.26297 | 1.0130 | 0.05032 |  | 1.0130 | 0.9147 |
| FEV1/FVC | Boys | 6.00 | 85 | BCCG | 4.26297 | 1.0099 | 0.05032 |  | 1.0099 | 0.9119 |
| FEV1/FVC | Boys | 6.00 | 86 | BCCG | 4.26297 | 1.0069 | 0.05032 |  | 1.0069 | 0.9092 |
| FEV1/FVC | Boys | 6.00 | 87 | BCCG | 4.26297 | 1.0038 | 0.05032 |  | 1.0038 | 0.9064 |
| FEV1/FVC | Boys | 6.00 | 88 | BCCG | 4.26297 | 1.0009 | 0.05032 |  | 1.0009 | 0.9038 |
| FEV1/FVC | Boys | 6.00 | 89 | BCCG | 4.26297 | 0.9980 | 0.05032 |  | 0.9980 | 0.9011 |
| FEV1/FVC | Boys | 6.00 | 90 | BCCG | 4.26297 | 0.9951 | 0.05032 |  | 0.9951 | 0.8985 |
| FEV1/FVC | Boys | 6.00 | 91 | BCCG | 4.26297 | 0.9922 | 0.05032 |  | 0.9922 | 0.8959 |
| FEV1/FVC | Boys | 6.00 | 92 | BCCG | 4.26297 | 0.9894 | 0.05032 |  | 0.9894 | 0.8934 |
| FEV1/FVC | Boys | 6.00 | 93 | BCCG | 4.26297 | 0.9866 | 0.05032 |  | 0.9866 | 0.8909 |
| FEV1/FVC | Boys | 6.00 | 94 | BCCG | 4.26297 | 0.9839 | 0.05032 |  | 0.9839 | 0.8884 |
| FEV1/FVC | Boys | 6.00 | 95 | BCCG | 4.26297 | 0.9812 | 0.05032 |  | 0.9812 | 0.8860 |
| FEV1/FVC | Boys | 6.00 | 96 | BCCG | 4.26297 | 0.9786 | 0.05032 |  | 0.9786 | 0.8836 |
| FEV1/FVC | Boys | 6.00 | 97 | BCCG | 4.26297 | 0.9759 | 0.05032 |  | 0.9759 | 0.8812 |
| FEV1/FVC | Boys | 6.00 | 98 | BCCG | 4.26297 | 0.9733 | 0.05032 |  | 0.9733 | 0.8789 |
| FEV1/FVC | Boys | 6.00 | 99 | BCCG | 4.26297 | 0.9708 | 0.05032 |  | 0.9708 | 0.8766 |
| FEV1/FVC | Boys | 6.00 | 100 | BCCG | 4.26297 | 0.9683 | 0.05032 |  | 0.9683 | 0.8743 |
| FEV1/FVC | Boys | 6.00 | 101 | BCCG | 4.26297 | 0.9658 | 0.05032 |  | 0.9658 | 0.8721 |
| FEV1/FVC | Boys | 6.00 | 102 | BCCG | 4.26297 | 0.9633 | 0.05032 |  | 0.9633 | 0.8698 |
| FEV1/FVC | Boys | 6.00 | 103 | BCCG | 4.26297 | 0.9609 | 0.05032 |  | 0.9609 | 0.8676 |
| FEV1/FVC | Boys | 6.00 | 104 | BCCG | 4.26297 | 0.9585 | 0.05032 |  | 0.9585 | 0.8655 |
| FEV1/FVC | Boys | 6.00 | 105 | BCCG | 4.26297 | 0.9561 | 0.05032 |  | 0.9561 | 0.8633 |
| FEV1/FVC | Boys | 6.00 | 106 | BCCG | 4.26297 | 0.9538 | 0.05032 |  | 0.9538 | 0.8612 |
| FEV1/FVC | Boys | 6.00 | 107 | BCCG | 4.26297 | 0.9514 | 0.05032 |  | 0.9514 | 0.8591 |
| FEV1/FVC | Boys | 6.00 | 108 | BCCG | 4.26297 | 0.9491 | 0.05032 |  | 0.9491 | 0.8571 |
| FEV1/FVC | Boys | 6.00 | 109 | BCCG | 4.26297 | 0.9469 | 0.05032 |  | 0.9469 | 0.8550 |
| FEV1/FVC | Boys | 6.00 | 110 | BCCG | 4.26297 | 0.9446 | 0.05032 |  | 0.9446 | 0.8530 |
| FEV1/FVC | Boys | 6.00 | 111 | BCCG | 4.26297 | 0.9424 | 0.05032 |  | 0.9424 | 0.8510 |
| FEV1/FVC | Boys | 6.00 | 112 | BCCG | 4.26297 | 0.9402 | 0.05032 |  | 0.9402 | 0.8490 |
| FEV1/FVC | Boys | 6.00 | 113 | BCCG | 4.26297 | 0.9381 | 0.05032 |  | 0.9381 | 0.8471 |
| FEV1/FVC | Boys | 6.00 | 114 | BCCG | 4.26297 | 0.9359 | 0.05032 |  | 0.9359 | 0.8451 |
| FEV1/FVC | Boys | 6.00 | 115 | BCCG | 4.26297 | 0.9338 | 0.05032 |  | 0.9338 | 0.8432 |
| FEV1/FVC | Boys | 6.00 | 116 | BCCG | 4.26297 | 0.9317 | 0.05032 |  | 0.9317 | 0.8413 |
| FEV1/FVC | Boys | 6.00 | 117 | BCCG | 4.26297 | 0.9297 | 0.05032 |  | 0.9297 | 0.8395 |
| FEV1/FVC | Boys | 6.00 | 118 | BCCG | 4.26297 | 0.9276 | 0.05032 |  | 0.9276 | 0.8376 |
| FEV1/FVC | Boys | 6.00 | 119 | BCCG | 4.26297 | 0.9256 | 0.05032 |  | 0.9256 | 0.8358 |
| FEV1/FVC | Boys | 6.00 | 120 | BCCG | 4.26297 | 0.9236 | 0.05032 |  | 0.9236 | 0.8340 |
| FEV1/FVC | Boys | 6.00 | 121 | BCCG | 4.26297 | 0.9216 | 0.05032 |  | 0.9216 | 0.8322 |
| FEV1/FVC | Boys | 6.00 | 122 | BCCG | 4.26297 | 0.9196 | 0.05032 |  | 0.9196 | 0.8304 |
| FEV1/FVC | Boys | 6.00 | 123 | BCCG | 4.26297 | 0.9177 | 0.05032 |  | 0.9177 | 0.8286 |
| FEV1/FVC | Boys | 6.00 | 124 | BCCG | 4.26297 | 0.9158 | 0.05032 |  | 0.9158 | 0.8269 |
| FEV1/FVC | Boys | 6.00 | 125 | BCCG | 4.26297 | 0.9139 | 0.05032 |  | 0.9139 | 0.8252 |
| FEV1/FVC | Boys | 6.00 | 126 | BCCG | 4.26297 | 0.9120 | 0.05032 |  | 0.9120 | 0.8235 |
| FEV1/FVC | Boys | 6.00 | 127 | BCCG | 4.26297 | 0.9101 | 0.05032 |  | 0.9101 | 0.8218 |
| FEV1/FVC | Boys | 6.00 | 128 | BCCG | 4.26297 | 0.9083 | 0.05032 |  | 0.9083 | 0.8201 |
| FEV1/FVC | Boys | 6.00 | 129 | BCCG | 4.26297 | 0.9064 | 0.05032 |  | 0.9064 | 0.8185 |
| FEV1/FVC | Boys | 6.00 | 130 | BCCG | 4.26297 | 0.9046 | 0.05032 |  | 0.9046 | 0.8168 |
| FEV1/FVC | Boys | 6.00 | 131 | BCCG | 4.26297 | 0.9028 | 0.05032 |  | 0.9028 | 0.8152 |
| FEV1/FVC | Boys | 6.00 | 132 | BCCG | 4.26297 | 0.9011 | 0.05032 |  | 0.9011 | 0.8136 |
| FEV1/FVC | Girls | 3.00 | 88 | NO |  | 0.9346 | 0.06449 |  | 0.9346 | 0.8286 |
| FEV1/FVC | Girls | 3.00 | 89 | NO |  | 0.9344 | 0.06449 |  | 0.9344 | 0.8283 |
| FEV1/FVC | Girls | 3.00 | 90 | NO |  | 0.9341 | 0.06449 |  | 0.9341 | 0.8280 |
| FEV1/FVC | Girls | 3.00 | 91 | NO |  | 0.9338 | 0.06449 |  | 0.9338 | 0.8277 |
| FEV1/FVC | Girls | 3.00 | 92 | NO |  | 0.9335 | 0.06449 |  | 0.9335 | 0.8275 |
| FEV1/FVC | Girls | 3.00 | 93 | NO |  | 0.9333 | 0.06449 |  | 0.9333 | 0.8272 |
| FEV1/FVC | Girls | 3.00 | 94 | NO |  | 0.9330 | 0.06449 |  | 0.9330 | 0.8269 |
| FEV1/FVC | Girls | 3.00 | 95 | NO |  | 0.9327 | 0.06449 |  | 0.9327 | 0.8267 |
| FEV1/FVC | Girls | 3.00 | 96 | NO |  | 0.9325 | 0.06449 |  | 0.9325 | 0.8264 |
| FEV1/FVC | Girls | 3.00 | 97 | NO |  | 0.9322 | 0.06449 |  | 0.9322 | 0.8262 |
| FEV1/FVC | Girls | 3.00 | 98 | NO |  | 0.9320 | 0.06449 |  | 0.9320 | 0.8259 |
| FEV1/FVC | Girls | 3.00 | 99 | NO |  | 0.9317 | 0.06449 |  | 0.9317 | 0.8257 |
| FEV1/FVC | Girls | 3.00 | 100 | NO |  | 0.9315 | 0.06449 |  | 0.9315 | 0.8254 |
| FEV1/FVC | Girls | 3.00 | 101 | NO |  | 0.9312 | 0.06449 |  | 0.9312 | 0.8252 |
| FEV1/FVC | Girls | 3.00 | 102 | NO |  | 0.9310 | 0.06449 |  | 0.9310 | 0.8249 |
| FEV1/FVC | Girls | 3.00 | 103 | NO |  | 0.9307 | 0.06449 |  | 0.9307 | 0.8247 |
| FEV1/FVC | Girls | 3.00 | 104 | NO |  | 0.9305 | 0.06449 |  | 0.9305 | 0.8244 |
| FEV1/FVC | Girls | 3.00 | 105 | NO |  | 0.9303 | 0.06449 |  | 0.9303 | 0.8242 |
| FEV1/FVC | Girls | 3.00 | 106 | NO |  | 0.9300 | 0.06449 |  | 0.9300 | 0.8240 |
| FEV1/FVC | Girls | 3.00 | 107 | NO |  | 0.9298 | 0.06449 |  | 0.9298 | 0.8237 |
| FEV1/FVC | Girls | 3.00 | 108 | NO |  | 0.9296 | 0.06449 |  | 0.9296 | 0.8235 |
| FEV1/FVC | Girls | 3.00 | 109 | NO |  | 0.9294 | 0.06449 |  | 0.9294 | 0.8233 |
| FEV1/FVC | Girls | 3.00 | 110 | NO |  | 0.9291 | 0.06449 |  | 0.9291 | 0.8231 |
| FEV1/FVC | Girls | 3.00 | 111 | NO |  | 0.9289 | 0.06449 |  | 0.9289 | 0.8228 |
| FEV1/FVC | Girls | 3.00 | 112 | NO |  | 0.9287 | 0.06449 |  | 0.9287 | 0.8226 |
| FEV1/FVC | Girls | 3.00 | 113 | NO |  | 0.9285 | 0.06449 |  | 0.9285 | 0.8224 |
| FEV1/FVC | Girls | 3.00 | 114 | NO |  | 0.9282 | 0.06449 |  | 0.9282 | 0.8222 |
| FEV1/FVC | Girls | 3.00 | 115 | NO |  | 0.9280 | 0.06449 |  | 0.9280 | 0.8220 |
| FEV1/FVC | Girls | 3.00 | 116 | NO |  | 0.9278 | 0.06449 |  | 0.9278 | 0.8217 |
| FEV1/FVC | Girls | 3.00 | 117 | NO |  | 0.9276 | 0.06449 |  | 0.9276 | 0.8215 |
| FEV1/FVC | Girls | 3.00 | 118 | NO |  | 0.9274 | 0.06449 |  | 0.9274 | 0.8213 |
| FEV1/FVC | Girls | 3.00 | 119 | NO |  | 0.9272 | 0.06449 |  | 0.9272 | 0.8211 |
| FEV1/FVC | Girls | 3.00 | 120 | NO |  | 0.9270 | 0.06449 |  | 0.9270 | 0.8209 |
| FEV1/FVC | Girls | 3.00 | 121 | NO |  | 0.9268 | 0.06449 |  | 0.9268 | 0.8207 |
| FEV1/FVC | Girls | 3.00 | 122 | NO |  | 0.9266 | 0.06449 |  | 0.9266 | 0.8205 |
| FEV1/FVC | Girls | 3.00 | 123 | NO |  | 0.9264 | 0.06449 |  | 0.9264 | 0.8203 |
| FEV1/FVC | Girls | 3.00 | 124 | NO |  | 0.9262 | 0.06449 |  | 0.9262 | 0.8201 |
| FEV1/FVC | Girls | 3.25 | 88 | NO |  | 0.9353 | 0.06062 |  | 0.9353 | 0.8356 |
| FEV1/FVC | Girls | 3.25 | 89 | NO |  | 0.9350 | 0.06062 |  | 0.9350 | 0.8353 |
| FEV1/FVC | Girls | 3.25 | 90 | NO |  | 0.9348 | 0.06062 |  | 0.9348 | 0.8350 |
| FEV1/FVC | Girls | 3.25 | 91 | NO |  | 0.9345 | 0.06062 |  | 0.9345 | 0.8348 |
| FEV1/FVC | Girls | 3.25 | 92 | NO |  | 0.9342 | 0.06062 |  | 0.9342 | 0.8345 |
| FEV1/FVC | Girls | 3.25 | 93 | NO |  | 0.9339 | 0.06062 |  | 0.9339 | 0.8342 |
| FEV1/FVC | Girls | 3.25 | 94 | NO |  | 0.9337 | 0.06062 |  | 0.9337 | 0.8340 |
| FEV1/FVC | Girls | 3.25 | 95 | NO |  | 0.9334 | 0.06062 |  | 0.9334 | 0.8337 |
| FEV1/FVC | Girls | 3.25 | 96 | NO |  | 0.9332 | 0.06062 |  | 0.9332 | 0.8334 |
| FEV1/FVC | Girls | 3.25 | 97 | NO |  | 0.9329 | 0.06062 |  | 0.9329 | 0.8332 |
| FEV1/FVC | Girls | 3.25 | 98 | NO |  | 0.9326 | 0.06062 |  | 0.9326 | 0.8329 |
| FEV1/FVC | Girls | 3.25 | 99 | NO |  | 0.9324 | 0.06062 |  | 0.9324 | 0.8327 |
| FEV1/FVC | Girls | 3.25 | 100 | NO |  | 0.9321 | 0.06062 |  | 0.9321 | 0.8324 |
| FEV1/FVC | Girls | 3.25 | 101 | NO |  | 0.9319 | 0.06062 |  | 0.9319 | 0.8322 |
| FEV1/FVC | Girls | 3.25 | 102 | NO |  | 0.9317 | 0.06062 |  | 0.9317 | 0.8319 |
| FEV1/FVC | Girls | 3.25 | 103 | NO |  | 0.9314 | 0.06062 |  | 0.9314 | 0.8317 |
| FEV1/FVC | Girls | 3.25 | 104 | NO |  | 0.9312 | 0.06062 |  | 0.9312 | 0.8315 |
| FEV1/FVC | Girls | 3.25 | 105 | NO |  | 0.9309 | 0.06062 |  | 0.9309 | 0.8312 |
| FEV1/FVC | Girls | 3.25 | 106 | NO |  | 0.9307 | 0.06062 |  | 0.9307 | 0.8310 |
| FEV1/FVC | Girls | 3.25 | 107 | NO |  | 0.9305 | 0.06062 |  | 0.9305 | 0.8308 |
| FEV1/FVC | Girls | 3.25 | 108 | NO |  | 0.9302 | 0.06062 |  | 0.9302 | 0.8305 |
| FEV1/FVC | Girls | 3.25 | 109 | NO |  | 0.9300 | 0.06062 |  | 0.9300 | 0.8303 |
| FEV1/FVC | Girls | 3.25 | 110 | NO |  | 0.9298 | 0.06062 |  | 0.9298 | 0.8301 |
| FEV1/FVC | Girls | 3.25 | 111 | NO |  | 0.9296 | 0.06062 |  | 0.9296 | 0.8298 |
| FEV1/FVC | Girls | 3.25 | 112 | NO |  | 0.9293 | 0.06062 |  | 0.9293 | 0.8296 |
| FEV1/FVC | Girls | 3.25 | 113 | NO |  | 0.9291 | 0.06062 |  | 0.9291 | 0.8294 |
| FEV1/FVC | Girls | 3.25 | 114 | NO |  | 0.9289 | 0.06062 |  | 0.9289 | 0.8292 |
| FEV1/FVC | Girls | 3.25 | 115 | NO |  | 0.9287 | 0.06062 |  | 0.9287 | 0.8290 |
| FEV1/FVC | Girls | 3.25 | 116 | NO |  | 0.9285 | 0.06062 |  | 0.9285 | 0.8288 |
| FEV1/FVC | Girls | 3.25 | 117 | NO |  | 0.9283 | 0.06062 |  | 0.9283 | 0.8285 |
| FEV1/FVC | Girls | 3.25 | 118 | NO |  | 0.9281 | 0.06062 |  | 0.9281 | 0.8283 |
| FEV1/FVC | Girls | 3.25 | 119 | NO |  | 0.9278 | 0.06062 |  | 0.9278 | 0.8281 |
| FEV1/FVC | Girls | 3.25 | 120 | NO |  | 0.9276 | 0.06062 |  | 0.9276 | 0.8279 |
| FEV1/FVC | Girls | 3.25 | 121 | NO |  | 0.9274 | 0.06062 |  | 0.9274 | 0.8277 |
| FEV1/FVC | Girls | 3.25 | 122 | NO |  | 0.9272 | 0.06062 |  | 0.9272 | 0.8275 |
| FEV1/FVC | Girls | 3.25 | 123 | NO |  | 0.9270 | 0.06062 |  | 0.9270 | 0.8273 |
| FEV1/FVC | Girls | 3.25 | 124 | NO |  | 0.9268 | 0.06062 |  | 0.9268 | 0.8271 |
| FEV1/FVC | Girls | 3.50 | 88 | NO |  | 0.9359 | 0.05724 |  | 0.9359 | 0.8418 |
| FEV1/FVC | Girls | 3.50 | 89 | NO |  | 0.9356 | 0.05724 |  | 0.9356 | 0.8415 |
| FEV1/FVC | Girls | 3.50 | 90 | NO |  | 0.9353 | 0.05724 |  | 0.9353 | 0.8412 |
| FEV1/FVC | Girls | 3.50 | 91 | NO |  | 0.9351 | 0.05724 |  | 0.9351 | 0.8409 |
| FEV1/FVC | Girls | 3.50 | 92 | NO |  | 0.9348 | 0.05724 |  | 0.9348 | 0.8407 |
| FEV1/FVC | Girls | 3.50 | 93 | NO |  | 0.9345 | 0.05724 |  | 0.9345 | 0.8404 |
| FEV1/FVC | Girls | 3.50 | 94 | NO |  | 0.9343 | 0.05724 |  | 0.9343 | 0.8401 |
| FEV1/FVC | Girls | 3.50 | 95 | NO |  | 0.9340 | 0.05724 |  | 0.9340 | 0.8399 |
| FEV1/FVC | Girls | 3.50 | 96 | NO |  | 0.9337 | 0.05724 |  | 0.9337 | 0.8396 |
| FEV1/FVC | Girls | 3.50 | 97 | NO |  | 0.9335 | 0.05724 |  | 0.9335 | 0.8393 |
| FEV1/FVC | Girls | 3.50 | 98 | NO |  | 0.9332 | 0.05724 |  | 0.9332 | 0.8391 |
| FEV1/FVC | Girls | 3.50 | 99 | NO |  | 0.9330 | 0.05724 |  | 0.9330 | 0.8388 |
| FEV1/FVC | Girls | 3.50 | 100 | NO |  | 0.9327 | 0.05724 |  | 0.9327 | 0.8386 |
| FEV1/FVC | Girls | 3.50 | 101 | NO |  | 0.9325 | 0.05724 |  | 0.9325 | 0.8383 |
| FEV1/FVC | Girls | 3.50 | 102 | NO |  | 0.9322 | 0.05724 |  | 0.9322 | 0.8381 |
| FEV1/FVC | Girls | 3.50 | 103 | NO |  | 0.9320 | 0.05724 |  | 0.9320 | 0.8379 |
| FEV1/FVC | Girls | 3.50 | 104 | NO |  | 0.9318 | 0.05724 |  | 0.9318 | 0.8376 |
| FEV1/FVC | Girls | 3.50 | 105 | NO |  | 0.9315 | 0.05724 |  | 0.9315 | 0.8374 |
| FEV1/FVC | Girls | 3.50 | 106 | NO |  | 0.9313 | 0.05724 |  | 0.9313 | 0.8371 |
| FEV1/FVC | Girls | 3.50 | 107 | NO |  | 0.9311 | 0.05724 |  | 0.9311 | 0.8369 |
| FEV1/FVC | Girls | 3.50 | 108 | NO |  | 0.9308 | 0.05724 |  | 0.9308 | 0.8367 |
| FEV1/FVC | Girls | 3.50 | 109 | NO |  | 0.9306 | 0.05724 |  | 0.9306 | 0.8365 |
| FEV1/FVC | Girls | 3.50 | 110 | NO |  | 0.9304 | 0.05724 |  | 0.9304 | 0.8362 |
| FEV1/FVC | Girls | 3.50 | 111 | NO |  | 0.9302 | 0.05724 |  | 0.9302 | 0.8360 |
| FEV1/FVC | Girls | 3.50 | 112 | NO |  | 0.9299 | 0.05724 |  | 0.9299 | 0.8358 |
| FEV1/FVC | Girls | 3.50 | 113 | NO |  | 0.9297 | 0.05724 |  | 0.9297 | 0.8356 |
| FEV1/FVC | Girls | 3.50 | 114 | NO |  | 0.9295 | 0.05724 |  | 0.9295 | 0.8354 |
| FEV1/FVC | Girls | 3.50 | 115 | NO |  | 0.9293 | 0.05724 |  | 0.9293 | 0.8351 |
| FEV1/FVC | Girls | 3.50 | 116 | NO |  | 0.9291 | 0.05724 |  | 0.9291 | 0.8349 |
| FEV1/FVC | Girls | 3.50 | 117 | NO |  | 0.9289 | 0.05724 |  | 0.9289 | 0.8347 |
| FEV1/FVC | Girls | 3.50 | 118 | NO |  | 0.9286 | 0.05724 |  | 0.9286 | 0.8345 |
| FEV1/FVC | Girls | 3.50 | 119 | NO |  | 0.9284 | 0.05724 |  | 0.9284 | 0.8343 |
| FEV1/FVC | Girls | 3.50 | 120 | NO |  | 0.9282 | 0.05724 |  | 0.9282 | 0.8341 |
| FEV1/FVC | Girls | 3.50 | 121 | NO |  | 0.9280 | 0.05724 |  | 0.9280 | 0.8339 |
| FEV1/FVC | Girls | 3.50 | 122 | NO |  | 0.9278 | 0.05724 |  | 0.9278 | 0.8337 |
| FEV1/FVC | Girls | 3.50 | 123 | NO |  | 0.9276 | 0.05724 |  | 0.9276 | 0.8335 |
| FEV1/FVC | Girls | 3.50 | 124 | NO |  | 0.9274 | 0.05724 |  | 0.9274 | 0.8333 |
| FEV1/FVC | Girls | 3.75 | 88 | NO |  | 0.9364 | 0.05426 |  | 0.9364 | 0.8472 |
| FEV1/FVC | Girls | 3.75 | 89 | NO |  | 0.9361 | 0.05426 |  | 0.9361 | 0.8469 |
| FEV1/FVC | Girls | 3.75 | 90 | NO |  | 0.9358 | 0.05426 |  | 0.9358 | 0.8466 |
| FEV1/FVC | Girls | 3.75 | 91 | NO |  | 0.9356 | 0.05426 |  | 0.9356 | 0.8463 |
| FEV1/FVC | Girls | 3.75 | 92 | NO |  | 0.9353 | 0.05426 |  | 0.9353 | 0.8461 |
| FEV1/FVC | Girls | 3.75 | 93 | NO |  | 0.9350 | 0.05426 |  | 0.9350 | 0.8458 |
| FEV1/FVC | Girls | 3.75 | 94 | NO |  | 0.9348 | 0.05426 |  | 0.9348 | 0.8455 |
| FEV1/FVC | Girls | 3.75 | 95 | NO |  | 0.9345 | 0.05426 |  | 0.9345 | 0.8453 |
| FEV1/FVC | Girls | 3.75 | 96 | NO |  | 0.9342 | 0.05426 |  | 0.9342 | 0.8450 |
| FEV1/FVC | Girls | 3.75 | 97 | NO |  | 0.9340 | 0.05426 |  | 0.9340 | 0.8447 |
| FEV1/FVC | Girls | 3.75 | 98 | NO |  | 0.9337 | 0.05426 |  | 0.9337 | 0.8445 |
| FEV1/FVC | Girls | 3.75 | 99 | NO |  | 0.9335 | 0.05426 |  | 0.9335 | 0.8442 |
| FEV1/FVC | Girls | 3.75 | 100 | NO |  | 0.9332 | 0.05426 |  | 0.9332 | 0.8440 |
| FEV1/FVC | Girls | 3.75 | 101 | NO |  | 0.9330 | 0.05426 |  | 0.9330 | 0.8437 |
| FEV1/FVC | Girls | 3.75 | 102 | NO |  | 0.9327 | 0.05426 |  | 0.9327 | 0.8435 |
| FEV1/FVC | Girls | 3.75 | 103 | NO |  | 0.9325 | 0.05426 |  | 0.9325 | 0.8433 |
| FEV1/FVC | Girls | 3.75 | 104 | NO |  | 0.9323 | 0.05426 |  | 0.9323 | 0.8430 |
| FEV1/FVC | Girls | 3.75 | 105 | NO |  | 0.9320 | 0.05426 |  | 0.9320 | 0.8428 |
| FEV1/FVC | Girls | 3.75 | 106 | NO |  | 0.9318 | 0.05426 |  | 0.9318 | 0.8426 |
| FEV1/FVC | Girls | 3.75 | 107 | NO |  | 0.9316 | 0.05426 |  | 0.9316 | 0.8423 |
| FEV1/FVC | Girls | 3.75 | 108 | NO |  | 0.9313 | 0.05426 |  | 0.9313 | 0.8421 |
| FEV1/FVC | Girls | 3.75 | 109 | NO |  | 0.9311 | 0.05426 |  | 0.9311 | 0.8419 |
| FEV1/FVC | Girls | 3.75 | 110 | NO |  | 0.9309 | 0.05426 |  | 0.9309 | 0.8416 |
| FEV1/FVC | Girls | 3.75 | 111 | NO |  | 0.9307 | 0.05426 |  | 0.9307 | 0.8414 |
| FEV1/FVC | Girls | 3.75 | 112 | NO |  | 0.9304 | 0.05426 |  | 0.9304 | 0.8412 |
| FEV1/FVC | Girls | 3.75 | 113 | NO |  | 0.9302 | 0.05426 |  | 0.9302 | 0.8410 |
| FEV1/FVC | Girls | 3.75 | 114 | NO |  | 0.9300 | 0.05426 |  | 0.9300 | 0.8408 |
| FEV1/FVC | Girls | 3.75 | 115 | NO |  | 0.9298 | 0.05426 |  | 0.9298 | 0.8405 |
| FEV1/FVC | Girls | 3.75 | 116 | NO |  | 0.9296 | 0.05426 |  | 0.9296 | 0.8403 |
| FEV1/FVC | Girls | 3.75 | 117 | NO |  | 0.9294 | 0.05426 |  | 0.9294 | 0.8401 |
| FEV1/FVC | Girls | 3.75 | 118 | NO |  | 0.9291 | 0.05426 |  | 0.9291 | 0.8399 |
| FEV1/FVC | Girls | 3.75 | 119 | NO |  | 0.9289 | 0.05426 |  | 0.9289 | 0.8397 |
| FEV1/FVC | Girls | 3.75 | 120 | NO |  | 0.9287 | 0.05426 |  | 0.9287 | 0.8395 |
| FEV1/FVC | Girls | 3.75 | 121 | NO |  | 0.9285 | 0.05426 |  | 0.9285 | 0.8393 |
| FEV1/FVC | Girls | 3.75 | 122 | NO |  | 0.9283 | 0.05426 |  | 0.9283 | 0.8391 |
| FEV1/FVC | Girls | 3.75 | 123 | NO |  | 0.9281 | 0.05426 |  | 0.9281 | 0.8389 |
| FEV1/FVC | Girls | 3.75 | 124 | NO |  | 0.9279 | 0.05426 |  | 0.9279 | 0.8387 |
| FEV1/FVC | Girls | 4.00 | 88 | NO |  | 0.9368 | 0.05169 |  | 0.9368 | 0.8518 |
| FEV1/FVC | Girls | 4.00 | 89 | NO |  | 0.9365 | 0.05169 |  | 0.9365 | 0.8515 |
| FEV1/FVC | Girls | 4.00 | 90 | NO |  | 0.9362 | 0.05169 |  | 0.9362 | 0.8512 |
| FEV1/FVC | Girls | 4.00 | 91 | NO |  | 0.9359 | 0.05169 |  | 0.9359 | 0.8509 |
| FEV1/FVC | Girls | 4.00 | 92 | NO |  | 0.9357 | 0.05169 |  | 0.9357 | 0.8506 |
| FEV1/FVC | Girls | 4.00 | 93 | NO |  | 0.9354 | 0.05169 |  | 0.9354 | 0.8504 |
| FEV1/FVC | Girls | 4.00 | 94 | NO |  | 0.9351 | 0.05169 |  | 0.9351 | 0.8501 |
| FEV1/FVC | Girls | 4.00 | 95 | NO |  | 0.9349 | 0.05169 |  | 0.9349 | 0.8499 |
| FEV1/FVC | Girls | 4.00 | 96 | NO |  | 0.9346 | 0.05169 |  | 0.9346 | 0.8496 |
| FEV1/FVC | Girls | 4.00 | 97 | NO |  | 0.9344 | 0.05169 |  | 0.9344 | 0.8493 |
| FEV1/FVC | Girls | 4.00 | 98 | NO |  | 0.9341 | 0.05169 |  | 0.9341 | 0.8491 |
| FEV1/FVC | Girls | 4.00 | 99 | NO |  | 0.9339 | 0.05169 |  | 0.9339 | 0.8488 |
| FEV1/FVC | Girls | 4.00 | 100 | NO |  | 0.9336 | 0.05169 |  | 0.9336 | 0.8486 |
| FEV1/FVC | Girls | 4.00 | 101 | NO |  | 0.9334 | 0.05169 |  | 0.9334 | 0.8483 |
| FEV1/FVC | Girls | 4.00 | 102 | NO |  | 0.9331 | 0.05169 |  | 0.9331 | 0.8481 |
| FEV1/FVC | Girls | 4.00 | 103 | NO |  | 0.9329 | 0.05169 |  | 0.9329 | 0.8478 |
| FEV1/FVC | Girls | 4.00 | 104 | NO |  | 0.9326 | 0.05169 |  | 0.9326 | 0.8476 |
| FEV1/FVC | Girls | 4.00 | 105 | NO |  | 0.9324 | 0.05169 |  | 0.9324 | 0.8474 |
| FEV1/FVC | Girls | 4.00 | 106 | NO |  | 0.9322 | 0.05169 |  | 0.9322 | 0.8471 |
| FEV1/FVC | Girls | 4.00 | 107 | NO |  | 0.9319 | 0.05169 |  | 0.9319 | 0.8469 |
| FEV1/FVC | Girls | 4.00 | 108 | NO |  | 0.9317 | 0.05169 |  | 0.9317 | 0.8467 |
| FEV1/FVC | Girls | 4.00 | 109 | NO |  | 0.9315 | 0.05169 |  | 0.9315 | 0.8464 |
| FEV1/FVC | Girls | 4.00 | 110 | NO |  | 0.9312 | 0.05169 |  | 0.9312 | 0.8462 |
| FEV1/FVC | Girls | 4.00 | 111 | NO |  | 0.9310 | 0.05169 |  | 0.9310 | 0.8460 |
| FEV1/FVC | Girls | 4.00 | 112 | NO |  | 0.9308 | 0.05169 |  | 0.9308 | 0.8458 |
| FEV1/FVC | Girls | 4.00 | 113 | NO |  | 0.9306 | 0.05169 |  | 0.9306 | 0.8456 |
| FEV1/FVC | Girls | 4.00 | 114 | NO |  | 0.9304 | 0.05169 |  | 0.9304 | 0.8453 |
| FEV1/FVC | Girls | 4.00 | 115 | NO |  | 0.9301 | 0.05169 |  | 0.9301 | 0.8451 |
| FEV1/FVC | Girls | 4.00 | 116 | NO |  | 0.9299 | 0.05169 |  | 0.9299 | 0.8449 |
| FEV1/FVC | Girls | 4.00 | 117 | NO |  | 0.9297 | 0.05169 |  | 0.9297 | 0.8447 |
| FEV1/FVC | Girls | 4.00 | 118 | NO |  | 0.9295 | 0.05169 |  | 0.9295 | 0.8445 |
| FEV1/FVC | Girls | 4.00 | 119 | NO |  | 0.9293 | 0.05169 |  | 0.9293 | 0.8443 |
| FEV1/FVC | Girls | 4.00 | 120 | NO |  | 0.9291 | 0.05169 |  | 0.9291 | 0.8441 |
| FEV1/FVC | Girls | 4.00 | 121 | NO |  | 0.9289 | 0.05169 |  | 0.9289 | 0.8439 |
| FEV1/FVC | Girls | 4.00 | 122 | NO |  | 0.9287 | 0.05169 |  | 0.9287 | 0.8437 |
| FEV1/FVC | Girls | 4.00 | 123 | NO |  | 0.9285 | 0.05169 |  | 0.9285 | 0.8435 |
| FEV1/FVC | Girls | 4.00 | 124 | NO |  | 0.9283 | 0.05169 |  | 0.9283 | 0.8433 |
| FEV1/FVC | Girls | 4.25 | 88 | NO |  | 0.9370 | 0.04951 |  | 0.9370 | 0.8555 |
| FEV1/FVC | Girls | 4.25 | 89 | NO |  | 0.9367 | 0.04951 |  | 0.9367 | 0.8553 |
| FEV1/FVC | Girls | 4.25 | 90 | NO |  | 0.9364 | 0.04951 |  | 0.9364 | 0.8550 |
| FEV1/FVC | Girls | 4.25 | 91 | NO |  | 0.9361 | 0.04951 |  | 0.9361 | 0.8547 |
| FEV1/FVC | Girls | 4.25 | 92 | NO |  | 0.9359 | 0.04951 |  | 0.9359 | 0.8544 |
| FEV1/FVC | Girls | 4.25 | 93 | NO |  | 0.9356 | 0.04951 |  | 0.9356 | 0.8542 |
| FEV1/FVC | Girls | 4.25 | 94 | NO |  | 0.9353 | 0.04951 |  | 0.9353 | 0.8539 |
| FEV1/FVC | Girls | 4.25 | 95 | NO |  | 0.9351 | 0.04951 |  | 0.9351 | 0.8536 |
| FEV1/FVC | Girls | 4.25 | 96 | NO |  | 0.9348 | 0.04951 |  | 0.9348 | 0.8534 |
| FEV1/FVC | Girls | 4.25 | 97 | NO |  | 0.9346 | 0.04951 |  | 0.9346 | 0.8531 |
| FEV1/FVC | Girls | 4.25 | 98 | NO |  | 0.9343 | 0.04951 |  | 0.9343 | 0.8529 |
| FEV1/FVC | Girls | 4.25 | 99 | NO |  | 0.9341 | 0.04951 |  | 0.9341 | 0.8526 |
| FEV1/FVC | Girls | 4.25 | 100 | NO |  | 0.9338 | 0.04951 |  | 0.9338 | 0.8524 |
| FEV1/FVC | Girls | 4.25 | 101 | NO |  | 0.9336 | 0.04951 |  | 0.9336 | 0.8521 |
| FEV1/FVC | Girls | 4.25 | 102 | NO |  | 0.9333 | 0.04951 |  | 0.9333 | 0.8519 |
| FEV1/FVC | Girls | 4.25 | 103 | NO |  | 0.9331 | 0.04951 |  | 0.9331 | 0.8516 |
| FEV1/FVC | Girls | 4.25 | 104 | NO |  | 0.9328 | 0.04951 |  | 0.9328 | 0.8514 |
| FEV1/FVC | Girls | 4.25 | 105 | NO |  | 0.9326 | 0.04951 |  | 0.9326 | 0.8512 |
| FEV1/FVC | Girls | 4.25 | 106 | NO |  | 0.9324 | 0.04951 |  | 0.9324 | 0.8509 |
| FEV1/FVC | Girls | 4.25 | 107 | NO |  | 0.9321 | 0.04951 |  | 0.9321 | 0.8507 |
| FEV1/FVC | Girls | 4.25 | 108 | NO |  | 0.9319 | 0.04951 |  | 0.9319 | 0.8505 |
| FEV1/FVC | Girls | 4.25 | 109 | NO |  | 0.9317 | 0.04951 |  | 0.9317 | 0.8502 |
| FEV1/FVC | Girls | 4.25 | 110 | NO |  | 0.9314 | 0.04951 |  | 0.9314 | 0.8500 |
| FEV1/FVC | Girls | 4.25 | 111 | NO |  | 0.9312 | 0.04951 |  | 0.9312 | 0.8498 |
| FEV1/FVC | Girls | 4.25 | 112 | NO |  | 0.9310 | 0.04951 |  | 0.9310 | 0.8496 |
| FEV1/FVC | Girls | 4.25 | 113 | NO |  | 0.9308 | 0.04951 |  | 0.9308 | 0.8493 |
| FEV1/FVC | Girls | 4.25 | 114 | NO |  | 0.9306 | 0.04951 |  | 0.9306 | 0.8491 |
| FEV1/FVC | Girls | 4.25 | 115 | NO |  | 0.9303 | 0.04951 |  | 0.9303 | 0.8489 |
| FEV1/FVC | Girls | 4.25 | 116 | NO |  | 0.9301 | 0.04951 |  | 0.9301 | 0.8487 |
| FEV1/FVC | Girls | 4.25 | 117 | NO |  | 0.9299 | 0.04951 |  | 0.9299 | 0.8485 |
| FEV1/FVC | Girls | 4.25 | 118 | NO |  | 0.9297 | 0.04951 |  | 0.9297 | 0.8483 |
| FEV1/FVC | Girls | 4.25 | 119 | NO |  | 0.9295 | 0.04951 |  | 0.9295 | 0.8481 |
| FEV1/FVC | Girls | 4.25 | 120 | NO |  | 0.9293 | 0.04951 |  | 0.9293 | 0.8479 |
| FEV1/FVC | Girls | 4.25 | 121 | NO |  | 0.9291 | 0.04951 |  | 0.9291 | 0.8477 |
| FEV1/FVC | Girls | 4.25 | 122 | NO |  | 0.9289 | 0.04951 |  | 0.9289 | 0.8475 |
| FEV1/FVC | Girls | 4.25 | 123 | NO |  | 0.9287 | 0.04951 |  | 0.9287 | 0.8473 |
| FEV1/FVC | Girls | 4.25 | 124 | NO |  | 0.9285 | 0.04951 |  | 0.9285 | 0.8471 |
| FEV1/FVC | Girls | 4.50 | 88 | NO |  | 0.9370 | 0.04766 |  | 0.9370 | 0.8587 |
| FEV1/FVC | Girls | 4.50 | 89 | NO |  | 0.9368 | 0.04766 |  | 0.9368 | 0.8584 |
| FEV1/FVC | Girls | 4.50 | 90 | NO |  | 0.9365 | 0.04766 |  | 0.9365 | 0.8581 |
| FEV1/FVC | Girls | 4.50 | 91 | NO |  | 0.9362 | 0.04766 |  | 0.9362 | 0.8578 |
| FEV1/FVC | Girls | 4.50 | 92 | NO |  | 0.9359 | 0.04766 |  | 0.9359 | 0.8575 |
| FEV1/FVC | Girls | 4.50 | 93 | NO |  | 0.9357 | 0.04766 |  | 0.9357 | 0.8573 |
| FEV1/FVC | Girls | 4.50 | 94 | NO |  | 0.9354 | 0.04766 |  | 0.9354 | 0.8570 |
| FEV1/FVC | Girls | 4.50 | 95 | NO |  | 0.9351 | 0.04766 |  | 0.9351 | 0.8568 |
| FEV1/FVC | Girls | 4.50 | 96 | NO |  | 0.9349 | 0.04766 |  | 0.9349 | 0.8565 |
| FEV1/FVC | Girls | 4.50 | 97 | NO |  | 0.9346 | 0.04766 |  | 0.9346 | 0.8562 |
| FEV1/FVC | Girls | 4.50 | 98 | NO |  | 0.9344 | 0.04766 |  | 0.9344 | 0.8560 |
| FEV1/FVC | Girls | 4.50 | 99 | NO |  | 0.9341 | 0.04766 |  | 0.9341 | 0.8557 |
| FEV1/FVC | Girls | 4.50 | 100 | NO |  | 0.9339 | 0.04766 |  | 0.9339 | 0.8555 |
| FEV1/FVC | Girls | 4.50 | 101 | NO |  | 0.9336 | 0.04766 |  | 0.9336 | 0.8552 |
| FEV1/FVC | Girls | 4.50 | 102 | NO |  | 0.9334 | 0.04766 |  | 0.9334 | 0.8550 |
| FEV1/FVC | Girls | 4.50 | 103 | NO |  | 0.9331 | 0.04766 |  | 0.9331 | 0.8547 |
| FEV1/FVC | Girls | 4.50 | 104 | NO |  | 0.9329 | 0.04766 |  | 0.9329 | 0.8545 |
| FEV1/FVC | Girls | 4.50 | 105 | NO |  | 0.9327 | 0.04766 |  | 0.9327 | 0.8543 |
| FEV1/FVC | Girls | 4.50 | 106 | NO |  | 0.9324 | 0.04766 |  | 0.9324 | 0.8540 |
| FEV1/FVC | Girls | 4.50 | 107 | NO |  | 0.9322 | 0.04766 |  | 0.9322 | 0.8538 |
| FEV1/FVC | Girls | 4.50 | 108 | NO |  | 0.9320 | 0.04766 |  | 0.9320 | 0.8536 |
| FEV1/FVC | Girls | 4.50 | 109 | NO |  | 0.9317 | 0.04766 |  | 0.9317 | 0.8533 |
| FEV1/FVC | Girls | 4.50 | 110 | NO |  | 0.9315 | 0.04766 |  | 0.9315 | 0.8531 |
| FEV1/FVC | Girls | 4.50 | 111 | NO |  | 0.9313 | 0.04766 |  | 0.9313 | 0.8529 |
| FEV1/FVC | Girls | 4.50 | 112 | NO |  | 0.9311 | 0.04766 |  | 0.9311 | 0.8527 |
| FEV1/FVC | Girls | 4.50 | 113 | NO |  | 0.9308 | 0.04766 |  | 0.9308 | 0.8525 |
| FEV1/FVC | Girls | 4.50 | 114 | NO |  | 0.9306 | 0.04766 |  | 0.9306 | 0.8522 |
| FEV1/FVC | Girls | 4.50 | 115 | NO |  | 0.9304 | 0.04766 |  | 0.9304 | 0.8520 |
| FEV1/FVC | Girls | 4.50 | 116 | NO |  | 0.9302 | 0.04766 |  | 0.9302 | 0.8518 |
| FEV1/FVC | Girls | 4.50 | 117 | NO |  | 0.9300 | 0.04766 |  | 0.9300 | 0.8516 |
| FEV1/FVC | Girls | 4.50 | 118 | NO |  | 0.9298 | 0.04766 |  | 0.9298 | 0.8514 |
| FEV1/FVC | Girls | 4.50 | 119 | NO |  | 0.9296 | 0.04766 |  | 0.9296 | 0.8512 |
| FEV1/FVC | Girls | 4.50 | 120 | NO |  | 0.9294 | 0.04766 |  | 0.9294 | 0.8510 |
| FEV1/FVC | Girls | 4.50 | 121 | NO |  | 0.9292 | 0.04766 |  | 0.9292 | 0.8508 |
| FEV1/FVC | Girls | 4.50 | 122 | NO |  | 0.9290 | 0.04766 |  | 0.9290 | 0.8506 |
| FEV1/FVC | Girls | 4.50 | 123 | NO |  | 0.9288 | 0.04766 |  | 0.9288 | 0.8504 |
| FEV1/FVC | Girls | 4.50 | 124 | NO |  | 0.9286 | 0.04766 |  | 0.9286 | 0.8502 |
| FEV1/FVC | Girls | 4.75 | 88 | NO |  | 0.9370 | 0.04605 |  | 0.9370 | 0.8613 |
| FEV1/FVC | Girls | 4.75 | 89 | NO |  | 0.9367 | 0.04605 |  | 0.9367 | 0.8610 |
| FEV1/FVC | Girls | 4.75 | 90 | NO |  | 0.9365 | 0.04605 |  | 0.9365 | 0.8607 |
| FEV1/FVC | Girls | 4.75 | 91 | NO |  | 0.9362 | 0.04605 |  | 0.9362 | 0.8604 |
| FEV1/FVC | Girls | 4.75 | 92 | NO |  | 0.9359 | 0.04605 |  | 0.9359 | 0.8602 |
| FEV1/FVC | Girls | 4.75 | 93 | NO |  | 0.9356 | 0.04605 |  | 0.9356 | 0.8599 |
| FEV1/FVC | Girls | 4.75 | 94 | NO |  | 0.9354 | 0.04605 |  | 0.9354 | 0.8596 |
| FEV1/FVC | Girls | 4.75 | 95 | NO |  | 0.9351 | 0.04605 |  | 0.9351 | 0.8594 |
| FEV1/FVC | Girls | 4.75 | 96 | NO |  | 0.9349 | 0.04605 |  | 0.9349 | 0.8591 |
| FEV1/FVC | Girls | 4.75 | 97 | NO |  | 0.9346 | 0.04605 |  | 0.9346 | 0.8588 |
| FEV1/FVC | Girls | 4.75 | 98 | NO |  | 0.9343 | 0.04605 |  | 0.9343 | 0.8586 |
| FEV1/FVC | Girls | 4.75 | 99 | NO |  | 0.9341 | 0.04605 |  | 0.9341 | 0.8583 |
| FEV1/FVC | Girls | 4.75 | 100 | NO |  | 0.9338 | 0.04605 |  | 0.9338 | 0.8581 |
| FEV1/FVC | Girls | 4.75 | 101 | NO |  | 0.9336 | 0.04605 |  | 0.9336 | 0.8578 |
| FEV1/FVC | Girls | 4.75 | 102 | NO |  | 0.9334 | 0.04605 |  | 0.9334 | 0.8576 |
| FEV1/FVC | Girls | 4.75 | 103 | NO |  | 0.9331 | 0.04605 |  | 0.9331 | 0.8574 |
| FEV1/FVC | Girls | 4.75 | 104 | NO |  | 0.9329 | 0.04605 |  | 0.9329 | 0.8571 |
| FEV1/FVC | Girls | 4.75 | 105 | NO |  | 0.9326 | 0.04605 |  | 0.9326 | 0.8569 |
| FEV1/FVC | Girls | 4.75 | 106 | NO |  | 0.9324 | 0.04605 |  | 0.9324 | 0.8567 |
| FEV1/FVC | Girls | 4.75 | 107 | NO |  | 0.9322 | 0.04605 |  | 0.9322 | 0.8564 |
| FEV1/FVC | Girls | 4.75 | 108 | NO |  | 0.9319 | 0.04605 |  | 0.9319 | 0.8562 |
| FEV1/FVC | Girls | 4.75 | 109 | NO |  | 0.9317 | 0.04605 |  | 0.9317 | 0.8560 |
| FEV1/FVC | Girls | 4.75 | 110 | NO |  | 0.9315 | 0.04605 |  | 0.9315 | 0.8557 |
| FEV1/FVC | Girls | 4.75 | 111 | NO |  | 0.9313 | 0.04605 |  | 0.9313 | 0.8555 |
| FEV1/FVC | Girls | 4.75 | 112 | NO |  | 0.9310 | 0.04605 |  | 0.9310 | 0.8553 |
| FEV1/FVC | Girls | 4.75 | 113 | NO |  | 0.9308 | 0.04605 |  | 0.9308 | 0.8551 |
| FEV1/FVC | Girls | 4.75 | 114 | NO |  | 0.9306 | 0.04605 |  | 0.9306 | 0.8549 |
| FEV1/FVC | Girls | 4.75 | 115 | NO |  | 0.9304 | 0.04605 |  | 0.9304 | 0.8546 |
| FEV1/FVC | Girls | 4.75 | 116 | NO |  | 0.9302 | 0.04605 |  | 0.9302 | 0.8544 |
| FEV1/FVC | Girls | 4.75 | 117 | NO |  | 0.9300 | 0.04605 |  | 0.9300 | 0.8542 |
| FEV1/FVC | Girls | 4.75 | 118 | NO |  | 0.9298 | 0.04605 |  | 0.9298 | 0.8540 |
| FEV1/FVC | Girls | 4.75 | 119 | NO |  | 0.9295 | 0.04605 |  | 0.9295 | 0.8538 |
| FEV1/FVC | Girls | 4.75 | 120 | NO |  | 0.9293 | 0.04605 |  | 0.9293 | 0.8536 |
| FEV1/FVC | Girls | 4.75 | 121 | NO |  | 0.9291 | 0.04605 |  | 0.9291 | 0.8534 |
| FEV1/FVC | Girls | 4.75 | 122 | NO |  | 0.9289 | 0.04605 |  | 0.9289 | 0.8532 |
| FEV1/FVC | Girls | 4.75 | 123 | NO |  | 0.9287 | 0.04605 |  | 0.9287 | 0.8530 |
| FEV1/FVC | Girls | 4.75 | 124 | NO |  | 0.9285 | 0.04605 |  | 0.9285 | 0.8528 |
| FEV1/FVC | Girls | 5.00 | 88 | NO |  | 0.9369 | 0.04463 |  | 0.9369 | 0.8635 |
| FEV1/FVC | Girls | 5.00 | 89 | NO |  | 0.9367 | 0.04463 |  | 0.9367 | 0.8632 |
| FEV1/FVC | Girls | 5.00 | 90 | NO |  | 0.9364 | 0.04463 |  | 0.9364 | 0.8630 |
| FEV1/FVC | Girls | 5.00 | 91 | NO |  | 0.9361 | 0.04463 |  | 0.9361 | 0.8627 |
| FEV1/FVC | Girls | 5.00 | 92 | NO |  | 0.9358 | 0.04463 |  | 0.9358 | 0.8624 |
| FEV1/FVC | Girls | 5.00 | 93 | NO |  | 0.9356 | 0.04463 |  | 0.9356 | 0.8622 |
| FEV1/FVC | Girls | 5.00 | 94 | NO |  | 0.9353 | 0.04463 |  | 0.9353 | 0.8619 |
| FEV1/FVC | Girls | 5.00 | 95 | NO |  | 0.9350 | 0.04463 |  | 0.9350 | 0.8616 |
| FEV1/FVC | Girls | 5.00 | 96 | NO |  | 0.9348 | 0.04463 |  | 0.9348 | 0.8614 |
| FEV1/FVC | Girls | 5.00 | 97 | NO |  | 0.9345 | 0.04463 |  | 0.9345 | 0.8611 |
| FEV1/FVC | Girls | 5.00 | 98 | NO |  | 0.9343 | 0.04463 |  | 0.9343 | 0.8609 |
| FEV1/FVC | Girls | 5.00 | 99 | NO |  | 0.9340 | 0.04463 |  | 0.9340 | 0.8606 |
| FEV1/FVC | Girls | 5.00 | 100 | NO |  | 0.9338 | 0.04463 |  | 0.9338 | 0.8604 |
| FEV1/FVC | Girls | 5.00 | 101 | NO |  | 0.9335 | 0.04463 |  | 0.9335 | 0.8601 |
| FEV1/FVC | Girls | 5.00 | 102 | NO |  | 0.9333 | 0.04463 |  | 0.9333 | 0.8599 |
| FEV1/FVC | Girls | 5.00 | 103 | NO |  | 0.9330 | 0.04463 |  | 0.9330 | 0.8596 |
| FEV1/FVC | Girls | 5.00 | 104 | NO |  | 0.9328 | 0.04463 |  | 0.9328 | 0.8594 |
| FEV1/FVC | Girls | 5.00 | 105 | NO |  | 0.9326 | 0.04463 |  | 0.9326 | 0.8591 |
| FEV1/FVC | Girls | 5.00 | 106 | NO |  | 0.9323 | 0.04463 |  | 0.9323 | 0.8589 |
| FEV1/FVC | Girls | 5.00 | 107 | NO |  | 0.9321 | 0.04463 |  | 0.9321 | 0.8587 |
| FEV1/FVC | Girls | 5.00 | 108 | NO |  | 0.9319 | 0.04463 |  | 0.9319 | 0.8584 |
| FEV1/FVC | Girls | 5.00 | 109 | NO |  | 0.9316 | 0.04463 |  | 0.9316 | 0.8582 |
| FEV1/FVC | Girls | 5.00 | 110 | NO |  | 0.9314 | 0.04463 |  | 0.9314 | 0.8580 |
| FEV1/FVC | Girls | 5.00 | 111 | NO |  | 0.9312 | 0.04463 |  | 0.9312 | 0.8578 |
| FEV1/FVC | Girls | 5.00 | 112 | NO |  | 0.9310 | 0.04463 |  | 0.9310 | 0.8575 |
| FEV1/FVC | Girls | 5.00 | 113 | NO |  | 0.9307 | 0.04463 |  | 0.9307 | 0.8573 |
| FEV1/FVC | Girls | 5.00 | 114 | NO |  | 0.9305 | 0.04463 |  | 0.9305 | 0.8571 |
| FEV1/FVC | Girls | 5.00 | 115 | NO |  | 0.9303 | 0.04463 |  | 0.9303 | 0.8569 |
| FEV1/FVC | Girls | 5.00 | 116 | NO |  | 0.9301 | 0.04463 |  | 0.9301 | 0.8567 |
| FEV1/FVC | Girls | 5.00 | 117 | NO |  | 0.9299 | 0.04463 |  | 0.9299 | 0.8565 |
| FEV1/FVC | Girls | 5.00 | 118 | NO |  | 0.9297 | 0.04463 |  | 0.9297 | 0.8563 |
| FEV1/FVC | Girls | 5.00 | 119 | NO |  | 0.9295 | 0.04463 |  | 0.9295 | 0.8561 |
| FEV1/FVC | Girls | 5.00 | 120 | NO |  | 0.9293 | 0.04463 |  | 0.9293 | 0.8558 |
| FEV1/FVC | Girls | 5.00 | 121 | NO |  | 0.9291 | 0.04463 |  | 0.9291 | 0.8556 |
| FEV1/FVC | Girls | 5.00 | 122 | NO |  | 0.9289 | 0.04463 |  | 0.9289 | 0.8554 |
| FEV1/FVC | Girls | 5.00 | 123 | NO |  | 0.9287 | 0.04463 |  | 0.9287 | 0.8552 |
| FEV1/FVC | Girls | 5.00 | 124 | NO |  | 0.9285 | 0.04463 |  | 0.9285 | 0.8550 |
| FEV1/FVC | Girls | 5.25 | 88 | NO |  | 0.9368 | 0.04335 |  | 0.9368 | 0.8655 |
| FEV1/FVC | Girls | 5.25 | 89 | NO |  | 0.9365 | 0.04335 |  | 0.9365 | 0.8652 |
| FEV1/FVC | Girls | 5.25 | 90 | NO |  | 0.9363 | 0.04335 |  | 0.9363 | 0.8650 |
| FEV1/FVC | Girls | 5.25 | 91 | NO |  | 0.9360 | 0.04335 |  | 0.9360 | 0.8647 |
| FEV1/FVC | Girls | 5.25 | 92 | NO |  | 0.9357 | 0.04335 |  | 0.9357 | 0.8644 |
| FEV1/FVC | Girls | 5.25 | 93 | NO |  | 0.9354 | 0.04335 |  | 0.9354 | 0.8641 |
| FEV1/FVC | Girls | 5.25 | 94 | NO |  | 0.9352 | 0.04335 |  | 0.9352 | 0.8639 |
| FEV1/FVC | Girls | 5.25 | 95 | NO |  | 0.9349 | 0.04335 |  | 0.9349 | 0.8636 |
| FEV1/FVC | Girls | 5.25 | 96 | NO |  | 0.9347 | 0.04335 |  | 0.9347 | 0.8634 |
| FEV1/FVC | Girls | 5.25 | 97 | NO |  | 0.9344 | 0.04335 |  | 0.9344 | 0.8631 |
| FEV1/FVC | Girls | 5.25 | 98 | NO |  | 0.9341 | 0.04335 |  | 0.9341 | 0.8628 |
| FEV1/FVC | Girls | 5.25 | 99 | NO |  | 0.9339 | 0.04335 |  | 0.9339 | 0.8626 |
| FEV1/FVC | Girls | 5.25 | 100 | NO |  | 0.9336 | 0.04335 |  | 0.9336 | 0.8623 |
| FEV1/FVC | Girls | 5.25 | 101 | NO |  | 0.9334 | 0.04335 |  | 0.9334 | 0.8621 |
| FEV1/FVC | Girls | 5.25 | 102 | NO |  | 0.9332 | 0.04335 |  | 0.9332 | 0.8618 |
| FEV1/FVC | Girls | 5.25 | 103 | NO |  | 0.9329 | 0.04335 |  | 0.9329 | 0.8616 |
| FEV1/FVC | Girls | 5.25 | 104 | NO |  | 0.9327 | 0.04335 |  | 0.9327 | 0.8614 |
| FEV1/FVC | Girls | 5.25 | 105 | NO |  | 0.9324 | 0.04335 |  | 0.9324 | 0.8611 |
| FEV1/FVC | Girls | 5.25 | 106 | NO |  | 0.9322 | 0.04335 |  | 0.9322 | 0.8609 |
| FEV1/FVC | Girls | 5.25 | 107 | NO |  | 0.9320 | 0.04335 |  | 0.9320 | 0.8607 |
| FEV1/FVC | Girls | 5.25 | 108 | NO |  | 0.9317 | 0.04335 |  | 0.9317 | 0.8604 |
| FEV1/FVC | Girls | 5.25 | 109 | NO |  | 0.9315 | 0.04335 |  | 0.9315 | 0.8602 |
| FEV1/FVC | Girls | 5.25 | 110 | NO |  | 0.9313 | 0.04335 |  | 0.9313 | 0.8600 |
| FEV1/FVC | Girls | 5.25 | 111 | NO |  | 0.9311 | 0.04335 |  | 0.9311 | 0.8598 |
| FEV1/FVC | Girls | 5.25 | 112 | NO |  | 0.9308 | 0.04335 |  | 0.9308 | 0.8595 |
| FEV1/FVC | Girls | 5.25 | 113 | NO |  | 0.9306 | 0.04335 |  | 0.9306 | 0.8593 |
| FEV1/FVC | Girls | 5.25 | 114 | NO |  | 0.9304 | 0.04335 |  | 0.9304 | 0.8591 |
| FEV1/FVC | Girls | 5.25 | 115 | NO |  | 0.9302 | 0.04335 |  | 0.9302 | 0.8589 |
| FEV1/FVC | Girls | 5.25 | 116 | NO |  | 0.9300 | 0.04335 |  | 0.9300 | 0.8587 |
| FEV1/FVC | Girls | 5.25 | 117 | NO |  | 0.9298 | 0.04335 |  | 0.9298 | 0.8585 |
| FEV1/FVC | Girls | 5.25 | 118 | NO |  | 0.9296 | 0.04335 |  | 0.9296 | 0.8582 |
| FEV1/FVC | Girls | 5.25 | 119 | NO |  | 0.9294 | 0.04335 |  | 0.9294 | 0.8580 |
| FEV1/FVC | Girls | 5.25 | 120 | NO |  | 0.9291 | 0.04335 |  | 0.9291 | 0.8578 |
| FEV1/FVC | Girls | 5.25 | 121 | NO |  | 0.9289 | 0.04335 |  | 0.9289 | 0.8576 |
| FEV1/FVC | Girls | 5.25 | 122 | NO |  | 0.9287 | 0.04335 |  | 0.9287 | 0.8574 |
| FEV1/FVC | Girls | 5.25 | 123 | NO |  | 0.9285 | 0.04335 |  | 0.9285 | 0.8572 |
| FEV1/FVC | Girls | 5.25 | 124 | NO |  | 0.9283 | 0.04335 |  | 0.9283 | 0.8570 |
| FEV1/FVC | Girls | 5.50 | 88 | NO |  | 0.9367 | 0.04219 |  | 0.9367 | 0.8673 |
| FEV1/FVC | Girls | 5.50 | 89 | NO |  | 0.9364 | 0.04219 |  | 0.9364 | 0.8670 |
| FEV1/FVC | Girls | 5.50 | 90 | NO |  | 0.9361 | 0.04219 |  | 0.9361 | 0.8667 |
| FEV1/FVC | Girls | 5.50 | 91 | NO |  | 0.9359 | 0.04219 |  | 0.9359 | 0.8665 |
| FEV1/FVC | Girls | 5.50 | 92 | NO |  | 0.9356 | 0.04219 |  | 0.9356 | 0.8662 |
| FEV1/FVC | Girls | 5.50 | 93 | NO |  | 0.9353 | 0.04219 |  | 0.9353 | 0.8659 |
| FEV1/FVC | Girls | 5.50 | 94 | NO |  | 0.9351 | 0.04219 |  | 0.9351 | 0.8657 |
| FEV1/FVC | Girls | 5.50 | 95 | NO |  | 0.9348 | 0.04219 |  | 0.9348 | 0.8654 |
| FEV1/FVC | Girls | 5.50 | 96 | NO |  | 0.9345 | 0.04219 |  | 0.9345 | 0.8651 |
| FEV1/FVC | Girls | 5.50 | 97 | NO |  | 0.9343 | 0.04219 |  | 0.9343 | 0.8649 |
| FEV1/FVC | Girls | 5.50 | 98 | NO |  | 0.9340 | 0.04219 |  | 0.9340 | 0.8646 |
| FEV1/FVC | Girls | 5.50 | 99 | NO |  | 0.9338 | 0.04219 |  | 0.9338 | 0.8644 |
| FEV1/FVC | Girls | 5.50 | 100 | NO |  | 0.9335 | 0.04219 |  | 0.9335 | 0.8641 |
| FEV1/FVC | Girls | 5.50 | 101 | NO |  | 0.9333 | 0.04219 |  | 0.9333 | 0.8639 |
| FEV1/FVC | Girls | 5.50 | 102 | NO |  | 0.9330 | 0.04219 |  | 0.9330 | 0.8636 |
| FEV1/FVC | Girls | 5.50 | 103 | NO |  | 0.9328 | 0.04219 |  | 0.9328 | 0.8634 |
| FEV1/FVC | Girls | 5.50 | 104 | NO |  | 0.9325 | 0.04219 |  | 0.9325 | 0.8631 |
| FEV1/FVC | Girls | 5.50 | 105 | NO |  | 0.9323 | 0.04219 |  | 0.9323 | 0.8629 |
| FEV1/FVC | Girls | 5.50 | 106 | NO |  | 0.9321 | 0.04219 |  | 0.9321 | 0.8627 |
| FEV1/FVC | Girls | 5.50 | 107 | NO |  | 0.9318 | 0.04219 |  | 0.9318 | 0.8624 |
| FEV1/FVC | Girls | 5.50 | 108 | NO |  | 0.9316 | 0.04219 |  | 0.9316 | 0.8622 |
| FEV1/FVC | Girls | 5.50 | 109 | NO |  | 0.9314 | 0.04219 |  | 0.9314 | 0.8620 |
| FEV1/FVC | Girls | 5.50 | 110 | NO |  | 0.9312 | 0.04219 |  | 0.9312 | 0.8618 |
| FEV1/FVC | Girls | 5.50 | 111 | NO |  | 0.9309 | 0.04219 |  | 0.9309 | 0.8615 |
| FEV1/FVC | Girls | 5.50 | 112 | NO |  | 0.9307 | 0.04219 |  | 0.9307 | 0.8613 |
| FEV1/FVC | Girls | 5.50 | 113 | NO |  | 0.9305 | 0.04219 |  | 0.9305 | 0.8611 |
| FEV1/FVC | Girls | 5.50 | 114 | NO |  | 0.9303 | 0.04219 |  | 0.9303 | 0.8609 |
| FEV1/FVC | Girls | 5.50 | 115 | NO |  | 0.9301 | 0.04219 |  | 0.9301 | 0.8607 |
| FEV1/FVC | Girls | 5.50 | 116 | NO |  | 0.9298 | 0.04219 |  | 0.9298 | 0.8605 |
| FEV1/FVC | Girls | 5.50 | 117 | NO |  | 0.9296 | 0.04219 |  | 0.9296 | 0.8602 |
| FEV1/FVC | Girls | 5.50 | 118 | NO |  | 0.9294 | 0.04219 |  | 0.9294 | 0.8600 |
| FEV1/FVC | Girls | 5.50 | 119 | NO |  | 0.9292 | 0.04219 |  | 0.9292 | 0.8598 |
| FEV1/FVC | Girls | 5.50 | 120 | NO |  | 0.9290 | 0.04219 |  | 0.9290 | 0.8596 |
| FEV1/FVC | Girls | 5.50 | 121 | NO |  | 0.9288 | 0.04219 |  | 0.9288 | 0.8594 |
| FEV1/FVC | Girls | 5.50 | 122 | NO |  | 0.9286 | 0.04219 |  | 0.9286 | 0.8592 |
| FEV1/FVC | Girls | 5.50 | 123 | NO |  | 0.9284 | 0.04219 |  | 0.9284 | 0.8590 |
| FEV1/FVC | Girls | 5.50 | 124 | NO |  | 0.9282 | 0.04219 |  | 0.9282 | 0.8588 |
| FEV1/FVC | Girls | 5.75 | 88 | NO |  | 0.9366 | 0.04112 |  | 0.9366 | 0.8689 |
| FEV1/FVC | Girls | 5.75 | 89 | NO |  | 0.9363 | 0.04112 |  | 0.9363 | 0.8686 |
| FEV1/FVC | Girls | 5.75 | 90 | NO |  | 0.9360 | 0.04112 |  | 0.9360 | 0.8684 |
| FEV1/FVC | Girls | 5.75 | 91 | NO |  | 0.9357 | 0.04112 |  | 0.9357 | 0.8681 |
| FEV1/FVC | Girls | 5.75 | 92 | NO |  | 0.9354 | 0.04112 |  | 0.9354 | 0.8678 |
| FEV1/FVC | Girls | 5.75 | 93 | NO |  | 0.9352 | 0.04112 |  | 0.9352 | 0.8675 |
| FEV1/FVC | Girls | 5.75 | 94 | NO |  | 0.9349 | 0.04112 |  | 0.9349 | 0.8673 |
| FEV1/FVC | Girls | 5.75 | 95 | NO |  | 0.9347 | 0.04112 |  | 0.9347 | 0.8670 |
| FEV1/FVC | Girls | 5.75 | 96 | NO |  | 0.9344 | 0.04112 |  | 0.9344 | 0.8668 |
| FEV1/FVC | Girls | 5.75 | 97 | NO |  | 0.9341 | 0.04112 |  | 0.9341 | 0.8665 |
| FEV1/FVC | Girls | 5.75 | 98 | NO |  | 0.9339 | 0.04112 |  | 0.9339 | 0.8662 |
| FEV1/FVC | Girls | 5.75 | 99 | NO |  | 0.9336 | 0.04112 |  | 0.9336 | 0.8660 |
| FEV1/FVC | Girls | 5.75 | 100 | NO |  | 0.9334 | 0.04112 |  | 0.9334 | 0.8657 |
| FEV1/FVC | Girls | 5.75 | 101 | NO |  | 0.9331 | 0.04112 |  | 0.9331 | 0.8655 |
| FEV1/FVC | Girls | 5.75 | 102 | NO |  | 0.9329 | 0.04112 |  | 0.9329 | 0.8653 |
| FEV1/FVC | Girls | 5.75 | 103 | NO |  | 0.9327 | 0.04112 |  | 0.9327 | 0.8650 |
| FEV1/FVC | Girls | 5.75 | 104 | NO |  | 0.9324 | 0.04112 |  | 0.9324 | 0.8648 |
| FEV1/FVC | Girls | 5.75 | 105 | NO |  | 0.9322 | 0.04112 |  | 0.9322 | 0.8645 |
| FEV1/FVC | Girls | 5.75 | 106 | NO |  | 0.9319 | 0.04112 |  | 0.9319 | 0.8643 |
| FEV1/FVC | Girls | 5.75 | 107 | NO |  | 0.9317 | 0.04112 |  | 0.9317 | 0.8641 |
| FEV1/FVC | Girls | 5.75 | 108 | NO |  | 0.9315 | 0.04112 |  | 0.9315 | 0.8638 |
| FEV1/FVC | Girls | 5.75 | 109 | NO |  | 0.9313 | 0.04112 |  | 0.9313 | 0.8636 |
| FEV1/FVC | Girls | 5.75 | 110 | NO |  | 0.9310 | 0.04112 |  | 0.9310 | 0.8634 |
| FEV1/FVC | Girls | 5.75 | 111 | NO |  | 0.9308 | 0.04112 |  | 0.9308 | 0.8632 |
| FEV1/FVC | Girls | 5.75 | 112 | NO |  | 0.9306 | 0.04112 |  | 0.9306 | 0.8629 |
| FEV1/FVC | Girls | 5.75 | 113 | NO |  | 0.9304 | 0.04112 |  | 0.9304 | 0.8627 |
| FEV1/FVC | Girls | 5.75 | 114 | NO |  | 0.9301 | 0.04112 |  | 0.9301 | 0.8625 |
| FEV1/FVC | Girls | 5.75 | 115 | NO |  | 0.9299 | 0.04112 |  | 0.9299 | 0.8623 |
| FEV1/FVC | Girls | 5.75 | 116 | NO |  | 0.9297 | 0.04112 |  | 0.9297 | 0.8621 |
| FEV1/FVC | Girls | 5.75 | 117 | NO |  | 0.9295 | 0.04112 |  | 0.9295 | 0.8619 |
| FEV1/FVC | Girls | 5.75 | 118 | NO |  | 0.9293 | 0.04112 |  | 0.9293 | 0.8617 |
| FEV1/FVC | Girls | 5.75 | 119 | NO |  | 0.9291 | 0.04112 |  | 0.9291 | 0.8614 |
| FEV1/FVC | Girls | 5.75 | 120 | NO |  | 0.9289 | 0.04112 |  | 0.9289 | 0.8612 |
| FEV1/FVC | Girls | 5.75 | 121 | NO |  | 0.9287 | 0.04112 |  | 0.9287 | 0.8610 |
| FEV1/FVC | Girls | 5.75 | 122 | NO |  | 0.9285 | 0.04112 |  | 0.9285 | 0.8608 |
| FEV1/FVC | Girls | 5.75 | 123 | NO |  | 0.9283 | 0.04112 |  | 0.9283 | 0.8606 |
| FEV1/FVC | Girls | 5.75 | 124 | NO |  | 0.9281 | 0.04112 |  | 0.9281 | 0.8604 |
| FEV1/FVC | Girls | 6.00 | 88 | NO |  | 0.9364 | 0.04013 |  | 0.9364 | 0.8704 |
| FEV1/FVC | Girls | 6.00 | 89 | NO |  | 0.9361 | 0.04013 |  | 0.9361 | 0.8701 |
| FEV1/FVC | Girls | 6.00 | 90 | NO |  | 0.9359 | 0.04013 |  | 0.9359 | 0.8698 |
| FEV1/FVC | Girls | 6.00 | 91 | NO |  | 0.9356 | 0.04013 |  | 0.9356 | 0.8696 |
| FEV1/FVC | Girls | 6.00 | 92 | NO |  | 0.9353 | 0.04013 |  | 0.9353 | 0.8693 |
| FEV1/FVC | Girls | 6.00 | 93 | NO |  | 0.9350 | 0.04013 |  | 0.9350 | 0.8690 |
| FEV1/FVC | Girls | 6.00 | 94 | NO |  | 0.9348 | 0.04013 |  | 0.9348 | 0.8688 |
| FEV1/FVC | Girls | 6.00 | 95 | NO |  | 0.9345 | 0.04013 |  | 0.9345 | 0.8685 |
| FEV1/FVC | Girls | 6.00 | 96 | NO |  | 0.9343 | 0.04013 |  | 0.9343 | 0.8682 |
| FEV1/FVC | Girls | 6.00 | 97 | NO |  | 0.9340 | 0.04013 |  | 0.9340 | 0.8680 |
| FEV1/FVC | Girls | 6.00 | 98 | NO |  | 0.9337 | 0.04013 |  | 0.9337 | 0.8677 |
| FEV1/FVC | Girls | 6.00 | 99 | NO |  | 0.9335 | 0.04013 |  | 0.9335 | 0.8675 |
| FEV1/FVC | Girls | 6.00 | 100 | NO |  | 0.9332 | 0.04013 |  | 0.9332 | 0.8672 |
| FEV1/FVC | Girls | 6.00 | 101 | NO |  | 0.9330 | 0.04013 |  | 0.9330 | 0.8670 |
| FEV1/FVC | Girls | 6.00 | 102 | NO |  | 0.9328 | 0.04013 |  | 0.9328 | 0.8667 |
| FEV1/FVC | Girls | 6.00 | 103 | NO |  | 0.9325 | 0.04013 |  | 0.9325 | 0.8665 |
| FEV1/FVC | Girls | 6.00 | 104 | NO |  | 0.9323 | 0.04013 |  | 0.9323 | 0.8663 |
| FEV1/FVC | Girls | 6.00 | 105 | NO |  | 0.9320 | 0.04013 |  | 0.9320 | 0.8660 |
| FEV1/FVC | Girls | 6.00 | 106 | NO |  | 0.9318 | 0.04013 |  | 0.9318 | 0.8658 |
| FEV1/FVC | Girls | 6.00 | 107 | NO |  | 0.9316 | 0.04013 |  | 0.9316 | 0.8656 |
| FEV1/FVC | Girls | 6.00 | 108 | NO |  | 0.9313 | 0.04013 |  | 0.9313 | 0.8653 |
| FEV1/FVC | Girls | 6.00 | 109 | NO |  | 0.9311 | 0.04013 |  | 0.9311 | 0.8651 |
| FEV1/FVC | Girls | 6.00 | 110 | NO |  | 0.9309 | 0.04013 |  | 0.9309 | 0.8649 |
| FEV1/FVC | Girls | 6.00 | 111 | NO |  | 0.9307 | 0.04013 |  | 0.9307 | 0.8647 |
| FEV1/FVC | Girls | 6.00 | 112 | NO |  | 0.9304 | 0.04013 |  | 0.9304 | 0.8644 |
| FEV1/FVC | Girls | 6.00 | 113 | NO |  | 0.9302 | 0.04013 |  | 0.9302 | 0.8642 |
| FEV1/FVC | Girls | 6.00 | 114 | NO |  | 0.9300 | 0.04013 |  | 0.9300 | 0.8640 |
| FEV1/FVC | Girls | 6.00 | 115 | NO |  | 0.9298 | 0.04013 |  | 0.9298 | 0.8638 |
| FEV1/FVC | Girls | 6.00 | 116 | NO |  | 0.9296 | 0.04013 |  | 0.9296 | 0.8636 |
| FEV1/FVC | Girls | 6.00 | 117 | NO |  | 0.9294 | 0.04013 |  | 0.9294 | 0.8634 |
| FEV1/FVC | Girls | 6.00 | 118 | NO |  | 0.9292 | 0.04013 |  | 0.9292 | 0.8631 |
| FEV1/FVC | Girls | 6.00 | 119 | NO |  | 0.9289 | 0.04013 |  | 0.9289 | 0.8629 |
| FEV1/FVC | Girls | 6.00 | 120 | NO |  | 0.9287 | 0.04013 |  | 0.9287 | 0.8627 |
| FEV1/FVC | Girls | 6.00 | 121 | NO |  | 0.9285 | 0.04013 |  | 0.9285 | 0.8625 |
| FEV1/FVC | Girls | 6.00 | 122 | NO |  | 0.9283 | 0.04013 |  | 0.9283 | 0.8623 |
| FEV1/FVC | Girls | 6.00 | 123 | NO |  | 0.9281 | 0.04013 |  | 0.9281 | 0.8621 |
| FEV1/FVC | Girls | 6.00 | 124 | NO |  | 0.9279 | 0.04013 |  | 0.9279 | 0.8619 |

## Table S1. Calibration of Jordanian equations in the Al-Qerem and Jarab validation cohort

| Outcome | Sex | n | Intercept | 95% CI (low) | 95% CI (high) | Slope | 95% CI (low) | 95% CI (high) | BA bias (95% LoA) |
| --- | --- | --- | --- | --- | --- | --- | --- | --- | --- |
| FEV1 | Boys | 400 | 0.030 | -0.045 | 0.105 | 0.965 | 0.896 | 1.034 | 0.007 (-0.256 to 0.270) |
|  | Girls | 365 | -0.015 | -0.103 | 0.073 | 1.010 | 0.925 | 1.094 | 0.005 (-0.254 to 0.264) |
| FVC | Boys | 400 | 0.093 | 0.016 | 0.170 | 0.908 | 0.841 | 0.974 | 0.013 (-0.269 to 0.295) |
|  | Girls | 365 | 0.019 | -0.076 | 0.115 | 0.990 | 0.903 | 1.077 | -0.008 (-0.298 to 0.282) |
| FEV1/FVC | Boys | 400 | 1.006 | 0.702 | 1.310 | -0.073 | -0.400 | 0.254 | -0.009 (-0.100 to 0.082) |
|  | Girls | 365 | -0.244 | -3.226 | 2.738 | 1.264 | -1.937 | 4.465 | -0.002 (-0.091 to 0.088) |

**Abbreviations:** FEV₁, forced expiratory volume in 1 second; FVC, forced vital capacity; FEV₁/FVC, ratio of FEV₁ to FVC; CI, confidence interval; n, number. BA, Bland-Altman; LoA, limits of agreement. BA bias was calculated as predicted minus raw measured value.

## Table S2. External validation: comparison of Jordanian, GLI 2022 race-neutral, and GLI 2012 Caucasian reference equations in the Al-Qerem and Jarab cohort

| Outcome | Sex | Reference system | n | z-score, M (SD) [95% CI] | % predicted, M (SD) [95% CI] | % < LLN (z < -1.645) [95% CI] |
| --- | --- | --- | --- | --- | --- | --- |
| FEV1 | Boys | GLI 2012 Caucasian | 400 | 0.00 (0.96) [-0.09 to 0.10] | 100.0 (12.5) [98.8 to 101.2] | 4.8 [2.9 to 7.3] |
|  |  | GLI 2022 race-neutral | 400 | 0.25 (0.98) [0.15 to 0.34] | 103.1 (13.0) [101.8 to 104.4] | 2.8 [1.4 to 4.9] |
|  |  | Jordanian | 400 | 0.08 (1.00) [-0.02 to 0.18] | 99.4 (12.4) [98.2 to 100.6] | 3.5 [1.9 to 5.8] |
|  | Girls | GLI 2012 Caucasian | 365 | 0.01 (0.95) [-0.09 to 0.11] | 100.0 (12.6) [98.7 to 101.3] | 4.4 [2.5 to 7.0] |
|  |  | GLI 2022 race-neutral | 365 | 0.29 (0.96) [0.19 to 0.39] | 103.8 (13.1) [102.4 to 105.1] | 1.9 [0.8 to 3.9] |
|  |  | Jordanian | 365 | 0.12 (1.15) [0.00 to 0.24] | 99.5 (12.6) [98.2 to 100.8] | 4.4 [2.5 to 7.0] |
| FEV1/FVC | Boys | GLI 2012 Caucasian | 400 | 0.26 (0.84) [0.18 to 0.34] | 101.2 (5.2) [100.7 to 101.7] | 3.5 [1.9 to 5.8] |
|  |  | GLI 2022 race-neutral | 400 | 0.22 (0.83) [0.13 to 0.30] | 101.1 (5.3) [100.6 to 101.6] | 3.5 [1.9 to 5.8] |
|  |  | Jordanian | 400 | 0.29 (1.00) [0.19 to 0.39] | 101.0 (5.0) [100.5 to 101.5] | 3.5 [1.9 to 5.8] |
|  | Girls | GLI 2012 Caucasian | 365 | -0.01 (0.89) [-0.11 to 0.08] | 99.4 (5.0) [98.9 to 100.0] | 4.9 [2.9 to 7.7] |
|  |  | GLI 2022 race-neutral | 365 | -0.03 (0.89) [-0.13 to 0.06] | 99.3 (5.1) [98.8 to 99.8] | 4.9 [2.9 to 7.7] |
|  |  | Jordanian | 365 | 0.04 (0.89) [-0.05 to 0.14] | 100.2 (4.9) [99.7 to 100.7] | 1.4 [0.4 to 3.2] |
| FVC | Boys | GLI 2012 Caucasian | 400 | -0.13 (0.91) [-0.22 to -0.04] | 98.3 (12.4) [97.1 to 99.5] | 5.5 [3.5 to 8.2] |
|  |  | GLI 2022 race-neutral | 400 | 0.06 (0.95) [-0.03 to 0.15] | 100.9 (12.9) [99.6 to 102.1] | 3.8 [2.1 to 6.1] |
|  |  | Jordanian | 400 | -0.06 (0.95) [-0.15 to 0.03] | 99.1 (12.3) [97.9 to 100.3] | 5.8 [3.7 to 8.5] |
|  | Girls | GLI 2012 Caucasian | 365 | 0.02 (0.96) [-0.08 to 0.12] | 100.5 (13.3) [99.1 to 101.8] | 4.4 [2.5 to 7.0] |
|  |  | GLI 2022 race-neutral | 365 | 0.28 (1.00) [0.18 to 0.38] | 104.1 (14.0) [102.6 to 105.5] | 2.7 [1.3 to 5.0] |
|  |  | Jordanian | 365 | 0.05 (0.98) [-0.05 to 0.15] | 100.8 (13.4) [99.4 to 102.2] | 3.8 [2.1 to 6.4] |

**Abbreviations:** FEV₁, forced expiratory volume in 1 second; FVC, forced vital capacity; FEV₁/FVC, ratio of FEV₁ to FVC; GLI, Global Lung Initiative; M, mean; SD, standard deviation; LLN, lower limit of normal; CI, confidence interval; n, number.
